# A proteomic approach reveals integrin activation state-dependent control of microtubule cortical targeting

**DOI:** 10.1038/ncomms7135

**Published:** 2015-01-22

**Authors:** Adam Byron, Janet A. Askari, Jonathan D. Humphries, Guillaume Jacquemet, Ewa J. Koper, Stacey Warwood, Colin K. Choi, Matthew J. Stroud, Christopher S. Chen, David Knight, Martin J. Humphries

**Affiliations:** 1 Wellcome Trust Centre for Cell-Matrix Research, Faculty of Life Sciences, University of Manchester, Manchester M13 9PT, UK; 2 Biological Mass Spectrometry Core Facility, Faculty of Life Sciences, University of Manchester, Manchester M13 9PT, UK; 3 Department of Biomedical Engineering, Boston University, Boston, Massachusetts 02215, USA; 4 Wyss Institute for Biologically Inspired Engineering, Harvard University, Boston, Massachusetts 02115, USA

## Abstract

Integrin activation, which is regulated by allosteric changes in receptor conformation, enables cellular responses to the chemical, mechanical and topological features of the extracellular microenvironment. A global view of how activation state converts the molecular composition of the region proximal to integrins into functional readouts is, however, lacking. Here, using conformation-specific monoclonal antibodies, we report the isolation of integrin activation state-dependent complexes and their characterization by mass spectrometry. Quantitative comparisons, integrating network, clustering, pathway and image analyses, define multiple functional protein modules enriched in a conformation-specific manner. Notably, active integrin complexes are specifically enriched for proteins associated with microtubule-based functions. Visualization of microtubules on micropatterned surfaces and live cell imaging demonstrate that active integrins establish an environment that stabilizes microtubules at the cell periphery. These data provide a resource for the interrogation of the global molecular connections that link integrin activation to adhesion signalling.

## Abstract (web summary)

Integrins are activated by many extracellular cues and respond by assembling diverse signalling complexes. Byron *et al.* use activation state-specific antibodies to proteomically characterize these complexes, and provide insight into integrin-dependent microtubule stabilization.

## Graphical abstract

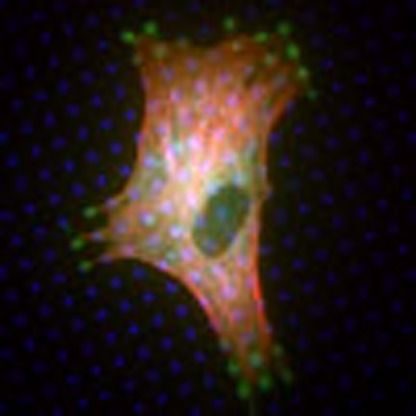

Integrins are a family of heterodimeric cell surface receptors that make essential contributions to both cell–extracellular matrix and cell–cell interactions. Integrin signal transduction influences cell morphology, migration, survival and differentiation in a multitude of developmental, homoeostatic and disease processes^1^,^2^. Integrin function is mediated by the tethering of extracellular ligands to the intracellular cytoskeleton, which in turn creates a spatially heterogeneous platform for the assembly of adhesion signalling complexes. Based on literature curation, it has been suggested that the molecular composition of these complexes comprises over 200 components, collectively referred to as the integrin adhesome^3^,^4^. Recent applications of techniques such as super-resolution microscopy^5^,^6^ and mass spectrometry (MS)-based proteomics^7^,^8^,^9^,^10^,^11^ have generated new insights into the complexity, composition, organization and mechanisms of regulation of adhesion complexes.

Current models of integrin activation state regulation incorporate three main conformational classes, corresponding to ligand-bound, active (or primed) and inactive receptor^12^. These distinct conformer classes exist in a dynamic equilibrium that can be modulated both from outside the cell by extracellular protein ligands and/or divalent cations (outside-in regulation) and from inside the cell by proteins, such as talin, that bind to integrin cytoplasmic tails (inside-out regulation)^1^,^13^. The interactions of integrin cytoplasmic domains with the cytoskeletal, adaptor and signalling molecules of the adhesome are complex and central to regulation of integrin-mediated cellular functions^14^. The process of integrin activation has been well studied, with talin having a well-characterized role in the final step, and more recently identified players, such as kindlins, acting as activity modulators^15^,^16^. There is also growing evidence that integrin inactivation, rather than being a default state, is positively regulated by the binding of other molecules, for example, ICAP-1 and SHARPIN^17^.

Monoclonal antibodies (mAbs) can also regulate integrin affinity, as they recognize epitopes exposed on integrins undergoing activation state-dependent conformational changes^18^. The study of integrin function has been greatly aided by the use of such reagents, as they can both report and induce a particular integrin activation state by causing a shift in the receptor conformational equilibrium. In addition, a large majority of activation state-specific mAbs act as allosteric agonists or antagonists and do not directly interfere with or compete for ligand binding^18^. Thus, stimulatory anti-integrin mAbs stabilize a receptor conformation that is competent to bind ligand and thereby activate integrin function. In contrast, inhibitory mAbs stabilize an integrin conformation that is unable to bind ligand and thus abrogate integrin-mediated functions.

We hypothesized that integrin activation state determines the intracellular molecular environment of integrins. We therefore developed a methodology for the systems-based analysis of activation state-dependent integrin proteomes. Here, we report marked differences in the protein composition of active and inactive integrin complexes and differential enrichment of specific functional groups of proteins. Microtubule plus-end tracking proteins (+TIPs) are enriched in adhesion complexes associated with active β1 integrins. Functional analyses reveal that integrin activation state determines cortical targeting of microtubules by establishing an environment that regulates microtubule stability at the cell periphery. Our work provides insights into the complexity of integrin signalling and the specificity of cellular processes that are dictated by integrin activation state. Moreover, this data resource primes further investigations into the molecular connections linking integrin activation state signalling and cell function.

## Results

### Integrin activation state directs adhesion complex formation

**Figure 1:**
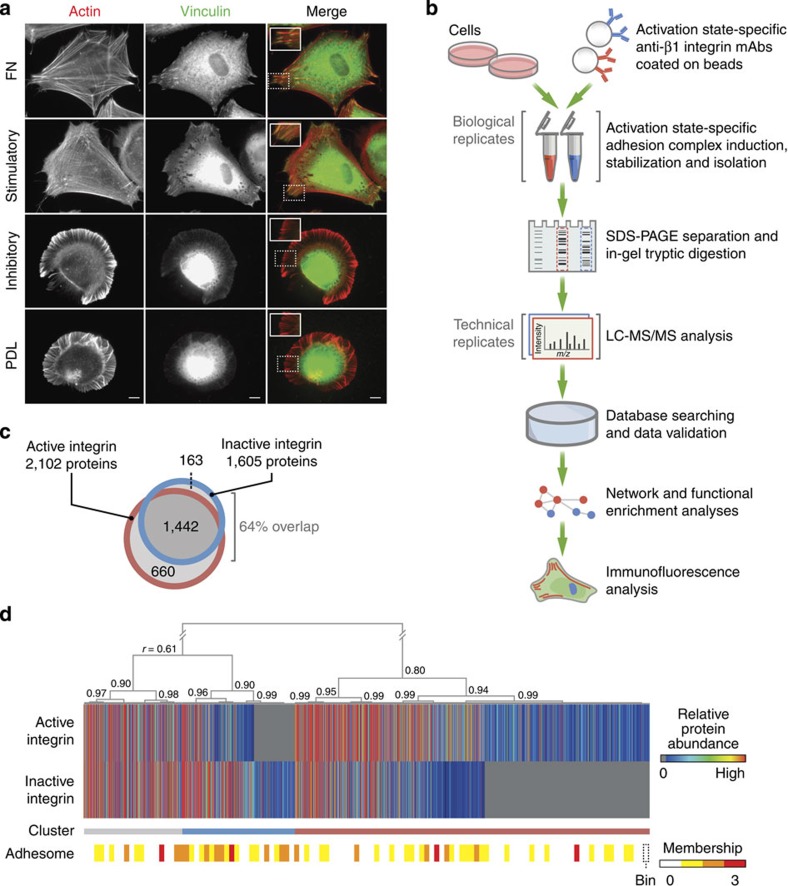
Proteomic analysis of integrin activation state-dependent adhesion complexes. (**a**) Immunofluorescence microscopy revealed the morphology of HFF cells spread on stimulatory and inhibitory anti-β1 integrin mAbs compared with those spread on FN and PDL. Cells were stained for actin (red) and vinculin (green). Scale bar, 10 μm. Inset images correspond to areas highlighted in white dotted boxes. (**b**) Workflow for the isolation and proteomic analysis of integrin activation state-dependent adhesion complexes from K562 cells using paramagnetic beads coated with activation state-specific anti-β1 integrin mAbs. The mAb-coated beads recruited integrins and associated proteins in live cells, and complexes were then stabilized with crosslinker and crosslinks cleaved under reducing conditions during extraction. Proteins were then separated by SDS–PAGE, and the whole lane was cut into 30 slices, which were subjected to in-gel trypsin digestion for analysis by MS. MS data for each adhesion complex isolation were acquired in technical duplicate, from duplicate biological isolations. (**c**) The distribution of proteins identified in active and inactive integrin data sets illustrated as a Venn diagram. (**d**) Hierarchical clustering analysis of the quantitative MS data. Pearson correlation coefficients (*r*) are indicated at dendrogram nodes; a threshold of *r*≥0.80 was used to identify clusters of distinct protein enrichment (red, active integrin; blue, inactive integrin; grey, unenriched). Accompanying heat bar (bottom) indicates the distribution of reported adhesome components^4^. Bin, 20 proteins.

To assess directly the role of integrin activity in the formation and composition of adhesion complexes, human foreskin fibroblasts (HFFs) were spread on immobilized, activation state-specific anti-β1 integrin mAbs^18^. The canonical integrin ligand fibronectin (FN) and the amino acid polymer poly-D-lysine (PDL) were used as positive and negative controls, respectively, for integrin-mediated adhesion complex formation. HFFs spread on both stimulatory and inhibitory mAbs to the same extent as on FN (Supplementary Fig. 1), but exhibited distinct morphological differences. Cells with integrins constrained in an active state produced organized stress fibres and recruited vinculin into adhesion complex-like clusters at the cell edge, resembling cells spread on FN (Fig. 1a). In contrast, cells with integrins constrained in an inactive conformation exhibited a more rounded morphology with few organized actin stress fibres, but with pronounced actin ruffles containing abundant microspikes around the cell periphery (Fig. 1a). In addition, these cells did not accumulate vinculin in adhesion complexes, and their morphology was reminiscent of HFFs spread on PDL, where integrins are not engaged. These results support the hypothesis that integrin activation state determines the composition of the local intracellular environment of adhesion complexes.

### Proteomic analysis of integrin activation state complexes

To identify compositional differences between protein complexes associated with active and inactive integrin, we adapted our previously described ligand affinity purification approach^7^,^19^ (Fig. 1b). Integrin-associated complexes were isolated from K562 cells using paramagnetic beads coated with the activation state-specific anti-β1 integrin mAbs used above. The mAb-coated beads recruited integrins and associated proteins in live cells, and complexes were then stabilized with crosslinker, extracted, purified and analysed by MS.

Proteomic analysis identified 2,265 proteins with at least 99% confidence (Supplementary Data 1). Label-free quantification demonstrated good reproducibility between data from technical and biological replicates, with similar distributions of protein abundance between activation states (Supplementary Fig. 2). There was considerable overlap (1,442 proteins; 64%) between proteins identified in complexes associated with active and inactive integrin (Fig. 1c). There were also a number of proteins detected in distinct types of complex, with 660 proteins (29%) unique to complexes associated with active β1 integrin and 163 proteins (7%) unique to those associated with inactive β1 integrin (Fig. 1c).

To examine the patterns of protein enrichment, hierarchical clustering was performed on the quantitative MS data, which organized proteins according to the similarity of their enrichment in both data sets. Importantly, both talin and kindlin, proteins known to induce integrin activation^16^, were highly enriched in active β1 integrin complexes (red cluster, Fig. 1d and Supplementary Table 1), demonstrating the validity of the data set. Interrogation of the clusters identified other well-characterized adhesome components that were enriched in either active integrin complexes (including vinculin, α-actinin, filamins, ezrin, moesin, Crk-like protein, vasodilator-stimulated phosphoprotein, liprin-α1, ACF7 and Arp2/3 complex subunits; red cluster, Fig. 1d, Supplementary Fig. 3 and Supplementary Table 1) or inactive integrin complexes (including calpain subunits, the LIM-domain protein thyroid receptor-interacting protein 6 and the small GTPases RhoA, RhoC and RhoG; blue cluster, Fig. 1d, Supplementary Fig. 3 and Supplementary Table 1). In keeping with the observations made by immunofluorescence (IF; Fig. 1a), these data demonstrate qualitative and quantitative differences between the composition of protein complexes within the environment of active and inactive integrin.

Of the 232 proteins reported in the current literature-curated adhesome inventory^4^, 77 (33%) were present in the integrin activation state data sets. This coverage of possible adhesome components is consistent with other proteomic studies^20^ and likely reflects the wide range of cell systems that were used to construct the original adhesome. Intrinsic adhesome components reported to reside within adhesion sites (core components) and transiently or peripherally associated adhesome components were represented to a similar extent in the MS data (31 and 37% of the respective adhesome divisions; Supplementary Fig. 3). Of the identified adhesome components, a similar proportion of core components was enriched in active and inactive integrin complexes (65% and 61%, respectively, compared with 64% reported in the reference adhesome; Supplementary Fig. 3). In contrast, adhesome components with similar abundance in both data sets (‘unenriched’, grey cluster, Fig. 1d and Supplementary Fig. 3) consisted of a smaller proportion of core components (42%). This may reflect a relatively transient or peripherally associated set of proteins recruited to integrin complexes in an activation state-independent manner.

### Integrin activation state determines the adhesion landscape

**Figure 2:**
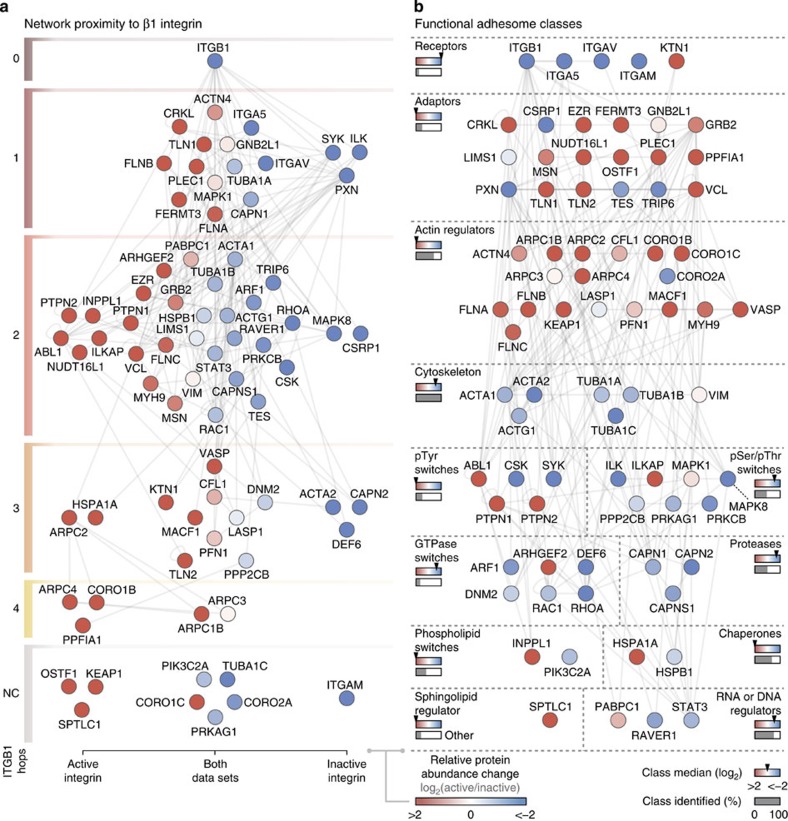
Analysis of the activation state-dependent adhesome network. (**a**) The interaction network of adhesome components^4^ identified by MS was arranged according to the number of reported protein interactions (hops) from β1 integrin (ITGB1). Proteins were clustered by their detection in active (left), inactive (right) or both (middle) integrin data sets. NC, not connected. (**b**) The identified adhesome network was arranged according to functional class^3^,^4^. Arrowheads on coloured bars indicate median protein enrichment for each class; grey bars indicate proportion of reported adhesome class identified by MS. Sphingolipid regulator quantification was derived from ‘other’ adhesome class. ‘Channel’ and ‘E3 ligase’ adhesome classes were not represented (0% identified). Nodes (proteins) are coloured according to their enrichment in active (red) or inactive (blue) integrin complexes (log_2_ transformed). Gene symbols are shown for clarity (see Supplementary Table 1 for protein names).

To place the identified proteins in the context of known protein–protein interactions, we constructed activation state-dependent integrin interactomes based on the quantitative MS data (Supplementary Figs 4 and 5). Network analysis of identified adhesome components showed similar numbers of proteins enriched in active and inactive integrin complexes at progressive interaction distances (hops) from β1 integrin, suggesting little influence of activation state on the network proximity (directness of interaction) of adhesome components to integrin (Fig. 2a). Interestingly, this highlights the capacity of inactive integrin to recruit complexes of proteins comprising some distally connected components of the adhesome. Analysis of the functional classes of adhesome proteins^3^,^4^ demonstrated that at least 40% of each of the classes of cytoskeletal proteins, actin regulators, pSer/pThr switches (kinases and phosphatases), phospholipid switches (phosphatidylinositol kinases and phosphatases), proteases, chaperones and RNA or DNA regulators were identified in the data sets (Fig. 2b). Although a major functional class in the proteomic data sets, a relatively small proportion (24%) of the large class of adhesome adaptors was identified, suggesting that these proteins may contribute to the specificity of distinct adhesion complexes. Furthermore, identified adaptors and actin regulators were enriched in active integrin complexes, whereas identified cytoskeletal proteins, GTPase switches (GTPases, GTPase-activating proteins and guanine nucleotide exchange factors) and proteases were enriched in inactive integrin complexes (Fig. 2b).

To determine the overrepresentation of proteins with certain functions in each data set, enrichment statistics were calculated for the proteins assigned to the active and inactive integrin clusters (Supplementary Table 2). Of the functional classes of adhesome components, cytoskeleton, actin-binding and microtubule terms were significantly overrepresented in the cluster of active integrin-enriched proteins. In contrast, kinase and GTP-binding terms were significantly overrepresented in the inactive integrin cluster (Supplementary Table 2). This suggests that whereas active integrins form protein complexes that associate to a greater extent with the actin and microtubule cytoskeleton, inactive integrin complexes may be more prominently involved with phosphorylation and GTPase regulation. Notably, the Rho and Ras GTPase family members RhoA, RhoC, RhoG, Rac2, Cdc42, Rap1A and Rap1B, which have defined roles in adhesion signalling and cytoskeletal organization^21^,^22^, and the Arf and Rab GTPase family members Arf1, Arf4, Arf5, Arf6, Rab1A, Rab8A, Rab11A, Rab13, Rab14 and Rab35, which are involved in membrane trafficking^23^, were all enriched in inactive integrin complexes. Other terms related to adhesome functional classes, including chaperone and RNA regulation terms, were also overrepresented in the data sets, mostly enriched to active integrin complexes. Intriguingly, proteins associated with cell division were overrepresented in both the active integrin and unenriched protein clusters (Supplementary Table 2), suggesting a link between integrin activation state and the cell division machinery. These data indicate that proteins with different cellular roles are recruited to integrins in an activation-specific manner and that these complexes can be isolated and detected by MS. The precise role that integrin activation state plays in the function of these proteins will require further study.

**Figure 3:**
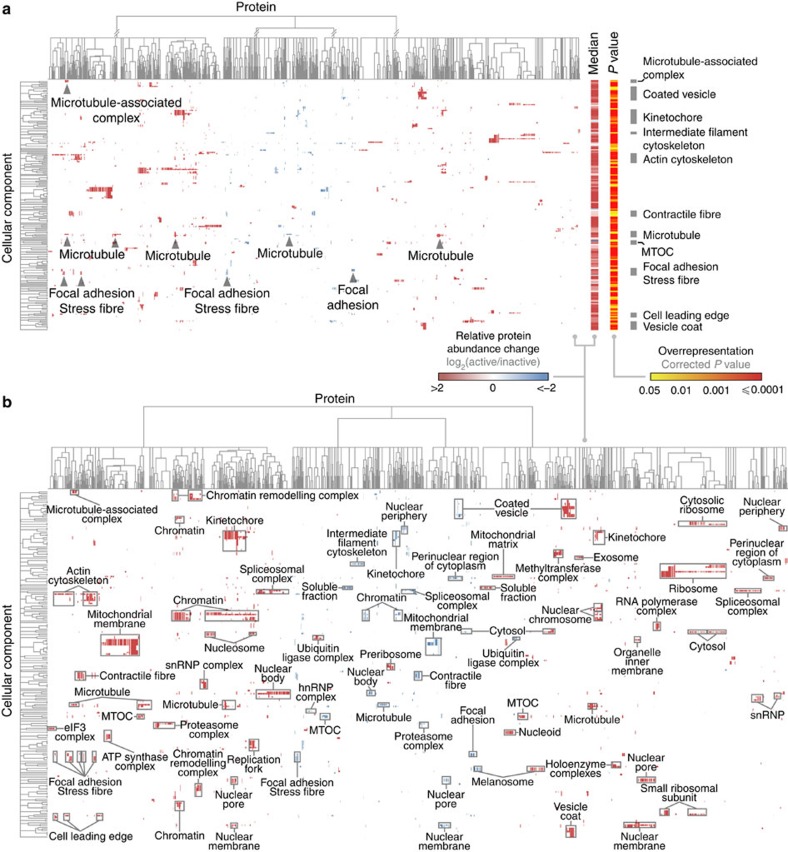
Functional enrichment map of the adhesion complex landscape. (**a**) Overrepresented cellular component terms from proteins identified by MS were hierarchically clustered according to protein enrichment in active (red) or inactive (blue) integrin complexes. This identified clusters of similarly enriched proteins associated with a similar set of functional terms. Arrowheads indicate clusters of proteins assigned focal adhesion and microtubule-associated terms. Accompanying heat bars (right) indicate median protein enrichment (log_2_ transformed) and false discovery rate-corrected *P* value (all <0.05; log_10_ scale) for each cellular component term. Grey bars (right) highlight focal adhesion and cytoskeleton terms. (**b**) Additional annotation of the cellular component terms on the functional enrichment map in **a** revealed the range and specificity of cellular localizations reported for proteins enriched in active and inactive integrin complexes. Clusters containing at least eight proteins were labelled in addition to the cell adhesion terms highlighted in **a**. MTOC, microtubule-organizing centre; snRNP, small nuclear ribonucleoprotein.

We next performed a global assessment of functional enrichment across all proteins identified by MS in adhesion complexes. Hierarchical clustering of the relative protein abundance changes was used to identify clusters of overrepresented cellular components in the context of protein recruitment to active or inactive integrin (Fig. 3a). Clusters of focal adhesion terms contained a balance of proteins enriched in active and inactive integrin complexes. This was supported by pathway and functional network analyses (Supplementary Fig. 6), which, as expected, identified significant enrichment of integrin signalling and cell adhesion components in the data sets. A number of the adhesion-related enzymes in these pathways (for example, Rac1, RhoA, Cdc42 and Arf6) were enriched in inactive integrin complexes (Supplementary Fig. 6), as noted above (Fig. 2b and Supplementary Table 2). A considerable proportion of the networks consisted of cytoskeletal proteins enriched in active integrin complexes, consistent with the observed enrichment of actin regulators and adaptors in the active integrin data set (Fig. 2b and Supplementary Table 2), and this was reflected in the functional enrichment map (Fig. 3a). Moreover, five of the six identified clusters of microtubule or microtubule-associated complex terms contained proteins enriched in active integrin complexes (Fig. 3a and Supplementary Fig. 6), suggesting that, as a functional class, microtubule-associated proteins are recruited to β1 integrin in an activation state-dependent manner.

Interrogation of the complete functional enrichment map revealed a number of clusters of unexpected terms, many of which, such as splicing and translational machinery, were enriched in active integrin complexes (Fig. 3b), which is consistent with previous analyses^20^ and emphasizes the complexity of the focal adhesion environment. Together, these data chart the functional landscape of adhesion complexes and reveal the integrin activation state-dependent recruitment of proteins known to have roles in a broad range of cellular compartments. This supports the hypothesis that integrin activation state dictates the molecular composition of the local intracellular environment of adhesion complexes.

### Microtubules target sites of integrin activation

Of the microtubule-associated proteins that were identified in active integrin complexes, +TIPs were well represented. These are a diverse group of proteins that specifically accumulate at microtubule plus ends (which grow away from the centrosome)^24^. A total of 15 (48%) of the known +TIPs were identified by MS, of which 73% were more than twofold enriched in active integrin complexes (Table 1). Microtubules are dynamic structures that are involved in co-ordinating actin polymerization, cargo transport and remodelling of the plasma membrane, and are thus intimately associated with key functions such as adhesion dynamics and cell migration^25^. In addition, microtubules have been reported to target and interact with focal adhesions^26^,^27^. Therefore, the observed enrichment of +TIPs in active integrin complexes suggested that receptor activation could be important for directing the growth of microtubules to the plasma membrane.

**Figure 4:**
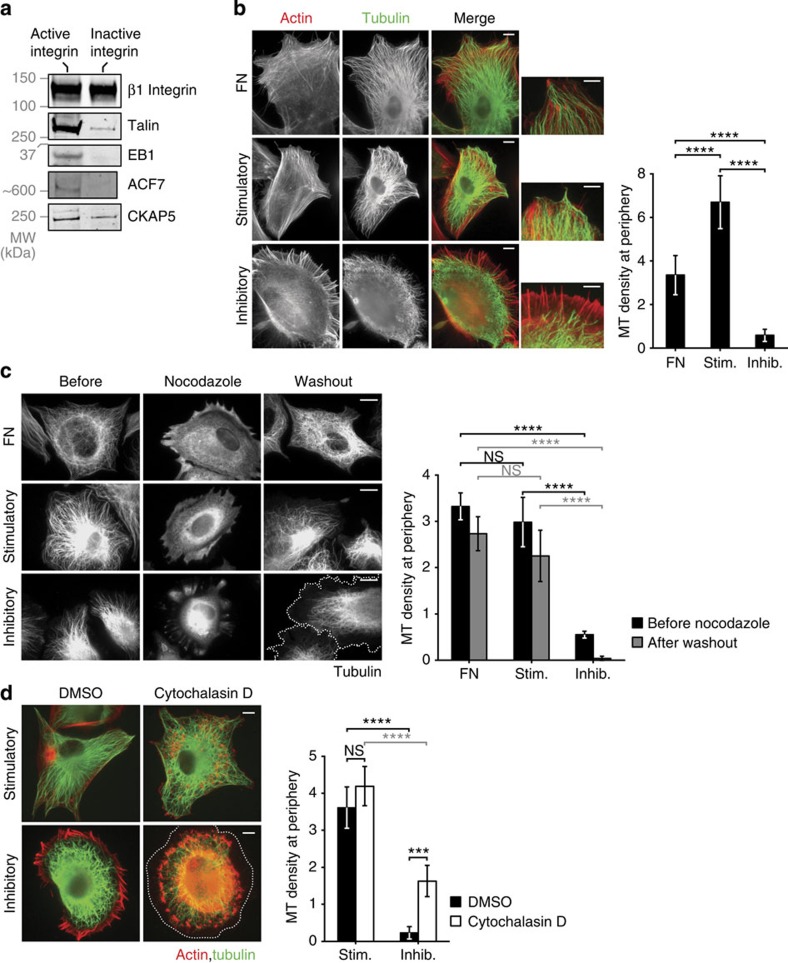
Microtubule (MT) morphology and dynamics are dictated by integrin activation state. (**a**) Enrichment of talin and three +TIPs, EB1, ACF7 and CKAP5, in complexes associated with active β1 integrin shown by western blotting (see Supplementary Fig. 10 for original blots). (**b**) HFFs spread on FN, stimulatory and inhibitory anti-β1 integrin mAbs stained for actin (red) and α-tubulin (green), with corresponding high-power images highlighting the difference in the location of MTs at the cell periphery in cells spread on the inhibitory mAb. MT density was calculated by counting the number of MTs within a 5 × 2 μm region of the cell periphery. Results are mean±s.d. (*n*=9, 10 and 8 cells for FN, stimulatory and inhibitory, respectively). (**c**) HFFs spread on FN, stimulatory and inhibitory mAbs for 1 h before treatment with 10 μM nocodazole for 45 min and subsequent washout for a further 45 min to examine MT regrowth. Cells were stained for tubulin; dotted line in bottom-right image indicates cell periphery. MT density was measured as in **b**. Results are mean±s.d. (*n*=3, 3 and 4 cells for FN, stimulatory and inhibitory, respectively). (**d**) HFFs spread on stimulatory and inhibitory mAbs for 1 h before addition of 20 μM cytochalasin D or dimethylsulphoxide (DMSO) vehicle control for a further 1 h. Cells were stained for actin (red) and α-tubulin (green); dotted line in bottom-right image indicates cell periphery. MT density was measured as in **b**. Results are mean±s.d. (*n*=5 and 5 DMSO-treated cells and 5 and 7 cytochalasin D-treated cells for stimulatory and inhibitory, respectively). Scale bars, 10 μm. ***P<0.001, ****P<0.0001; one-way analysis of variance with Tukey’s *post hoc* correction in **b**, two-way analysis of variance with Tukey’s *post hoc* correction in **c** and **d** (see Supplementary Table 4 for statistics source data). Inhib., inhibitory; MW, molecular weight; NS, nonsignificant; Stim., stimulatory.

To assess the recruitment of +TIPs to integrin complexes, affinity-isolated proteins were subjected to western blotting (WB; Fig. 4a). As expected, talin was strongly enriched in complexes associated with active β1 integrin, confirming the MS data. Moreover, the +TIPs EB1, ACF7 and CKAP5 were also enriched in active integrin complexes (Fig. 4a). To test the integrin activation state dependence of +TIP recruitment, HFFs were spread on integrin activation state-specific mAbs and stained for tubulin. HFFs spread on stimulatory mAb contained microtubules that extended to the cell periphery in a similar manner to those on FN (Fig. 4b). In contrast, cells spread on inhibitory mAb contained microtubules that did not reach the edge of the cell (Fig. 4b). The integrin activation state-dependent growth of microtubules to the cell periphery was also observed in other cell types and for the localization of the +TIP EB1 (Supplementary Fig. 7), and with a variety of stimulatory mAbs (Supplementary Fig. 8), substantiating the correlation between active integrin and microtubule targeting to the cell periphery. Furthermore, quantification of the expression of endogenous EB1 showed that integrin activation state did not alter the level of EB1 in areas away from the cell periphery, indicating that the observed effect was not due to differential expression of EB1 (Supplementary Fig. 7d).

To assess further the role that integrin activation state plays in microtubule organization, the regrowth of microtubules was evaluated after depolymerization with nocodazole. HFFs spread on stimulatory or inhibitory integrin mAbs underwent a rapid depolymerization of the microtubule network upon nocodazole treatment similar to cells on FN. For cells on both FN and stimulatory mAb, subsequent washout of nocodazole allowed regrowth of the microtubules to the cell periphery (Fig. 4c). In contrast, microtubules of cells on inhibitory mAb failed to reach the cell periphery and exhibited reduced regrowth after nocodazole washout (Fig. 4c). These results demonstrate that microtubule regrowth after depolymerization is affected by integrin receptor activation state and that active β1 integrin, or lack of inactive integrin, is required for microtubules to reach the cell periphery. Together, these observations confirm and extend the results obtained from MS analysis and indicate that microtubule growth is directed to active, or away from inactive, integrin at the plasma membrane.

Previous studies have shown that the growth of microtubules towards the cell periphery can be guided by interactions with actin filaments^28^,^29^. To test whether targeting of microtubules to active integrin was dependent on actin, HFFs were spread on the activation state-specific mAbs followed by depolymerization of the actin cytoskeletal network. Addition of cytochalasin D to cells plated on stimulatory mAb did not affect the ability of microtubules to reach the cell periphery (Fig. 4d). For cells spread on inhibitory mAb, depolymerization of actin resulted in a slight increase in the number of microtubules at the edge of the cell but not to the level measured for cells on stimulatory mAb (Fig. 4d). These findings indicate that actin filaments are not required to maintain microtubules at the cell periphery, suggesting an independent mechanism to link microtubules with active integrin. Also, the fact that microtubule targeting of the cell periphery in cells on inhibitory mAb was only partially rescued after actin depolymerization indicates that, although microtubule growth to the plasma membrane is impeded to some extent by the retrograde flow of actin at the ruffles observed in these cells, it does not account for the activation state-dependent effects observed.

### Active integrins stabilize microtubules at the cell cortex

**Figure 5:**
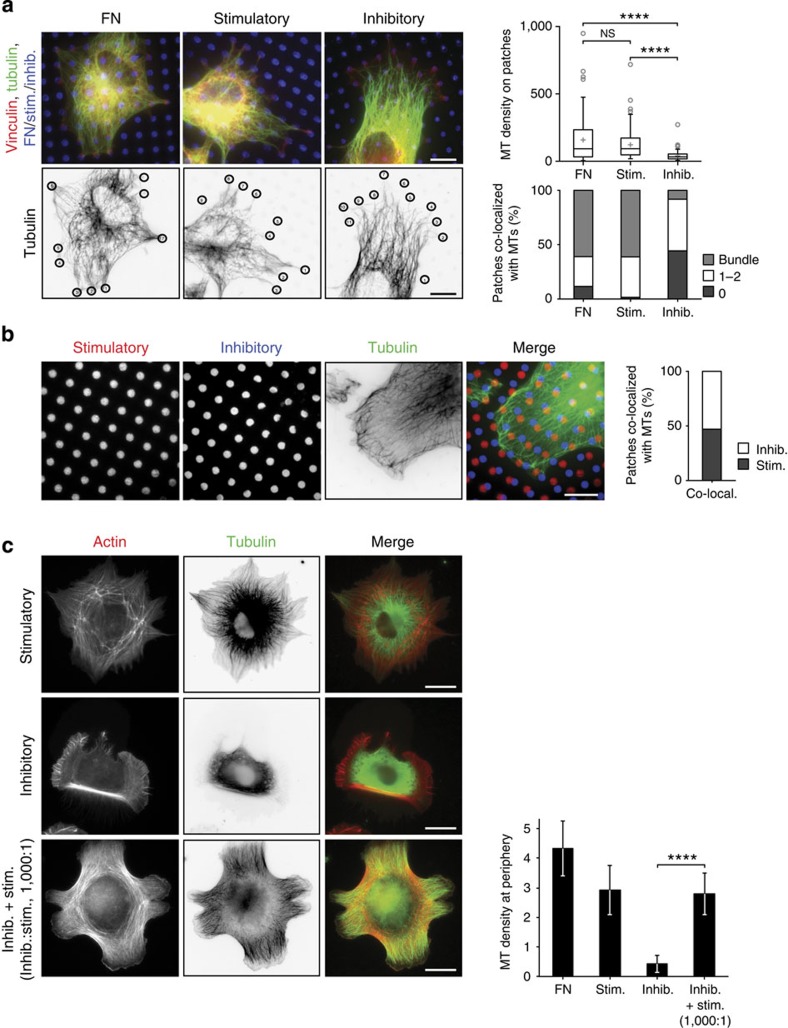
Active integrin stabilizes microtubules (MTs) at the cell cortex. (**a**) MT targeting was assessed using engineered micropatterns coated with FN, stimulatory and inhibitory mAbs. HFFs spread on micropatterns were stained for vinculin (red), α-tubulin (green) and ligand (blue) (upper image panel). MT density in areas of the cell periphery corresponding to ligand-coated patches (black circles in lower image panel) was quantified by measuring fluorescence intensity in the tubulin channel (upper graph). Results are mean±s.d. (*n*=95, 125 and 97 patches for FN, stimulatory and inhibitory, respectively). The same regions were also categorized as containing no MTs, 1–2 MTs or several MTs bundled together, which was expressed as a percentage of *n* patches (lower graph). (**b**) HFFs spread on micropatterns with alternating patches of stimulatory (red) and inhibitory (blue) mAbs were stained for α-tubulin (green). Patches of stimulatory or inhibitory mAb at the cell periphery targeted by MTs were expressed as a percentage of *n* patches (*n*=864 patches). (**c**) HFFs spread on stimulatory, inhibitory and a mixture of both mAbs (1,000:1, inhibitory:stimulatory molar ratio) were stained for actin (red) and α-tubulin (green). MT density was measured as in Fig. 4b. Results are mean±s.d. (*n*=9, 12, 11 and 10 cells for FN, stimulatory, inhibitory and inhibitory plus stimulatory, respectively). Scale bars, 10 μm. ****P<0.0001; Kruskal–Wallis test with Dunn’s *post hoc* correction in **a**, two-tailed unpaired *t* test with Welch’s correction in **c** (see Supplementary Table 4 for statistics source data). Co-local., co-localized; Inhib., inhibitory; NS, nonsignificant; Stim., stimulatory.

Cells spread on stimulatory, but not inhibitory, anti-integrin mAb form vinculin-rich focal adhesion-like structures (Fig. 1a), suggesting that integrins are also clustered at these sites. To test whether integrin clustering rather than activation state was responsible for microtubule growth to the cell periphery, engineered micropatterns of activation state-specific mAbs^30^ were used to force β1 integrins into discrete areas of the cell and cluster them on specific mAbs. Consistent with the observations on non-patterned surfaces, microtubules targeted patches of FN or stimulatory integrin mAb at the periphery of cells (Fig. 5a). In many cells spread on the stimulatory mAb, microtubules formed bundles to target the areas of active integrin. In contrast, microtubules of cells spread on inhibitory mAb micropatterns remained distributed evenly throughout the cell, with very few or no microtubules targeting the mAb-coated patches at the periphery of cells (Fig. 5a). Quantification of tubulin intensity over peripheral ligand patches and analysis of microtubule numbers recruited to the patches confirmed the recruitment of microtubules to zones of active integrin. Therefore, these data demonstrate that microtubule targeting to the cell periphery depends on integrin activation state and not clustering.

To investigate the targeting of microtubules to each integrin activation state within an individual cell, micropatterned surfaces were engineered with discrete, alternating arrays of stimulatory and inhibitory mAbs. Surprisingly, microtubules within an individual cell did not preferentially target stimulatory over inhibitory mAb patches (Fig. 5b). To explain this finding, we hypothesized that patches of active integrin may initiate active integrin-related signalling pathways within the cell that could override the effects from areas of inactive integrin. To test this possibility, HFFs were spread on surfaces coated with inhibitory integrin mAb in the presence of a range of concentrations of stimulatory integrin mAb. Remarkably, even a low concentration of stimulatory mAb (0.001 × molar equivalent) was able to rescue the effect of the inhibitory mAb ligand and promote growth of microtubules to the cell periphery (Fig. 5c). These data indicate that rather than exclusively targeting areas of active integrin, microtubules are recruited to the environment of active integrin via a permissive signal that is dominant over inhibitory signals.

**Figure 6:**
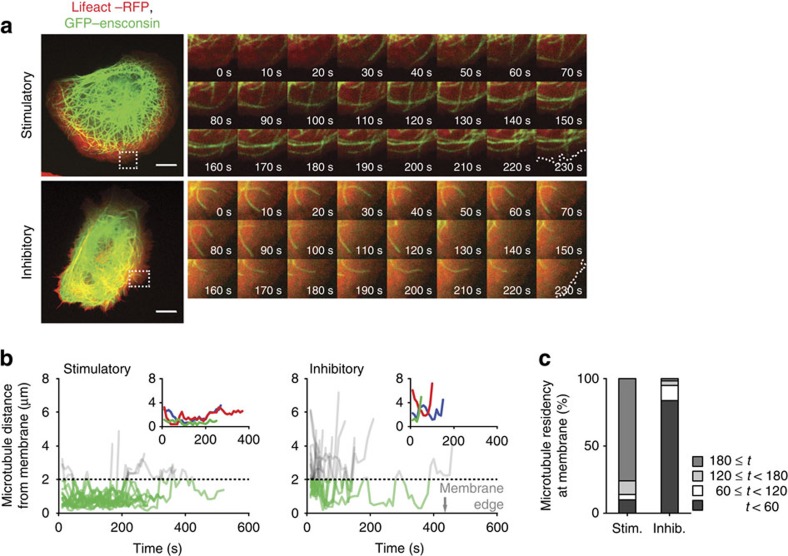
Active integrin creates an environment that stabilizes microtubules at the cell cortex. (**a**) U2OS cells expressing Lifeact–RFP (red) and GFP–ensconsin (green) to visualize actin and microtubules, respectively, were spread on stimulatory and inhibitory mAbs, and microtubule dynamics were tracked by live confocal microscopy. Scale bars, 10 μm. Sequences of images (right panels) correspond to areas highlighted in white dotted boxes (left panels) recorded over a period of 230 s (one image acquired every 10 s; see Supplementary Movies 1 and 2). White dotted line in final image of each sequence indicates the cell periphery as defined by the actin channel. (**b**) The distance between microtubule tips and the cell edge over the lifetime of each microtubule in the field of view (*n*=15 microtubules for both stimulatory and inhibitory). Microtubules within 2 μm of the cell periphery are shown in green. Inset graphs display traces from three individual microtubules. (**c**) The length of time (*t*, s) each quantified microtubule remained within 2 μm of the cell periphery in cells plated on stimulatory and inhibitory mAbs was expressed as a percentage of *n* microtubules (*n*=71 and 61 microtubules for stimulatory and inhibitory, respectively). Inhib., inhibitory; Stim., stimulatory.

Microtubules exhibit dynamic growth, catastrophe and regrowth, and as fixed images only provided a snapshot of the dynamic system, live cell imaging was performed to assess the effect of integrin activation state on microtubule dynamics. U2OS cells transfected with GFP–ensconsin^31^ and Lifeact–RFP^32^ to visualize microtubules and actin, respectively, were spread on activation state-specific mAbs and imaged by confocal microscopy (Fig. 6a and Supplementary Movies 1 and 2). These analyses revealed that, whereas microtubules reached the periphery of cells plated on both mAbs, microtubules of cells receiving activation signals from the stimulatory integrin mAb exhibited significantly longer lifetimes at the plasma membrane compared with those in cells on inhibitory mAb (Fig. 6b,c). In addition, in support of these findings, live imaging of cells expressing EB3–GFP showed that activation of integrin increased the displacement of this +TIP protein, representing increased microtubule growth (Supplementary Fig. 9 and Supplementary Movies 3, 4 and 5). These data demonstrate that activated integrin creates an environment or initiates signals that modulate +TIP dynamics, stabilize microtubules and result in the accumulation of microtubules at the cell periphery.

## Discussion

Here, we have isolated and characterized the composition of protein complexes associated with both active and inactive β1 integrins. We identified a third of reported adhesome components^4^ in β1 integrin complexes, of which 65 (84%) displayed activation state-specific profiles of recruitment. Essential activation-dependent, adhesion-related molecules, such as talin, kindlin and vinculin, were identified among the adhesome components specifically enriched in active integrin complexes, thereby validating the data set. A new finding deriving from our analysis of the MS data, and an exemplar of how this data resource might be exploited, was a link between integrin activation state and the dynamics of microtubules at the cell cortex. We have demonstrated that active integrins establish an environment that enables the stable penetration of microtubules to the cell periphery. These observations indicate that the proteomic data sets are representative of the different membrane and cytoplasmic protein environments surrounding active and inactive β1 integrins, and demonstrate the capacity of this data resource to identify proteins that play roles in activation state-dependent integrin signalling.

Stimulatory mAbs activate integrin function by stabilizing conformations that are competent to bind extracellular matrix ligands such as FN^13^. We have shown that mAb-induced integrin activation manifests itself not only by increased cell adhesion^33^ but also by an upregulation of associated signalling pathways and remodelling of the actin cytoskeleton. These findings are consistent with previous reports that either used engineered integrin mutants to restrict leg separation, and therefore constrain integrin activation, which resulted in reduced cell spreading^12^, or demonstrated enrichment of the cytoskeletal proteins IQGAP1 and filamin-A in sites of integrin activation but not integrin inactivation^30^. Indeed, both IQGAP1 and filamin-A were enriched in active integrin complexes in the present study (Supplementary Table 1). We identified many cytoskeleton-binding proteins enriched in the active integrin data set, which is likely to reflect more robust physical links from active integrin complexes to the cytoskeleton. The depletion of GTPases and GTPase-regulating proteins in the active integrin data set could suggest either that the regulation of membrane and cytoskeleton organization may be more prominent in the environment of inactive integrins (for example, RhoG-regulated integrin internalization^34^) or it is conceivable that inhibition of GTPase function results in accumulation of a higher level of inactive enzyme.

Although the composition of the environment surrounding active integrin has been well studied, there is less known about that surrounding inactive integrin or the concept of a non-canonical signalling environment for integrins. Inactive integrins are diffusely distributed throughout the cell membrane^12^. It has been suggested that the inactive state is regulated by protein binding, akin to the regulation of the active state. Proteins such as ICAP-1 (ref. 35), RPS6KA3 (RSK2 (ref. 36)), filamin^37^ and SHARPIN^38^ have been reported to induce integrin inactivation by binding to receptor cytoplasmic tails, and liprin-α1 has been reported to co-localize with inactive receptor^39^. Of these proteins, only RSK2 was detected by MS and enriched in inactive integrin complexes (Supplementary Table 1). The presence of the other proteins could be integrin heterodimer- or cell type-specific. In addition, as the complexes identified in this study were induced upon stabilization of integrin conformation from the outside of the cell, they may reflect an outside-in inactive complex. Recently, differences in outside-in and inside-out activation signals have been reported^40^ and, although speculative, similar modes of regulation may also occur to regulate integrin inactivation. Nonetheless, it will be interesting to investigate if the proteins enriched in inactive integrin complexes are actively recruited to inactive integrin or dispersed in the cytoplasm adjacent to the plasma membrane as a consequence of inactivation of signalling pathways.

Our MS experiments were performed using non-adherent cells that are less likely to be polarized than adherent cells, and which probably do not form mature focal adhesions. Nevertheless, we identified in the active integrin sample many of the +TIP proteins that have been shown to be necessary for cortical capture of microtubules (EB1 (ref. 41), ACF7 (ref. 28)) as well as putative microtubule-cell cortex anchoring-related proteins (IQGAP^42^, mDia^43^, CLASP1 and 2 (ref. 44), dynein^45^, LL5α and β^44^, moesin^46^, septins^47^), which strengthens the suggestion that active integrin is required for this process. In addition, activation of Rho GTPases is often required for microtubule capture by these molecules^48^, and we identified the Rho guanine nucleotide exchange factor ARHGEF2 (GEF-H1)^49^ in the active integrin data set. It should also be noted that previous studies have determined a role for ILK in controlling microtubule dynamics and polarity^50^,^51^, a protein that was not found enriched to active integrin in this study. To reveal further insights into the mechanism of microtubule targeting to areas of active integrin, we performed a series of short interfering RNA-mediated knockdown experiments that targeted either candidates derived from the literature, +TIPs or candidate proteins identified by analysis of the intersection of +TIPs and integrin protein–protein interaction networks generated from the proteomic data (Supplementary Table 3). Interestingly, a reduction in the expression of the chosen candidate proteins did not affect the ability of microtubules to target the cell periphery. These data likely reflect that multiple redundant pathways exist to control cortical targeting of microtubules. The experiments using engineered micropatterns demonstrated that microtubules target areas of active integrin, and that active integrin increases their residency at the cell periphery. Intriguingly, even very low concentrations of stimulatory mAb could overcome the effect of inhibitory mAb, suggesting that only a small proportion of active integrin is required to establish an environment that allows microtubule targeting and capture. This further suggests that there may be regions of active integrin in the membrane of migrating cells to which microtubules are specifically targeted, in a process that may involve lipid rafts^52^.

Our study charts the functional landscape of adhesion complexes and reveals the integrin activation state-dependent recruitment of proteins with a broad range of cellular functions. Many of these processes have been poorly studied in the context of adhesion^20^, yet there is evidence that proteins involved in, for example, protein synthesis^53^ are specifically recruited to integrin adhesion sites. Thus, the enrichment of these unexpected components in integrin complexes warrants additional study. The results generated by this work provide a valuable resource to inform further investigations of the mechanisms of integrin activation and inactivation.

## Methods

### Reagents

Antibodies to human β1 integrin used as ligands were 12G10 (ref. 33; Serotec, MCA 2028, 10 μg ml^−1^), which stabilizes the active integrin conformation, and 4B4 (Beckman Coulter, 6603113, 10 μg ml^−1^), which stabilizes the inactive integrin conformation. Both 12G10 and 4B4 are the same isotype (IgG1). Other antibodies used were directed against α-tubulin (YL1/2; Abcam, ab6061, 1:1,000 for IF), ACF7 (CU119; provided by R. K. Liem, 1:2,000 for WB), β1 integrin (JB1A; provided by J. A. Wilkins, 1:1,000 for WB), CKAP5 (E-17; Santa Cruz Biotechnology, sc240235, 1:200 for WB), EB1 (Santa Cruz Biotechnology; H-70, sc-15347, 5 μg ml^−1^ for IF or 1A11, sc-47704, 1:1,000 for WB), talin (C-20; Santa Cruz Biotechnology, sc-7534, 1:2,000 for WB), vinculin (hVIN-1–FITC conjugate; Sigma-Aldrich, F-7053, 1:500 for IF) and FN (39B6; 5 μg ml^−1^)^54^. Actin was visualized with phalloidin–Texas Red conjugate (Life Technologies, T7471, 1:500 for IF) or Lifeact–RFP (provided by R. Wedlich-Söldner). Tubulin was visualized with GFP–ensconsin (provided by S. Woolner). Species-specific fluorescent dye-conjugated secondary antibodies were obtained for IF from Jackson Immunoresearch (1:400), and FN, PDL, cytochalasin D and nocodazole were from Sigma-Aldrich.

### Cell culture

K562 cells were cultured in RPMI 1640 medium supplemented with 10% (v/v) fetal calf serum (Lonza Bioscience) and 2 mM L-glutamine. Telomerase-immortalized HFF (provided by K. Clark), HeLa and U2OS cells were cultured in DMEM supplemented with 10% (v/v) fetal calf serum and 2 mM L-glutamine. All cells were maintained at 37 °C in a humidified 5% (v/v) CO_2_ atmosphere. HeLa cells stably expressing EB3–GFP were a gift from A. Tighe and S. S. Taylor. All cell lines were obtained from either the American Type Culture Collection or the European Collection of Animal Cell Cultures unless otherwise stated.

### Integrin activation state adhesion complex isolation

Integrin adhesion complexes were isolated using an approach similar to the ligand affinity purification method described by Humphries *et al.*^7^, which was adapted to enable isolation of integrin activity-dependent complexes. Activation state-specific anti-β1 integrin mAbs (12G10, anti-active β1 integrin; 4B4, anti-inactive β1 integrin)^18^ (200 μg ml^−1^) were coupled to 4.5-μm-diameter tosyl-activated paramagnetic beads (M-450 Dynabeads; Life Technologies) as described in the manufacturer’s protocol. Coated beads were incubated with cells in HEPES-buffered saline (150 mM NaCl, 25 mM HEPES, pH 7.4) containing 4.5 mg ml^−1^ glucose and supplemented with 0.2% (w/v) BSA and 1 mM divalent cations at 70 r.p.m. for 30 min at 37 °C (Innova 4230, New Brunswick Scientific Co., Inc.). Cation conditions (1 mM MnCl_2_ for 12G10, or 1 mM CaCl_2_ and 1 mM MgCl_2_ for 4B4) were chosen to promote maximal exposure of the relevant activation state-dependent mAb epitopes. Bead-bound cells were crosslinked and lysed, and isolated protein complexes were washed and eluted as described previously^7^,^19^. In brief, integrin activation state-dependent adhesion complexes were allowed to form in K562 cells against paramagnetic beads coated with activation state-specific anti-β1 integrin mAbs. Protein complexes were then stabilized with DTBP, a thiol cleavable crosslinker, and crosslinks cleaved under reducing conditions during recovery of the complexes. Proteins were then separated by SDS–polyacrylamide gel electrophoresis (SDS–PAGE) and immunoblotted or prepared for analysis by MS (see below).

### Immunoblotting

Following SDS–PAGE, resolved proteins were transferred to nitrocellulose membrane (Whatman). Membranes were blocked and probed as described previously^7^. Secondary antibodies used were donkey polyclonal Alexa Fluor 680-conjugated anti-goat IgG or anti-mouse IgG (Life Technologies, 1:5,000) and donkey polyclonal IRDye 800-conjugated anti-mouse IgG (Rockland Immunochemicals, 1:5,000). Membranes were scanned using the Odyssey infrared imaging system (LI-COR Biosciences). Images were cropped and linearly adjusted for levels using Photoshop (version CS6; Adobe) and assembled using Illustrator (version CS6; Adobe; see Supplementary Fig. 10 for uncropped images).

### MS data acquisition

Following SDS–PAGE, entire gel lanes were sliced into 30 pieces per lane and subjected to in-gel digestion with trypsin as described^7^. Peptide samples were analysed by liquid chromatography-tandem MS using a nanoACQUITY UltraPerformance liquid chromatography system (Waters) coupled online to an LTQ Velos (Thermo Fisher Scientific). Peptides were concentrated and desalted on a Symmetry C_18_ preparative column (20 mm × 180 μm inner diameter, 5-μm particle size; Waters) and separated on a bridged ethyl hybrid C_18_ analytical column (250 mm × 75 μm inner diameter, 1.7-μm particle size; Waters) using a 45-min linear gradient from 1 to 25% (v/v) acetonitrile in 0.1% (v/v) formic acid at a flow rate of 200 nl min^−1^. Peptides were selected for fragmentation automatically by data-dependent analysis.

### MS data analysis

Tandem mass spectra were extracted using extract_msn (Thermo Fisher Scientific) executed in Mascot Daemon (version 2.2.2; Matrix Science). Peak list files were searched against the IPI Human database (version 3.70, release date 4 March 2010) modified to contain ten additional contaminants and reagent sequences of non-human origin. Searches were submitted to an in-house Mascot server (version 2.2.03; Matrix Science)^55^ as described previously^7^. Mass tolerances for precursor and fragment ions were 0.4 and 0.5 Da, respectively. Data were validated in Scaffold (version 3.00.06; Proteome Software) using a threshold of identification of at least 90% probability at the peptide level, assignment of at least two unique, validated peptides, and at least 99% probability at the protein level (estimated protein false discovery rate 0.1% for all data sets).

### MS data deposition

MS data were deposited in ProteomeXchange ( http://proteomecentral.proteomexchange.org) via the PRIDE partner repository^56^ with the data set identifier PXD000155 ( DOI: 10.6019/PXD000155). Details of all identified proteins are provided in Supplementary Data 1.

### MS data quantification

Relative protein abundance was calculated using the unweighted spectral count of a given protein normalized to the total number of spectra observed in the entire sample and to the molecular weight of that protein (normalized spectral count). MS data for each adhesion complex isolation were acquired in technical duplicate, for which normalized spectral counts were summed, and final results were reported as mean normalized spectral counts of biological duplicate isolations. Only proteins with a spectral count of at least four were used for further analysis. Statistical analysis of differential relative protein abundance was performed using QSpec^57^ (estimated false discovery rate <5%).

### Hierarchical clustering analysis

Unsupervised hierarchical clustering was performed on the basis of uncentred Pearson correlation using Cluster 3.0 (C Clustering Library, version 1.37)^58^. Distances between hits were computed using a complete-linkage matrix. Clustering results were visualized using Java TreeView (version 1.1.1)^59^ and MultiExperiment Viewer (version 4.1.01)^60^.

### Interaction network analysis

Interaction network analysis was performed using Cytoscape (version 3.0.2)^61^. Protein hits were mapped onto a merged human interactome consisting of protein–protein interactions reported in the Protein Interaction Network Analysis platform *Homo sapiens*, *Mus musculus* and *Rattus norvegicus* networks (release date 10 December 2012)^62^, the extracellular matrix interactions database MatrixDB (release date 20 April 2012)^63^ and the literature-curated adhesome^3^,^4^. Multiple subunits or paralogues of proteins reported in the adhesome database were considered as separate adhesome components. Topological parameters were computed from undirected graphs, excluding self-interactions, using NetworkAnalyzer^64^.

### Functional enrichment analyses

Groups of proteins identified by hierarchical clustering analysis were analysed using the Database for Annotation, Visualization and Integrated Discovery (version 6.7)^65^. For clarity, only top-level SwissProt Protein Information Resource keywords were considered. Keywords with fold enrichment ≥1.5, Bonferroni-corrected *P* value <0.05, EASE score (modified Fisher’s exact test) <0.05 and at least two proteins per keywords were considered significantly overrepresented.

For generation of the functional enrichment map, overrepresentation of Gene Ontology terms was calculated using High-Throughput GoMiner^66^. One hundred randomizations were performed and data were thresholded for a 5% false discovery rate. Overrepresented terms with ≥5 and ≤500 assigned proteins were reported. Relative protein abundance ratios (binary-logarithm-transformed fold changes) were mapped onto proteins assigned to each overrepresented term, and the data matrix was subjected to hierarchical clustering analysis as described above.

For pathway analysis, mean normalized spectral count data for each identified protein were normalized to the lowest-value sample and filtered for presence across all experimental conditions. Pathway analysis was performed using IPA (Ingenuity Systems), including direct and indirect relationships, and overrepresented canonical pathways (*P*<0.05, Fisher’s exact test) were manually interrogated.

Functional interaction networks were generated using EGAN (version 1.4)^67^. Overrepresented association nodes (functional terms; *P*<0.05, Fisher’s exact test) were manually interrogated and used to seed association networks based on protein–protein interactions and literature co-occurrence.

### Preparation of micropatterned substrates

Poly(dimethyl siloxane) (PDMS; Sylgard 184; Dow Corning) stamps with patterns were cast from photoresist-patterned silicon wafers as described previously^30^,^68^. Patterns (2 μm in diameter) were arranged in square arrays with 9-μm distance between the centres of each patch. To constrain β1 integrin conformation, PDMS was inked with either activation state-specific anti-β1 integrin mAbs (12G10, active; 4B4, inactive) or plasma FN at 50 μg ml^−1^ in PBS for 1 h at room temperature. The stamps were then submerged in sterile water and dried using a stream of N_2_. The stamps were placed feature-face down on PDMS-coated glass coverslips that were treated with ultraviolet ozone (Jelight Company) to transfer the patterned protein to the substrates. Double stamp patterns were created by stamping twice in a random order. Finally, Pluronic F-127 (Sigma-Aldrich) was adsorbed onto the coverslips to prevent nonspecific protein adsorption to the PDMS substrates.

### IF microscopy

Cells were spread on either FN, PDL or anti-β1 integrin mAbs coated on glass-bottom dishes at 10 μg ml^−1^ (MatTek) or on micropatterned coverslips (as described above) in serum-free DMEM containing 25 mM HEPES at 37 °C, 8% (v/v) CO_2_ for 1 h. If required, cytochalasin D at a final concentration of 20 μM, nocodazole at 10 μM or equivalent volume of dimethylsulphoxide vehicle was added and incubation continued as needed by the experiment. The cells were fixed in either −20 °C methanol or, to enable visualization of both actin and microtubules, 4% (w/v) paraformaldehyde/0.5% (v/v) glutaraldehyde (both from Sigma-Aldrich) and permeabilized with 0.1% (w/v) Triton X-100 (Sigma-Aldrich) if required. Cells were incubated with primary antibodies for 45 min, washed with PBS and then incubated with the appropriate fluorescent secondary antibodies for 30 min. Anti-mouse IgG fluorescent conjugates were used to stain the mAb-coated micropatterns, whereas FN patterns were visualized with anti-FN (39B6) followed by anti-mouse secondary. After further washing in PBS, cells were imaged at room temperature using a DeltaVision system (Applied Precision) comprising a wide-field inverted microscope (model IX-70; Olympus) with × 60/1.42 Plan Apo N or × 100/1.35 UPLAN APO objectives. Images were captured using a CCD camera (model CH350; Photometrics) and Softworx analysis software (Applied Precision).

### Image analysis

Images were compiled and analysed using ImageJ (version 1.42q; National Institutes of Health). To quantify microtubule density at the edge of cells on flat surfaces, the number of individual microtubule within each of a series of 5 × 2 μm boxes drawn around the entire cell periphery was counted. To quantify microtubule density over ligand-coated micropatterns, images of the microtubule cytoskeleton were background filtered with a rolling ball radius of 50 pixels. Using the IgG image as a template, areas corresponding to the patches were applied to the microtubule image at the cell periphery, which was visualized with vinculin staining. Density of microtubules over the patches was measured using the ImageJ built-in measure tool. Statistical analysis was performed using Prism (version 6.0d; GraphPad; see Supplementary Table 4 for statistics source data). To quantify endogenous EB1 expression, cells were stained with an anti-EB1 antibody (H-70; Santa Cruz Biotechnology) and regions away from the cell periphery selected at random. The amount of EB1-positive signal in areas at least 40 μm^2^ was determined as a percentage of the total measured area. Images were assembled using Photoshop and Illustrator.

### Plasmid transfection and live microscopy

HeLa cells were transfected with the EB1–GFP plasmid (provided by C. Ballestrem), using Lipofectamine Plus reagent (Invitrogen) according to the manufacturer’s instructions. To visualize microtubules and actin dynamics, U2OS cells were transiently transfected with a triple GFP-tagged version of the ensconsin microtubule-binding domain^31^ and Lifeact–RFP, using Lipofectamine 2000 reagent (Invitrogen) according to the manufacturer’s instructions. Following 4 h incubation, the transfection mixture was removed, fresh medium was added and transfected cells were used the following day. For optimal image resolution, the normal culture medium was replaced by Ham’s F-12 medium (Gibco) containing 25 mM HEPES without serum. Cells were plated on mAb-coated glass-bottom dishes for 30 min, and images were collected every 10 s at 37 °C on a spinning disk confocal microscope (Marianas; Intelligent Imaging Innovations) with a × 100 objective. Microtubule dynamics were analysed by measuring the distance between the cell edge (identified using the actin staining) and the growing tip of each microtubule, for each frame, using ImageJ. The whole of the cell periphery was used for quantification; microtubules targeting the membrane were tracked and their lifetimes at the membrane were measured. Microtubules were chosen for quantification only if their ends were visible and identifiable between frames. Videos were assembled using ImageJ.

To analyse the dynamics of microtubule plus ends, HeLa cells stably transfected with EB3–GFP were plated on FN- or mAb-coated glass-bottom dishes for 60 min, and images were collected every 10 s at 37 °C using total internal reflection fluorescence (TIRF) microscopy. Images were collected on a TE2000 microscope (Nikon), equipped with a perfect focus system to eliminate focus drift, using the × 100/1.49 Apo TIRF objective. The 488-nm laser line was manually adjusted until TIRF was achieved and the images were then collected through the Elements software (Nikon) using a Cascade 512B EM CCD camera (Photometrics). Acquired images were analysed in MATLAB (MathWorks) using plusTipTracker software^69^ to detect, track and visualize +TIP comets automatically. Maximum permitted gap length was 12 frames, maximum angle for forward growth was 30°, maximum angle for backward growth was 10°, minimum sub-track length was three frames, maximum shrinkage factor was 1.5 (relative to growth speed), search radius was 5–10 pixels and fluctuation radius was 1 pixel. Growth excursions of +TIP comet tracks, excluding shrinkage and pause events, were used to assess microtubule growth dynamics. Videos of +TIP comets detected and tracked using plusTipTracker were assembled using ImageJ.

## Author contributions

J.D.H. and M.J.H. conceived the project; A.B., J.A.A., J.D.H. and M.J.H. designed the experiments; A.B., J.A.A., J.D.H., G.J., E.J.K., S.W., C.K.C. and M.J.S. performed the experiments; A.B., J.A.A., J.D.H. and G.J. analysed the data; A.B., J.A.A., J.D.H., G.J. and M.J.H. interpreted the results; C.K.C., C.S.C. and D.K. contributed reagents/materials/analysis tools; A.B., J.A.A., J.D.H. and M.J.H. wrote the paper; all authors read and approved the manuscript.

## Additional information

**How to cite this article:** Byron, A. *et al.* A proteomic approach reveals integrin activation state-dependent control of microtubule cortical targeting. *Nat. Commun.* 6:6135 doi: 10.1038/ncomms7135 (2015).

**Accession codes**: MS data were deposited in ProteomeXchange ( http://proteomecentral.proteomexchange.org) via the PRIDE partner repository ( http://www.ebi.ac.uk/pride) with the data set identifier PXD000155 (DOI: 10.6019/PXD000155).

## Supplementary Material

Supplementary Figures and Tables Supplementary Figures 1-10, Supplementary Tables 1-4 and Supplementary References

Supplementary Data 1 Proteins identified in active or inactive integrin adhesion complexes by mass spectrometric analysis.

Supplementary Movie 1 Time-lapse imaging of a U2OS cell plated on stimulatory anti–β1 integrin mAb. Cells were transfected with GFP–ensconsin microtubule-binding domain and Lifeact–RFP. GFP channel only is shown with inverted contrast. Time interval between acquired frames was 10 s.

Supplementary Movie 2 Time-lapse imaging of a U2OS cell plated on inhibitory anti–β1 integrin mAb. Cells were transfected with GFP–ensconsin microtubule-binding domain and Lifeact–RFP. GFP channel only is shown with inverted contrast. Time interval between acquired frames was 10 s.

Supplementary Movie 3 Time-lapse imaging of a HeLa cell plated on FN. Cells were stably transfected with EB3–GFP. The movie shows +TIP comets automatically tracked for one cell per movie. Time interval between acquired frames was 10 s.

Supplementary Movie 4 Time-lapse imaging of a HeLa cell plated on stimulatory anti–β1 integrin mAb. Cells were stably transfected with EB3–GFP. The movie shows +TIP comets automatically tracked for one cell per movie. Time interval between acquired frames was 10 s.

Supplementary Movie 5 Time-lapse imaging of a HeLa cell plated on inhibitory anti–β1 integrin mAb. Cells were stably transfected with EB3–GFP. The movie shows +TIP comets automatically tracked for one cell per movie. Time interval between acquired frames was 10 s.

## Figures and Tables

**Table 1:** Activation state-dependent recruitment of microtubule plus-end tracking proteins (+TIPs) to integrin complexes.

**Gene name**	**Protein name**	**Alias(es)**	**Fold enrichment (active/inactive)**
*ARHGEF2*	Rho/rac guanine nucleotide exchange factor 2	GEF-H1	4.3
*BCAS2*	Pre-mRNA-splicing factor SPF27	DAM1	1.9
*CDK5RAP2*	CDK5 regulatory subunit-associated protein 2	CEP215	U
*CKAP5*	Cytoskeleton-associated protein 5	Ch-TOG, XMAP215	3.4
*CLASP1*	Cytoplasmic linker-associated protein 1	CLIP-associating protein 1, hOrbit1	5.1
*CLASP2*	Cytoplasmic linker-associated protein 2	CLIP-associating protein 2, hOrbit2	U
*DCTN1*	Dynactin subunit 1	p150-glued	1.6
*DIAPH1*	Protein diaphanous homologue 1	DRF1, mDia	2.5
*DST*	Dystonin	BPA	U
*DYNC1H1*	Cytoplasmic dynein 1 heavy chain 1	—	3.1
*KIF18B*	Kinesin-like protein KIF18B	—	U
*KIF2C*	Kinesin-like protein KIF2C	MCAK	1.4
*MACF1*	Microtubule-actin crosslinking factor 1	ABP620, ACF7	8.0
*MAPRE1*	Microtubule-associated protein RP/EB family member 1	EB1	1.7
*MAPRE2*	Microtubule-associated protein RP/EB family member 2	EB2	3.6

U, unique to active integrin adhesion complexes.

All +TIPs identified in adhesion complexes by MS are displayed. Proteins were classified as +TIP family members according to Gene Ontology assignment (GO:0051010, microtubule plus end binding; GO:0035371, microtubule plus end) or as described^24^,^70^. Non-identified +TIPs: *APC, CLIP1, KIF17, KIF2B, KNSTRN, MAPRE3, MLPH, MTUS2, MYO5A, NAV1, PAFAH1B1, PSRC1, SLAIN2, SPAG5, SRCIN1, STIM1*.
