# Supplementary material for: A proteomic approach reveals integrin activation state-dependent control of microtubule cortical targeting
Source: Nat Commun. 2015 Jan 22;6:6135. doi: 10.1038/ncomms7135 (PMC4317495; doi:10.1038/ncomms7135)
Supplement: Supplementary Figures and Tables — Supplementary Figures 1-10, Supplementary Tables 1-4 and Supplementary References [file ncomms7135-s1.pdf]

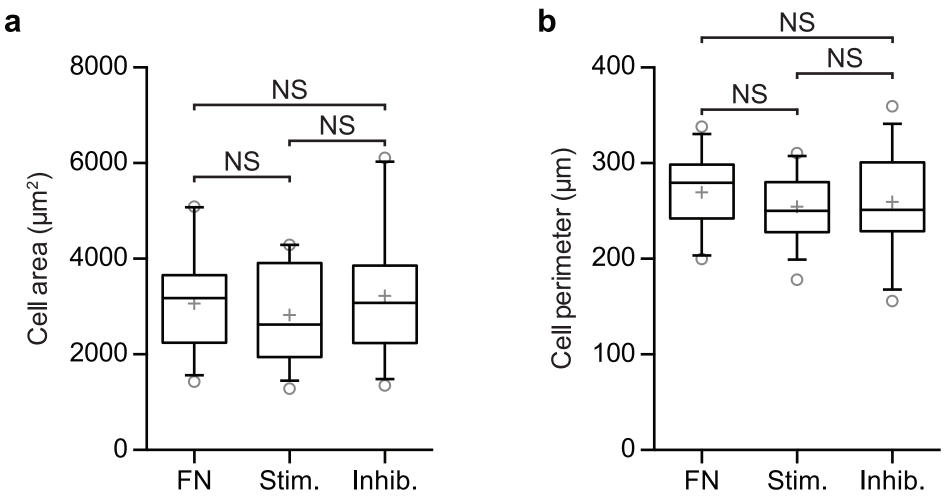

Supplementary Figure 1 | Morphology of cells spread on activity-specific anti-integrin mAbs. (**a**,**b**) HFFs were spread on FN or stimulatory or inhibitory anti-β1 integrin mAbs for 1 h. Cell areas (**a**) and perimeters (**b**) were measured using ImageJ. Box-and-whisker plots shows the median (line), mean (plus sign), 25th and 75th percentiles (box) and 5th and 95th percentiles (whiskers) (n = 31, 31 and 33 cells for FN, stimulatory and inhibitory, respectively). Comparisons between the three independent groups were determined by Kruskal–Wallis test with Dunn's multiple comparisons test.

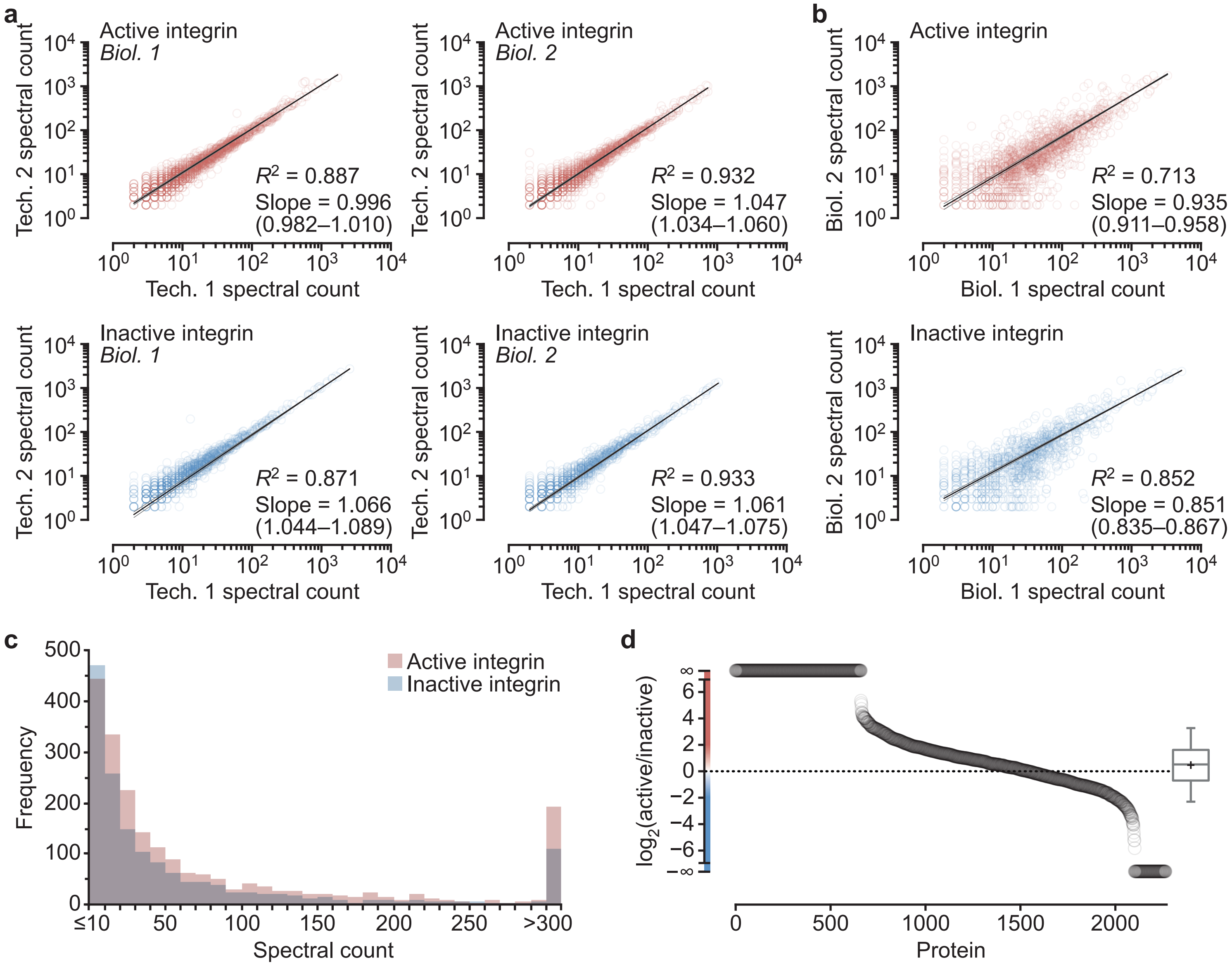

Supplementary Figure 2 | Quantification and reproducibility of MS data. (**a**,**b**) Spectral counts of proteins identified in active (red circles) and inactive (blue circles) integrin complexes were compared between technical replicate (**a**) and biological replicate (**b**) analyses. Proteins detected in both compared samples are displayed. Log–log lines (black lines) and associated 95% confidence bands (thin grey lines) were calculated by nonlinear regression analysis (least squares fit). Goodness of fit (R^2) and slope best fit values (95% confidence interval in parentheses) are displayed. Hougaard's measure of skewness was <0.10 (almost linear) for all slope best fits. (**c**) Frequency distribution of spectral counts of proteins identified in active (red) and inactive (blue) integrin data sets. Bins, 10 spectral counts. (**d**) Abundance ratios of proteins identified in active relative to inactive integrin complexes were determined from normalised spectral counts and $\log_2$ transformed. Colour bar indicates higher relative abundance in the active (red) or inactive (blue) integrin data sets. Non-infinite abundance ratios were normally distributed (non-Gaussian distribution $P > 0.05$; D'Agostino & Pearson omnibus normality test), with median abundance ratio 0.52 (0.40–0.60, 95% confidence interval). Box-and-whisker plot shows the median (line), mean (plus sign), 25th and 75th percentiles (box) and 5th and 95th percentiles (whiskers).

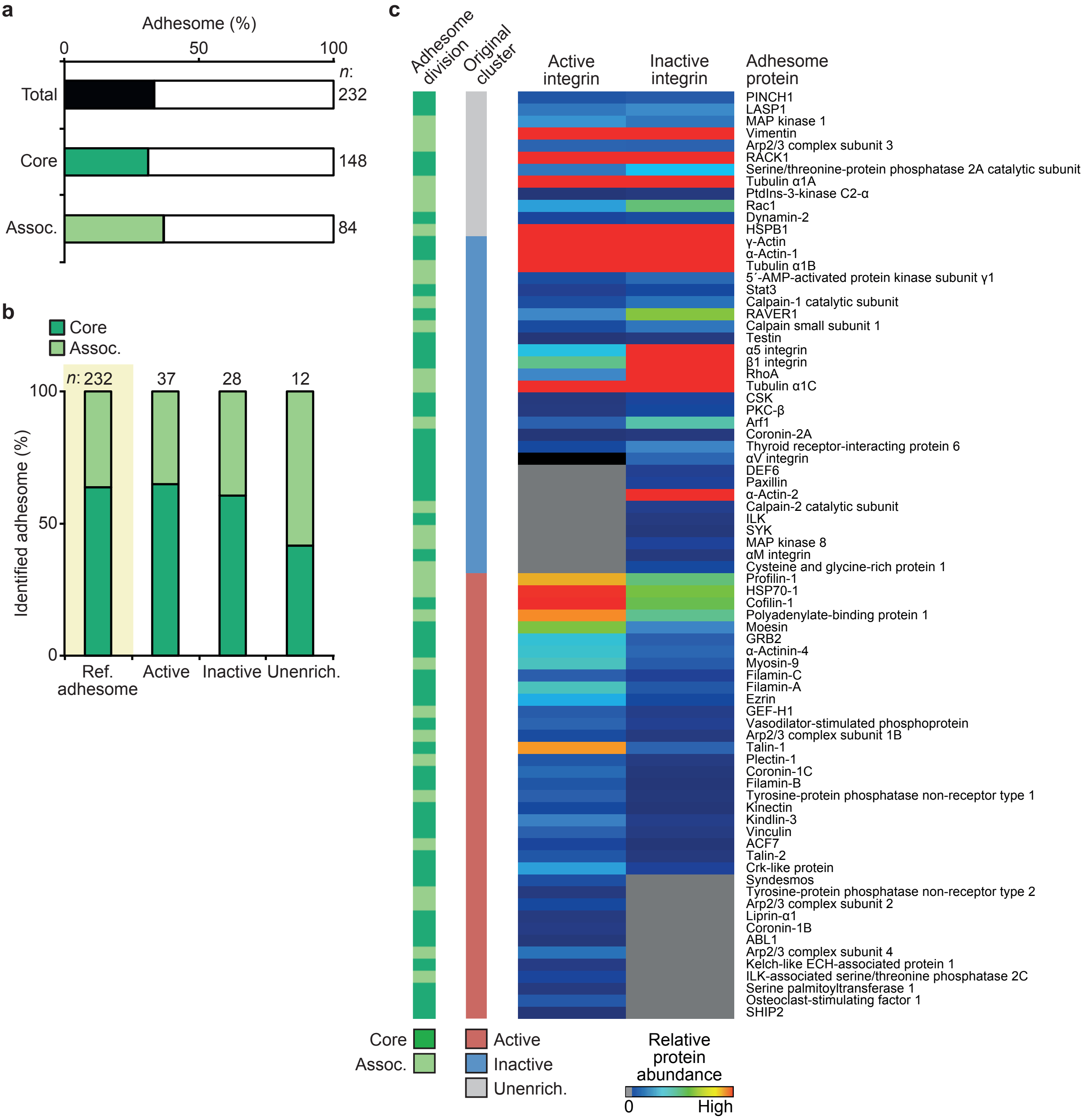

Supplementary Figure 3 | Adhesome components identified in activation state–dependent integrin complexes. (**a**) Proportions of the literature-curated adhesome[1] identified by MS. Of the total reported adhesome (n = 232 proteins), 77 proteins (33%) were identified by MS. Adhesome divisions of reported intrinsic (core) components (46 proteins; 31%) and transiently or peripherally associated components (31 proteins; 37%) were represented to a similar extent in the MS data. (**b**) Proportions of identified adhesome proteins reported as core or associated adhesome components. Of the identified adhesome proteins enriched in active integrin complexes (n = 37 proteins), 24 proteins (65%) were core adhesome components; of those enriched in inactive integrin complexes (n = 28 proteins), a similar proportion (17 proteins; 61%) were core adhesome components. These proportions are similar to the 64% of the total adhesome reported as core components (yellow box, reference adhesome). In contrast, identified adhesome proteins with similar abundance in both data sets (unenriched) were fewer (n = 12 proteins) and had a smaller proportion of core adhesome components (5 proteins; 42%). This may reflect a relatively transient or peripherally associated set of proteins recruited to integrin complexes in an activation state–independent manner. (**c**) Heat map shows relative protein abundance (normalised spectral count) for identified adhesome components (grey, not detected). Clusters of differentially enriched proteins are indicated, and protein order is preserved from the original hierarchical clustering analysis (see Fig. 1d and Supplementary Table 1 for full, hierarchically clustered MS data). Heat bar (left) indicates the distribution of core and associated adhesome components.

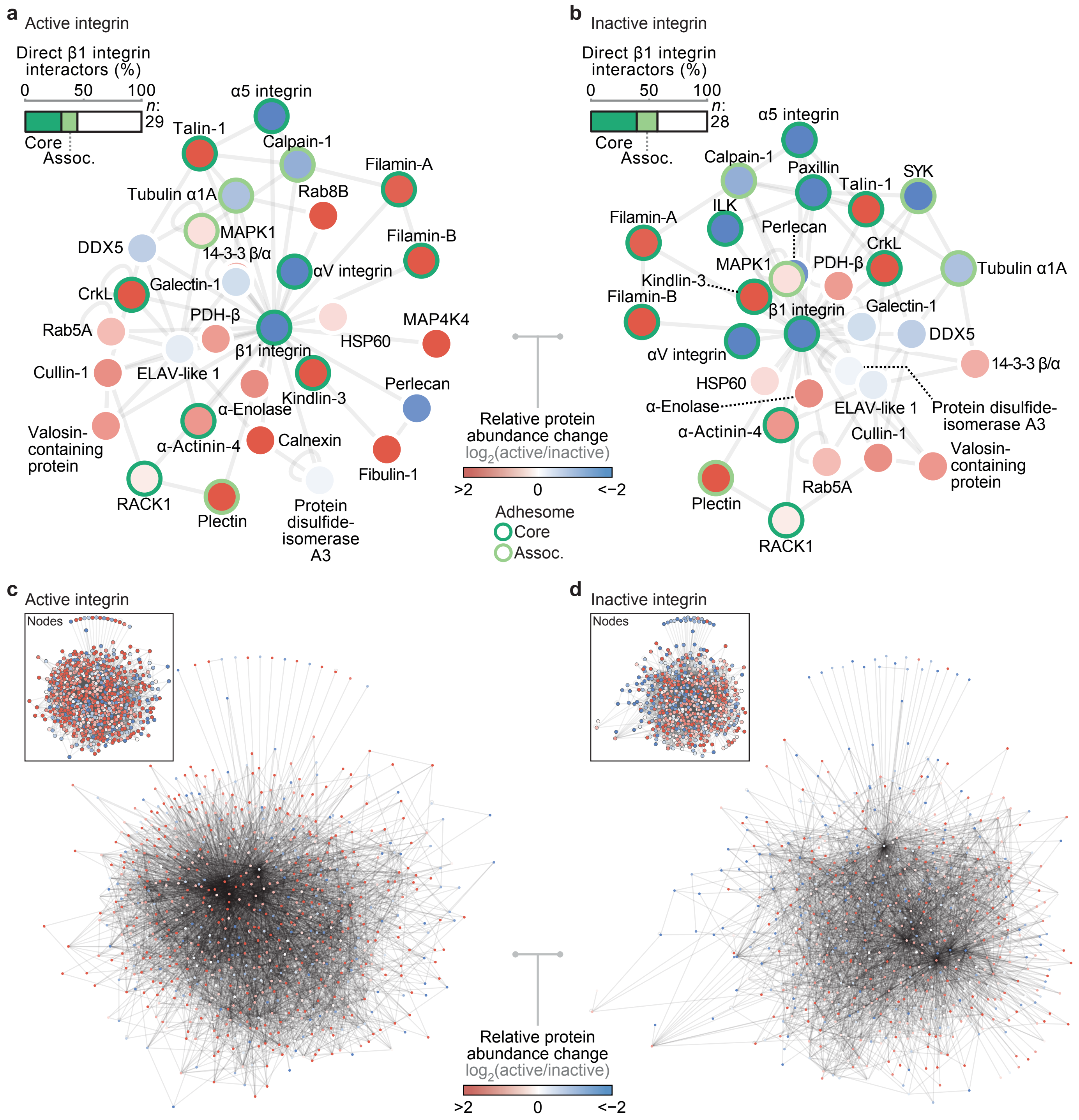

Supplementary Figure 4 | Interaction network analysis of activation state–dependent integrin complexes. (**a**–**d**) Quantitative MS data for all proteins identified in the integrin data sets were mapped onto a merged, multi-source interactome database to generate active and inactive integrin interactomes (see methods for details). Of the identified proteins reported to interact directly with β1 integrin (the one-hop interactions networks) (*n* = 29 and 28 for active and inactive integrin, respectively), 13 proteins (45%) in the active integrin network (**a**) and 16 proteins (57%) in the inactive integrin network (**b**) were reported adhesome components. Of these adhesome components, 69% were intrinsic (core) adhesome components in the active (9 proteins) and inactive (11 proteins) integrin networks. Analysis of the β1 integrin two-hop interaction networks (proteins reported to interact directly with β1 integrin or with β1 integrin–binding partners) identified 751 proteins in the active integrin network (**c**, inset) and 636 proteins in the inactive integrin network (**d**, inset), which represents 9% and 7% of the total reported β1 integrin two-hop interactome, respectively. These networks were highly interconnected, with 5152 and 3556 protein interactions in the active (**c**) and inactive (**d**) integrin networks, respectively. Nodes (circles) represent proteins identified by MS and are coloured according to protein enrichment in active (red) or inactive (blue) integrin complexes ($\log_2$ transformed). Edges (grey lines) represent reported protein-protein interactions. Networks were clustered using the yFiles Organic algorithm implemented in Cytoscape.

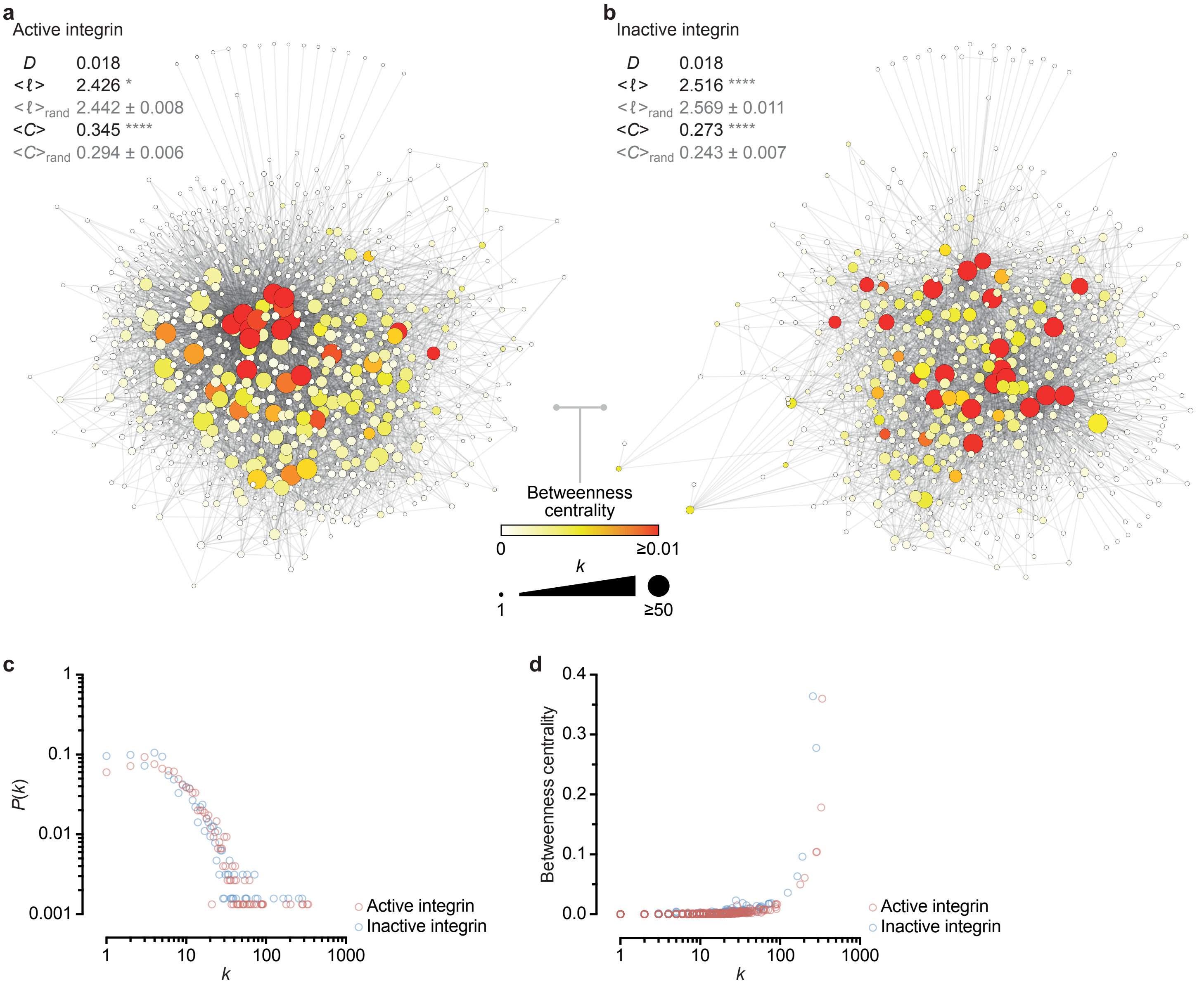

Supplementary Figure 5 | Topological analysis of integrin adhesion complex networks. (**a**,**b**) β1 integrin two-hop interaction networks constructed from the active (**a**) and inactive (**b**) integrin data sets were clustered using the yFiles Organic algorithm implemented in Cytoscape. Self-interactions were removed and topological parameters were computed using NetworkAnalyzer: *D*, network density; $\langle \ell \rangle$, average shortest path length; $\langle C \rangle$, average clustering coefficient. Shortest path length represents the ability of two nodes (proteins; circles) in a network to interact via the smallest number of edges (protein-protein interactions; grey lines). For both integrin networks, this value was smaller than those expected by random network simulations ($\langle \ell \rangle_{rand}$, grey font), indicating highly interconnected (navigable) networks. Nodes are sized according to degree (number of interactions; *k*) and coloured according to betweenness centrality. Betweenness centrality represents the potential of a node to be part of the shortest path length between different pairs of nodes in the network and is a measure of the load on or the flow of network traffic through the node. High betweenness centrality equates to a network bottleneck (high-traffic node). The clustering coefficient represents the capacity for groups of nodes to interact. A high clustering coefficient equates to highly interconnected clusters in the network. Clustering coefficients of both networks were larger than those of random simulations ($\langle C \rangle_{rand}$, grey font), indicating that the proteins identified by MS have a tendency to form groups within the networks, in line with the proposed modular nature of the adhesome[2,3]. Random networks were generated from 10^5 degree-preserving edge shuffles of each network. Results are mean ± s.d. ($n = 10^4$ rounds of randomisation). $^*P < 0.05$, $^{****}P < 0.0001$; two-tailed one-sample *Z* test. (**c**,**d**) Plots show the relationship between degree and the degree distribution, $P(k)$ (**c**), or betweenness centrality (**d**) for the active and inactive integrin networks. Highly connected proteins correlated with increased betweenness centrality, suggesting that hubs may influence the adhesion networks through both direct and indirect interactions.

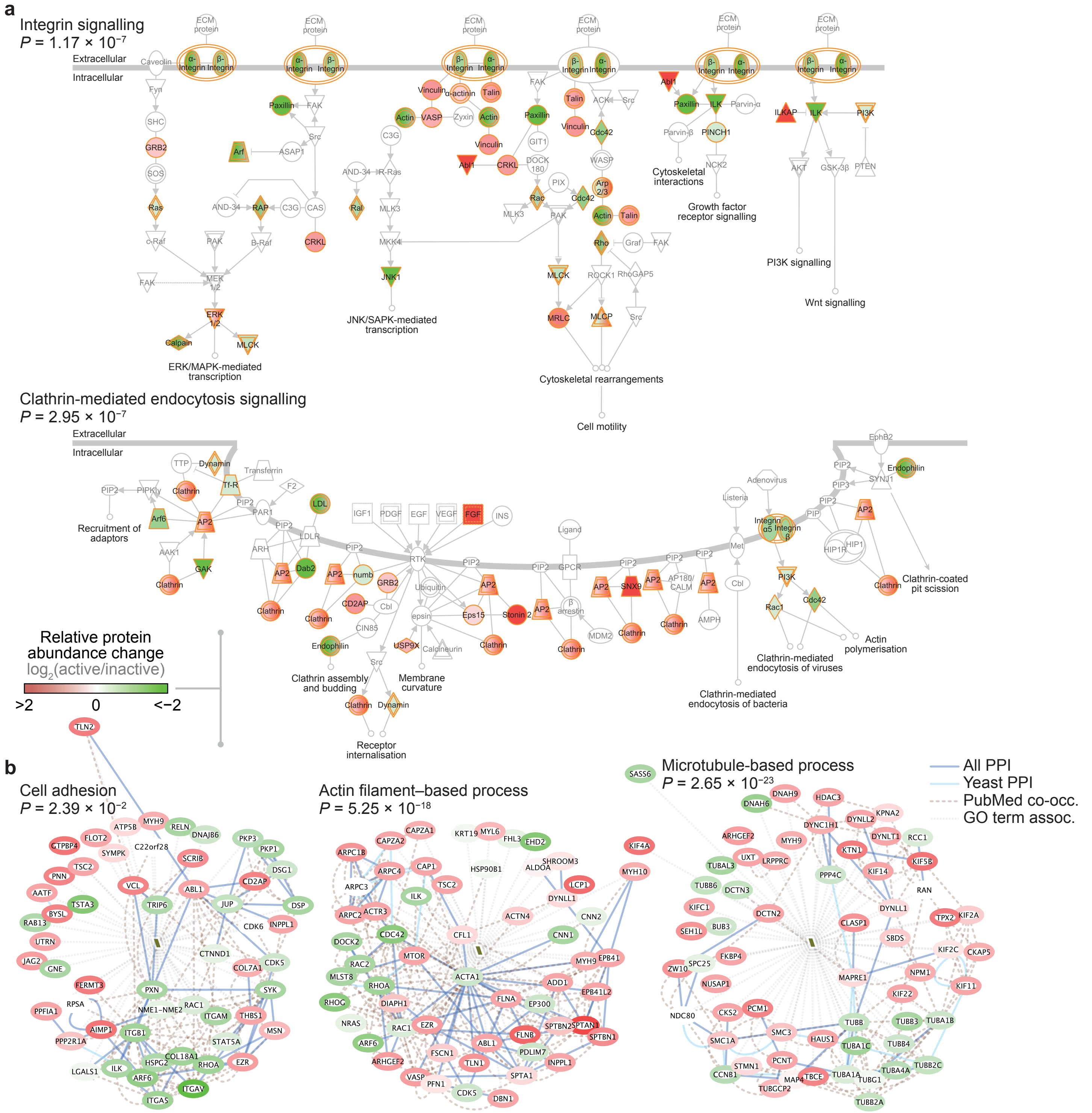

Supplementary Figure 6 | Pathway and functional interaction network analyses of activation state–dependent integrin complexes. (**a**) Overrepresented canonical pathways that contain a number of adhesome proteins were selected ($P < 0.0001$; Fisher's exact test). Coloured nodes (shapes) represent proteins identified by MS and are coloured according to protein enrichment in active (red) or inactive (green) integrin complexes ($\log_2$ transformed). Nodes with multiple colours comprise quantitative contributions from multiple identified subunits or members of a complex or group. Grey nodes represent pathway members not identified in the integrin data sets. Edges (grey lines) represent direct functional interactions (no arrowhead, binding only; pointed arrowhead, acts on, including binding; blunt arrowhead, inhibits, including binding; blunt arrowhead with pointed arrowhead, inhibits and acts on, including binding; circular arrowhead, leads to). (**b**) Functional interaction networks for overrepresented adhesion and cytoskeleton Gene Ontology terms were selected ($P < 0.05$; Fisher's exact test). Nodes (ovals) represent proteins identified by MS, and node borders are coloured according to protein enrichment in active (red) or inactive (green) integrin complexes ($\log_2$ transformed). Edges (lines) represent reported protein-protein interactions (blue solid lines), literature co-occurrence (brown dashed lines) or Gene Ontology term association (grey dotted lines). A number of the adhesion-related enzymes in the integrin signalling pathway (**a**) and the cell adhesion and actin filament networks (**b**) (e.g. RhoA, Rac1, Cdc42 and Arf6) were enriched in inactive integrin complexes. Furthermore, excluding tubulin, most (41 proteins; 80%) of the proteins in the overrepresented microtubule-based process network (**b**) were enriched in active integrin complexes.

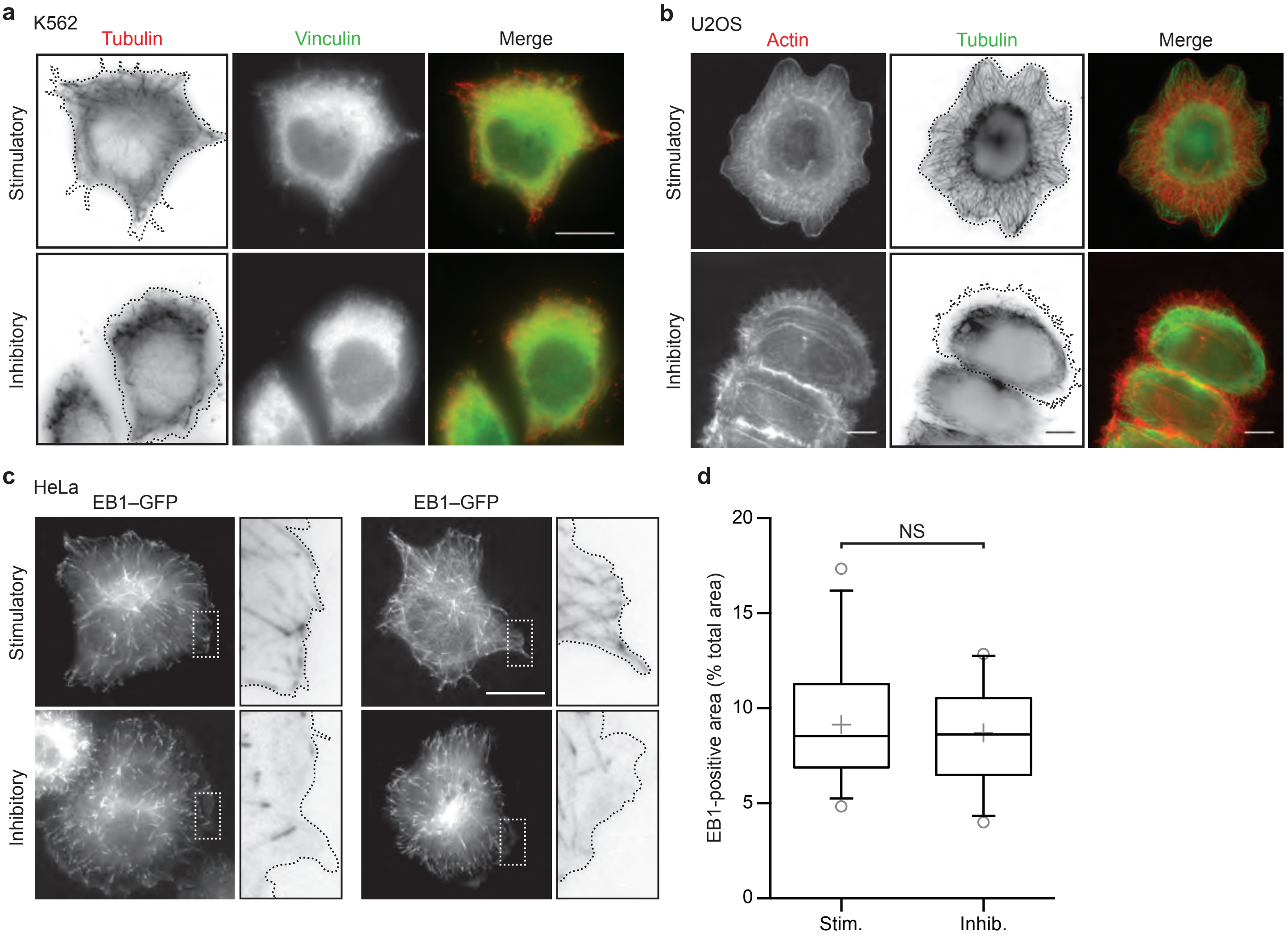

Supplementary Figure 7 | Microtubule growth in cells spread on stimulatory and inhibitory anti-β1 integrin mAbs. (**a**) K562 cells were stained for α-tubulin (red) and vinculin (green). (**b**) U2OS cells were stained for actin (red) and α-tubulin (green). (**c**) HeLa cells expressing EB1–GFP were imaged to visualise microtubule plus ends. Two representative images are shown. Inverted contrast images correspond to areas highlighted in white dotted boxes; black dotted lines indicate cell periphery. Scale bars, 10 μm. (**d**) HFF cells were stained for EB1. Regions away from the cell periphery with areas of at least 40 μm^2 were selected at random, and the amount of EB1-positive signal was determined as a percentage of the total measured area. Box-and-whisker plot shows the median (line), mean (plus sign), 25th and 75th percentiles (box) and 5th and 95th percentiles (whiskers) (n = 26 and 29 cell areas for stimulatory and inhibitory, respectively). Comparison between the two independent groups was determined by two-tailed Mann–Whitney test.

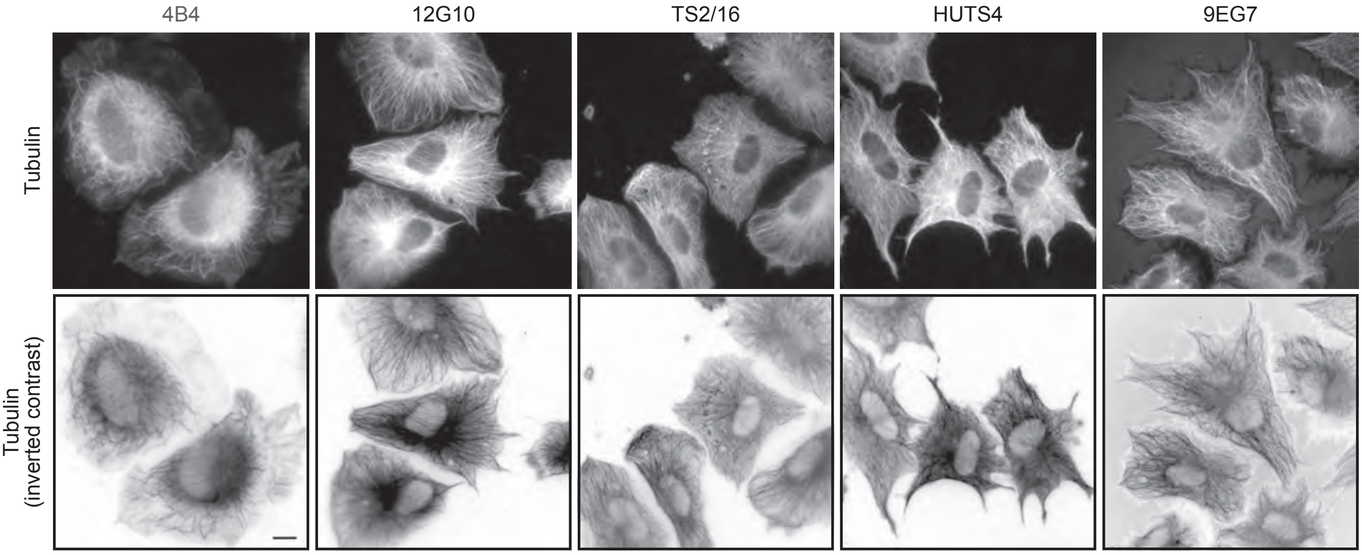

Supplementary Figure 8 | Microtubule growth in cells spread on additional stimulatory anti-β1 integrin mAbs. HFF cells were spread on the inhibitory and stimulatory mAbs used throughout this study (4B4 and 12G10, respectively) in addition to other stimulatory anti-β1 integrin mAbs (TS2/16, HUTS4 and 9EG7). Cells were stained for α-tubulin and imaged to visualise microtubule growth to the cell periphery. Inverted contrast images are also shown. Scale bar, 10 μm.

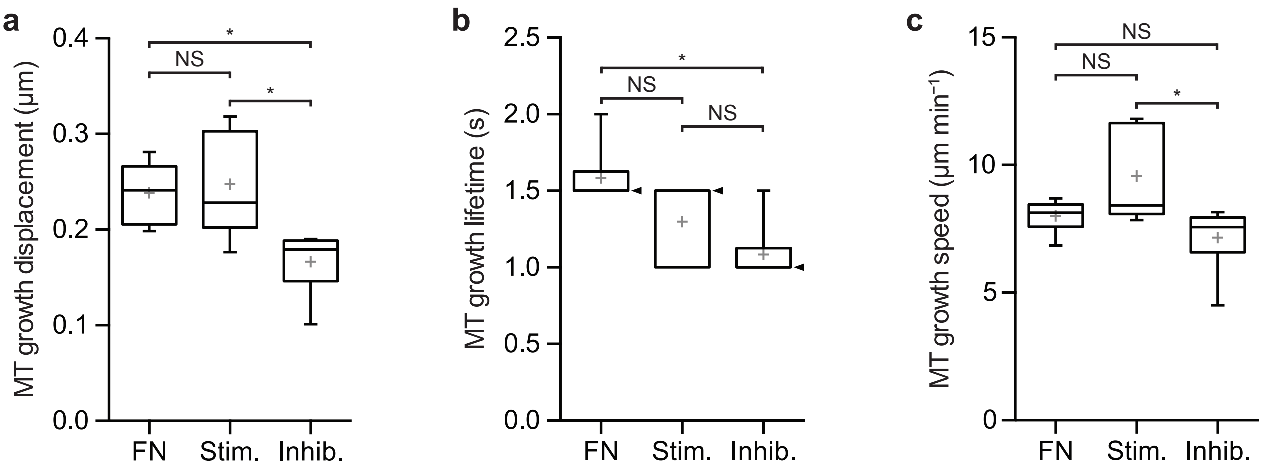

Supplementary Figure 9 | Microtubule growth dynamics in cells spread on activity-specific anti-integrin mAbs. (**a**–**c**) HeLa cells expressing EB3–GFP were spread on FN, stimulatory and inhibitory mAbs and imaged by live TIRF microscopy. Microtubule plus-end dynamics were tracked and analysed using plusTipTracker[4] (see Supplementary Movies 3–5). Median values for displacement (**a**), lifetime (**b**) and speed (**c**) of +TIP comet track growth were determined for each cell from at least 700 tracks per cell. Box-and-whisker plots show the median (line), mean (plus sign), 25th and 75th percentiles (box) and 5th and 95th percentiles (whiskers) (n = 6, 5 and 6 cells for FN, stimulatory and inhibitory, respectively). Arrowheads indicate obscured median lines. *$P < 0.05$; Kruskal–Wallis test with Dunn's multiple comparisons test.

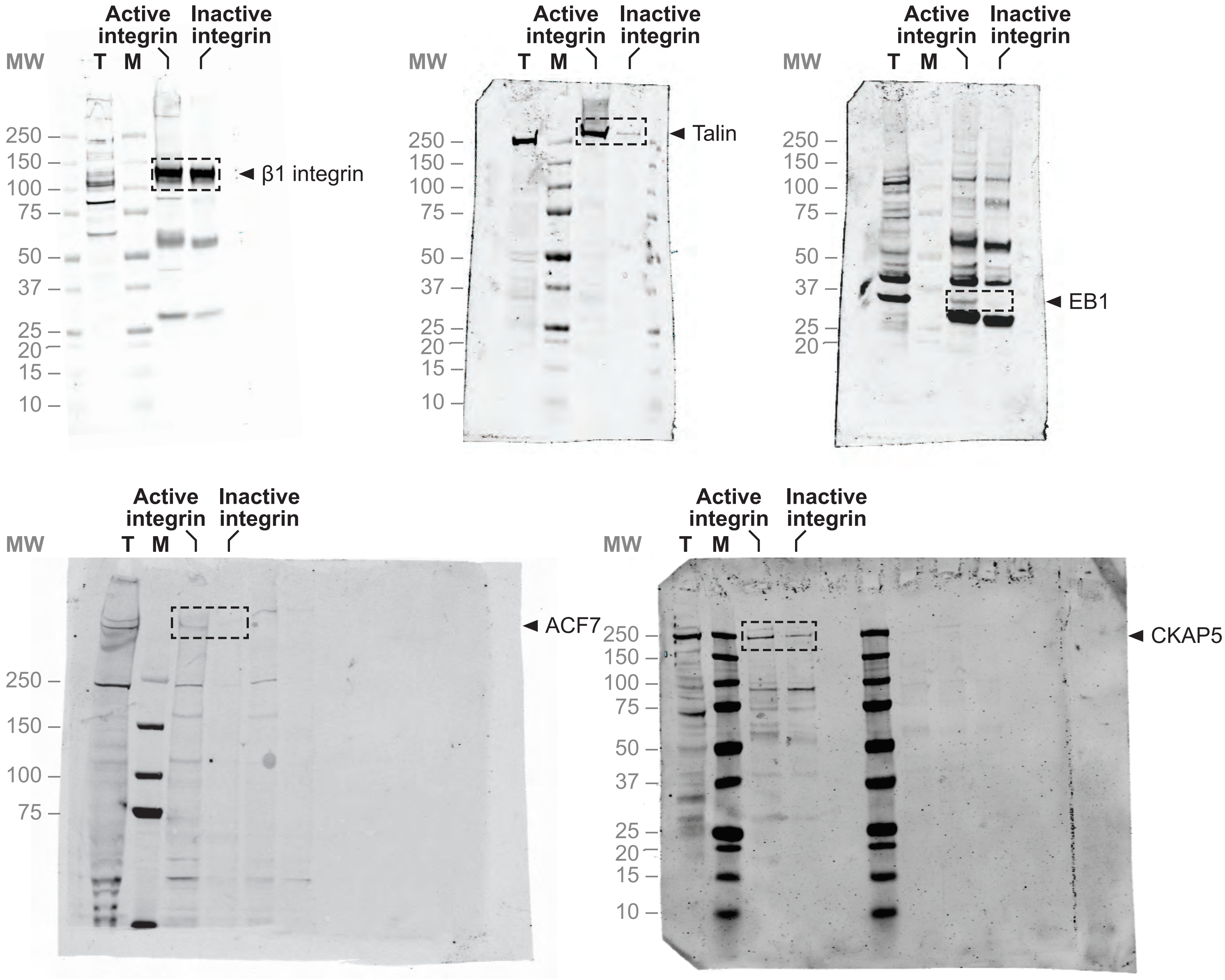

Supplementary Figure 10 | Original blots. Dashed boxes indicate cropped regions shown in Figure 4a. Arrowheads indicate expected band position for each protein probed. MW, molecular weight (kDa), grey font; T, total cell lysate; M, molecular weight marker.

Supplementary Table 1 | Hierarchical clustering analysis of proteins identified in active or inactive integrin adhesion complexes.

#	IPI Accession	UniProtKB Accession	Gene name	Protein name	Protein MW (kDa)	Relative abundance (mean normalised spectral count) ×10³		Fold enrichment (active/inactive integrin)[1]	Specific enrichment[2]		Cluster[3]	Adhesome[4]	Hierarchical clustering
						Active integrin	Inactive integrin		Active integrin	Inactive integrin			
252	IPI00219018	P04406	GAPDH	Glyceraldehyde-3-phosphate dehydrogenase	36	2.036	2.266	0.90	False	False	Unenriched	–	
406	IPI00019380	Q09161	NCBP1	Nuclear cap-binding protein subunit 1	92	0.521	0.580	0.90	False	False	Unenriched	–	
62	IPI00217473	P02008	HBZ	Hemoglobin subunit zeta	16	14.302	15.861	0.90	False	False	Unenriched	–	
237	IPI00012493	P60866	RPS20	40S ribosomal protein S20	13	5.655	6.277	0.90	False	False	Unenriched	–	
1325	IPI00607685	Q9NZJ9	NUDT4	Isoform 1 of Diphosphoinositol polyphosphate phosphohydrolase 2	20	0.324	0.359	0.90	False	False	Unenriched	–	
246	IPI00031517	Q14566	MCM6	DNA replication licensing factor MCM6	93	0.835	0.910	0.92	False	False	Unenriched	–	
434	IPI00010865	P67870	CSNK2B	Casein kinase II subunit beta	25	1.783	1.952	0.91	False	False	Unenriched	–	
611	IPI00025252	P30101	PDIA3	Protein disulfide-isomerase A3	57	0.523	0.572	0.91	False	False	Unenriched	–	
640	IPI00373877	Q5BKZ1	ZNF326	Isoform 1 of Zinc finger protein 326	66	0.390	0.427	0.91	False	False	Unenriched	–	
39	IPI00215965	P09651	HNRNPA1	Isoform A1-B of Heterogeneous nuclear ribonucleoprotein A1	39	7.671	8.432	0.91	False	False	Unenriched	–	
1369	IPI00004324	O43617	TRAPPC3	Trafficking protein particle complex subunit 3	20	0.414	0.456	0.91	False	False	Unenriched	–	
1220	IPI00107339	Q9BYB4	GNB1L	Isoform 1 of Guanine nucleotide-binding protein subunit beta-like protein 1	36	0.252	0.278	0.91	False	False	Unenriched	–	
90	IPI00376798	P62913	RPL11	Isoform 1 of 60S ribosomal protein L11	20	9.149	9.906	0.92	False	False	Unenriched	–	
201	IPI00008433	P46782	RPS5	40S ribosomal protein S5	23	3.907	4.221	0.93	False	False	Unenriched	–	
1302	IPI00177509	Q8IUR0	TRAPPC5	Trafficking protein particle complex subunit 5	21	0.408	0.441	0.93	False	False	Unenriched	–	
68	IPI00215780	P39019	RPS19	40S ribosomal protein S19	16	13.384	14.196	0.94	False	False	Unenriched	–	
568	IPI00100151	Q9H0D6	XRN2	Isoform 1 of 5'-3' exoribonuclease 2	109	0.204	0.217	0.94	False	False	Unenriched	–	
885	IPI00329650	Q8NFH5	NUP35	Nucleoporin NUP53	35	0.584	0.624	0.94	False	False	Unenriched	–	
1208	IPI00016868	Q9Y4R8	TELO2	Telomere length regulation protein TEL2 homolog	92	0.067	0.072	0.93	False	False	Unenriched	–	
10	IPI00414676	P08238	HSP90AB1	Heat shock protein HSP 90-beta	83	9.928	9.764	1.02	False	False	Unenriched	–	
171	IPI00018350	P33992	MCM5	DNA replication licensing factor MCM5	82	1.344	1.329	1.01	False	False	Unenriched	–	
49	IPI00554648	P05787	KRT8	Keratin, type II cytoskeletal 8	54	6.229	6.216	1.00	False	False	Unenriched	–	
1031	IPI00002895	P63272	SUPT4H1	Transcription elongation factor SPT4	13	0.836	0.834	1.00	False	False	Unenriched	–	
843	IPI00555956	P28070	PSMB4	Proteasome subunit beta type-4	29	0.708	0.704	1.01	False	False	Unenriched	–	
157	IPI00643041	P62826	RAN	GTP-binding nuclear protein Ran	24	5.233	5.082	1.03	False	False	Unenriched	–	
310	IPI00027252	Q99623	PHB2	Prohibitin-2	33	2.064	2.017	1.02	False	False	Unenriched	–	
785	IPI00003588	O43324	EEF1E1	Eukaryotic translation elongation factor 1 epsilon-1	20	0.900	0.881	1.02	False	False	Unenriched	–	
1289	IPI00171665	Q8NFH4	NUP37	Nucleoporin Nup37	37	0.195	0.190	1.03	False	False	Unenriched	–	
1451	IPI00103142	Q8WVJ2	NUDCD2	NudC domain-containing protein 2	18	0.244	0.238	1.03	False	False	Unenriched	–	
118	IPI00419585	P62937	PPIA	Peptidyl-prolyl cis-trans isomerase A	18	8.868	9.275	0.96	False	False	Unenriched	–	
261	IPI00293655	Q92499	DDX1	ATP-dependent RNA helicase DDX1	82	0.976	1.020	0.96	False	False	Unenriched	–	
256	IPI00216044	Q9UKM9	RALY	Isoform 1 of RNA-binding protein Raly	30	2.600	2.709	0.96	False	False	Unenriched	–	
1076	IPI00013241	Q9BZL1	UBL5	Ubiquitin-like protein 5	9	1.679	1.750	0.96	False	False	Unenriched	–	
377	IPI00220706	P69891	HBG1	Hemoglobin subunit gamma-1	16	20.032	20.879	0.96	False	False	Unenriched	–	
1104	IPI00025717	O75431	MTX2	Metaxin-2	30	0.331	0.345	0.96	False	False	Unenriched	–	
1224	IPI00221328	O15247	CLIC2	Chloride intracellular channel protein 2	28	0.365	0.384	0.95	False	False	Unenriched	–	
645	IPI00000811	P28072	PSMB6	Proteasome subunit beta type-6	25	1.141	1.177	0.97	False	False	Unenriched	–	
461	IPI00029744	Q04837	SSBP1	Single-stranded DNA-binding protein, mitochondrial	17	2.499	2.598	0.96	False	False	Unenriched	–	
663	IPI00180954	Q14011	CIRBP	Cold-inducible RNA-binding protein	19	1.274	1.321	0.96	False	False	Unenriched	–	
966	IPI00003217	Q99436	PSMB7	Proteasome subunit beta type-7	30	0.322	0.334	0.96	False	False	Unenriched	–	
482	IPI00003482	Q16698	DECR1	2,4-dienoyl-CoA reductase, mitochondrial	36	1.214	1.226	0.99	False	False	Unenriched	–	
372	IPI00005613	Q01081	U2AF1	Splicing factor U2AF 35 kDa subunit	28	2.026	2.049	0.99	False	False	Unenriched	–	
628	IPI00295485	O95757	HSPA4L	Heat shock 70 kDa protein 4L	94	0.070	0.071	0.99	False	False	Unenriched	–	
147	IPI00016610	Q15365	PCBP1	Poly(rC)-binding protein 1	37	4.024	4.028	1.00	False	False	Unenriched	–	
680	IPI00024719	O14929	HAT1	Histone acetyltransferase type B catalytic subunit	50	0.527	0.528	1.00	False	False	Unenriched	–	
463	IPI00011274	O14979	HNRPDL	Isoform 1 of Heterogeneous nuclear ribonucleoprotein D-like	46	2.010	2.018	1.00	False	False	Unenriched	–	
687	IPI00220219	P35606	COPB2	Coatomer subunit beta'	102	0.225	0.226	1.00	False	False	Unenriched	–	
248	IPI00306369	Q08J23	NSUN2	tRNA (cytosine-5-)-methyltransferase NSUN2	86	0.866	0.879	0.99	False	False	Unenriched	–	
259	IPI00017303	P43246	MSH2	DNA mismatch repair protein Msh2	105	0.676	0.687	0.98	False	False	Unenriched	–	
515	IPI00219757	P09211	GSTP1	Glutathione S-transferase P	23	1.700	1.731	0.98	False	False	Unenriched	–	
408	IPI00011200	O43175	PHGDH	D-3-phosphoglycerate dehydrogenase	57	1.048	1.068	0.98	False	False	Unenriched	–	
1425	IPI00299145	P48668	KRT6C	Keratin, type II cytoskeletal 6C	60	3.669	3.741	0.98	False	False	Unenriched	–	
1834	IPI00015011	Q9NXK8	FBXL12	Isoform 1 of F-box/LRR-repeat protein 12	37	0.058	0.060	0.97	False	False	Unenriched	–	
1924	IPI00909552	Q9H2H8	PPIL3	Peptidyl-prolyl cis-trans isomerase	33	0.065	0.067	0.97	False	False	Unenriched	–	
52	IPI00013296	P62269	RPS18	40S ribosomal protein S18	18	13.833	16.218	0.85	False	False	Unenriched	–	
172	IPI00554788	P05783	KRT18	Keratin, type I cytoskeletal 18	48	3.139	3.703	0.85	False	False	Unenriched	–	
255	IPI00337385	O75400	PRPF40A	Isoform 1 of Pre-mRNA-processing factor 40 homolog A	109	0.473	0.558	0.85	False	False	Unenriched	–	
921	IPI00788612	P48059	LIMS1	Particularly interesting new Cys-His protein	38	0.320	0.378	0.85	False	False	Unenriched	YES	
901	IPI00954798	Q9UQ88	CDK11A	PITSLRE serine/threonine-protein kinaseCDC2L1	90	0.140	0.166	0.84	False	False	Unenriched	–	
134	IPI00301936	Q15717	ELAVL1	ELAV-like protein 1	39	3.563	4.235	0.84	False	False	Unenriched	–	
328	IPI00550363	P37802	TAGLN2	Transgelin-2	22	3.005	3.566	0.84	False	False	Unenriched	–	
1060	IPI00398625	Q86YZ3	HRNR	Hornerin	282	0.040	0.047	0.85	False	False	Unenriched	–	
449	IPI00217966	P00338	LDHA	Isoform 1 of L-lactate dehydrogenase A chain	37	1.160	1.327	0.87	False	False	Unenriched	–	
357	IPI00220038	Q9BXP5	SRRT	Isoform 2 of Serrate RNA effector molecule homolog	100	0.442	0.510	0.87	False	False	Unenriched	–	
772	IPI00000861	Q14847	LASP1	Isoform 1 of LIM and SH3 domain protein 1	30	0.686	0.790	0.87	False	False	Unenriched	YES	
288	IPI00008240	P56192	MARS	Methionyl-tRNA synthetase, cytoplasmic	101	0.692	0.805	0.86	False	False	Unenriched	–	
520	IPI00007346	O43447	PPIH	Peptidyl-prolyl cis-trans isomerase H	19	1.933	2.237	0.86	False	False	Unenriched	–	
1533	IPI00061989	Q96FF9	CDCA5	Sororin	28	0.129	0.149	0.87	False	False	Unenriched	–	
241	IPI00550689	Q9Y3I0	C22orf28	UPF0027 protein C22orf28	55	1.275	1.444	0.88	False	False	Unenriched	–	
873	IPI00025156	Q9UNE7	STUB1	Isoform 1 of STIP1 homology and U box-containing protein 1	35	0.492	0.558	0.88	False	False	Unenriched	–	
251	IPI00012066	Q15366	PCBP2	poly(rC) binding protein 2 isoform b	38	4.271	4.802	0.89	False	False	Unenriched	–	
100	IPI00008438	P46783	RPS10	40S ribosomal protein S10	19	7.035	7.944	0.89	False	False	Unenriched	–	
159	IPI00026271	P62263	RPS14	40S ribosomal protein S14	16	7.624	8.605	0.89	False	False	Unenriched	–	
1379	IPI00465436	P04040	CAT	Catalase	60	0.126	0.142	0.89	False	False	Unenriched	–	
753	IPI00744211	O60573	EIF4E2	Eukaryotic translation initiation factor 4E type 2	28	0.741	0.838	0.88	False	False	Unenriched	–	
773	IPI00030363	P24752	ACAT1	Acetyl-CoA acetyltransferase, mitochondrial	45	0.489	0.553	0.88	False	False	Unenriched	–	
1512	IPI00554538	O14773	TPP1	Putative uncharacterized protein TPP1	60	0.088	0.099	0.89	False	False	Unenriched	–	
1287	IPI00328815	Q86UV5	USP48	USP48 protein	121	0.057	0.064	0.89	False	False	Unenriched	–	
85	IPI00798360	P82979	SARNP	18 kDa protein	18	9.747	11.692	0.83	False	False	Unenriched	–	
717	IPI00016077	O75323	GBAS	Protein NipSnap homolog 2	34	0.642	0.769	0.83	False	False	Unenriched	–	
657	IPI00219217	P07195	LDHB	L-lactate dehydrogenase B chain	37	0.979	1.179	0.83	False	False	Unenriched	–	
458	IPI00017412	P35250	RFC2	Isoform 1 of Replication factor C subunit 2	39	0.907	1.094	0.83	False	False	Unenriched	–	
1127	IPI00219518	P40616	ARL1	ADP-ribosylation factor-like protein 1	20	0.342	0.413	0.83	False	False	Unenriched	–	
320	IPI00013774	Q13547	HDAC1	Histone deacetylase 1	55	0.936	1.144	0.82	False	False	Unenriched	–	
864	IPI00410324	Q3MHD2	LSM12	Isoform 1 of Protein LSM12 homolog	22	0.861	1.047	0.82	False	False	Unenriched	–	
1435	IPI00009949	Q92530	PSMF1	Proteasome inhibitor PI31 subunit	30	0.134	0.165	0.81	False	False	Unenriched	–	
1480	IPI00293350	Q99598	TSNAX	Translin-associated protein X	33	0.144	0.177	0.81	False	False	Unenriched	–	
1382	IPI00251559	Q5VTR2	RNF20	E3 ubiquitin-protein ligase BRE1A	114	0.047	0.058	0.81	False	False	Unenriched	–	
1799	IPI00004416	O43633	CHMP2A	Charged multivesicular body protein 2a	25	0.072	0.088	0.82	False	False	Unenriched	–	
669	IPI00032406	O60884	DNAJA2	DnaJ homolog subfamily A member 2	46	0.424	0.528	0.80	False	False	Unenriched	–	
649	IPI00029733	Q04828	AKR1C1	Aldo-keto reductase family 1 member C1	37	0.596	0.743	0.80	False	False	Unenriched	–	
1457	IPI00027230	P14625	HSP90B1	Endoplasmin	92	0.226	0.282	0.80	False	False	Unenriched	–	
1126	IPI00013180	P41223	BUD31	Protein BUD31 homolog	17	0.555	0.695	0.80	False	False	Unenriched	–	
1300	IPI00291646	Q5JYA1	MTHFD1L	Methylenetetrahydrofolate dehydrogenase (NADP+ dependent) 1-like	106	0.056	0.070	0.80	False	False	Unenriched	–	
313	IPI00012535	P31689	DNAJA1	DnaJ homolog subfamily A member 1	45	1.339	1.649	0.81	False	False	Unenriched	–	
605	IPI00015195	Q12996	CSTF3	Cleavage stimulation factor subunit 3	83	0.316	0.389	0.81	False	False	Unenriched	–	
1319	IPI00009654	Q9Y5R8	TRAPPC1	Trafficking protein particle complex subunit 1	17	0.326	0.402	0.81	False	False	Unenriched	–	
1686	IPI00011107	P48735	IDH2	Isocitrate dehydrogenase [NADP], mitochondrial	51	0.059	0.073	0.81	False	False	Unenriched	–	
783	IPI00006504	Q9NR50	EIF2B3	Isoform 1 of Translation initiation factor eIF-2B subunit gamma	50	0.366	0.453	0.81	False	False	Unenriched	–	
1831	IPI00479905	O96000	NDUFB10	NADH dehydrogenase [ubiquinone] 1 beta subcomplex subunit 10	21	0.165	0.204	0.81	False	False	Unenriched	–	
88	IPI00013452	P07814	EPRS	Bifunctional aminoacyl-tRNA synthetase	171	1.078	0.876	1.23	False	False	Unenriched	–	
367	IPI00909703	P50995	ANXA11	Annexin A11	46	1.512	1.227	1.23	False	False	Unenriched	–	
80	IPI00010896	O00299	CLIC1	Chloride intracellular channel protein 1	27	7.164	5.804	1.23	False	False	Unenriched	–	
278	IPI00027626	P40227	CCT6A	T-complex protein 1 subunit zeta	58	1.326	1.075	1.23	False	False	Unenriched	–	
420	IPI00027350	P32119	PRDX2	Peroxiredoxin-2	22	3.944	3.197	1.23	False	False	Unenriched	–	
32	IPI00011253	P23396	RPS3	40S ribosomal protein S3	27	15.149	12.334	1.23	False	False	Unenriched	–	
293	IPI00031812	P67809	YBX1	Nuclease-sensitive element-binding protein 1	36	2.388	1.944	1.23	False	False	Unenriched	–	
1239	IPI00026965	P61077	UBE2D3	Isoform 1 of Ubiquitin-conjugating enzyme E2 D3	17	0.521	0.425	1.23	False	False	Unenriched	–	
84	IPI00220834	P13010	XRCC5	ATP-dependent DNA helicase 2 subunit 2	83	2.231	1.795	1.24	False	False	Unenriched	–	
1212	IPI00397801	Q5D862	FLG2	Filaggrin-2	248	0.047	0.038	1.24	False	False	Unenriched	–	
774	IPI00011284	P21964	COMT	Isoform Membrane-bound of Catechol O-methyltransferase	30	0.696	0.559	1.25	False	False	Unenriched	–	
231	IPI00216951	P14868	DARS	Aspartyl-tRNA synthetase, cytoplasmic	57	1.720	1.422	1.21	False	False	Unenriched	–	
239	IPI00221093	P08708	RPS17	40S ribosomal protein S17	16	5.742	4.740	1.21	False	False	Unenriched	–	
481	IPI00219622	P25787	PSMA2	Proteasome subunit alpha type-2	26	1.726	1.425	1.21	False	False	Unenriched	–	
500	IPI00009010	Q9UI30	TRMT112	tRNA methyltransferase 112 homolog	14	3.426	2.819	1.22	False	False	Unenriched	–	
644	IPI00003479	P28482	MAPK1	Mitogen-activated protein kinase 1	41	0.824	0.677	1.22	False	False	Unenriched	YES	
1899	IPI00003515	Q15643	TRIP11	Thyroid receptor-interacting protein 11	228	0.009	0.007	1.29	False	False	Unenriched	–	
1150	IPI00301082	P43355	MAGEA1	Melanoma-associated antigen 1	34	0.339	0.278	1.22	False	False	Unenriched	–	
898	IPI00032823	Q9Y3B2	EXOSC1	3'-5' exoribonuclease CSL4 homolog	21	0.988	0.807	1.22	False	False	Unenriched	–	
380	IPI00018146	P27348	YWHAQ	14-3-3 protein theta	28	2.160	1.771	1.22	False	False	Unenriched	–	
1160	IPI00008531	Q9UKL0	RCOR1	REST corepressor 1	53	0.159	0.130	1.22	False	False	Unenriched	–	
1540	IPI00301395	Q9H3G5	CPVL	Probable serine carboxypeptidase CPVL	54	0.119	0.097	1.23	False	False	Unenriched	–	
139	IPI00219153	P35268	RPL22	60S ribosomal protein L22	15	10.537	8.790	1.20	False	False	Unenriched	–	
498	IPI00783872	Q14444	CAPRIN1	Isoform 1 of Caprin-1	78	0.616	0.515	1.20	False	False	Unenriched	–	
315	IPI00017726	Q99714	HSD17B10	Isoform 1 of 3-hydroxyacyl-CoA dehydrogenase type-2	27	2.800	2.326	1.20	False	False	Unenriched	–	
26	IPI00171611	Q71DI3	HIST2H3A	Histone H3.2	15	24.160	20.002	1.21	False	False	Unenriched	–	
1495	IPI00215901	P54819	AK2	Isoform 1 of Adenylate kinase 2, mitochondrial	26	0.183	0.151	1.21	False	False	Unenriched	–	
1692	IPI00021570	O60869	EDF1	Isoform 1 of Endothelial differentiation-related factor 1	16	0.297	0.246	1.21	False	False	Unenriched	–	
1207	IPI00218097	O60271	SPAG9	145 kDa protein	145	0.035	0.029	1.21	False	False	Unenriched	–	
793	IPI00001757	Q9Y5S9	RBM8A	Isoform 1 of RNA-binding protein 8A	20	1.235	1.024	1.21	False	False	Unenriched	–	
1715	IPI00003565	O75832	PSMD10	26S proteasome non-ATPase regulatory subunit 10	24	0.177	0.147	1.20	False	False	Unenriched	–	
99	IPI00008530	P05388	RPLP0	60S acidic ribosomal protein P0	34	5.490	4.243	1.29	False	False	Unenriched	–	
222	IPI00301263	P27708	CAD	CAD protein	243	0.391	0.303	1.29	False	False	Unenriched	–	
113	IPI00010153	P62829	RPL23	60S ribosomal protein L23	15	12.025	9.341	1.29	False	False	Unenriched	–	
445	IPI00029266	P62304	SNRPE	Small nuclear ribonucleoprotein E	11	5.237	4.066	1.29	False	False	Unenriched	–	
851	IPI00419531	Q9P2I0	CPSF2	Cleavage and polyadenylation specificity factor subunit 2	88	0.228	0.177	1.29	False	False	Unenriched	–	
76	IPI00304596	Q15233	NONO	Non-POU domain-containing octamer-binding protein	54	4.309	3.309	1.30	False	False	Unenriched	–	
782	IPI00514908	Q5T749	KPRP	Keratinocyte proline-rich protein	64	0.323	0.248	1.30	False	False	Unenriched	–	
1798	IPI00328200	Q2TAL8	QRICH1	Glutamine-rich protein 1	86	0.025	0.019	1.32	False	False	Unenriched	–	
369	IPI00260715	P35637	FUS	Fus-like protein (Fragment)	53	1.128	0.895	1.26	False	False	Unenriched	–	
154	IPI00024933	P30050	RPL12	Isoform 1 of 60S ribosomal protein L12	18	8.149	6.467	1.26	False	False	Unenriched	–	
761	IPI00029132	Q06187	BTK	Tyrosine-protein kinase BTK	76	0.272	0.216	1.26	False	False	Unenriched	–	
342	IPI00465439	P04075	ALDOA	Fructose-bisphosphate aldolase A	39	1.754	1.397	1.26	False	False	Unenriched	–	
698	IPI00031627	P24928	POLR2A	DNA-directed RNA polymerase II subunit RPB1	217	0.088	0.070	1.26	False	False	Unenriched	–	
74	IPI00784154	P10809	HSPD1	60 kDa heat shock protein, mitochondrial	61	4.256	3.397	1.25	False	False	Unenriched	–	
1119	IPI00025695	O75419	CDC45L	CDC45-related protein	66	0.189	0.151	1.25	False	False	Unenriched	–	
1170	IPI00293533	P37198	NUP62	Nuclear pore glycoprotein p62	53	0.190	0.152	1.25	False	False	Unenriched	–	
126	IPI00025091	P62280	RPS11	40S ribosomal protein S11	18	7.292	5.731	1.27	False	False	Unenriched	–	
516	IPI00008575	Q07666	KHDRBS1	Isoform 1 of KH domain-containing, RNA-binding, signal transduction-associated protein 1	48	0.726	0.570	1.27	False	False	Unenriched	–	
1754	IPI00217949	Q16763	UBE2S	Ubiquitin-conjugating enzyme E2 S	24	0.174	0.137	1.27	False	False	Unenriched	–	
161	IPI00008557	Q9NZI8	IGF2BP1	Insulin-like growth factor 2 mRNA-binding protein 1	63	2.138	1.682	1.27	False	False	Unenriched	–	
1866	IPI00009210	Q9UNI6	DUSP12	Dual specificity protein phosphatase 12	38	0.074	0.058	1.28	False	False	Unenriched	–	
1531	IPI00174852	Q9H944	MED20	Mediator of RNA polymerase II transcription subunit 20	23	0.310	0.243	1.28	False	False	Unenriched	–	
631	IPI00299571	Q15084	PDIA6	Isoform 2 of Protein disulfide-isomerase A6	54	0.664	0.520	1.28	False	False	Unenriched	–	
483	IPI00014238	Q15046	KARS	Isoform Cytoplasmic of Lysyl-tRNA synthetase	68	0.674	0.527	1.28	False	False	Unenriched	–	
2172	IPI00923461	Q92896	GLG1	golgi apparatus protein 1 isoform 2	136	0.019	0.014	1.36	False	False	Unenriched	–	
1698	IPI00908883	P61163	ACTR1A	Alpha-centractin	38	0.066	0.052	1.27	False	False	Unenriched	–	
69	IPI00221092	P62249	RPS16	40S ribosomal protein S16	16	12.323	11.718	1.05	False	False	Unenriched	–	
929	IPI00873326	Q92890	UFD1L	Putative uncharacterized protein UFD1L	39	0.397	0.377	1.05	False	False	Unenriched	–	
249	IPI00012442	Q13283	G3BP1	Ras GTPase-activating protein-binding protein 1	52	1.569	1.496	1.05	False	False	Unenriched	–	
625	IPI00219512	Q9Y5K5	UCHL5	Isoform 2 of Ubiquitin carboxyl-terminal hydrolase isozyme L5	36	0.736	0.702	1.05	False	False	Unenriched	–	
1151	IPI00023529	Q00534	CDK6	Cell division protein kinase 6	37	1.127	1.076	1.05	False	False	Unenriched	–	
292	IPI00334587	Q99729	HNRNPAB	Isoform 2 of Heterogeneous nuclear ribonucleoprotein A/B	36	2.132	2.060	1.03	False	False	Unenriched	–	
528	IPI00011698	O00422	SAP18	Sin3A-associated protein, 18kDa	20	1.336	1.288	1.04	False	False	Unenriched	–	
168	IPI00028888	Q14103	HNRNPD	Isoform 1 of Heterogeneous nuclear ribonucleoprotein D0	38	3.133	3.014	1.04	False	False	Unenriched	–	

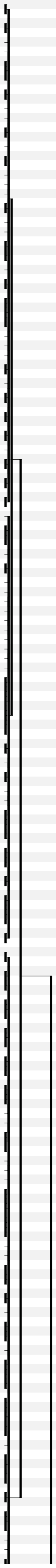

Supplementary Table 1 (cont.) | Hierarchical clustering analysis of proteins identified in active or inactive integrin adhesion complexes.

#	IPI Accession	UniProtKB Accession	Gene name	Protein name	Protein MW (kDa)	Relative abundance (mean normalised spectral count) $\times 10^3$ Active integrin	Relative abundance (mean normalised spectral count) $\times 10^3$ Inactive integrin	Fold enrichment (active/inactive integrin)[1]	Specific enrichment[2] Active integrin	Specific enrichment[2] Inactive integrin	Cluster[3]	Adhesome[4]	Hierarchical clustering
826	IPI00430812	P62633	CNBP	Isoform 1 of Cellular nucleic acid-binding protein	19	1.121	1.078	1.04	False	False	Unenriched	–	
428	IPI00021926	P62333	PSMC6	26S protease regulatory subunit S10B	44	1.054	1.012	1.04	False	False	Unenriched	–	
429	IPI00021926	P62333	PSMC6	26S protease regulatory subunit S10B	44	1.054	1.012	1.04	False	False	Unenriched	–	
641	IPI00007019	Q9Y3C6	PPIL1	Peptidyl-prolyl cis-trans isomerase-like 1	18	1.580	1.514	1.04	False	False	Unenriched	–	
1696	IPI00328658	Q9UBL3	ASH2L	Isoform 1 of Set1/Ash2 histone methyltransferase complex subunit ASH2	69	0.042	0.040	1.05	False	False	Unenriched	–	
43	IPI00418471	P08670	VIM	Vimentin	54	5.789	5.413	1.07	False	False	Unenriched	YES	
1466	IPI00001134	Q9Y580	RBM7	RNA-binding protein 7	31	0.221	0.206	1.07	False	False	Unenriched	–	
186	IPI00026202	Q02543	RPL18A	60S ribosomal protein L18a	21	4.742	4.419	1.07	False	False	Unenriched	–	
2002	IPI00155601	Q9BQ69	MACROD1	MACRO domain-containing protein 1	36	0.098	0.091	1.08	False	False	Unenriched	–	
1017	IPI00016339	P51148	RAB5C	Ras-related protein Rab-5C	23	0.654	0.607	1.08	False	False	Unenriched	–	
658	IPI00172580	Q7Z3B4	NUP54	Nucleoporin 54kDa variant (Fragment)	56	0.496	0.465	1.07	False	False	Unenriched	–	
1415	IPI00017767	Q99735	MGST2	Microsomal glutathione S-transferase 2	17	0.496	0.465	1.07	False	False	Unenriched	–	
784	IPI00063729	Q96J01	THOC3	THO complex subunit 3	42	0.411	0.387	1.06	False	False	Unenriched	–	
1654	IPI00018192	Q6RFH5	WDR74	Isoform 1 of WD repeat-containing protein 74	42	0.099	0.094	1.05	False	False	Unenriched	–	
1125	IPI00005162	O15145	ARPC3	Actin-related protein 2/3 complex subunit 3	21	0.504	0.477	1.06	False	False	Unenriched	YES*	
1316	IPI00219006	Q86WX3	RPS19BP1	Active regulator of SIRT1	15	0.643	0.608	1.06	False	False	Unenriched	–	
57	IPI00413108	P08865	RPSA	33 kDa protein	33	8.620	7.844	1.10	False	False	Unenriched	–	
425	IPI00017334	P35232	PHB	Prohibitin	30	1.745	1.589	1.10	False	False	Unenriched	–	
848	IPI00299048	Q13576	IQGAP2	Isoform 1 of Ras GTPase-activating-like protein IQGAP2	181	0.054	0.049	1.10	False	False	Unenriched	–	
471	IPI00299155	P25789	PSMA4	Proteasome subunit alpha type-4	29	1.527	1.384	1.10	False	False	Unenriched	–	
1305	IPI00017376	Q15437	SEC23B	Protein transport protein Sec23B	86	0.093	0.084	1.11	False	False	Unenriched	–	
1720	IPI00005791	O14777	NDC80	Kinetochore protein NDC80 homolog	74	0.050	0.045	1.11	False	False	Unenriched	–	
314	IPI00382470	P07900	HSP90AA1	Isoform 2 of Heat shock protein HSP 90-alpha	98	4.937	4.433	1.11	False	False	Unenriched	–	
1587	IPI00024698	O60828	PQBP1	Isoform 1 of Polyglutamine-binding protein 1	30	0.170	0.153	1.11	False	False	Unenriched	–	
624	IPI00291946	Q14694	USP10	Ubiquitin carboxyl-terminal hydrolase 10	88	0.350	0.321	1.09	False	False	Unenriched	–	
1089	IPI00217472	P09105	HBQ1	Hemoglobin subunit theta-1	16	0.891	0.816	1.09	False	False	Unenriched	–	
1030	IPI00006754	P61962	DCAF7	DDB1- and CUL4-associated factor 7	39	0.332	0.306	1.08	False	False	Unenriched	–	
1763	IPI00657959			36 kDa protein	36	0.050	0.046	1.09	False	False	Unenriched	–	
133	IPI00419373	P51991	HNRNPA3	Isoform 1 of Heterogeneous nuclear ribonucleoprotein A3	40	3.932	3.377	1.16	False	False	Unenriched	–	
1131	IPI00030781	P42224	STAT1	Isoform Alpha of Signal transducer and activator of transcription 1-alpha/beta	87	0.096	0.083	1.16	False	False	Unenriched	–	
1253	IPI00002966	P34932	HSPA4	Heat shock 70 kDa protein 4	94	0.118	0.101	1.17	False	False	Unenriched	–	
177	IPI00746004	Q71UM5	RPS27L	40S ribosomal protein S27-like	9	15.863	13.558	1.17	False	False	Unenriched	–	
1582	IPI00953925	O00170	AIP	Aryl hydrocarbon receptor interacting protein	38	0.068	0.058	1.17	False	False	Unenriched	–	
541	IPI00031514	P40937	RFC5	Replication factor C subunit 5	38	0.942	0.837	1.13	False	False	Unenriched	–	
728	IPI00219861	P24666	ACP1	Isoform 1 of Low molecular weight phosphotyrosine protein phosphatase	18	1.440	1.278	1.13	False	False	Unenriched	–	
36	IPI00848226	P63244	GNB2L1	Guanine nucleotide-binding protein subunit beta-2-like 1	35	10.477	9.276	1.13	False	False	Unenriched	YES	
1056	IPI00375370	Q53GB2	SEC13	Protein SEC13 homolog	36	0.398	0.351	1.13	False	False	Unenriched	–	
1226	IPI00167572	Q52LJ0	FAM98B	Protein FAM98B	37	0.344	0.304	1.13	False	False	Unenriched	–	
388	IPI00646304	P23284	PPIB	Peptidyl-prolyl cis-trans isomerase B	24	2.347	2.047	1.15	False	False	Unenriched	–	
650	IPI00015833	Q9NX63	CHCHD3	Coiled-coil-helix-coiled-coil-helix domain-containing protein 3, mitochondrial	26	1.135	0.999	1.14	False	False	Unenriched	–	
1087	IPI00005537	P52815	MRPL12	Mitochondrial dicarboxylate carrier	48	0.251	0.220	1.14	False	False	Unenriched	–	
589	IPI00328985	Q86W42	THOC6	Isoform 1 of THO complex subunit 6 homolog	38	0.858	0.741	1.16	False	False	Unenriched	–	
1624	IPI00018235	Q9UBV8	PEF1	Peflin	30	0.170	0.147	1.16	False	False	Unenriched	–	
149	IPI00759560	Q04637	EIF4G1	EIF4G1 protein	172	0.687	0.595	1.15	False	False	Unenriched	–	
1585	IPI00009901	P61970	NUTF2	Nuclear transport factor 2	14	0.216	0.188	1.15	False	False	Unenriched	–	
1674	IPI00155562	Q9BV68	RNF126	Isoform 2 of RING finger protein 126	34	0.178	0.154	1.16	False	False	Unenriched	–	
30	IPI00217471	P02100	HBE1	Hemoglobin subunit epsilon	16	21.577	30.239	0.71	False	False	Unenriched	–	
242	IPI00016832	P25786	PSMA1	Isoform Short of Proteasome subunit alpha type-1	30	2.429	3.400	0.71	False	False	Unenriched	–	
422	IPI00152695	Q6UXN9	WDR82	WD repeat-containing protein 82	35	1.047	1.461	0.72	False	False	Unenriched	–	
1021	IPI00783097	P41250	GARS	Glycyl-tRNA synthetase	83	0.139	0.193	0.72	False	False	Unenriched	–	
273	IPI00011913	Q13151	HNRNPA0	Heterogeneous nuclear ribonucleoprotein A0	31	2.590	3.582	0.72	False	False	Unenriched	–	
780	IPI00010219	Q9HBM1	SPC25	Kinetochore protein Spc25	26	0.684	0.948	0.72	False	False	Unenriched	–	
1146	IPI00019755	P78417	GSTO1	Glutathione S-transferase omega-1	28	0.342	0.475	0.72	False	False	Unenriched	–	
543	IPI00012998	Q16630	CPSF6	Isoform 1 of Cleavage and polyadenylation specificity factor subunit 6	59	0.569	0.814	0.70	False	False	Unenriched	–	
583	IPI00604590	P22392	NME2	Nucleoside diphosphate kinase	33	0.698	1.000	0.70	False	False	Unenriched	–	
637	IPI00005492	P61964	WDR5	WD repeat-containing protein 5	37	0.568	0.811	0.70	False	False	Unenriched	–	
1569	IPI00024915	P30044	PRDX5	Isoform Mitochondrial of Peroxiredoxin-5, mitochondrial	22	0.252	0.358	0.70	False	False	Unenriched	–	
731	IPI00010201	P48556	PSMD8	proteasome 26S non-ATPase subunit 8	40	0.387	0.544	0.71	False	False	Unenriched	–	
925	IPI00304435	Q9BPW8	NIPSNAP1	Protein NipSnap homolog 1	33	0.262	0.368	0.71	False	False	Unenriched	–	
849	IPI00014399	Q13643	FHL3	Four and a half LIM domains protein 3	31	0.474	0.667	0.71	False	False	Unenriched	–	
1080	IPI00019918	Q9NUU7	DDX19A	ATP-dependent RNA helicase DDX19A	54	0.187	0.264	0.71	False	False	Unenriched	–	
87	IPI00376379	Q7Z794	KRT77	keratin 77	62	2.614	3.876	0.67	False	False	Unenriched	–	
1049	IPI00013949	O43765	SGTA	Small glutamine-rich tetratricopeptide repeat-containing protein alpha	34	0.324	0.480	0.68	False	False	Unenriched	–	
282	IPI00550451	P62136	PPP1CA	Serine/threonine-protein phosphatase PP1-alpha catalytic subunit	38	1.726	2.568	0.67	False	False	Unenriched	–	
1048	IPI00010717	Q03426	MVK	Mevalonate kinase	42	0.168	0.250	0.67	False	False	Unenriched	–	
699	IPI00292020	P19623	SRM	Spermidine synthase	34	0.646	0.952	0.68	False	False	Unenriched	–	
903	IPI00002349	Q7Z417	NUFIP2	Nuclear fragile X mental retardation-interacting protein 2	76	0.193	0.285	0.68	False	False	Unenriched	–	
156	IPI00889541	Q92841	DDX17	Isoform 4 of Probable ATP-dependent RNA helicase DDX17	80	3.354	4.927	0.68	False	False	Unenriched	–	
893	IPI00006408	Q9Y314	NOSIP	Nitric oxide synthase-interacting protein	33	0.410	0.602	0.68	False	False	Unenriched	–	
643	IPI00429689	P62714	PPP2CB	Serine/threonine-protein phosphatase 2A catalytic subunit beta isoform	36	0.692	1.014	0.68	False	False	Unenriched	YES	
906	IPI00304693	P52594	AGFG1	Isoform 2 of Arf-GAP domain and FG repeats-containing protein 1	54	0.237	0.348	0.68	False	False	Unenriched	–	
1805	IPI00216308	P21796	VDAC1	Voltage-dependent anion-selective channel protein 1	31	0.098	0.143	0.69	False	False	Unenriched	–	
50	IPI00554676	P69892	HBG2	Hemoglobin subunit gamma-2	16	21.329	30.825	0.69	False	False	Unenriched	–	
279	IPI00246058	Q8WUM4	PDCD6IP	Programmed cell death 6-interacting protein	96	0.627	0.903	0.69	False	False	Unenriched	–	
622	IPI00059292	Q96A72	MAGOHB	Protein mago nashi homolog 2	17	1.343	1.950	0.69	False	False	Unenriched	–	
1038	IPI00013810	O43709	WBSCR22	Isoform 1 of Uncharacterized methyltransferase WBSCR22	32	0.225	0.328	0.69	False	False	Unenriched	–	
1499	IPI00852584	Q96EV2	RBM33	Isoform 1 of RNA-binding protein 33	130	0.028	0.040	0.70	False	False	Unenriched	–	
2076	IPI00783133	Q9HCE5	METTL14	Methyltransferase-like protein 14	52	0.035	0.050	0.70	False	False	Unenriched	–	
220	IPI00012340	Q13242	SFRS9	Splicing factor, arginine/serine-rich 9	26	3.110	3.977	0.78	False	False	Unenriched	–	
16	IPI00396378	P22626	HNRNPA2B1	Isoform B1 of Heterogeneous nuclear ribonucleoproteins A2/B1	37	13.757	17.446	0.79	False	False	Unenriched	–	
447	IPI00028006	P49721	PSMB2	Proteasome subunit beta type-2	23	1.666	2.114	0.79	False	False	Unenriched	–	
907	IPI00013939	P15927	RPA2	Isoform 1 of Replication protein A 32 kDa subunit	29	0.509	0.642	0.79	False	False	Unenriched	–	
327	IPI00031820	Q9Y285	FARSA	Phenylalanyl-tRNA synthetase alpha chain	58	0.960	1.240	0.77	False	False	Unenriched	–	
1438	IPI00418497	Q3ZCQ8	TIMM50	Isoform 2 of Mitochondrial import inner membrane translocase subunit TIM50	50	0.120	0.155	0.77	False	False	Unenriched	–	
752	IPI00011770	O00483	NDUFA4	NADH dehydrogenase [ubiquinone] 1 alpha subcomplex subunit 4	9	2.184	2.817	0.78	False	False	Unenriched	–	
1057	IPI00021167	O75569	PRKRA	Isoform 1 of Interferon-inducible double stranded RNA-dependent protein kinase activator A	34	0.343	0.443	0.77	False	False	Unenriched	–	
721	IPI00305152	O94979	SEC31A	Isoform 3 of Protein transport protein Sec31A	122	0.138	0.180	0.77	False	False	Unenriched	–	
727	IPI00295081	P23258	TUBG1	Tubulin gamma-1 chain	51	0.340	0.443	0.77	False	False	Unenriched	–	
167	IPI00003881	P52597	HNRNPF	Heterogeneous nuclear ribonucleoprotein F	46	3.983	5.186	0.77	False	False	Unenriched	–	
1588	IPI00294486	Q99956	DUSP9	Dual specificity protein phosphatase 9	42	0.096	0.125	0.77	False	False	Unenriched	–	
1574	IPI00180292	Q9UQB8	BAIAP2	Isoform 5 of Brain-specific angiogenesis inhibitor 1-associated protein 2	57	0.071	0.092	0.77	False	False	Unenriched	–	
1811	IPI00029558	O95298	NDUFC2	NADH dehydrogenase [ubiquinone] 1 subunit C2	14	0.180	0.234	0.77	False	False	Unenriched	–	
379	IPI00479145	P08727	KRT19	Keratin, type I cytoskeletal 19	44	4.441	5.868	0.76	False	False	Unenriched	–	
711	IPI00007682	P38606	ATP6V1A	V-type proton ATPase catalytic subunit A	68	0.289	0.382	0.76	False	False	Unenriched	–	
61	IPI00179964	P26599	PTBP1	Isoform 1 of Polypyrimidine tract-binding protein 1	57	3.683	4.855	0.76	False	False	Unenriched	–	
1547	IPI00075081	Q9BXW9	FANCD2	Isoform 1 of Fanconi anemia group D2 protein	166	0.028	0.037	0.76	False	False	Unenriched	–	
792	IPI00218236	P62140	PPP1CB	Serine/threonine-protein phosphatase PP1-beta catalytic subunit	37	1.734	2.275	0.76	False	False	Unenriched	–	
1564	IPI00000606	O95801	TTC4	Tetratricopeptide repeat protein 4	45	0.104	0.137	0.76	False	False	Unenriched	–	
209	IPI00022462	P02786	TFRC	Transferrin receptor protein 1	85	1.000	1.337	0.75	False	False	Unenriched	–	
1149	IPI00106698	Q9BRX2	PELO	Protein pelota homolog	43	0.214	0.287	0.75	False	False	Unenriched	–	
1250	IPI00410590	Q8ND56	LSM14A	Isoform 2 of Protein LSM14 homolog A	51	0.103	0.137	0.75	False	False	Unenriched	–	
676	IPI00939544	P69892	HBG2	17 kDa protein	17	17.414	23.123	0.75	False	False	Unenriched	–	
1152	IPI00009101	Q6ZRS2	SRCAP	Isoform 2 of Helicase SRCAP	337	0.007	0.009	0.78	False	False	Unenriched	–	
391	IPI00024320	P98179	RBM3	Putative RNA-binding protein 3	17	2.999	4.040	0.74	False	False	Unenriched	–	
747	IPI00003927	Q08752	PPID	Peptidyl-prolyl cis-trans isomerase D	41	0.479	0.651	0.74	False	False	Unenriched	–	
476	IPI00009822	P61011	SRP54	Signal recognition particle 54 kDa protein	56	0.675	0.914	0.74	False	False	Unenriched	–	
1176	IPI00162563	O75150	RNF40	Isoform 1 of E3 ubiquitin-protein ligase BRE1B	114	0.054	0.073	0.74	False	False	Unenriched	–	
60	IPI00215637	O00571	DDX3X	ATP-dependent RNA helicase DDX3X	73	3.392	4.641	0.73	False	False	Unenriched	–	
635	IPI00219219	P09382	LGALS1	Galectin-1	15	1.387	1.897	0.73	False	False	Unenriched	–	
504	IPI00014263	Q15056	EIF4H	Isoform Long of Eukaryotic translation initiation factor 4H	27	1.197	1.635	0.73	False	False	Unenriched	–	
1092	IPI00016746	Q13951	CBFB	Isoform 1 of Core-binding factor subunit beta	22	0.504	0.688	0.73	False	False	Unenriched	–	
1663	IPI00016725	Q9UKN8	GTF3C4	General transcription factor 3C polypeptide 4	92	0.035	0.048	0.73	False	False	Unenriched	–	
1901	IPI00031647	Q9BRP1	PDCD2L	Programmed cell death protein 2-like	39	0.083	0.113	0.73	False	False	Unenriched	–	
153	IPI00646917	O43809	NUDT21	Cleavage and polyadenylation specificity factor subunit 5	26	3.791	6.666	0.57	False	False	Unenriched	–	
204	IPI00410714	P69905	HBA1	Hemoglobin subunit alpha	15	4.973	8.710	0.57	False	False	Unenriched	–	
21	IPI00183294	P35658	NUP214	Isoform 1 of Nuclear pore complex protein Nup214	214	1.755	3.037	0.58	False	False	Unenriched	–	
223	IPI00015952	P78344	EIF4G2	Eukaryotic translation initiation factor 4gamma 2	110	0.638	1.113	0.57	False	False	Unenriched	–	
578	IPI00290952	Q14684	RRP1B	Isoform 1 of Ribosomal RNA processing protein 1 homolog B	84	0.219	0.381	0.57	False	False	Unenriched	–	
987	IPI00745433	Q9UKV8	EIF2C2	Isoform 1 of Protein argonaute-2	97	0.113	0.196	0.58	False	False	Unenriched	–	
1370	IPI00010810	P13804	ETFA	Electron transfer flavoprotein subunit alpha, mitochondrial	35	0.193	0.336	0.57	False	False	Unenriched	–	
121	IPI00103994	Q9P2J5	LARS	Leucyl-tRNA synthetase, cytoplasmic	134	0.808	1.382	0.58	False	False	Unenriched	–	
480	IPI00291510	P12268	IMPDH2	Inosine-5'-monophosphate dehydrogenase 2	56	0.618	1.062	0.58	False	False	Unenriched	–	
999	IPI00010860	O00233	PSMD9	Isoform p27-L of 26S proteasome non-ATPase regulatory subunit 9	25	0.458	0.789	0.58	False	False	Unenriched	–	
618	IPI00019383	P51570	GALK1	Galactokinase	45	0.507	0.856	0.59	False	False	Unenriched	–	
211	IPI00018522	Q99873	PRMT1	HMT1 hnRNP methyltransferase-like 2 isoform 1	42	1.902	3.223	0.59	False	False	Unenriched	–	
1203	IPI00016620	Q9H939	PSTPIP2	Isoform 1 of Proline-serine-threonine phosphatase-interacting protein 2	39	0.203	0.345	0.59	False	False	Unenriched	–	
192	IPI00019733	P78406	RAE1	mRNA export factor	41	1.860	3.320	0.56	False	False	Unenriched	–	
733	IPI00009137	O95639	CPSF4	Isoform 1 of Cleavage and polyadenylation specificity factor subunit 4	30	0.586	1.046	0.56	False	False	Unenriched	–	
892	IPI00005724	O43813	LANCL1	LanC-like protein 1	45	0.219	0.391	0.56	False	False	Unenriched	–	
452	IPI00002894	P28340	POLD1	DNA polymerase delta catalytic subunit	124	0.260	0.461	0.56	False	False	Unenriched	–	
411	IPI00221108	P04818	TYMS	Thymidylate synthase	36	1.082	1.919	0.56	False	False	Unenriched	–	
913	IPI00003348	P62879	GNB2	Guanine nucleotide-binding protein G(I)/G(S)/G(T) subunit beta-2	37	0.282	0.500	0.56	False	False	Unenriched	–	
1500	IPI00013981	P07947	YES1	Proto-oncogene tyrosine-protein kinase Yes	61	0.322	0.569	0.57	False	False	Unenriched	–	
759	IPI00012835	Q13363	CTBP1	C-terminal-binding protein 1	48	0.372	0.669	0.56	False	False	Unenriched	–	
1058	IPI00021626	Q9BTD8	RBM42	Isoform 1 of RNA-binding protein 42	50	0.135	0.244	0.55	False	False	Unenriched	–	
346	IPI00025019	P20618	PSMB1	Proteasome subunit beta type-1	26	1.521	2.761	0.55	False	False	Unenriched	–	
1290	IPI00011997	Q9H7E9	C8orf33	Isoform 1 of UPF0488 protein C8orf33	25	0.242	0.439	0.55	False	False	Unenriched	–	
1667	IPI00152658	Q8TDX7	NEK7	Serine/threonine-protein kinase Nek7	35	0.113	0.205	0.55	False	False	Unenriched	–	
114	IPI00783186	O75179	ANKRD17	Isoform 2 of Ankyrin repeat domain-containing protein 17	274	0.424	0.782	0.54	False	False	Unenriched	–	
263	IPI00009867	P13647	KRT5	Keratin, type II cytoskeletal 5	62	3.383	6.253	0.54	False	False	Unenriched	–	
210	IPI00012795	Q13347	EIF3I	Eukaryotic translation initiation factor 3 subunit I	37	1.966	3.640	0.54	False	False	Unenriched	–	
1081	IPI00180675	Q71U36	TUBA1A	Tubulin alpha-1A chain	50	11.234	20.593	0.55	True	False	Unenriched	YES*	
4	IPI00220327	P04264	KRT1	Keratin, type II cytoskeletal 1	66	15.973	29.935	0.53	False	False	Unenriched	–	
1437	IPI00002580	O00443	PIK3C2A	Phosphatidylinositol-4-phosphate 3-kinase C2 domain-containing subunit alpha	191	0.021	0.040	0.53	False	False	Unenriched	YES	
1301	IPI00023095	Q15773	MLF2	Myeloid leukemia factor 2	28	0.257	0.479	0.54	False	False	Unenriched	–	
1777	IPI00010271	P63000	RAC1	Isoform A of Ras-related C3 botulinum toxin substrate 1	21	0.888	1.656	0.54	False	False	Unenriched	YES	
395	IPI00005159	P61160	ACTR2	Actin-related protein 2	45	0.891	1.682	0.53	False	False	Unenriched	–	
882	IPI00018971	P19474	TRIM21	52 kDa Ro protein	54	0.229	0.433	0.53	False	False	Unenriched	–	
926	IPI00298558	Q9BUL8	PDCD10	Programmed cell death protein 10	25	0.354	0.675	0.52	False	False	Unenriched	–	
1251	IPI00028438	Q8IY22	CMIP	c-Maf-inducing protein isoform C-mip	86	0.058	0.110	0.53	False	False	Unenriched	–	
1200	IPI00022239	P53582	METAP1	Methionine aminopeptidase 1	43	0.159	0.304	0.52	False	False	Unenriched	–	
750	IPI00180154	Q99700	ATXN2	Isoform 1 of Ataxin-2	140	0.132	0.254	0.52	False	False	Unenriched	–	
1317	IPI00022820	Q00403	GTF2B	Transcription initiation factor IIB	35	0.150	0.289	0.52	False	False	Unenriched	–	
47	IPI00000875	P26641	EEF1G	Elongation factor 1-gamma	56	3.473	6.867	0.51	False	False	Unenriched	–	
478	IPI00470879	O95758	ROD1	Isoform 1 of Regulator of differentiation 1	57	1.233	2.435	0.51	False	False	Unenriched	–	
1584	IPI00170796	Q9UBQ0	VPS29	Isoform 1 of Vacuolar protein sorting-associated protein 29	21	0.106	0.210	0.50	False	False	Unenriched	–	
107	IPI00013214	P25205	MCM3	MCM3 minichromosome maintenance deficient 3 (S. cerevisiae), isoform CRA_b	96	1.170	2.323	0.50	False	False	Unenriched	–	
1071	IPI00028266	O95067	CCNB2	G2/mitotic-specific cyclin-B2	45	0.179	0.355	0.50	False	False	Unenriched	–	
1326	IPI00450768	Q04695	KRT17	Keratin, type I cytoskeletal 17	48	2.519	4.995	0.50	False	False	Unenriched	–	
175	IPI00003269	Q562R1	ACTBL2	Beta-actin-like protein 2	42	16.135	32.273	0.50	False	False	Unenriched	–	
648	IPI00065671	Q9BZX2	UCK2	Isoform 1 of Uridine-cytidine kinase 2	29	0.745	1.484	0.50	False	False	Unenriched	–	
800	IPI00154451	Q96T76	MMS19	MMS19-like protein	116	0.102	0.203	0.50	False	False	Unenriched	–	
1614	IPI00032140	P50454	SERPINH1	Serpin H1	46	0.078	0.156	0.50	False	False	Unenriched	–	
34	IPI00013881	P31943	HNRNPH1	Heterogeneous nuclear ribonucleoprotein H	49	5.681	11.050	0.51	False	False	Unenriched	–	
1072	IPI00007691	Q9Y296	TRAPPC4	Trafficking protein particle complex subunit 4	24	0.348	0.679	0.51	False	False	Unenriched	–	

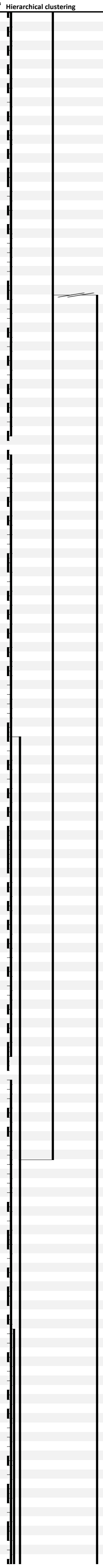

Supplementary Table 1 (cont.) | Hierarchical clustering analysis of proteins identified in active or inactive integrin adhesion complexes.

					Protein	Relative abundance (mean normalised spectral count) ×10[3]		Fold enrichment	Specific enrichment[2]				
#	IPI Accession	UniProtKB Accession	Gene name	Protein name	MW (kDa)	Active integrin	Inactive integrin	(active/inactive integrin)[1]	Active integrin	Inactive integrin	Cluster[3]	Adhesome[4]	Hierarchical clustering
682	IPI00027996	P57737	CORO7	Isoform 1 of Coronin-7	101	0.185	0.361	0.51	False	False	Unenriched	–	
1874	IPI00021739	O75420	GIGYF1	PERQ amino acid-rich with GYF domain-containing protein 1	115	0.018	0.034	0.53	False	False	Unenriched	–	
359	IPI00031570	Q9BSD7	C1orf57	Nucleoside-triphosphatase C1orf57	21	2.157	4.230	0.51	False	False	Unenriched	–	
419	IPI00015947	P25685	DNAJB1	DnaJ homolog subfamily B member 1	38	0.942	1.849	0.51	False	False	Unenriched	–	
1750	IPI00554711	P14923	JUP	Junction plakoglobin	82	0.086	0.168	0.51	False	False	Unenriched	–	
802	IPI00179026	Q9ULC4	MCTS1	Isoform 1 of Malignant T cell-amplified sequence 1	21	0.559	1.100	0.51	False	False	Unenriched	–	
1801	IPI00012833	P60510	PPP4C	Serine/threonine-protein phosphatase 4 catalytic subunit	35	0.072	0.142	0.51	False	False	Unenriched	–	
208	IPI00030783	P42229	STAT5A	Signal transducer and activator of transcription 5A	91	0.747	1.239	0.60	False	False	Unenriched	–	
479	IPI00001738	Q99567	NUP88	Nuclear pore complex protein Nup88	84	0.403	0.667	0.60	False	False	Unenriched	–	
301	IPI00456631	O60341	KDM1	Isoform 1 of Lysine-specific histone demethylase 1	93	0.558	0.918	0.61	False	False	Unenriched	–	
254	IPI00479306	P28074	PSMB5	Proteasome subunit beta type-5	28	2.410	3.976	0.61	False	False	Unenriched	–	
1020	IPI00247295	Q8NF91	SYNE1	Isoform 4 of Nesprin-1	1005	0.002	0.003	0.67	False	False	Unenriched	–	
1024	IPI00007935	Q96HC4	PDLIM5	PDZ and LIM domain protein 5	64	0.116	0.190	0.61	False	False	Unenriched	–	
364	IPI00001661	P18754	RCC1	regulator of chromosome condensation 1 isoform a	48	0.930	1.558	0.60	False	False	Unenriched	–	
704	IPI00010157	P31153	MAT2A	S-adenosylmethionine synthetase isoform type-2	44	0.394	0.662	0.60	False	False	Unenriched	–	
705	IPI00007155	Q9UK41	VPS28	Vacuolar protein sorting-associated protein 28 homolog	25	0.662	1.108	0.60	False	False	Unenriched	–	
143	IPI00083708	Q9Y520	PRRC2C	Isoform 7 of Protein BAT2-like 2	317	0.336	0.560	0.60	False	False	Unenriched	–	
1105	IPI00815713	Q13428	TCOF1	Isoform 4 of Treacle protein	156	0.053	0.089	0.60	False	False	Unenriched	–	
18	IPI00017617	P17844	DDX5	Probable ATP-dependent RNA helicase DDX5	69	5.706	9.123	0.63	False	False	Unenriched	–	
633	IPI00008449	Q6UN15	FIP1L1	Isoform 3 of Pre-mRNA 3'-end-processing factor FIP1	58	0.420	0.675	0.62	False	False	Unenriched	–	
23	IPI00217963	P08779	KRT16	Keratin, type I cytoskeletal 16	51	6.723	10.935	0.61	False	False	Unenriched	–	
188	IPI00300725	P02538	KRT6A	Keratin, type II cytoskeletal 6A	60	4.422	7.197	0.61	False	False	Unenriched	–	
975	IPI00218505	Q8WW33	GTSF1	Gametocyte-specific factor 1	19	0.602	0.982	0.61	False	False	Unenriched	–	
718	IPI00217357	Q8IX12	CCAR1	Isoform 1 of Cell division cycle and apoptosis regulator protein 1	133	0.084	0.136	0.62	False	False	Unenriched	–	
1303	IPI00033030	Q16186	ADRM1	Proteasomal ubiquitin receptor ADRM1	42	0.156	0.253	0.62	False	False	Unenriched	–	
375	IPI00410067	Q7Z2W4	ZC3HAV1	Isoform 1 of Zinc finger CCCH-type antiviral protein 1	101	0.404	0.652	0.62	False	False	Unenriched	–	
1109	IPI00019269	Q9GZS3	WDR61	WD repeat-containing protein 61	34	0.191	0.309	0.62	False	False	Unenriched	–	
444	IPI00217442	Q8IWZ2	hCG_2045902	ANKHD1-EIF4EBP3 protein	277	0.347	0.551	0.63	False	False	Unenriched	–	
732	IPI00165361	Q9BTE7	DCUN1D5	DCN1-like protein 5	28	0.576	0.914	0.63	False	False	Unenriched	–	
874	IPI00007321	O75608	LYPLA1	Acyl-protein thioesterase 1	28	0.586	0.929	0.63	False	False	Unenriched	–	
716	IPI00007611	P48047	ATP5O	ATP synthase subunit O, mitochondrial	23	0.833	1.312	0.63	False	False	Unenriched	–	
181	IPI00795318	P33991	MCM4	DNA replication licensing factor MCM4	93	0.851	1.335	0.64	False	False	Unenriched	–	
1513	IPI00007605	Q9UKK6	NXT1	NTF2-related export protein 1	16	0.315	0.494	0.64	False	False	Unenriched	–	
165	IPI00007188	P05141	SLC25A5	ADP/ATP translocase 2	33	2.981	4.610	0.65	False	False	Unenriched	–	
1225	IPI00165171	Q96DF8	DGCR14	Protein DGCR14	53	0.128	0.198	0.65	False	False	Unenriched	–	
178	IPI00216184	Q13492	PICALM	Isoform 2 of Phosphatidylinositol-binding clathrin assembly protein	69	1.414	2.201	0.64	False	False	Unenriched	–	
1586	IPI00295585	Q9UFW8	CGGBP1	CGG triplet repeat-binding protein 1	19	0.155	0.242	0.64	False	False	Unenriched	–	
714	IPI00514550	Q8N1K8	DNM2	dynamin 2 isoform 2	98	0.164	0.256	0.64	False	False	Unenriched	YES	
1133	IPI00007811	P11802	CDK4	Cell division protein kinase 4	34	1.008	1.574	0.64	False	False	Unenriched	–	
104	IPI00000873	P26640	VARS	Valyl-tRNA synthetase	140	0.982	1.473	0.67	False	False	Unenriched	–	
915	IPI00009253	P54920	NAPA	Alpha-soluble NSF attachment protein	33	0.456	0.682	0.67	False	False	Unenriched	–	
41	IPI00025512	P04792	HSPB1	Heat shock protein beta-1	23	10.911	16.447	0.66	False	False	Unenriched	YES	
531	IPI00828189	P22061	PCMT1	Isoform 2 of Protein-L-isoaspartate(D-aspartate) O-methyltransferase	30	1.073	1.617	0.66	False	False	Unenriched	–	
1375	IPI00398131	Q6ZN17	LIN28B	Isoform 1 of Protein lin-28 homolog B	27	0.149	0.225	0.66	False	False	Unenriched	–	
958	IPI00022373	Q9ULX3	NOB1	RNA-binding protein NOB1	48	0.250	0.377	0.66	False	False	Unenriched	–	
790	IPI00015262	Q99439	CNN2	Calponin-2	34	0.515	0.778	0.66	False	False	Unenriched	–	
345	IPI00002460	P20073	ANXA7	Isoform 1 of Annexin A7	53	1.031	1.573	0.66	False	False	Unenriched	–	
1353	IPI00003309	P52434	POLR2H	DNA-directed RNA polymerases I, II, and III subunit RPABC3	17	0.322	0.492	0.65	False	False	Unenriched	–	
311	IPI00219616	P60891	PRPS1	Ribose-phosphate pyrophosphokinase 1	35	1.590	2.422	0.66	False	False	Unenriched	–	
1351	IPI00816060	Q14475	HBE1	Epsilon-globin (Fragment)	4	13.072	19.914	0.66	False	False	Unenriched	–	
1277	IPI00182469	O60716	CTNND1	Isoform 1AB of Catenin delta-1	107	0.035	0.053	0.66	False	False	Unenriched	–	
1427	IPI00031397	O95573	ACSL3	Long-chain-fatty-acid--CoA ligase 3	80	0.082	0.124	0.66	False	False	Unenriched	–	
1921	IPI00890795	Q8WVI0	C3orf78	UPF0640 protein	9	0.336	0.510	0.66	False	False	Unenriched	–	
798	IPI00291930	Q14677	CLINT1	Clathrin interactor 1	70	0.258	0.396	0.65	False	False	Unenriched	–	
1227	IPI00028059	P49757	NUMB	Isoform 1 of Protein numb homolog	71	0.128	0.196	0.65	False	False	Unenriched	–	
1161	IPI00005040	P11310	ACADM	Isoform 1 of Medium-chain specific acyl-CoA dehydrogenase, mitochondrial	47	0.216	0.331	0.65	False	False	Unenriched	–	
1940	IPI00556369	Q96Q15	SMG1	411 kDa protein	411	0.006	0.009	0.67	False	False	Unenriched	–	
1421	IPI00028160	P08397	HMBS	Isoform 1 of Porphobilinogen deaminase	39	0.111	0.170	0.65	False	False	Unenriched	–	
1503	IPI00001960	Q9Y696	CLIC4	Chloride intracellular channel protein 4	29	0.161	0.248	0.65	False	False	Unenriched	–	
1906	IPI00430803	Q6GMV3	C2orf79	Uncharacterized protein C2orf79	16	0.113	0.173	0.65	False	False	Unenriched	–	
1632	IPI00465211	Q96MX6	WDR92	HZGJ	85	0.030	0.045	0.67	False	False	Unenriched	–	
1637	IPI00000005	P01111	NRAS	GTPase NRas	21	0.189	0.289	0.65	False	False	Unenriched	–	
1	IPI00021440	P63261	ACTG1	Actin, cytoplasmic 2	42	49.453	107.033	0.46	False	False	Inactive	YES	
12	IPI00019359	P35527	KRT9	Keratin, type I cytoskeletal 9	62	8.198	17.771	0.46	False	False	Inactive	–	
358	IPI00290078	P19013	KRT4	keratin 4	64	0.599	1.305	0.46	False	False	Inactive	–	
521	IPI00023530	Q00535	CDK5	Cell division protein kinase 5	33	1.353	2.957	0.46	False	False	Inactive	–	
71	IPI00013468	O43684	BUB3	Isoform 1 of Mitotic checkpoint protein BUB3	37	4.063	8.950	0.45	False	False	Inactive	–	
196	IPI00021428	P68133	ACTA1	Actin, alpha skeletal muscle	42	32.231	70.888	0.45	False	False	Inactive	YES*	
73	IPI00514856	Q14157	UBAP2L	Isoform 1 of Ubiquitin-associated protein 2-like	115	1.319	2.895	0.46	False	False	Inactive	–	
66	IPI00955014	P06493	CDC2	cell division cycle 2 isoform 1	34	4.382	9.630	0.46	False	False	Inactive	–	
933	IPI00217494	Q92540	SMG7	Isoform 1 of Protein SMG7	127	0.091	0.200	0.46	False	False	Inactive	–	
299	IPI00029048	Q14166	TTLL12	Tubulin--tyrosine ligase-like protein 12	74	0.516	1.139	0.45	False	False	Inactive	–	
13	IPI00021304	P35908	KRT2	Keratin, type II cytoskeletal 2 epidermal	66	9.840	22.099	0.45	False	False	Inactive	–	
221	IPI00218693	P07741	APRT	Adenine phosphoribosyltransferase	20	3.223	7.236	0.45	False	False	Inactive	–	
234	IPI00641706	Q9BUF5	TUBB6	46 kDa protein	46	12.385	27.688	0.45	False	False	Inactive	–	
675	IPI00023598	P04350	TUBB4	Tubulin beta-4 chain	50	21.541	48.079	0.45	False	False	Inactive	–	
1237	IPI00021692	Q9NR56	MBNL1	Isoform 1 of Muscleblind-like protein 1	42	1.136	2.535	0.45	False	False	Inactive	–	
1115	IPI00002922	Q9BZK7	TBL1XR1	F-box-like/WD repeat-containing protein TBL1XR1	56	0.132	0.294	0.45	False	False	Inactive	–	
1468	IPI00009480	Q99627	COPS8	COP9 signalosome complex subunit 8	23	0.172	0.383	0.45	False	False	Inactive	–	
723	IPI00908469	Q9BUF5	TUBB6	Tubulin beta-6 chain	47	12.272	27.810	0.44	False	False	Inactive	–	
862	IPI00027485	P06730	EIF4E	Eukaryotic translation initiation factor 4E	25	0.432	0.977	0.44	False	False	Inactive	–	
3	IPI00383296	P52272	HNRNPM	Isoform 2 of Heterogeneous nuclear ribonucleoprotein M	74	17.816	37.612	0.47	False	False	Inactive	–	
5	IPI00396485	P68104	EEF1A1	Elongation factor 1-alpha 1	50	18.176	38.404	0.47	False	False	Inactive	–	
409	IPI00410118	Q96PU8	QKI	Isoform 4 of Protein quaking	40	0.940	1.987	0.47	False	False	Inactive	–	
316	IPI00337397	P52948	NUP98	Isoform 5 of Nuclear pore complex protein Nup98-Nup96	196	0.229	0.482	0.48	False	False	Inactive	–	
7	IPI00792677	P68363	TUBA1B	Tubulin alpha-ubiquitous chain	46	17.880	37.664	0.47	False	False	Inactive	YES	
1444	IPI00478960	Q5T6F2	UBAP2	Ubiquitin associated protein 2 (UBAP2), transcript variant 1	73	0.235	0.494	0.48	False	False	Inactive	–	
992	IPI00473047	P54619	PRKAG1	5'-AMP-ACTIVATED PROTEIN KINASE, GAMMA-1 SUBUNIT	39	0.264	0.557	0.47	False	False	Inactive	YES	
1418	IPI00107122	Q9BVL2	NUPL1	Isoform 1 of Nucleoporin p58/p45	61	0.047	0.100	0.47	False	False	Inactive	–	
418	IPI00375441	Q96AE4	FUBP1	Isoform 1 of Far upstream element-binding protein 1	68	0.777	1.631	0.48	False	False	Inactive	–	
1229	IPI00016513	P61026	RAB10	Ras-related protein Rab-10	23	0.870	1.825	0.48	False	False	Inactive	–	
734	IPI00009841	Q96MX4	EWSR1	Ewing sarcoma breakpoint region 1 isoform 1	69	0.249	0.538	0.46	False	False	Inactive	–	
1086	IPI00005948	Q9BV20	MRI1	Isoform 1 of Methylthioribose-1-phosphate isomerase	39	0.185	0.397	0.47	False	False	Inactive	–	
130	IPI00377261	Q96I24	FUBP3	Isoform 1 of Far upstream element-binding protein 3	62	1.889	4.022	0.47	False	False	Inactive	–	
1697	IPI00020985	Q09472	EP300	Histone acetyltransferase p300	264	0.008	0.017	0.47	False	False	Inactive	–	
170	IPI00550821	Q8N684	CPSF7	Isoform 1 of Cleavage and polyadenylation specificity factor subunit 7	52	1.606	3.330	0.48	False	False	Inactive	–	
270	IPI00456359	Q8WWM7	ATXN2L	Isoform 1 of Ataxin-2-like protein	113	0.420	0.869	0.48	False	False	Inactive	–	
659	IPI00024523	O75190	DNAJB6	Isoform A of DnaJ homolog subfamily B member 6	36	0.530	1.091	0.49	False	False	Inactive	–	
1689	IPI00739539	A5A3E0	POTEF	POTE ankyrin domain family member F	121	1.902	3.917	0.49	False	False	Inactive	–	
412	IPI00329629	Q99615	DNAJC7	DnaJ homolog subfamily C member 7	56	0.645	1.349	0.48	False	False	Inactive	–	
835	IPI00306436	P40763	STAT3	Isoform Del-701 of Signal transducer and activator of transcription 3	88	0.106	0.222	0.48	False	False	Inactive	YES	
494	IPI00009946	Q15785	TOMM34	Mitochondrial import receptor subunit TOM34	35	0.872	1.815	0.48	False	False	Inactive	–	
1169	IPI00015164	O43929	ORC4L	Origin recognition complex subunit 4	50	0.135	0.282	0.48	False	False	Inactive	–	
1158	IPI00027032	O95372	LYPLA2	Acyl-protein thioesterase 2	25	0.199	0.413	0.48	False	False	Inactive	–	
948	IPI00023122	Q9NR12	PDLIM7	Isoform 1 of PDZ and LIM domain protein 7	50	0.177	0.363	0.49	False	False	Inactive	–	
325	IPI00013871	P23921	RRM1	Ribonucleoside-diphosphate reductase large subunit	90	0.448	0.917	0.49	False	False	Inactive	–	
470	IPI00384444	P02533	KRT14	Keratin, type I cytoskeletal 14	52	5.271	10.765	0.49	False	True	Inactive	–	
923	IPI00218529	Q01196	RUNX1	Isoform AML-1I of Runt-related transcription factor 1	50	0.202	0.412	0.49	False	False	Inactive	–	
1398	IPI00025753	Q02413	DSG1	Desmoglein-1	114	0.049	0.099	0.49	False	False	Inactive	–	
2160	IPI00018364	P61225	RAP2B	Ras-related protein Rap-2b	21	0.051	0.105	0.49	False	False	Inactive	–	
594	IPI00027442	P49588	AARS	Alanyl-tRNA synthetase, cytoplasmic	107	0.186	0.379	0.49	False	False	Inactive	–	
1006	IPI00042604	Q9BUT9	FAM195A	Isoform 1 of Protein FAM195A	18	0.608	1.239	0.49	False	False	Inactive	–	
1833	IPI00293665	P04259	KRT6B	Keratin, type II cytoskeletal 6B	60	4.266	8.688	0.49	False	True	Inactive	–	
376	IPI00102856	Q8WU79	SMAP2	Isoform 1 of Stromal membrane-associated protein 2	47	0.703	1.691	0.42	False	False	Inactive	–	
105	IPI00003704	Q9BWF3	RBM4	Isoform 1 of RNA-binding protein 4	40	2.588	6.234	0.42	False	False	Inactive	–	
803	IPI00300026	P50225	SULT1A1	Sulfotransferase 1A1	44	0.172	0.414	0.42	False	False	Inactive	–	
137	IPI00034015	Q92879	CUGBP1	Isoform 1 of CUG-BP- and ETR-3-like factor 1	52	1.695	4.107	0.41	False	False	Inactive	–	
490	IPI00013475	Q13885	TUBB2A	Tubulin beta-2A chain	50	23.452	56.635	0.41	False	False	Inactive	–	
1396	IPI00019182	P78337	PITX1	Pituitary homeobox 1	34	0.133	0.322	0.41	False	False	Inactive	–	
1279	IPI00009702	Q6P2H3	CCDC21	Isoform 2 of Coiled-coil domain-containing protein 21	86	0.059	0.143	0.41	False	False	Inactive	–	
56	IPI00299904	P33993	MCM7	Isoform 1 of DNA replication licensing factor MCM7	81	2.014	4.790	0.42	False	False	Inactive	–	
136	IPI00007752	P68371	TUBB2C	Tubulin beta-2C chain	50	26.511	62.980	0.42	False	False	Inactive	–	
1514	IPI00220648	Q15126	PMVK	Phosphomevalonate kinase	22	0.147	0.350	0.42	False	False	Inactive	–	
110	IPI00013174	Q96PK6	RBM14	Isoform 1 of RNA-binding protein 14	69	1.506	3.602	0.42	False	False	Inactive	–	
2044	IPI00643141	Q92997	DVL3	Segment polarity protein dishevelled homolog DVL-3	78	0.039	0.093	0.42	False	False	Inactive	–	
106	IPI00034049	Q92900	UPF1	Isoform 1 of Regulator of nonsense transcripts 1	124	0.769	1.884	0.41	False	False	Inactive	–	
998	IPI00220578	P08754	GNAI3	Guanine nucleotide-binding protein G(k) subunit alpha	41	0.227	0.554	0.41	False	False	Inactive	–	
244	IPI00644708	Q01085	TIAL1	TIA-1 related protein isoform 2	43	1.182	2.909	0.41	False	False	Inactive	–	
1114	IPI00022434	P02768	ALB	Putative uncharacterized protein ALB	72	7.670	18.873	0.41	False	False	Inactive	–	
706	IPI00015954	Q9NR31	SAR1A	GTP-binding protein SAR1a	22	0.645	1.587	0.41	False	False	Inactive	–	
920	IPI00219910			22 kDa protein	22	0.494	1.216	0.41	False	False	Inactive	–	
232	IPI00784233	Q6Y7W6	GIGYF2	Isoform 1 of PERQ amino acid-rich with GYF domain-containing protein 2	150	0.391	0.971	0.40	False	False	Inactive	–	
1318	IPI00003318	Q9UHR6	ZNHIT2	Zinc finger HIT domain-containing protein 2	43	0.126	0.313	0.40	False	False	Inactive	–	
119	IPI00479186	P14618	PKM2	Isoform M2 of Pyruvate kinase isozymes M1/M2	58	1.820	4.210	0.43	False	False	Inactive	–	
219	IPI00306043	Q9Y5A9	YTHDF2	Isoform 1 of YTH domain family protein 2	62	0.897	2.074	0.43	False	False	Inactive	–	
558	IPI00011285	P07384	CAPN1	Calpain-1 catalytic subunit	82	0.284	0.656	0.43	False	False	Inactive	YES	
432	IPI00028481	P61006	RAB8A	Ras-related protein Rab-8A	24	1.185	2.722	0.44	False	False	Inactive	–	
467	IPI00026230	P55795	HNRNPH2	Heterogeneous nuclear ribonucleoprotein H2	49	3.162	7.264	0.44	False	False	Inactive	–	
2	IPI00909140	P07437	TUBB	Tubulin beta chain	50	32.811	75.554	0.43	False	False	Inactive	–	
14	IPI00009865	P13645	KRT10	Keratin, type I cytoskeletal 10	59	10.343	23.784	0.43	False	False	Inactive	–	
1554	IPI00428954	Q86U70	LDB1	Isoform 3 of LIM domain-binding protein 1	43	0.034	0.077	0.44	False	False	Inactive	–	
1673	IPI00514971	O75935	DCTN3	Isoform 3 of Dynactin subunit 3	18	0.120	0.276	0.43	False	False	Inactive	–	
795	IPI00291398	P31483	TIA1	Isoform Long of Nucleolysin TIA-1 isoform p40	43	0.754	1.774	0.43	False	False	Inactive	–	
1075	IPI00016676	Q15388	TOMM20	Mitochondrial import receptor subunit TOM20 homolog	16	0.446	1.047	0.43	False	False	Inactive	–	
1097	IPI00782992	Q9UQ35	SRRM2	Isoform 1 of Serine/arginine repetitive matrix protein 2	300	0.020	0.048	0.42	False	False	Inactive	–	
539	IPI00032633	Q9Y2Z9	COQ6	Ubiquinone biosynthesis monooxygenase COQ6	51	0.138	0.322	0.43	False	False	Inactive	–	
912	IPI00797945	Q96T88	UHRF1	E3 ubiquitin-protein ligase UHRF1	90	0.118	0.275	0.43	False	False	Inactive	–	
1070	IPI00219953	P30085	CMPK1	UMP-CMP kinase 1 isoform a	26	0.319	0.744	0.43	False	False	Inactive	–	
1772	IPI00432493	Q08117	AES	Amino-terminal enhancer of split	22	0.098	0.229	0.43	False	False	Inactive	–	
15	IPI00479786	Q92945	KHSRP	KH-type splicing regulatory protein	73	5.132	13.047	0.39	False	False	Inactive	–	
356	IPI00410277	O95319	CUGBP2	Isoform 3 of CUG-BP- and ETR-3-like factor 2	56	0.920	2.343	0.39	False	False	Inactive	–	
567	IPI00297455	Q9ULX6	AKAP8L	A-kinase anchor protein 8-like	72	0.251	0.639	0.39	False	False	Inactive	–	
407	IPI00759613	Q8WZ42	TTN	titin isoform N2-A	3713	0.004	0.009	0.44	False	False	Inactive	–	
176	IPI00171127	Q5T6F2	UBAP2	Ubiquitin-associated protein 2	117	0.650	1.669	0.39	False	False	Inactive	–	
1583	IPI00033770	Q9NXW9	ALKBH4	Isoform 1 of Alkylated DNA repair protein alkB homolog 4	34	0.106	0.272	0.39	False	False	Inactive	–	
899	IPI00010433	Q9UPW6	SATB2	DNA-binding protein SATB2	83	0.110	0.282	0.39	False	False	Inactive	–	
1391	IPI00396406	Q9H000	MKRN2	Probable E3 ubiquitin-protein ligase makorin-2	47	0.032	0.082	0.39	False	False	Inactive	–	
144	IPI00013070	Q9BUJ2	HNRNPUL1	Isoform 1 of Heterogeneous nuclear ribonucleoprotein U-like protein 1	96	0.835	2.162	0.39	False	False	Inactive	–	
880	IPI00018434	Q99816	TSG101	Isoform 1 of Tumor susceptibility gene 101 protein	44	0.249	0.642	0.39	False	False	Inactive	–	
189	IPI00217661	Q8IY67	RAVER1	RAVER1	80	0.784	2.038	0.38	False	False	Inactive	YES	
1041	IPI00016381	P51159	RAB27A	Isoform Long of Ras-related protein Rab-27A	25	0.302	0.786	0.38	False	False	Inactive	–	
616	IPI00337426	Q9NSY1	BMP2K	Isoform 1 of BMP-2-inducible protein kinase	129	0.138	0.359	0.38	False	False	Inactive	–	
588	IPI00217920	Q8IZ83	ALDH16A1	Isoform 1 of Aldehyde dehydrogenase family 16 member A1	85	0.236	0.624	0.38	False	False	Inactive	–	
337	IPI00829652	Q9UPN6	SCAF8	Putative RNA-binding protein 16	141	0.244	0.640	0.38	False	False	Inactive	–	
1007	IPI00410684	O95628	CNOT4	Isoform 4 of CCR4-NOT transcription complex subunit 4	71	0.114	0.297	0.38	False	False	Inactive	–	

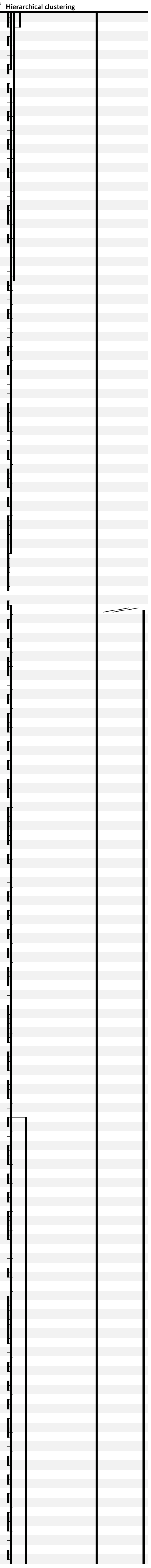

Supplementary Table 1 (cont.) | Hierarchical clustering analysis of proteins identified in active or inactive integrin adhesion complexes.

#	IPI Accession	UniProtKB Accession	Gene name	Protein name	Protein MW (kDa)	Relative abundance (mean normalised spectral count) ×10[3]		Fold enrichment (active/inactive integrin)[1]	Specific enrichment[2]		Cluster[3]	Adhesome[4]
						Active integrin	Inactive integrin		Active integrin	Inactive integrin		
847	IPI00033130	Q9UBE0	SAE1	SUMO-activating enzyme subunit 1	38	0.262	0.683	0.38	False	False	Inactive	–
1493	IPI00375652	Q17RY0	CPEB4	Isoform 1 of Cytoplasmic polyadenylation element-binding protein 4	80	0.044	0.115	0.38	False	False	Inactive	–
155	IPI00165230	Q96EP5	DAZAP1	Isoform 1 of DAZ-associated protein 1	43	1.783	4.813	0.37	False	False	Inactive	–
22	IPI00013877	P31942	HNRNPH3	Isoform 1 of Heterogeneous nuclear ribonucleoprotein H3	37	7.551	20.190	0.37	False	False	Inactive	–
1112	IPI00025084	P04632	CAPNS1	Calpain small subunit 1	28	0.255	0.681	0.37	False	False	Inactive	YES*
297	IPI00026952	Q9Y446	PKP3	Plakophilin-3	87	0.465	1.249	0.37	False	False	Inactive	–
1059	IPI00789324	P14923	JUP	Junction plakoglobin	63	0.752	2.015	0.37	False	True	Inactive	–
510	IPI00290142	P17812	CTPS	CTP synthase 1	67	0.325	0.885	0.37	False	False	Inactive	–
72	IPI00025815	Q13148	TARDBP	TDP43	45	2.792	7.591	0.37	False	False	Inactive	–
949	IPI00328938	Q8WVD3	RNF138	Isoform 1 of E3 ubiquitin-protein ligase RNF138	28	0.357	0.972	0.37	False	False	Inactive	–
142	IPI00946481	Q9NR56	MBNL1	Putative uncharacterized protein MBNL1	36	1.986	5.440	0.37	False	False	Inactive	–
1008	IPI00022597	P61081	UBE2M	NEDD8-conjugating enzyme Ubc12	21	0.384	1.051	0.37	False	False	Inactive	–
1911	IPI00024097	Q9UGI8	TES	Isoform 1 of Testin	48	0.021	0.058	0.36	False	False	Inactive	YES
217	IPI00010700	P48634	BAT2	Isoform 1 of Large proline-rich protein BAT2	229	0.223	0.616	0.36	False	False	Inactive	–
719	IPI00216008	P11413	G6PD	Isoform Long of Glucose-6-phosphate 1-dehydrogenase	64	0.169	0.464	0.36	False	False	Inactive	–
1721	IPI00220079	Q9Y6Q9	NCOA3	Isoform 2 of Nuclear receptor coactivator 3	154	0.007	0.019	0.37	False	False	Inactive	–
1627	IPI00003326	P36404	ARL2	ADP-ribosylation factor-like protein 2	21	0.120	0.330	0.36	False	False	Inactive	–
366	IPI00004273	P49756	RBM25	Isoform 1 of RNA-binding protein 25	100	0.279	0.781	0.36	False	False	Inactive	–
195	IPI00554777	P08243	ASNS	Asparagine synthetase [glutamine-hydrolyzing]	64	0.901	2.515	0.36	False	False	Inactive	–
1003	IPI00305978	O43488	AKR7A2	Aflatoxin B1 aldehyde reductase member 2	40	0.241	0.673	0.36	False	False	Inactive	–
852	IPI00242630	Q86Y56	HEATR2	Isoform 1 of HEAT repeat-containing protein 2	93	0.104	0.291	0.36	False	False	Inactive	–
1557	IPI00304932	O43159	RRP8	Ribosomal RNA-processing protein 8	51	0.042	0.119	0.35	False	False	Inactive	–
382	IPI00789806	P28838	LAP3	Isoform 2 of Cytosol aminopeptidase	53	0.399	1.881	0.21	False	False	Inactive	–
576	IPI00027175	P30626	SRI	Sorcin	22	0.609	2.828	0.22	False	False	Inactive	–
700	IPI00003923	P11172	UMPS	Isoform 1 of Uridine 5'-monophosphate synthase	52	0.174	0.816	0.21	False	False	Inactive	–
44	IPI00306604	P08648	ITGA5	Integrin alpha-5	115	1.082	5.174	0.21	False	True	Inactive	YES
1153	IPI00216805	O94788	ALDH1A2	Isoform 1 of Retinal dehydrogenase 2	57	0.066	0.317	0.21	False	False	Inactive	–
570	IPI00412880	Q86X55	CARM1	Isoform 1 of Histone-arginine methyltransferase CARM1	63	0.194	0.973	0.20	False	False	Inactive	–
1113	IPI00018627	Q9GZZ1	NAT13	Isoform 1 of N-acetyltransferase 13	19	0.231	1.154	0.20	False	False	Inactive	–
1570	IPI00784107	Q96G46	DUS3L	Isoform 1 of tRNA-dihydrouridine synthase 3-like	73	0.020	0.098	0.20	False	False	Inactive	–
38	IPI00217563	P05556	ITGB1	Isoform Beta-1A of Integrin beta-1	88	1.549	7.572	0.20	False	False	Inactive	YES
1716	IPI00658145	Q6PJG6	C7orf27	Isoform 1 of HEAT repeat-containing protein C7orf27	88	0.008	0.040	0.20	False	False	Inactive	–
1193	IPI00008791	O76071	CIAO1	Probable cytosolic iron-sulfur protein assembly protein CIAO1	38	0.047	0.233	0.20	False	False	Inactive	–
1549	IPI00002324	Q9NZL9	MAT2B	Isoform 1 of Methionine adenosyltransferase 2 subunit beta	38	0.047	0.233	0.20	False	False	Inactive	–
967	IPI00022822	P39060	COL18A1	Isoform 2 of Collagen alpha-1(XVIII) chain	154	0.035	0.173	0.20	False	False	Inactive	–
1961	IPI00013160	P41214	LGTN	Isoform 1 of Ligatin	65	0.016	0.076	0.21	False	False	Inactive	–
326	IPI00000897	P42694	HELZ	HELZ protein	219	0.100	0.532	0.19	False	True	Inactive	–
555	IPI00215963	Q14687	GSE1	Isoform 1 of Genetic suppressor element 1	136	0.086	0.455	0.19	False	False	Inactive	–
1235	IPI00013999	P15976	GATA1	Isoform 1 of Erythroid transcription factor	43	0.082	0.443	0.19	False	False	Inactive	–
1352	IPI00010080	O95747	OXSR1	Serine/threonine-protein kinase OSR1	58	0.043	0.238	0.18	False	False	Inactive	–
1760	IPI00300096	Q15286	RAB35	Ras-related protein Rab-35	23	0.219	1.201	0.18	False	False	Inactive	–
740	IPI00023103	O14641	DVL2	Segment polarity protein dishevelled homolog DVL-2	79	0.088	0.454	0.19	False	False	Inactive	–
1542	IPI00884996	Q9C0G6	DNAH6	Isoform 1 of Dynein heavy chain 6, axonemal	476	0.002	0.011	0.18	False	False	Inactive	–
415	IPI00641384	O15027	SEC16A	SEC16 homolog A	252	0.077	0.304	0.25	False	False	Inactive	–
547	IPI00298935	Q7LBC6	KDM3B	Isoform 1 of Lysine-specific demethylase 3B	192	0.060	0.243	0.25	False	False	Inactive	–
548	IPI00184871	Q9Y530	C6orf130	Uncharacterized protein C6orf130	17	0.893	3.616	0.25	False	False	Inactive	–
661	IPI00005780	O15294	OGT	Isoform 3 of UDP-N-acetylglucosamine--peptide N-acetylglucosaminyltransferase 110 kDa subunit	117	0.100	0.382	0.26	False	False	Inactive	–
697	IPI00013933	P15924	DSP	Isoform DPI of Desmoplakin	332	0.027	0.105	0.26	False	False	Inactive	–
1507	IPI00303568	Q9H7Z7	PTGES2	Prostaglandin E synthase 2	42	0.069	0.263	0.26	False	False	Inactive	–
1532	IPI00024802	O14981	BTAF1	TATA-binding protein-associated factor 172	207	0.010	0.037	0.27	False	False	Inactive	–
817	IPI00549389	Q9BV86	METTL11A	Protein of unknown function DUF858, methyltransferase-like family protein	25	0.314	1.211	0.26	False	False	Inactive	–
1154	IPI00924503	Q9H3S7	PTPN23	Tyrosine-protein phosphatase non-receptortype 23	165	0.031	0.118	0.26	False	False	Inactive	–
1047	IPI00020944	P37268	FDFT1	Squalene synthetase	48	0.122	0.474	0.26	False	False	Inactive	–
1360	IPI00008771	Q03014	HHEX	Hematopoietically-expressed homeobox protein HHEX	30	0.084	0.328	0.26	False	False	Inactive	–
1590	IPI00007096	Q9H496	TOR1AIP2	interferon alpha responsive	15	0.101	0.394	0.26	False	False	Inactive	–
305	IPI00022977	P12277	CKB	Creatine kinase B-type	43	0.605	2.756	0.22	False	False	Inactive	–
245	IPI00954006	Q9NTZ6	RBM12	Putative uncharacterized protein DKFZp667H197 (Fragment)	100	0.293	1.327	0.22	False	False	Inactive	–
604	IPI00396218	Q6P3W7	SCYL2	SCY1-like protein 2	104	0.102	0.462	0.22	False	False	Inactive	–
1390	IPI00878754	Q86UA1	PRPF39	Isoform 1 of Pre-mRNA-processing factor 39	78	0.009	0.042	0.21	False	False	Inactive	–
466	IPI00478231	P61586	RHOA	Transforming protein RhoA	22	0.779	3.392	0.23	False	False	Inactive	YES
766	IPI00022184	Q8TB72	PUM2	Isoform 3 of Pumilio homolog 2	114	0.221	0.962	0.23	False	False	Inactive	–
884	IPI00218343	Q9BQE3	TUBA1C	Tubulin alpha-1C chain	50	7.557	32.785	0.23	False	True	Inactive	YES*
672	IPI00395347	Q71RC2	LARP4	Isoform 1 of La-related protein 4	81	0.149	0.659	0.23	False	False	Inactive	–
878	IPI00020454	P27707	DCK	Deoxycytidine kinase	31	0.223	0.980	0.23	False	False	Inactive	–
511	IPI00016669	Q15382	RHEB	GTP-binding protein Rheb	20	0.677	2.968	0.23	False	False	Inactive	–
1928	IPI00024281	O14893	SIP1	Isoform 1 of Survival of motor neuron protein-interacting protein 1	32	0.031	0.138	0.22	False	False	Inactive	–
941	IPI00003505	Q15645	TRIP13	Isoform 1 of Thyroid receptor-interacting protein 13	49	0.109	0.484	0.23	False	False	Inactive	–
1120	IPI00002857	Q16539	MAPK14	Isoform CSBP2 of Mitogen-activated protein kinase 14	41	0.105	0.472	0.22	False	False	Inactive	–
1180	IPI00789069	Q9NVJ2	ARL8B	ADP-ribosylation factor-like protein 8B	21	0.192	0.812	0.24	False	False	Inactive	–
1751	IPI00328846	Q86UY5	FAM83A	Isoform 1 of Protein FAM83A	47	0.043	0.182	0.24	False	False	Inactive	–
1327	IPI00290305	Q96S44	TP53RK	TP53-regulating kinase	28	0.116	0.492	0.24	False	False	Inactive	–
1774	IPI00027838	Q9BQ04	RBM4B	RNA-binding protein 4B	40	0.529	2.244	0.24	False	False	Inactive	–
1664	IPI00013212	P41240	CSK	Tyrosine-protein kinase CSK	51	0.040	0.162	0.25	False	False	Inactive	YES
2080	IPI00013929	P15923	TCF3	Isoform E12 of Transcription factor E2-alpha	68	0.015	0.061	0.25	False	False	Inactive	–
435	IPI00019046	Q9NW64	RBM22	Isoform 1 of Pre-mRNA-splicing factor RBM22	47	0.502	1.492	0.34	False	False	Inactive	–
842	IPI00175096	Q8N5M4	TTC9C	Tetratricopeptide repeat protein 9C	20	0.385	1.143	0.34	False	False	Inactive	–
212	IPI00332371	P17858	PFKL	Isoform 1 of 6-phosphofructokinase, liver type	85	0.634	1.884	0.34	False	False	Inactive	–
914	IPI00745793	P14635	CCNB1	G2/mitotic-specific cyclin-B1	48	0.152	0.450	0.34	False	False	Inactive	–
1838	IPI00219617	P11908	PRPS2	Isoform 1 of Ribose-phosphate pyrophosphokinase 2	35	0.329	0.979	0.34	False	False	Inactive	–
833	IPI00411706	P10768	ESD	S-formylglutathione hydrolase	31	0.235	0.701	0.34	False	False	Inactive	–
775	IPI00007750	P68366	TUBA4A	Tubulin alpha-4A chain	50	7.680	22.899	0.34	False	False	Inactive	–
1752	IPI00102096	Q8IYB5	SMAP1	Isoform 1 of Stromal membrane-associated protein 1	50	0.071	0.210	0.34	False	False	Inactive	–
1128	IPI00168112	Q8NB37	PDDC1	Isoform 1 of Parkinson disease 7 domain-containing protein 1	23	0.204	0.600	0.34	False	False	Inactive	–
561	IPI00019345	P62834	RAP1A	Ras-related protein Rap-1A	21	0.923	2.683	0.34	False	False	Inactive	–
1872	IPI00872071	P50224	SULT1A3	Isoform 1 of Sulfotransferase 1A3/1A4	34	0.085	0.244	0.35	False	False	Inactive	–
148	IPI00247871	O14776	TCERG1	Isoform 1 of Transcription elongation regulator 1	124	0.438	1.416	0.31	False	False	Inactive	–
729	IPI00306057	Q9H0Z9	RBM38	Isoform 1 of RNA-binding protein 38	25	0.443	1.424	0.31	False	False	Inactive	–
499	IPI00030357	P00374	DHFR	Dihydrofolate reductase	21	0.857	2.789	0.31	False	False	Inactive	–
1159	IPI00219628	P05771	PRKCB	Isoform Beta-II of Protein kinase C beta type	77	0.058	0.189	0.31	False	False	Inactive	YES*
1028	IPI00024284	P98160	HSPG2	Basement membrane-specific heparan sulfate proteoglycan core protein	469	0.015	0.049	0.31	False	False	Inactive	–
443	IPI00289499	P31939	ATIC	Bifunctional purine biosynthesis protein PURH	65	0.365	1.132	0.32	False	False	Inactive	–
684	IPI00014808	Q15102	PAFAH1B3	Platelet-activating factor acetylhydrolase IB subunit gamma	26	0.443	1.369	0.32	False	False	Inactive	–
308	IPI00386120	Q15637	SF1	Isoform 6 of Splicing factor 1	62	0.557	1.746	0.32	False	False	Inactive	–
1374	IPI00217355	Q8IWR0	ZC3H7A	Isoform 1 of Zinc finger CCCH domain-containing protein 7A	111	0.027	0.086	0.31	False	False	Inactive	–
660	IPI00221345	Q9BYJ9	YTHDF1	Isoform 1 of YTH domain family protein 1	61	0.412	1.251	0.33	False	False	Inactive	–
825	IPI00215914	P84077	ARF1	ADP-ribosylation factor 1	21	0.453	1.373	0.33	False	False	Inactive	YES
1102	IPI00328737	Q86UK7	ZNF598	Isoform 1 of Zinc finger protein 598	99	0.070	0.213	0.33	False	False	Inactive	–
1771	IPI00290192	O75792	RNASEH2A	Ribonuclease H2 subunit A	33	0.031	0.093	0.33	False	False	Inactive	–
111	IPI00032355	Q14671	PUM1	Pumilio homolog 1 (Drosophila), isoform CRA_c	130	0.659	2.021	0.33	False	False	Inactive	–
1215	IPI00031655	Q9BRG1	VPS25	Vacuolar protein-sorting-associated protein 25	21	0.223	0.684	0.33	False	False	Inactive	–
1989	IPI00018009	Q96F86	EDC3	Enhancer of mRNA-decapping protein 3	56	0.013	0.039	0.33	False	False	Inactive	–
2049	IPI00023736	Q92828	CORO2A	Coronin-2A	60	0.012	0.037	0.32	False	False	Inactive	YES
2250	IPI00012669	Q9H0W8	C19orf61	Isoform 1 of Protein SMG9	58	0.012	0.038	0.32	False	False	Inactive	–
1626	IPI00645078	P22314	UBA1	Ubiquitin-like modifier-activating enzyme 1	118	0.021	0.066	0.32	False	False	Inactive	–
387	IPI00953461	Q92615	LARP4B	La-related protein 4B	81	0.315	1.087	0.29	False	False	Inactive	–
296	IPI00012069	P15559	NQO1	NAD(P)H dehydrogenase [quinone] 1	31	1.126	3.824	0.29	False	False	Inactive	–
291	IPI00009790	Q01813	PFKP	6-phosphofructokinase type C	86	0.568	1.935	0.29	False	False	Inactive	–
550	IPI00465179	P08237	PFKM	phosphofructokinase, muscle isoform 1	93	0.199	0.678	0.29	False	False	Inactive	–
1269	IPI00795676	P62491	RAB11A	22 kDa protein	22	0.177	0.603	0.29	False	False	Inactive	–
1497	IPI00013185	P41229	KDM5C	Isoform 1 of Lysine-specific demethylase 5C	176	0.016	0.053	0.30	False	False	Inactive	–
762	IPI00016373	P51153	RAB13	Ras-related protein Rab-13	23	0.692	2.426	0.29	False	False	Inactive	–
1117	IPI00023234	Q9UBT2	UBA2	SUMO-activating enzyme subunit 2	71	0.056	0.195	0.29	False	False	Inactive	–
414	IPI00010270	P15153	RAC2	Ras-related C3 botulinum toxin substrate 2	21	1.172	4.389	0.27	False	False	Inactive	–
77	IPI00218342	P11586	MTHFD1	C-1-tetrahydrofolate synthase, cytoplasmic	102	0.887	3.295	0.27	False	False	Inactive	–
1658	IPI00216492	P31942	HNRNPH3	Isoform 2 of Heterogeneous nuclear ribonucleoprotein H3	35	4.347	16.155	0.27	False	True	Inactive	–
693	IPI00301561	Q15654	TRIP6	Thyroid receptor-interacting protein 6	50	0.212	0.760	0.28	False	False	Inactive	YES
863	IPI00032955	Q9Y508	RNF114	Isoform 1 of RING finger protein 114	26	0.318	1.142	0.28	False	False	Inactive	–
953	IPI00005719	P62820	RAB1A	Isoform 1 of Ras-related protein Rab-1A	23	0.517	1.849	0.28	False	False	Inactive	–
1931	IPI00184997	Q15398	DLGAP5	discs large homolog 7 isoform b	94	0.011	0.041	0.27	False	False	Inactive	–
2001	IPI00182106	O00255	MEN1	Isoform 2 of Menin	67	0.016	0.058	0.28	False	False	Inactive	–
1482	IPI00073713	Q96DH6	MSI2	Isoform 1 of RNA-binding protein Musashi homolog 2	35	0.051	0.185	0.28	False	False	Inactive	–
751	IPI00022202	Q00325	SLC25A3	Isoform A of Phosphate carrier protein, mitochondrial	40	0.207	0.754	0.27	False	False	Inactive	–
1288	IPI00847896	Q9BQ67	GRWD1	Glutamate-rich WD repeat-containing protein 1	49	0.066	0.241	0.27	False	False	Inactive	–
1837	IPI00876841	A7E2V4	KIAA0913	Isoform 3 of Zinc finger SWIM domain-containing protein KIAA0913	198	0.004	0.013	0.31	False	False	Inactive	–
866	IPI00102897	Q5HYI8	RABL3	Rab-like protein 3	26	0.313	1.149	0.27	False	False	Inactive	–
1636	IPI00030782	P42226	STAT6	Isoform 1 of Signal transducer and activator of transcription 6	94	0.019	0.071	0.27	False	False	Inactive	–
1905	IPI00004397	P11234	RALB	Ras-related protein Ral-B	26	0.069	0.255	0.27	False	False	Inactive	–
216	IPI00383581	Q14697	GANAB	Neutral alpha-glucosidase AB	113	0.208	1.500	0.14	False	False	Inactive	–
1322	IPI00001641	Q9Y5N6	ORC6L	Origin recognition complex subunit 6	28	0.077	0.557	0.14	False	False	Inactive	–
1062	IPI00016786	P60953	CDC42	Isoform 2 of Cell division control protein 42 homolog	21	0.240	1.755	0.14	False	True	Inactive	–
1147	IPI00032426	Q9Y316	MEMO1	Protein MEMO1	34	0.066	0.466	0.14	False	False	Inactive	–
402	IPI00017342	P84095	RHOG	Rho-related GTP-binding protein RhoG	21	0.706	5.285	0.13	False	True	Inactive	–
1657	IPI00401264	Q9BS26	ERP44	Endoplasmic reticulum resident protein 44	47	0.023	0.171	0.13	False	False	Inactive	–
1399	IPI00396131	Q7Z739	YTHDF3	YTH domain protein 3	65	0.155	1.188	0.13	False	True	Inactive	–
776	IPI00289861	Q5TAX3	ZCCHC11	Isoform 1 of Terminal uridylyltransferase 4	185	0.031	0.209	0.15	False	True	Inactive	–
900	IPI00797249	Q7Z4W1	DCXR	26 kDa protein	26	0.174	1.166	0.15	False	True	Inactive	–
1448	IPI00026833	P30520	ADSS	Adenylosuccinate synthetase isozyme 2	50	0.042	0.287	0.15	False	False	Inactive	–
1719	IPI00023729	Q9H479	FN3K	Fructosamine-3-kinase	35	0.029	0.195	0.15	False	False	Inactive	–
485	IPI00553131	Q14376	GALE	UDP-glucose 4-epimerase	38	0.330	2.123	0.16	False	False	Inactive	–
1393	IPI00003949	P61088	UBE2N	Ubiquitin-conjugating enzyme E2 N	17	0.089	0.571	0.16	False	False	Inactive	–
1841	IPI00020898	P51812	RPS6KA3	Ribosomal protein S6 kinase alpha-3	84	0.030	0.191	0.16	False	False	Inactive	–
982	IPI00639879	Q5VT52	RPRD2	Isoform 3 of Regulation of nuclear pre-mRNA domain-containing protein 2	153	0.026	0.172	0.15	False	False	Inactive	–
1543	IPI00216565	A8MPS7	YDJC	Isoform 1 of UPF0249 protein ydjC homolog	34	0.044	0.294	0.15	False	False	Inactive	–
756	IPI00013862	P23919	DTYMK	Thymidylate kinase	24	0.297	1.708	0.17	False	True	Inactive	–
344	IPI00021842	P02649	APOE	Apolipoprotein E	36	0.558	3.197	0.17	False	False	Inactive	–
1321	IPI00178899	Q13309	SKP2	Isoform 1 of S-phase kinase-associated protein 2	48	0.068	0.387	0.18	False	False	Inactive	–
1034	IPI00258168	O43251	RBFOX2	Isoform 8 of RNA-binding protein 9	47	0.086	0.501	0.17	False	False	Inactive	–
1445	IPI00295386	P16152	CBR1	Carbonyl reductase [NADPH] 1	30	0.084	0.493	0.17	False	False	Inactive	–
1753	IPI00385511	Q9UPQ9	TNRC6B	Putative uncharacterized protein TNRC6B	187	0.005	0.032	0.16	False	False	Inactive	–
1511	IPI00792993	Q96PM9	ZNF385A	zinc finger protein 385A isoform a	40	0.027	0.157	0.17	False	False	Inactive	–
755	IPI00025616	P49005	POLD2	DNA polymerase delta subunit 2	51	0.119	0.714	0.17	False	False	Inactive	–
1785	IPI00220424	O75925	PIAS1	Protein inhibitor of activated STAT protein 1	72	0.021	0.128	0.16	False	False	Inactive	–
1453	IPI00853093	Q6P1Q9	METTL2B	Isoform 1 of Methyltransferase-like protein 2B	43	0.047	0.279	0.17	False	False	Inactive	–
1538	IPI00215920	P62330	ARF6	ADP-ribosylation factor 6	20	0.090	0.554	0.16	False	False	Inactive	–
1539	IPI00022018	O60762	DPM1	Dolichol-phosphate mannosyltransferase	30	0.048	0.297	0.16	False	False	Inactive	–
823	IPI00018873	P43490	NAMPT	Nicotinamide phosphoribosyltransferase	56	0.064	0.621	0.10	False	False	Inactive	–
890	IPI00017305	Q15418	RPS6KA1	Ribosomal protein S6 kinase alpha-1	83	0.035	0.339	0.10	False	False	Inactive	–
1284	IPI00302458	Q9UIA9	XPO7	Exportin-7	124	0.012	0.125	0.10	False	True	Inactive	–
1306	IPI00514295	Q9ULV3	CIZ1	CDKN1A interacting zinc finger protein 1 isoform 1	100	0.018	0.184	0.10	False	False	Inactive	–
1345	IPI00168884	O75787	ATP6AP2	Renin receptor	39	0.037	0.368	0.10	False	True	Inactive	–
954	IPI00006451	P46459	NSF	Vesicle-fusing ATPase	83	0.033	0.345	0.10	False	True	Inactive	–
1093	IPI00015148	P61224	RAP1B	Ras-related protein Rap-1b	21	0.326	3.491	0.09	False	True	Inactive	–
1397	IPI00479432	Q8N6R0	METTL13	Isoform 3 of Methyltransferase-like protein 13	69	0.016	0.168	0.10	False	True	Inactive	–
1255	IPI00290684	Q96T60	PNKP	Bifunctional polynucleotide phosphatase/kinase	57	0.025	0.335	0.07	False	False	Inactive	–
917	IPI00026969	Q9Y6Y8	SEC23IP	Isoform 1 of SEC23-interacting protein	111	0.023	0.268	0.09	False	True	Inactive	–

Hierarchical clustering

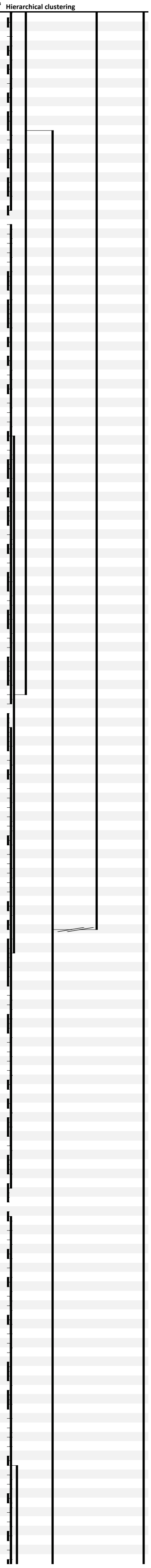

Supplementary Table 1 (cont.) | Hierarchical clustering analysis of proteins identified in active or inactive integrin adhesion complexes.

#	IPI Accession	UniProtKB Accession	Gene name	Protein name	Protein MW (kDa)	Relative abundance (mean normalised spectral count) $\times 10^3$: Active integrin	Relative abundance (mean normalised spectral count) $\times 10^3$: Inactive integrin	Fold enrichment (active/inactive integrin)[1]	Specific enrichment[2]: Active integrin	Specific enrichment[2]: Inactive integrin	Cluster[3]	Adhesome[4]
1661	IPI00027547	P81605	DCD	Dermcidin	11	0.092	1.074	0.09	False	True	Inactive	–
1309	IPI00027434	P08134	RHOC	Rho-related GTP-binding protein RhoC	22	0.229	2.591	0.09	False	True	Inactive	–
1541	IPI00384180	Q86U90	YRDC	YrdC domain-containing protein, mitochondrial	29	0.025	0.286	0.09	False	True	Inactive	–
1055	IPI00184821	Q13057	COASY	Isoform 1 of Bifunctional coenzyme A synthase	62	0.052	0.419	0.12	False	False	Inactive	–
807	IPI00657645	Q8IZH2	XRN1	Isoform 2 of 5'-3' exoribonuclease 1	193	0.029	0.233	0.12	False	True	Inactive	–
1729	IPI00023556	Q9NP77	SSU72	Isoform 1 of RNA polymerase II subunit A C-terminal domain phosphatase SSU72	23	0.047	0.384	0.12	False	False	Inactive	–
940	IPI00411356	Q9UN37	VPS4A	Vacuolar protein sorting-associated protein 4A	49	0.053	0.444	0.12	False	True	Inactive	–
712	IPI00014361	Q13630	TSTA3	GDP-L-fucose synthase	36	0.140	1.212	0.12	False	False	Inactive	–
1515	IPI00018783	Q9BY32	ITPA	Inosine triphosphate pyrophosphatase	21	0.072	0.618	0.12	False	False	Inactive	–
1955	IPI00291928	P61106	RAB14	Ras-related protein Rab-14	24	0.105	0.921	0.11	False	False	Inactive	–
1546	IPI00100980	Q9NZN4	EHD2	EH domain-containing protein 2	61	0.012	0.109	0.11	False	False	Inactive	–
690	IPI00023647	A0AVT1	UBA6	Isoform 1 of Ubiquitin-like modifier-activating enzyme 6	118	0.018	0.356	0.05	False	False	Inactive	–
709	IPI00301204	Q8NBN7	RDH13	Retinol dehydrogenase 13	36	0.082	1.254	0.07	False	True	Inactive	–
1194	IPI00014539	P17482	HOXB9	Homeobox protein Hox-B9	28	0.051	0.768	0.07	False	False	Inactive	–
1085	IPI00029737	O60488	ACSL4	Isoform Long of Long-chain-fatty-acid--CoA ligase 4	79	0.019	0.317	0.06	False	True	Inactive	–
1395	IPI00101782	Q96IJ6	GMPPA	Isoform 1 of Mannose-1-phosphate guanyltransferase alpha	46	0.022	0.358	0.06	False	True	Inactive	–
1385	IPI00409659	Q9UHD9	UBQLN2	Ubiquilin-2	66	0.016	0.259	0.06	False	True	Inactive	–
630	IPI00374068	Q6UY14	ADAMTSL4	Isoform 1 of ADAMTS-like protein 4	117	0.017	0.428	0.04	False	True	Inactive	–
871	IPI00178375	O43314	PPIP5K2	Isoform 2 of Inositol hexakisphosphate and diphosphoinositol-pentakisphosphate kinase 2	138	0.007	0.216	0.03	False	True	Inactive	–
591	IPI00027505	P06756	ITGAV	Isoform 1 of Integrin alpha-V	116	0.009	0.522	0.02	False	True	Inactive	YES
877	IPI00012728	P33121	ACSL1	Isoform 1 of Long-chain-fatty-acid--CoA ligase 1	78	0.013	0.483	0.03	False	True	Inactive	–
888	IPI00742996	Q8ND24	RNF214	RING finger protein 214	78	0.013	0.482	0.03	False	True	Inactive	–
1566	IPI00023876	P55212	CASP6	Isoform Alpha of Caspase-6	33	0	0.233	ND (inactive)	False	True	Inactive	–
1739	IPI00179713	Q9Y6M1	IGF2BP2	Isoform 1 of Insulin-like growth factor 2 mRNA-binding protein 2	66	0	0.293	ND (inactive)	False	True	Inactive	–
1359	IPI00181702	O95104	SFRS15	Isoform 1 of Splicing factor, arginine/serine-rich 15	126	0	0.105	ND (inactive)	False	True	Inactive	–
1608	IPI00011726	O00442	RTCD1	Isoform 1 of RNA 3'-terminal phosphate cyclase	39	0	0.291	ND (inactive)	False	True	Inactive	–
1274	IPI00006050	Q9Y4C2	FAM115A	Isoform 1 of Protein FAM115A	102	0	0.152	ND (inactive)	False	True	Inactive	–
1536	IPI00147874	Q9NR45	NANS	Sialic acid synthase	40	0	0.309	ND (inactive)	False	True	Inactive	–
1930	IPI00291800	Q9NRA8	EIF4ENIF1	Isoform 1 of Eukaryotic translation initiation factor 4E transporter	108	0	0.030	ND (inactive)	False	False	Inactive	–
1974	IPI00328798	Q86UU0	BCL9L	Isoform 1 of B-cell CLL/lymphoma 9-like protein	157	0	0.037	ND (inactive)	False	True	Inactive	–
1982	IPI00004924	Q9H4E7	DEF6	Differentially expressed in FDCP 6 homolog	74	0	0.112	ND (inactive)	False	True	Inactive	YES
2166	IPI00294618	Q92733	PRCC	Proline-rich protein PRCC	52	0	0.064	ND (inactive)	False	False	Inactive	–
1599	IPI00744702	Q96EY5	FAM125A	Isoform 1 of Multivesicular body subunit 12A	29	0	0.430	ND (inactive)	False	True	Inactive	–
1977	IPI00031411	Q14517	FAT1	507 kDa protein	507	0	0.009	ND (inactive)	False	True	Inactive	–
1963	IPI00013466	O43681	ASNA1	ATPase ASNA1	39	0	0.142	ND (inactive)	False	True	Inactive	–
1873	IPI00241562	P78509	RELN	reelin isoform a	388	0	0.015	ND (inactive)	False	True	Inactive	–
2071	IPI00002487	P42898	MTHFR	Methylenetetrahydrofolate reductase	75	0	0.096	ND (inactive)	False	True	Inactive	–
1368	IPI00013301	P41273	TNFSF9	Tumor necrosis factor ligand superfamily member 9	27	0	0.675	ND (inactive)	False	True	Inactive	–
1603	IPI00159322	Q9UGU0	TCF20	Isoform 1 of Transcription factor 20	212	0	0.011	ND (inactive)	False	False	Inactive	–
1814	IPI00007182	Q9BVC4	MLST8	Isoform 3 of Target of rapamycin complex subunit LST8	24	0	0.115	ND (inactive)	False	False	Inactive	–
2032	IPI00915008	Q9UBP6	METTL1	34 kDa protein	34	0	0.211	ND (inactive)	False	True	Inactive	–
2062	IPI00100239	Q9H1A3	METTL9	Isoform 1 of Methyltransferase-like protein 9	37	0	0.217	ND (inactive)	False	True	Inactive	–
533	IPI00878440	P43487	RANBP1	Putative uncharacterized protein RANBP1	19	0	0.380	ND (inactive)	False	False	Inactive	–
1206	IPI00021328	Q6NXR0	IRGC	Interferon-inducible GTPase 5	50	0	0.053	ND (inactive)	False	False	Inactive	–
1467	IPI00001735	Q9Y618	NCOR2	Isoform 1 of Nuclear receptor corepressor 2	275	0	0.025	ND (inactive)	False	True	Inactive	–
1530	IPI00552671	Q9UIW2	PLXNA1	Plexin-A1	211	0	0.045	ND (inactive)	False	True	Inactive	–
939	IPI00026268	P62873	GNB1	Guanine nucleotide-binding protein G(I)/G(S)/G(T) subunit beta-1	37	0	0.071	ND (inactive)	False	False	Inactive	–
258	IPI00382804	Q14222	EEF1A1	EEF1A protein (Fragment)	24	0	4.266	ND (inactive)	False	False	Inactive	–
2257	IPI00002149	Q9Y6B6	SAR1B	GTP-binding protein SAR1b	22	0	0.377	ND (inactive)	False	False	Inactive	–
1168	IPI00413730	Q9NR19	ACSS2	Acetyl-coenzyme A synthetase, cytoplasmic	79	0	0.280	ND (inactive)	False	True	Inactive	–
492	IPI00218030	Q6PFW1	PPIP5K1	Isoform 5 of Inositol hexakisphosphate and diphosphoinositol-pentakisphosphate kinase 1	133	0	0.084	ND (inactive)	False	False	Inactive	–
2245	IPI00023137	O14657	TOR1B	Torsin-1B	38	0	0.073	ND (inactive)	False	False	Inactive	–
1693	IPI00215919	P84085	ARF5	ADP-ribosylation factor 5	21	0	0.552	ND (inactive)	False	False	Inactive	–
770	IPI00930130	Q9BUF5	TUBB6	Tubulin beta-2C chain	45	0	60.600	ND (inactive)	False	True	Inactive	–
2258	IPI00015671	A6NHL2	TUBAL3	Isoform 1 of Tubulin alpha chain-like 3	50	0	1.502	ND (inactive)	False	False	Inactive	–
2205	IPI00011488	Q13043	STK4	Isoform 1 of Serine/threonine-protein kinase 4	56	0	0.069	ND (inactive)	False	True	Inactive	–
1230	IPI00215918	P18085	ARF4	ADP-ribosylation factor 4	21	0	2.065	ND (inactive)	False	True	Inactive	–
2225	IPI00306439	Q9BVV7	C18orf55	TIM21-like protein, mitochondrial	28	0	0.118	ND (inactive)	False	False	Inactive	–
2256	IPI00024502	Q9NRR5	UBQLN4	Ubiquilin-4	64	0	0.086	ND (inactive)	False	False	Inactive	–
2182	IPI00643808	Q9NZJ0	DTL	Isoform 1 of Denticleless protein homolog	79	0	0.056	ND (inactive)	False	True	Inactive	–
1768	IPI00014311	Q13617	CUL2	Cullin-2	90	0	0.098	ND (inactive)	False	True	Inactive	–
2211	IPI00004567	Q9NVM4	PRMT7	Isoform 1 of Protein arginine N-methyltransferase 7	78	0	0.042	ND (inactive)	False	False	Inactive	–
2249	IPI00939594	Q7OIA6	MOB2	Isoform 1 of Mps one binder kinase activator-like 2	27	0	0.082	ND (inactive)	False	False	Inactive	–
1054	IPI00031420	O60701	UGDH	UDP-glucose 6-dehydrogenase	55	0	0.581	ND (inactive)	False	True	Inactive	–
2117	IPI00292827	Q9P289	MST4	Serine/threonine-protein kinase MST4	47	0	0.047	ND (inactive)	False	False	Inactive	–
1726	IPI00182938	O43865	AHCYL1	Isoform 1 of Putative adenosylhomocysteinase 2	59	0	0.103	ND (inactive)	False	True	Inactive	–
1951	IPI00023162	Q9Y223	GNE	Bifunctional UDP-N-acetylglucosamine 2-epimerase/N-acetylmannosamine kinase	79	0	0.098	ND (inactive)	False	True	Inactive	–
1956	IPI00024540	Q9NR46	SH3GLB2	Isoform 1 of Endophilin-B2	44	0	0.085	ND (inactive)	False	False	Inactive	–
1647	IPI00008475	Q01581	HMGCS1	Hydroxymethylglutaryl-CoA synthase, cytoplasmic	57	0	0.194	ND (inactive)	False	True	Inactive	–
1728	IPI00071509	Q13835	PKP1	Isoform 2 of Plakophilin-1	83	0	0.060	ND (inactive)	False	True	Inactive	–
1404	IPI00060149	Q96C06	IRAK1	interleukin-1 receptor-associated kinase 1 isoform 3	68	0	0.284	ND (inactive)	False	True	Inactive	–
2159	IPI00219067	P28161	GSTM2	Glutathione S-transferase Mu 2	26	0	0.085	ND (inactive)	False	False	Inactive	–
1875	IPI00295741	P07858	CTSB	Cathepsin B	38	0	0.167	ND (inactive)	False	True	Inactive	–
2045	IPI00001640	Q9Y5N5	N6AMT1	N(6)-adenine-specific DNA methyltransferase 1	23	0	0.168	ND (inactive)	False	True	Inactive	–
2046	IPI00001640	Q9Y5N5	N6AMT1	N(6)-adenine-specific DNA methyltransferase 1	23	0	0.168	ND (inactive)	False	True	Inactive	–
1678	IPI00477227	Q8N1N4	KRT78	Isoform 1 of Keratin, type II cytoskeletal 78	57	0	0.587	ND (inactive)	False	False	Inactive	–
1980	IPI00157365	Q9Y5A7	NUB1	NEDD8 ultimate buster 1 (Negative regulator of ubiquitin-like proteins 1) (Renal carcinoma antigen NY-REN-18). Isoform 2	72	0	0.107	ND (inactive)	False	True	Inactive	–
1651	IPI00220030	P49023	PXN	Isoform Alpha of Paxillin	61	0	0.136	ND (inactive)	False	True	Inactive	YES
957	IPI00013232	Q13422	IKZF1	Isoform Ik1 of DNA-binding protein Ikaros	58	0	0.067	ND (inactive)	False	False	Inactive	–
1789	IPI00954527	P62736	ACTA2	ACTA2 protein (Fragment)	37	0	27.649	ND (inactive)	False	True	Inactive	YES*
1806	IPI00464951	Q9P0W2	HMG20B	Isoform 1 of SWI/SNF-related matrix-associated actin-dependent regulator of chromatin subfamily E member 1-related	36	0	0.082	ND (inactive)	False	False	Inactive	–
1828	IPI00017993	P35326	SPRR2A	Small proline-rich protein 2A	8	0	0.902	ND (inactive)	False	False	Inactive	–
1835	IPI00300086	Q15274	QPRT	Nicotinate-nucleotide pyrophosphorylase [carboxylating]	31	0	0.206	ND (inactive)	False	True	Inactive	–
2255	IPI00166394	Q8N2F6	ARMC10	Isoform 1 of Armadillo repeat-containing protein 10	38	0	0.058	ND (inactive)	False	False	Inactive	–
2261	IPI00031545	Q14571	ITPR2	Isoform Long of Inositol 1,4,5-trisphosphate receptor type 2	308	0	0.011	ND (inactive)	False	False	Inactive	–
2265	IPI00942512	P12035	KRT3	keratin 3	64	0	0.760	ND (inactive)	False	False	Inactive	–
2004	IPI00743931	Q9H8Y8	GORASP2	Golgi reassembly-stacking protein 2	49	0	0.056	ND (inactive)	False	False	Inactive	–
1294	IPI00886911			Actin alpha 1 skeletal muscle protein	51	0	0.051	ND (inactive)	False	False	Inactive	–
2232	IPI00939845	Q9NVE7	PANK4	Pantothenate kinase 4	86	0	0.051	ND (inactive)	False	True	Inactive	–
1770	IPI00007401	O15397	IPO8	Importin-8	120	0	0.087	ND (inactive)	False	True	Inactive	–
1821	IPI00784029	Q9BUP3	HTATIP2	Isoform 1 of Oxidoreductase HTATIP2	27	0	0.225	ND (inactive)	False	True	Inactive	–
1864	IPI00307591	O15014	ZNF609	Zinc finger protein 609	151	0	0.054	ND (inactive)	False	False	Inactive	–
1556	IPI00401900	Q7Z7F0	KIAA0907	Isoform 1 of UPF0469 protein KIAA0907	65	0	0.195	ND (inactive)	False	True	Inactive	–
2110	IPI00022449	Q92608	DOCK2	Isoform 1 of Dedicator of cytokinesis protein 2	212	0	0.013	ND (inactive)	False	False	Inactive	–
2199	IPI00439948	Q8WUF5	PPP1R13L	Isoform 1 of RelA-associated inhibitor	89	0	0.044	ND (inactive)	False	False	Inactive	–
2226	IPI00306439	Q9BVV7	C18orf55	TIM21-like protein, mitochondrial	28	0	0.118	ND (inactive)	False	False	Inactive	–
1917	IPI00174190	Q8WUK0	PTPMT1	Isoform 1 of Protein-tyrosine phosphatase mitochondrial 1	23	0	0.408	ND (inactive)	False	True	Inactive	–
2079	IPI00102128	Q96LA8	PRMT6	Isoform 1 of Protein arginine N-methyltransferase 6	42	0	0.132	ND (inactive)	False	True	Inactive	–
2179	IPI00294787	Q9Y4B4	RAD54L2	Helicase ARIP4	163	0	0.024	ND (inactive)	False	True	Inactive	–
2027	IPI00013909	Q9BRQ8	AIFM2	Isoform 1 of Apoptosis-inducing factor 2	41	0	0.189	ND (inactive)	False	True	Inactive	–
2140	IPI00328268	Q504Z1	EIF4G3	EIF4G3 protein	147	0	0.085	ND (inactive)	False	False	Inactive	–
1010	IPI00004671	Q14789	GOLGB1	Golgin subfamily B member 1	376	0	0.009	ND (inactive)	False	False	Inactive	–
2217	IPI00103487	Q8WX94	NLRP7	Isoform 1 of NACHT, LRR and PYD domains-containing protein 7	112	0	0.020	ND (inactive)	False	False	Inactive	–
1800	IPI00000104	O60942	RNGTT	Isoform 1 of mRNA-capping enzyme	69	0	0.083	ND (inactive)	False	True	Inactive	–
1769	IPI00025447	P68104	EEF1A1	Elongation factor 1-alpha	48	0	7.711	ND (inactive)	False	False	Inactive	–
1868	IPI00397768	Q9HCJ3	RAVER2	Isoform 2 of Ribonucleoprotein PTB-binding 2	73	0	0.132	ND (inactive)	False	True	Inactive	–
1894	IPI00015560	Q6IA86	ELP2	Isoform 1 of Elongator complex protein 2	92	0	0.084	ND (inactive)	False	True	Inactive	–
1518	IPI00152688	Q8TEA1	NSUN6	Putative methyltransferase NSUN6	52	0	0.167	ND (inactive)	False	True	Inactive	–
2223	IPI00028385	Q9BWH2	FUNDC2	Putative uncharacterized protein FUNDC2	15	0	0.184	ND (inactive)	False	False	Inactive	–
1926	IPI00012966	Q99081	TCF12	Transcription factor 12	73	0	0.072	ND (inactive)	False	True	Inactive	–
1981	IPI00016568	P27144	AK4	Adenylate kinase isoenzyme 4, mitochondrial	25	0	0.110	ND (inactive)	False	False	Inactive	–
2029	IPI00746777	P11766	ADH5	Alcohol dehydrogenase class-3	40	0	0.110	ND (inactive)	False	True	Inactive	–
1967	IPI00940874	P49750	YLPM1	Isoform 1 of YLP motif-containing protein 1	220	0	0.027	ND (inactive)	False	True	Inactive	–
2056	IPI00640177	P14859	POU2F1	POU domain, class 2, transcription factor 1	72	0	0.099	ND (inactive)	False	True	Inactive	–
1653	IPI00045839	Q32P28	LEPRE1	Isoform 3 of Prolyl 3-hydroxylase 1	91	0	0.121	ND (inactive)	False	True	Inactive	–
1293	IPI00220070	Q16877	PFKFB4	6-phosphofructo-2-kinase/fructose-2,6-biphosphatase 4	54	0	0.387	ND (inactive)	False	True	Inactive	–
2136	IPI00179438	P98082	DAB2	Isoform 1 of Disabled homolog 2	82	0	0.027	ND (inactive)	False	False	Inactive	–
1680	IPI00289758	P17655	CAPN2	Calpain-2 catalytic subunit	80	0	0.104	ND (inactive)	False	True	Inactive	YES
1682	IPI00375578	Q6UVJ0	SASS6	Spindle assembly abnormal protein 6 homolog	74	0	0.142	ND (inactive)	False	True	Inactive	–
1257	IPI00014474	O43823	AKAP8	A-kinase anchor protein 8	76	0	0.234	ND (inactive)	False	True	Inactive	–
2233	IPI00387168	Q8NBP7	PCSK9	Isoform 1 of Proprotein convertase subtilisin/kexin type 9	74	0	0.052	ND (inactive)	False	True	Inactive	–
1275	IPI00107855	Q9BQS7	HEPH	Isoform 1 of Hephaestin	130	0	0.149	ND (inactive)	False	True	Inactive	–
2236	IPI00020510	Q9NZ45	CISD1	CDGSH iron sulfur domain-containing protein 1	12	0	0.184	ND (inactive)	False	False	Inactive	–
1893	IPI00074270	Q9H2P9	DPH5	Isoform 1 of Diphthine synthase	32	0	0.173	ND (inactive)	False	True	Inactive	–
1593	IPI00013219	Q13418	ILK	Integrin-linked protein kinase	51	0	0.054	ND (inactive)	False	False	Inactive	YES
2216	IPI00289491	O15234	CASC3	Protein CASC3	76	0	0.035	ND (inactive)	False	False	Inactive	–
1710	IPI00005578	Q9H223	EHD4	EH domain-containing protein 4	61	0	0.181	ND (inactive)	False	True	Inactive	–
1929	IPI00071180	Q9UMX0	UBQLN1	Isoform 2 of Ubiquilin-1	59	0	0.122	ND (inactive)	False	False	Inactive	–
1757	IPI00179473	Q13501	SQSTM1	Isoform 1 of Sequestosome-1	48	0	0.109	ND (inactive)	False	True	Inactive	–
2047	IPI00930066			kappa-actin	104	0	3.251	ND (inactive)	False	False	Inactive	–
1562	IPI00217272	Q86V21	AACS	Isoform 1 of Acetoacetyl-CoA synthetase	75	0	0.199	ND (inactive)	False	True	Inactive	–
2088	IPI00005184	O15164	TRIM24	Isoform Long of Transcription intermediary factor 1-alpha	117	0	0.028	ND (inactive)	False	False	Inactive	–
1865	IPI00018597	P43405	SYK	Isoform Long of Tyrosine-protein kinase SYK	72	0	0.031	ND (inactive)	False	False	Inactive	YES
1923	IPI00016480	Q9NX02	NLRP2	Isoform 1 of NACHT, LRR and PYD domains-containing protein 2	121	0	0.041	ND (inactive)	False	True	Inactive	–
1993	IPI00376199	Q7Z5L9	IRF2BP2	Isoform 1 of Interferon regulatory factor 2-binding protein 2	61	0	0.072	ND (inactive)	False	False	Inactive	–
2084	IPI00295849	P53611	RABGGTB	Geranylgeranyl transferase type-2 subunit beta	37	0	0.119	ND (inactive)	False	True	Inactive	–
1676	IPI00015609	Q9NXF7	DCAF16	DDB1- and CUL4-associated factor 16	24	0	0.492	ND (inactive)	False	True	Inactive	–
1724	IPI00220767	P25791	LMO2	Isoform 2 of Rhombotin-2	18	0	0.146	ND (inactive)	False	False	Inactive	–
1758	IPI00003848	Q9UDY4	DNAJB4	DnaJ homolog subfamily B member 4	38	0	0.387	ND (inactive)	False	True	Inactive	–
1914	IPI00020191	Q12974	PTP4A2	Isoform 1 of Protein tyrosine phosphatase type IVA 2	19	0	0.345	ND (inactive)	False	True	Inactive	–
1975	IPI00008034	Q9ULC3	RAB23	Ras-related protein Rab-23	27	0	0.123	ND (inactive)	False	False	Inactive	–
2083	IPI00015809	Q9NPF4	OSGEP	Probable O-sialoglycoprotein endopeptidase	36	0	0.123	ND (inactive)	False	True	Inactive	–
1361	IPI00157304	Q9BWW4	SSBP3	Isoform 1 of Single-stranded DNA-binding protein 3	40	0	0.180	ND (inactive)	False	True	Inactive	–
1718	IPI00061528	Q96EK9	KTI12	Protein KTI12 homolog	39	0	0.217	ND (inactive)	False	False	Inactive	–
1734	IPI00006558	Q9Y371	SH3GLB1	Isoform 1 of Endophilin-B1	41	0	0.160	ND (inactive)	False	True	Inactive	–
1780	IPI00004068	Q93074	MED12	Mediator of RNA polymerase II transcription subunit 12	247	0	0.009	ND (inactive)	False	False	Inactive	–
1927	IPI00002926	Q9H9H4	VPS37B	Vacuolar protein sorting-associated protein 37B	31	0	0.209	ND (inactive)	False	True	Inactive	–
1348	IPI00645608	Q8IU81	IRF2BP1	Interferon regulatory factor 2-binding protein 1	62	0	0.247	ND (inactive)	False	True	Inactive	–
2204	IPI00003145	P45983	MAPK8	Isoform 1 of Mitogen-activated protein kinase 8	44	0	0.138	ND (inactive)	False	False	Inactive	YES
1948	IPI00011619	O43252	PAPSS1	Bifunctional 3'-phosphoadenosine 5'-phosphosulfate synthase 1	71	0	0.101	ND (inactive)	False	True	Inactive	–
2022	IPI00217987	P11215	ITGAM	Integrin alpha-M	127	0	0.043	ND (inactive)	False	True	Inactive	YES
1630	IPI00152083	Q8TAP9	C7orf11	TTD non-photosensitive 1 protein	19	0	0.116	ND (inactive)	False	False	Inactive	–
2107	IPI00171186	Q8N8D1	PDCD7	Programmed cell death protein 7	55	0	0.048	ND (inactive)	False	False	Inactive	–
1528	IPI00015285	Q99447	PCYT2	Ethanolamine-phosphate cytidylyltransferase	44	0	0.283	ND (inactive)	False	True	Inactive	–
2095	IPI00062419	Q96GA7	SDSL	Serine dehydratase-like	35	0	0.205	ND (inactive)	False	True	Inactive	–
2038	IPI00910128	P22570	FDXR	NADPH:adrenodoxin oxidoreductase, mitochondrial	57	0	0.058	ND (inactive)	False	False	Inactive	–
1492	IPI00007047	P05109	S100A8	Protein S100-A8	11	0	0.537	ND (inactive)	False	False	Inactive	–
1709	IPI00101524	Q96H20	SNF8	Isoform 1 of Vacuolar-sorting protein SNF8	29	0	0.324	ND (inactive)	False	True	Inactive	–
1745	IPI00465123	O43299	KIAA0415	KIAA0415 gene product	165	0	0.028	ND (inactive)	False	True	Inactive	–
2072	IPI00329583	Q8NB90	SPATA5	Isoform 1 of Spermatogenesis-associated protein 5	98	0	0.096	ND (inactive)	False	True	Inactive	–
1470	IPI00005042	A0AV96	RBM47	Isoform 2 of RNA-binding protein 47	57	0	0.155	ND (inactive)	False	True	Inactive	–
2184	IPI00745792	Q9HBL8	NMRAL1	NmrA-like family protein	37	0	0.119	ND (inactive)	False	True	Inactive	–
1743	IPI00643885	Q13620	CUL4B	Cullin-4B	103	0	0.021	ND (inactive)	False	False	Inactive	–
2192	IPI00032498	Q9BTE1	DCTN5	Dynactin subunit 5	20	0	0.148	ND (inactive)	False	False	Inactive	–
1819	IPI00028083	P49770	EIF2B2	Translation initiation factor eIF-2B subunit beta	39	0	0.170	ND (inactive)	False	True	Inactive	–

Hierarchical clustering

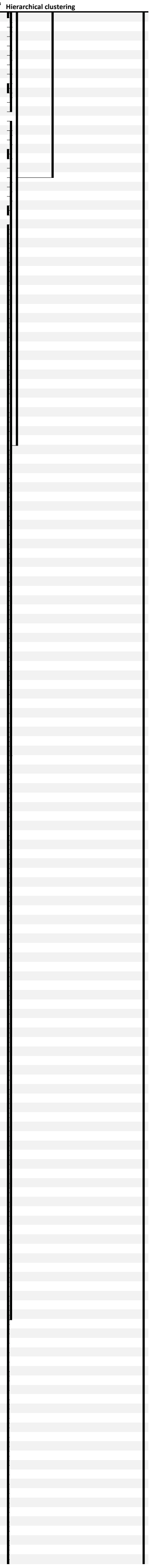

Supplementary Table 1 (cont.) | Hierarchical clustering analysis of proteins identified in active or inactive integrin adhesion complexes.

#	IPI Accession	UniProtKB Accession	Gene name	Protein name	Protein MW (kDa)	Relative abundance (mean normalised spectral count) ×10[3]		Fold enrichment (active/inactive integrin)[1]	Specific enrichment[2]		Cluster[3]	Adhesome[4]
						Active integrin	Inactive integrin		Active integrin	Inactive integrin		
2195	IPI00007304	O95376	ARIH2	Protein ariadne-2 homolog	58	0	0.076	ND (inactive)	False	True	Inactive	–
1897	IPI00152220	Q5T6S3	PHF19	Isoform 2 of PHD finger protein 19	23	0	0.228	ND (inactive)	False	True	Inactive	–
1997	IPI00930183	Q9BY27	DGCR6L	DGCR6L protein	25	0	0.233	ND (inactive)	False	True	Inactive	–
1999	IPI00024664	P45974	USP5	Isoform Long of Ubiquitin carboxyl-terminal hydrolase 5	96	0	0.029	ND (inactive)	False	False	Inactive	–
2020	IPI00013683	Q13509	TUBB3	Tubulin beta-3 chain	50	0	13.003	ND (inactive)	False	False	Inactive	–
1198	IPI00739464			Actin, gamma 1	17	0	0.325	ND (inactive)	False	False	Inactive	–
2152	IPI00607799	Q9BUT1	BDH2	Isoform 1 of 3-hydroxybutyrate dehydrogenase type 2	27	0	0.143	ND (inactive)	False	True	Inactive	–
1962	IPI00001676	Q8TAT6	NPLOC4	Isoform 2 of Nuclear protein localization protein 4 homolog	69	0	0.064	ND (inactive)	False	True	Inactive	–
1803	IPI00021264	P51911	CNN1	Calponin-1	33	0	0.269	ND (inactive)	False	True	Inactive	–
1913	IPI00783502	Q53ET0	CRTC2	CREB-regulated transcription coactivator 2	73	0	0.090	ND (inactive)	False	True	Inactive	–
2158	IPI00168554	Q9BYN0	SRXN1	Sulfiredoxin-1	14	0	0.197	ND (inactive)	False	False	Inactive	–
1432	IPI00294739	Q9Y3Z3	SAMHD1	Isoform 1 of SAM domain and HD domain-containing protein 1	72	0	0.233	ND (inactive)	False	True	Inactive	–
1953	IPI00062882	Q7Z7A3	CTU1	Cytoplasmic tRNA 2-thiolation protein 1	36	0	0.073	ND (inactive)	False	False	Inactive	–
2019	IPI00065063	Q96LJ7	DHRS1	Dehydrogenase/reductase SDR family member 1	34	0	0.162	ND (inactive)	False	True	Inactive	–
2149	IPI00298949	O14976	GAK	Cyclin-G-associated kinase	143	0	0.015	ND (inactive)	False	False	Inactive	–
1756	IPI00152089	Q8TB03	CXorf38	Isoform 1 of Uncharacterized protein CXorf38	37	0	0.149	ND (inactive)	False	True	Inactive	–
2111	IPI00025340	Q96GD0	PDXP	Pyridoxal phosphate phosphatase	32	0	0.113	ND (inactive)	False	False	Inactive	–
1942	IPI00442073	P21291	CSRP1	Cysteine and glycine-rich protein 1	21	0	0.219	ND (inactive)	False	True	Inactive	YES
2096	IPI00296370	Q9UIC8	LCMT1	Putative uncharacterized protein LCMT1	39	0	0.142	ND (inactive)	False	True	Inactive	–
2138	IPI00306871	Q92830	KAT2A	Isoform 1 of Histone acetyltransferase KAT2A	94	0	0.029	ND (inactive)	False	False	Inactive	–
152	IPI00302925	P50990	CCT8	59 kDa protein	59	2.687	1.871	1.44	False	False	Active	–
841	IPI00031521	P40938	RFC3	Replication factor C subunit 3	41	0.416	0.290	1.43	False	False	Active	–
194	IPI00017963	P62316	SNRPD2	Small nuclear ribonucleoprotein Sm D2	14	8.673	6.011	1.44	False	False	Active	–
812	IPI00465028	P60174	TPI1	triosephosphate isomerase 1 isoform 2	31	0.746	0.517	1.44	False	False	Active	–
523	IPI00328319	Q09028	RBBP4	Histone-binding protein RBBP4	48	0.938	0.650	1.44	False	False	Active	–
410	IPI00470891	O75534	CSDE1	Isoform Long of Cold shock domain-containing protein E1	89	0.636	0.441	1.44	False	False	Active	–
647	IPI00024175	O14818	PSMA7	Isoform 1 of Proteasome subunit alpha type-7	28	1.222	0.848	1.44	False	False	Active	–
238	IPI00384456	P52701	MSH6	Isoform GTBP-N of DNA mismatch repair protein Msh6	153	0.546	0.376	1.45	False	False	Active	–
253	IPI00290566	P17987	TCP1	T-complex protein 1 subunit alpha	60	1.634	1.124	1.45	False	False	Active	–
274	IPI00021263	P63104	YWHAZ	14-3-3 protein zeta/delta	28	3.096	2.135	1.45	False	False	Active	–
340	IPI00030320	P26196	DDX6	Probable ATP-dependent RNA helicase DDX6	54	1.249	0.857	1.46	False	False	Active	–
593	IPI00216691	P07737	PFN1	Profilin-1	15	2.400	1.646	1.46	False	False	Active	YES
283	IPI00376005	P63241	EIF5A	Isoform 2 of Eukaryotic translation initiation factor 5A-1	20	3.755	2.575	1.46	False	False	Active	–
671	IPI00555878	Q9NRW3	APOBEC3C	Probable DNA dC->dU-editing enzyme APOBEC-3C	23	1.180	0.809	1.46	False	False	Active	–
1191	IPI00029695	Q12824	SMARCB1	Isoform A of SWI/SNF-related matrix-associated actin-dependent regulator of chromatin subfamily B member 1	44	0.252	0.173	1.46	False	False	Active	–
1026	IPI00016249	P51114	FXR1	Isoform 1 of Fragile X mental retardation syndrome-related protein 1	70	0.124	0.084	1.48	False	False	Active	–
82	IPI00140420	Q7KZF4	SND1	Staphylococcal nuclease domain-containing protein 1	102	2.070	1.464	1.41	False	False	Active	–
526	IPI00023919	P62195	PSMC5	26S protease regulatory subunit 8	46	0.886	0.627	1.41	False	False	Active	–
294	IPI00290435	Q99661	KIF2C	Isoform 1 of Kinesin-like protein KIF2C	81	0.943	0.665	1.42	False	False	Active	–
796	IPI00290416	Q9NTK5	OLA1	Isoform 1 of Obg-like ATPase 1	45	0.534	0.377	1.42	False	False	Active	–
166	IPI00013415	P62081	RPS7	40S ribosomal protein S7	22	4.907	3.436	1.43	False	False	Active	–
374	IPI00166010	A5YKK6	CNOT1	Isoform 1 of CCR4-NOT transcription complex subunit 1	267	0.199	0.139	1.43	False	False	Active	–
744	IPI00026219	Q10570	CPSF1	Cleavage and polyadenylation specificity factor subunit 1	161	0.147	0.103	1.43	False	False	Active	–
1053	IPI00294178	P30154	PPP2R1B	Isoform 1 of Serine/threonine-protein phosphatase 2A 65 kDa regulatory subunit A beta isoform	66	0.323	0.226	1.43	False	False	Active	–
832	IPI00018398	P17980	PSMC3	26S protease regulatory subunit 6A	49	0.470	0.328	1.43	False	False	Active	–
623	IPI00005511	Q7RTV0	PHF5A	PHD finger-like domain-containing protein 5A	12	2.748	1.928	1.43	False	False	Active	–
1236	IPI00031681	P24941	CDK2	Cell division protein kinase 2	34	1.063	0.747	1.42	True	False	Active	–
64	IPI00910458	Q5T6W2	HNRNPK	Heterogeneous nuclear ribonucleoprotein K	49	5.216	3.748	1.39	False	False	Active	–
598	IPI00795769			52 kDa protein	52	0.632	0.450	1.40	False	False	Active	–
620	IPI00008552	O76003	GLRX3	Glutaredoxin-3	37	0.588	0.420	1.40	False	False	Active	–
1607	IPI00022465	O14578	CIT	Isoform 1 of Citron Rho-interacting kinase	231	0.013	0.010	1.30	False	False	Active	–
260	IPI00219097	P26583	HMGB2	High mobility group protein B2	24	3.585	2.390	1.50	False	False	Active	–
332	IPI00025273	P22102	GART	Isoform Long of Trifunctional purine biosynthetic protein adenosine-3	108	0.579	0.386	1.50	False	False	Active	–
1074	IPI00011916	Q13155	AIMP2	Aminoacyl tRNA synthetase complex-interacting multifunctional protein 2	35	0.356	0.238	1.50	False	False	Active	–
17	IPI00179330	P62979	RPS27A	ubiquitin and ribosomal protein S27a precursor	18	31.714	21.573	1.47	False	False	Active	–
319	IPI00788907	Q96HS1	PGAM5	Isoform 1 of Serine/threonine-protein phosphatase PGAM5, mitochondrial	32	1.886	1.283	1.47	False	False	Active	–
670	IPI00220835	P60468	SEC61B	Protein transport protein Sec61 subunit beta	10	3.449	2.345	1.47	False	False	Active	–
974	IPI00745613	Q9NPD3	EXOSC4	Exosome complex exonuclease RRP41	26	0.684	0.465	1.47	False	False	Active	–
89	IPI00219156	P62888	RPL30	60S ribosomal protein L30	13	16.921	11.420	1.48	False	False	Active	–
610	IPI00291093	P19388	POLR2E	DNA-directed RNA polymerases I, II, and III subunit RPABC1	25	1.558	1.051	1.48	False	False	Active	–
737	IPI00152881	Q8TF72	SHROOM3	shroom family member 3 protein	217	0.030	0.020	1.50	False	False	Active	–
331	IPI00302850	P62314	SNRPD1	Small nuclear ribonucleoprotein Sm D1	13	5.828	3.923	1.49	False	False	Active	–
517	IPI00328840	Q86V81	THOC4	THO complex 4	28	1.716	1.156	1.48	False	False	Active	–
264	IPI00184330	P49736	MCM2	DNA replication licensing factor MCM2	102	0.852	0.574	1.48	False	False	Active	–
1252	IPI00220637	P49591	SARS	Seryl-tRNA synthetase, cytoplasmic	59	0.190	0.128	1.48	False	False	Active	–
2016	IPI00845339	P08107	HSPA1A	Heat shock 70 kDa protein 1	70	2.894	1.958	1.48	False	False	Active	YES
1358	IPI00554590	Q9H2M9	RAB3GAP2	Isoform 1 of Rab3 GTPase-activating protein non-catalytic subunit	156	0.047	0.032	1.47	False	False	Active	–
673	IPI00007818	Q9UKF6	CPSF3	Cleavage and polyadenylation specificity factor subunit 3	77	0.344	0.231	1.49	False	False	Active	–
818	IPI00292975	Q9P2N5	RBM27	RNA-binding protein 27	119	0.189	0.127	1.49	False	False	Active	–
1019	IPI00289601	Q92769	HDAC2	Isoform 2 of Histone deacetylase 2	66	0.740	0.497	1.49	False	False	Active	–
25	IPI00883857	Q00839	HNRNPU	Isoform Long of Heterogeneous nuclear ribonucleoprotein U	91	5.059	3.346	1.51	False	False	Active	–
287	IPI00018465	Q99832	CCT7	T-complex protein 1 subunit eta	59	1.519	1.005	1.51	False	False	Active	–
536	IPI00029623	P60900	PSMA6	Proteasome subunit alpha type-6	27	1.520	1.004	1.51	False	False	Active	–
1040	IPI00169383	P00558	PGK1	Phosphoglycerate kinase 1	45	0.310	0.205	1.51	False	False	Active	–
185	IPI00029731	P18077	RPL35A	60S ribosomal protein L35a	13	6.697	4.413	1.52	False	False	Active	–
45	IPI00026781	P49327	FASN	Fatty acid synthase	273	1.271	0.836	1.52	False	False	Active	–
115	IPI00025491	P60842	EIF4A1	Eukaryotic initiation factor 4A-I	46	3.931	2.586	1.52	False	False	Active	–
2264	IPI00871157	P78362	SRPK2	Putative uncharacterized protein SRPK2 (Fragment)	82	0.158	0.104	1.52	False	False	Active	–
54	IPI00010740	P23246	SFPQ	Isoform Long of Splicing factor, proline- and glutamine-rich	76	3.604	2.353	1.53	False	False	Active	–
1634	IPI00017510	P00403	MT-CO2	Cytochrome c oxidase subunit 2	26	0.233	0.151	1.54	False	False	Active	–
285	IPI00182757	Q8N163	KIAA1967	Isoform 1 of Protein KIAA1967	103	0.847	0.624	1.36	False	False	Active	–
300	IPI00293434	P37108	SRP14	Signal recognition particle 14 kDa protein	15	4.954	3.638	1.36	False	False	Active	–
343	IPI00012382	P09012	SNRPA	U1 small nuclear ribonucleoprotein A	31	2.047	1.498	1.37	False	False	Active	–
63	IPI00221091	P62244	RPS15A	40S ribosomal protein S15a	15	15.069	11.019	1.37	False	False	Active	–
722	IPI00299214	P04183	TK1	Thymidine kinase, cytosolic	25	1.014	0.742	1.37	False	False	Active	–
389	IPI00220642	P61981	YWHAG	14-3-3 protein gamma	28	3.438	2.511	1.37	False	False	Active	–
48	IPI00001159	Q92616	GCN1L1	Translational activator GCN1	293	1.090	0.795	1.37	False	False	Active	–
1394	IPI00554681	Q16718	NDUFA5	NADH dehydrogenase [ubiquinone] 1 alpha subcomplex subunit 5	13	0.659	0.481	1.37	False	False	Active	–
151	IPI00009328	P38919	EIF4A3	Eukaryotic initiation factor 4A-III	47	2.827	2.060	1.37	False	False	Active	–
433	IPI00294159	P53007	SLC25A1	Tricarboxylate transport protein, mitochondrial	34	1.472	1.073	1.37	False	False	Active	–
2037	IPI00384265	Q9NZB2	FAM120A	Isoform F of Constitutive coactivator of PPAR-gamma-like protein 1	125	0.014	0.011	1.27	False	False	Active	–
108	IPI00000874	Q06830	PRDX1	Peroxiredoxin-1	22	8.130	5.899	1.38	False	False	Active	–
609	IPI00396171	P27816	MAP4	Isoform 1 of Microtubule-associated protein 4	121	0.213	0.154	1.38	False	False	Active	–
791	IPI00746351	Q9Y2L1	DIS3	Isoform 1 of Exosome complex exonuclease RRP44	109	0.207	0.151	1.37	False	False	Active	–
370	IPI00658000	O00425	IGF2BP3	Isoform 1 of Insulin-like growth factor 2 mRNA-binding protein 3	64	1.304	0.943	1.38	False	False	Active	–
930	IPI00294955	Q9Y4Z0	LSM4	U6 snRNA-associated Sm-like protein LSm4	15	0.859	0.622	1.38	False	False	Active	–
1469	IPI00871174	Q99417	MYCBP	C-Myc-binding protein	12	0.756	0.547	1.38	False	False	Active	–
552	IPI00643920	P29401	TKT	Transketolase	69	0.489	0.369	1.33	False	False	Active	–
615	IPI00012268	Q13200	PSMD2	26S proteasome non-ATPase regulatory subunit 2	100	0.305	0.229	1.33	False	False	Active	–
614	IPI00025347	Q92979	EMG1	Probable ribosome biogenesis protein NEP1	27	1.240	0.939	1.32	False	False	Active	–
811	IPI00473011	P02042	HBD	Hemoglobin subunit delta	16	5.766	4.360	1.32	False	False	Active	–
286	IPI00302927	P50991	CCT4	T-complex protein 1 subunit delta	58	1.550	1.147	1.35	False	False	Active	–
551	IPI00017381	P35249	RFC4	Replication factor C subunit 4	40	1.005	0.745	1.35	False	False	Active	–
484	IPI00005045	Q9UG63	ABCF2	ATP-binding cassette sub-family F member 2	71	0.548	0.409	1.34	False	False	Active	–
1242	IPI00746165	O75083	WDR1	Isoform 1 of WD repeat-containing protein 1	66	0.146	0.109	1.34	False	False	Active	–
1411	IPI00218848	P56385	ATP5I	ATP synthase, H+ transporting, mitochondrial F0 complex, subunit E	8	1.322	0.984	1.34	False	False	Active	–
19	IPI00844578	Q08211	DHX9	ATP-dependent RNA helicase A	141	3.798	2.408	1.58	False	False	Active	–
507	IPI00026167	P55769	NHP2L1	NHP2-like protein 1	14	3.775	2.388	1.58	False	False	Active	–
272	IPI00017448	P63220	RPS21	40S ribosomal protein S21	9	10.903	6.957	1.57	False	False	Active	–
31	IPI00003865	P11142	HSPA8	Isoform 1 of Heat shock cognate 71 kDa protein	71	6.423	4.117	1.56	False	False	Active	–
681	IPI00216319	Q04917	YWHAH	14-3-3 protein eta	28	2.194	1.406	1.56	False	False	Active	–
491	IPI00009057	Q9UN86	G3BP2	Isoform A of Ras GTPase-activating protein-binding protein 2	54	1.061	0.681	1.56	False	False	Active	–
1510	IPI00010090	P48507	GCLM	Glutamate--cysteine ligase regulatory subunit	31	0.265	0.170	1.56	False	False	Active	–
365	IPI00219160	P49207	RPL34	60S ribosomal protein L34	13	4.326	2.625	1.65	False	False	Active	–
347	IPI00291006	P40926	MDH2	Malate dehydrogenase, mitochondrial	36	2.296	1.412	1.63	False	False	Active	–
437	IPI00440493	P25705	ATP5A1	ATP synthase subunit alpha, mitochondrial	60	1.071	0.656	1.63	False	False	Active	–
743	IPI00940237	O00148	DDX39	ATP-dependent RNA helicase DDX39	49	2.524	1.550	1.63	False	False	Active	–
1498	IPI00441959	Q8N4C6	NIN	Isoform 7 of Ninein	248	0.007	0.004	1.75	False	False	Active	–
592	IPI00940685	Q96DI7	SNRNP40	U5 small nuclear ribonucleoprotein 40 kDa protein	39	0.792	0.491	1.61	False	False	Active	–
869	IPI00305374	Q96FV9	THOC1	Isoform 1 of THO complex subunit 1	76	0.234	0.145	1.61	False	False	Active	–
145	IPI00027834	P14866	HNRNPL	Heterogeneous nuclear ribonucleoprotein L	64	2.257	1.408	1.60	False	False	Active	–
361	IPI00291922	P28066	PSMA5	Proteasome subunit alpha type-5	26	2.626	1.643	1.60	False	False	Active	–
1920	IPI00023510	P20339	RAB5A	Ras-related protein Rab-5A	24	0.147	0.092	1.60	False	False	Active	–
265	IPI00456758	P46776	RPL27A	60S ribosomal protein L27a	17	5.600	3.289	1.70	False	False	Active	–
468	IPI00004860	P54136	RARS	Isoform Complexed of Arginyl-tRNA synthetase, cytoplasmic	75	0.683	0.401	1.70	False	False	Active	–
322	IPI00017964	P62318	SNRPD3	Small nuclear ribonucleoprotein Sm D3	14	5.473	3.222	1.70	False	False	Active	–
401	IPI00169325	Q8NI36	WDR36	WD repeat-containing protein 36	105	0.528	0.311	1.70	False	False	Active	–
206	IPI00215790	P63173	RPL38	60S ribosomal protein L38	8	16.156	9.545	1.69	False	False	Active	–
8	IPI00453473	P62805	HIST4H4	Histone H4	11	118.613	69.961	1.70	False	False	Active	–
1452	IPI00000728	Q9Y4E8	USP15	Isoform 1 of Ubiquitin carboxyl-terminal hydrolase 15	112	0.084	0.049	1.71	False	False	Active	–
128	IPI00438229	Q13263	TRIM28	Isoform 1 of Transcription intermediary factor 1-beta	89	1.876	1.094	1.71	False	False	Active	–
430	IPI00012011	P23528	CFL1	Cofilin-1	19	3.093	1.805	1.71	False	False	Active	YES
441	IPI00002214	P52292	KPNA2	Importin subunit alpha-2	58	1.059	0.615	1.72	False	False	Active	–
652	IPI00026665	P47897	QARS	Glutaminyl-tRNA synthetase (QARS)	90	0.367	0.213	1.72	False	False	Active	–
1181	IPI00017596	Q15691	MAPRE1	Microtubule-associated protein RP/EB family member 1	30	0.451	0.263	1.71	False	False	Active	–
125	IPI00008524	P11940	PABPC1	Isoform 1 of Polyadenylate-binding protein 1	71	2.535	1.519	1.67	False	False	Active	YES
564	IPI00444452	Q9HCE1	MOV10	Isoform 1 of Putative helicase MOV-10	114	0.361	0.216	1.67	False	False	Active	–
352	IPI00218606	P62266	RPS23	40S ribosomal protein S23	16	3.830	2.299	1.67	False	False	Active	–
1333	IPI00029485	Q14203	DCTN1	Isoform p150 of Dynactin subunit 1	142	0.054	0.033	1.64	False	False	Active	–
442	IPI00294536	Q9Y3F4	STRAP	Serine-threonine kinase receptor-associatedprotein	40	1.156	0.689	1.68	False	False	Active	–
1088	IPI00332511	P63151	PPP2R2A	Serine/threonine-protein phosphatase 2A 55 kDa regulatory subunit B alpha isoform	52	0.267	0.159	1.68	False	False	Active	–
27	IPI00217467	P10412	HIST1H1E	Histone H1.4	22	22.899	13.673	1.67	False	False	Active	–
981	IPI00018768	Q15631	TSN	Translin	26	0.692	0.413	1.68	False	False	Active	–
1687	IPI00922265	Q8N4V1	MMGT1	Isoform 1 of Membrane magnesium transporter 1	15	0.293	0.175	1.67	False	False	Active	–
814	IPI00024821	O00487	PSMD14	26S proteasome non-ATPase regulatory subunit 14	35	0.574	0.340	1.69	False	False	Active	–
200	IPI00396435	O43143	DHX15	Putative pre-mRNA-splicing factor ATP-dependent RNA helicase DHX15	91	1.340	0.797	1.68	False	False	Active	–
1436	IPI00170924	Q9NQE9	HINT3	Histidine triad nucleotide-binding protein 3	20	0.166	0.098	1.69	False	False	Active	–
318	IPI00025039	P22087	FBL	rRNA 2'-O-methyltransferase fibrillarin	34	1.896	1.066	1.78	False	False	Active	–
505	IPI00000816	P62258	YWHAE	14-3-3 protein epsilon	29	2.349	1.320	1.78	False	False	Active	–
236	IPI00026302	P62899	RPL31	60S ribosomal protein L31	14	6.635	3.727	1.78	False	False	Active	–
368	IPI00171798	O94776	MTA2	Metastasis-associated protein MTA2	75	0.836	0.470	1.78	False	False	Active	–
854	IPI00023101	Q92600	RQCD1	Cell differentiation protein RCD1 homolog	34	0.618	0.347	1.78	False	False	Active	–
942	IPI00216318	P31946	YWHAB	Isoform Long of 14-3-3 protein beta/alpha	28	2.885	1.619	1.78	False	False	Active	–
1023	IPI00790530	Q9BW27	NUP85	Nuclear pore complex protein Nup85	75	0.197	0.110	1.79	False	False	Active	–
215	IPI00012750	P62851	RPS25	40S ribosomal protein S25	14	6.743	3.754	1.80	False	False	Active	–
1043	IPI00783835	O94822	RNF160	zinc finger protein 294	205	0.068	0.038	1.79	False	False	Active	–
665	IPI00748807	Q12769	NUP160	Isoform 1 of Nuclear pore complex protein Nup160	162	0.192	0.107	1.79	False	False	Active	–
228	IPI00221089	P62277	RPS13	40S ribosomal protein S13	17	6.532	3.652	1.79	False	False	Active	–
1611	IPI00024381	Q92989	CLP1	Polyribonucleotide 5'-hydroxyl-kinase Clp1	48	0.083	0.046	1.80	False	False	Active	–
459	IPI00019329	P63167	DYNLL1	Dynein light chain 1, cytoplasmic	10	6.185	3.430	1.80	False	False	Active	–
546	IPI00470610	Q96C36	PYCR2	Pyrroline-5-carboxylate reductase 2	34	1.478	0.817	1.81	False	False	Active	–
451	IPI00027107	P49411	TUFM	Tu translation elongation factor, mitochondrial precursor	50	1.050	0.580	1.81	False	False	Active	–
1179	IPI00293817	Q99747	NAPG	Gamma-soluble NSF attachment protein	35	0.228	0.126	1.81	False	False	Active	–
112	IPI00890837	A6NHR9	SMCHD1	Isoform 1 of Structural maintenance of chromosomes flexible hinge domain-containing protein 1	226	0.736	0.405	1.82	False	False	Active	–
440	IPI00303476	P06576	ATP5B	ATP synthase subunit beta, mitochondrial	57	1.131	0.622	1.82	False	False	Active	–

Hierarchical clustering

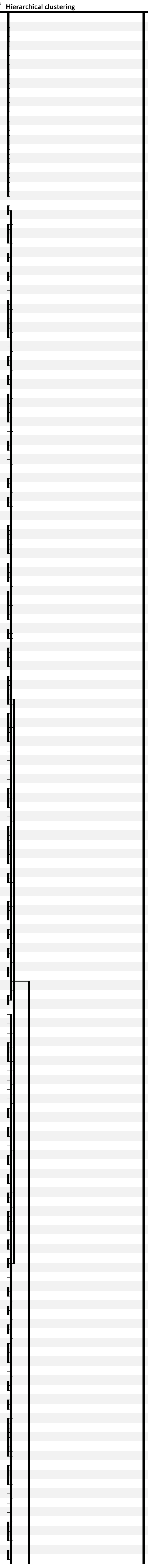

Supplementary Table 1 (cont.) | Hierarchical clustering analysis of proteins identified in active or inactive integrin adhesion complexes.

#	IPI Accession	UniProtKB Accession	Gene name	Protein name	Protein MW (kDa)	Relative abundance (mean normalised spectral count) ×10³		Fold enrichment (active/inactive integrin)[1]	Specific enrichment[2]		Cluster[3]	Adhesome[4]	Hierarchical clustering
						Active integrin	Inactive integrin		Active integrin	Inactive integrin			
240	IPI00219155	P61353	RPL27	60S ribosomal protein L27	16	5.261	2.899	1.81	False	False	Active	–	
1202	IPI00479997	P16949	STMN1	Stathmin	17	0.517	0.284	1.82	False	False	Active	–	
1659	IPI00007144	Q9UNX3	RPL26L1	60S ribosomal protein L26-like 1	17	1.653	0.910	1.82	False	False	Active	–	
65	IPI00419880	P61247	RPS3A	40S ribosomal protein S3a	30	8.250	4.500	1.83	False	False	Active	–	
493	IPI00301923	P50750	CDK9	Isoform 1 of Cell division protein kinase 9	43	1.010	0.551	1.83	False	False	Active	–	
590	IPI00023344	Q92797	SYMPK	Isoform 1 of Symplekin	141	0.242	0.132	1.83	False	False	Active	–	
385	IPI00305068	O94906	PRPF6	Pre-mRNA-processing factor 6	107	0.539	0.294	1.83	False	False	Active	–	
1090	IPI00072541	Q5JVF3	PCID2	Isoform 3 of PCI domain-containing protein 2	43	0.363	0.198	1.83	False	False	Active	–	
390	IPI00018278	Q71UI9	H2AFV	Histone H2A.V	14	34.940	19.125	1.83	False	False	Active	–	
666	IPI00292059	P49790	NUP153	Nuclear pore complex protein Nup153	154	0.182	0.100	1.82	False	False	Active	–	
1292	IPI00220741	P02549	SPTA1	Isoform 1 of Spectrin alpha chain, erythrocyte	280	0.036	0.020	1.80	False	False	Active	–	
2131	IPI00292134	P42566	EPS15	Isoform 1 of Epidermal growth factor receptor substrate 15	99	0.020	0.011	1.82	False	False	Active	–	
280	IPI00001639	Q14974	KPNB1	Importin subunit beta-1	97	0.952	0.542	1.76	False	False	Active	–	
801	IPI00030275	Q12931	TRAP1	Heat shock protein 75 kDa, mitochondrial	80	1.293	0.738	1.75	False	False	Active	–	
53	IPI00216592	P07910	HNRNPC	Isoform C1 of Heterogeneous nuclear ribonucleoproteins C1/C2	32	9.569	5.411	1.77	False	False	Active	–	
472	IPI00171199	P25788	PSMA3	Isoform 2 of Proteasome subunit alpha type-3	28	1.731	0.983	1.76	False	False	Active	–	
745	IPI00383046	Q96DG6	CMBL	Carboxymethylenebutenolidase homolog	28	0.818	0.464	1.76	False	False	Active	–	
1384	IPI00001146	P62312	LSM6	U6 snRNA-associated Sm-like protein LSm6	9	1.287	0.729	1.77	False	False	Active	–	
545	IPI00555747	Q13310	PABPC4	Isoform 2 of Polyadenylate-binding protein 4	70	1.817	1.048	1.73	False	False	Active	–	
1228	IPI00031679	Q9BQ75	C3orf26	Uncharacterized protein C3orf26	32	0.320	0.185	1.73	False	False	Active	–	
116	IPI00554723	P27635	RPL10	60S ribosomal protein L10	25	6.006	3.474	1.73	False	False	Active	–	
1842	IPI00215911	P27695	APEX1	DNA-(apurinic or apyrimidinic site) lyase	36	0.126	0.073	1.73	False	False	Active	–	
1084	IPI00550746	Q9Y266	NUDC	Nuclear migration protein nudC	38	0.362	0.207	1.75	False	False	Active	–	
1810	IPI00006092	O15305	PMM2	Phosphomannomutase 2	28	0.103	0.059	1.75	False	False	Active	–	
20	IPI00186290	P13639	EEF2	Elongation factor 2	95	6.164	2.455	2.51	False	False	Active	–	
243	IPI00644127	P41252	IARS	Isoleucyl-tRNA synthetase, cytoplasmic	145	0.725	0.289	2.51	False	False	Active	–	
333	IPI00216230	P42166	TMPO	Lamina-associated polypeptide 2, isoform alpha	75	1.147	0.458	2.50	False	False	Active	–	
323	IPI00029822	P51532	SMARCA4	SWI/SNF-related matrix-associated actin-dependent regulator of chromatin a4 isoform D	188	0.391	0.155	2.52	False	False	Active	–	
579	IPI00550882	P32322	PYCR1	Pyrroline-5-carboxylate reductase	36	1.172	0.466	2.52	False	False	Active	–	
746	IPI00003362	P11021	HSPA5	HSPA5 protein	72	0.773	0.307	2.52	False	False	Active	–	
338	IPI00465044	Q9P258	RCC2	Protein RCC2	56	1.283	0.516	2.49	False	False	Active	–	
486	IPI00297982	P41091	EIF2S3	Eukaryotic translation initiation factor 2 subunit 3	51	1.073	0.430	2.50	False	False	Active	–	
1083	IPI00299524	Q15021	NCAPD2	Condensin complex subunit 1	157	0.079	0.032	2.47	False	False	Active	–	
146	IPI00413324	P18621	RPL17	60S ribosomal protein L17	21	7.266	2.965	2.45	False	False	Active	–	
191	IPI00003377	Q16629	SFRS7	Isoform 1 of Splicing factor, arginine/serine-rich 7	27	5.887	2.400	2.45	False	False	Active	–	
606	IPI00852685	O60610	DIAPH1	Isoform 1 of Protein diaphanous homolog 1	141	0.256	0.103	2.49	False	False	Active	–	
654	IPI00299608	Q99460	PSMD1	Isoform 1 of 26S proteasome non-ATPase regulatory subunit 1	106	0.309	0.125	2.47	False	False	Active	–	
11	IPI00081836	Q96KK5	HIST1H2AG	Histone H2A type 1-H	14	68.037	27.630	2.46	False	False	Active	–	
295	IPI00010720	P48643	CCT5	T-complex protein 1 subunit epsilon	60	1.558	0.633	2.46	False	False	Active	–	
629	IPI00002520	P34897	SHMT2	Serine hydroxymethyltransferase, mitochondrial	56	0.722	0.293	2.46	False	False	Active	–	
779	IPI00026670	Q15370	TCEB2	Transcription elongation factor B polypeptide 2	13	1.562	0.632	2.47	False	False	Active	–	
1568	IPI00238688	Q6ZRY4	RBPMS2	RNA-binding protein with multiple splicing 2	22	0.147	0.060	2.45	False	False	Active	–	
180	IPI00031691	P32969	RPL9	60S ribosomal protein L9	22	6.127	2.378	2.58	False	False	Active	–	
267	IPI00216237	Q9Y3U8	RPL36	60S ribosomal protein L36	12	8.467	3.287	2.58	False	False	Active	–	
1362	IPI00250297	Q9NRN7	AASDHPPT	L-aminoadipate-semialdehyde dehydrogenase-phosphopantetheinyl transferase	36	0.282	0.109	2.59	False	False	Active	–	
202	IPI00291939	Q14683	SMC1A	Structural maintenance of chromosomes protein 1A	143	0.803	0.313	2.57	False	False	Active	–	
524	IPI00021187	Q9Y265	RUVBL1	Isoform 1 of RuvB-like 1	50	0.989	0.385	2.57	False	False	Active	–	
266	IPI00290770	P49368	CCT3	chaperonin containing TCP1, subunit 3 isoform b	60	1.760	0.686	2.57	False	False	Active	–	
757	IPI00007074	P54577	YARS	Tyrosyl-tRNA synthetase, cytoplasmic	59	0.417	0.163	2.56	False	False	Active	–	
1018	IPI00232533	P47813	EIF1AX	Eukaryotic translation initiation factor 1A, X-chromosomal	16	0.797	0.311	2.56	False	False	Active	–	
735	IPI00647650	O15372	EIF3H	Eukaryotic translation initiation factor 3, subunit 3 gamma, 40kDa, isoform CRA_b	42	0.669	0.261	2.56	False	False	Active	–	
1936	IPI00166293	Q8N257	HIST3H2BB	Histone H2B type 3-B	14	21.808	8.531	2.56	False	False	Active	–	
559	IPI00549672	Q9UNM6	PSMD13	HSPC027	43	0.988	0.389	2.54	False	False	Active	–	
575	IPI00554737	P30153	PPP2R1A	Serine/threonine-protein phosphatase 2A 65 kDa regulatory subunit A alpha isoform	65	0.668	0.264	2.53	False	False	Active	–	
586	IPI00879531			9 kDa protein	9	8.736	3.427	2.55	True	False	Active	–	
715	IPI00419919	P47914	RPL29	60S ribosomal protein L29	18	1.348	0.530	2.54	False	False	Active	–	
1371	IPI00041127	Q9NVP2	ASF1B	Histone chaperone ASF1B	22	0.128	0.050	2.56	False	False	Active	–	
42	IPI00449049	P09874	PARP1	Poly [ADP-ribose] polymerase 1	113	3.333	1.370	2.43	False	False	Active	–	
417	IPI00219994	P55060	CSE1L	Isoform 3 of Exportin-2	108	0.871	0.359	2.43	False	False	Active	–	
353	IPI00465248	P06733	ENO1	Isoform alpha-enolase of Alpha-enolase	47	1.613	0.667	2.42	False	False	Active	–	
710	IPI00013723	Q13526	PIN1	Peptidyl-prolyl cis-trans isomerase NIMA-interacting 1	18	1.741	0.719	2.42	False	False	Active	–	
489	IPI00027808	P30876	POLR2B	DNA-directed RNA polymerase II subunit RPB2	134	0.354	0.146	2.42	False	False	Active	–	
824	IPI00020042	P43686	PSMC4	Isoform 1 of 26S protease regulatory subunit 6B	47	0.526	0.217	2.42	False	False	Active	–	
6	IPI00296337	P78527	PRKDC	Isoform 1 of DNA-dependent protein kinase catalytic subunit	469	3.265	1.355	2.41	False	False	Active	–	
584	IPI00006980	Q9Y224	C14orf166	UPF0568 protein C14orf166	28	1.302	0.540	2.41	False	False	Active	–	
324	IPI00219420	Q9UQE7	SMC3	Structural maintenance of chromosomes protein 3	142	0.543	0.226	2.40	False	False	Active	–	
585	IPI00297572	O60306	AQR	Intron-binding protein aquarius	171	0.213	0.089	2.39	False	False	Active	–	
1662	IPI00306290	O43592	XPOT	Exportin-T	110	0.036	0.015	2.40	False	False	Active	–	
135	IPI00217975	P20700	LMNB1	Lamin-B1	66	2.788	1.186	2.35	False	False	Active	–	
46	IPI00015953	Q9NR30	DDX21	Isoform 1 of Nucleolar RNA helicase 2	87	4.355	1.849	2.36	False	False	Active	–	
881	IPI00815770	O60493	SNX3	Isoform 1 of Sorting nexin-3	19	1.020	0.433	2.36	False	False	Active	–	
67	IPI00299573	P62424	RPL7A	60S ribosomal protein L7a	30	8.757	3.694	2.37	False	False	Active	–	
129	IPI00299000	Q9UQ80	PA2G4	Proliferation-associated protein 2G4	44	4.443	1.878	2.37	False	False	Active	–	
1172	IPI00014310	Q13616	CUL1	Cullin-1	90	0.116	0.049	2.37	False	False	Active	–	
799	IPI00215610	Q00013	MPP1	55 kDa erythrocyte membrane protein	52	0.428	0.185	2.31	False	False	Active	–	
943	IPI00012645	O15020	SPTBN2	Isoform 1 of Spectrin beta chain, brain 2	271	0.023	0.010	2.30	False	False	Active	–	
1221	IPI00294879	P46060	RANGAP1	Ran GTPase-activating protein 1	64	0.141	0.060	2.35	False	False	Active	–	
160	IPI00247583	P46778	RPL21	60S ribosomal protein L21	19	7.413	3.235	2.29	False	False	Active	–	
959	IPI00029267	P08579	SNRPB2	U2 small nuclear ribonucleoprotein B''	25	1.535	0.670	2.29	False	False	Active	–	
667	IPI00020127	P27694	RPA1	Replication protein A 70 kDa DNA-binding subunit	68	0.441	0.191	2.31	False	False	Active	–	
1392	IPI00219839	Q96JB3	HIC2	Isoform 2 of Hypermethylated in cancer 2 protein	64	0.178	0.077	2.31	False	False	Active	–	
396	IPI00031519	P26358	DNMT1	Isoform 1 of DNA (cytosine-5)-methyltransferase 1	190	0.290	0.127	2.28	False	False	Active	–	
1429	IPI00418336	Q68E01	INTS3	Isoform 2 of Integrator complex subunit 3	118	0.064	0.028	2.29	False	False	Active	–	
1605	IPI00294402	Q9UBD5	ORC3L	Isoform 1 of Origin recognition complex subunit 3	82	0.062	0.027	2.30	False	False	Active	–	
163	IPI00297211	O60264	SMARCA5	SWI/SNF-related matrix-associated actin-dependent regulator of chromatin subfamily A member 5	122	1.185	0.421	2.81	False	False	Active	–	
763	IPI00007402	O95373	IPO7	Importin-7	120	0.232	0.083	2.80	False	False	Active	–	
109	IPI00013485	P15880	RPS2	40S ribosomal protein S2	31	5.932	2.122	2.80	False	False	Active	–	
218	IPI00295857	P53621	COPA	Isoform 1 of Coatomer subunit alpha	138	0.892	0.319	2.80	False	False	Active	–	
427	IPI00234252	Q92922	SMARCC1	SWI/SNF complex subunit SMARCC1	123	0.446	0.160	2.79	False	False	Active	–	
1204	IPI00215966	O95059	RPP14	Ribonuclease P protein subunit p14	14	0.916	0.328	2.79	False	False	Active	–	
572	IPI00029631	P84090	ERH	Enhancer of rudimentary homolog	12	3.204	1.160	2.76	False	False	Active	–	
277	IPI00470528	P61313	RPL15	60S ribosomal protein L15	24	3.811	1.371	2.78	False	False	Active	–	
980	IPI00041325	Q9NX24	NHP2	H/ACA ribonucleoprotein complex subunit 2	17	1.186	0.427	2.78	False	False	Active	–	
289	IPI00054042	P78347	GTF2I	Isoform 1 of General transcription factor II-I	112	0.764	0.280	2.73	False	False	Active	–	
850	IPI00395694	Q9Y5L0	TNPO3	Isoform 2 of Transportin-3	104	0.227	0.083	2.73	False	False	Active	–	
754	IPI00335168	P60660	MYL6	Isoform Non-muscle of Myosin light polypeptide 6	17	1.826	0.666	2.74	False	False	Active	–	
1458	IPI00007001	Q9Y3B7	MRPL11	39S ribosomal protein L11, mitochondrial	21	0.428	0.156	2.74	False	False	Active	–	
197	IPI00003519	Q15029	EFTUD2	116 kDa U5 small nuclear ribonucleoprotein component	109	1.419	0.526	2.70	False	False	Active	–	
1343	IPI00033907	Q9H1A4	ANAPC1	Anaphase-promoting complex subunit 1	217	0.048	0.018	2.67	False	False	Active	–	
845	IPI00012007	P23526	AHCY	Adenosylhomocysteinase	48	0.561	0.208	2.70	False	False	Active	–	
1164	IPI00022648	P55010	EIF5	Eukaryotic translation initiation factor 5	49	0.273	0.101	2.70	False	False	Active	–	
908	IPI00015029	Q15185	PTGES3	Prostaglandin E synthase 3	19	1.110	0.411	2.70	False	False	Active	–	
1011	IPI00168317	Q8NBT2	SPC24	Kinetochore protein Spc24	22	0.884	0.326	2.71	False	False	Active	–	
207	IPI00219365	P26038	MSN	Moesin	68	2.003	0.753	2.66	False	False	Active	YES	
692	IPI00021327	P62993	GRB2	Isoform 1 of Growth factor receptor-bound protein 2	25	1.135	0.427	2.66	False	False	Active	YES	
638	IPI00216298	P10599	TXN	Thioredoxin	12	3.025	1.134	2.67	False	False	Active	–	
602	IPI00015018	Q15181	PPA1	Inorganic pyrophosphatase	33	1.154	0.435	2.65	False	False	Active	–	
895	IPI00013679	P33316	DUT	Isoform 1 of Deoxyuridine 5'-triphosphate nucleotidohydrolase, mitochondrial	27	0.971	0.366	2.65	False	False	Active	–	
284	IPI00221222	P53999	SUB1	Activated RNA polymerase II transcriptional coactivator p15	14	6.543	2.445	2.68	False	False	Active	–	
2042	IPI00604402	Q53H96	PYCRL	Putative uncharacterized protein PYCRL	29	0.102	0.038	2.68	False	False	Active	–	
269	IPI00396370	P55884	EIF3B	Isoform 1 of Eukaryotic translation initiation factor 3 subunit B	92	1.110	0.427	2.60	False	False	Active	–	
381	IPI00017451	Q15459	SF3A1	Splicing factor 3A subunit 1	89	0.702	0.269	2.61	False	False	Active	–	
1064	IPI00011416	Q13011	ECH1	Delta(3,5)-Delta(2,4)-dienoyl-CoA isomerase, mitochondrial	36	0.666	0.255	2.61	False	False	Active	–	
952	IPI00395769	P36542	ATP5C1	Isoform Heart of ATP synthase subunit gamma, mitochondrial	33	0.674	0.259	2.60	False	False	Active	–	
1201	IPI00013184	P41227	ARD1A	N-terminal acetyltransferase complex ARD1 subunit homolog A	26	0.332	0.127	2.61	False	False	Active	–	
738	IPI00219840	P53680	AP2S1	Isoform 1 of AP-2 complex subunit sigma	17	1.427	0.544	2.62	False	False	Active	–	
1139	IPI00002557	Q9UBF2	COPG2	Coatomer subunit gamma-2	98	0.162	0.062	2.61	False	False	Active	–	
79	IPI00215884	Q07955	SFRS1	Isoform ASF-1 of Splicing factor, arginine/serine-rich 1	28	8.449	3.212	2.63	False	False	Active	–	
1749	IPI00029705	Q9BSJ2	TUBGCP2	Gamma-tubulin complex component 2	103	0.050	0.019	2.63	False	False	Active	–	
1802	IPI00011307	P13995	MTHFD2	Bifunctional methylenetetrahydrofolate dehydrogenase/cyclohydrolase, mitochondrial	38	0.091	0.035	2.60	False	False	Active	–	
117	IPI00465361	P26373	RPL13	60S ribosomal protein L13	24	7.553	4.078	1.85	False	False	Active	–	
306	IPI00004968	Q9UMS4	PRPF19	Pre-mRNA-processing factor 19	55	1.564	0.843	1.86	False	False	Active	–	
562	IPI00032003	P50402	EMD	Emerin	29	1.539	0.829	1.86	False	False	Active	–	
350	IPI00873899	Q8NE71	ABCF1	Isoform 1 of ATP-binding cassette sub-family F member 1	96	0.676	0.366	1.85	False	False	Active	–	
394	IPI00640703	Q9HAV4	XPO5	Isoform 1 of Exportin-5	136	0.454	0.243	1.87	False	False	Active	–	
1138	IPI00021347	P68036	UBE2L3	Ubiquitin-conjugating enzyme E2 L3	18	0.340	0.182	1.87	False	False	Active	–	
626	IPI00032853	Q9NPE3	NOP10	H/ACA ribonucleoprotein complex subunit 3	8	4.095	2.163	1.89	False	False	Active	–	
683	IPI00744692	P37837	TALDO1	Transaldolase	38	0.786	0.414	1.90	False	False	Active	–	
540	IPI00412344	Q96SB4	SRPK1	Isoform 2 of Serine/threonine-protein kinase SRPK1	74	0.603	0.320	1.88	False	False	Active	–	
691	IPI00150269	O43172	PRPF4	Isoform 1 of U4/U6 small nuclear ribonucleoprotein Prp4	58	0.486	0.258	1.88	False	False	Active	–	
1022	IPI00216730	Q8IUE6	HIST2H2AB	Histone H2A type 2-B	14	15.006	7.969	1.88	False	False	Active	–	
339	IPI00221106	Q13435	SF3B2	splicing factor 3B subunit 2	100	0.609	0.305	2.00	False	False	Active	–	
122	IPI00412579	P62906	RPL10A	60S ribosomal protein L10a	25	6.538	3.292	1.99	False	False	Active	–	
506	IPI00479058	P62841	RPS15	40S ribosomal protein S15	17	2.910	1.467	1.98	False	False	Active	–	
1354	IPI00465315	P99999	CYCS	Cytochrome c	12	0.816	0.412	1.98	False	False	Active	–	
141	IPI00644712	P12956	XRCC6	ATP-dependent DNA helicase 2 subunit 1	70	2.070	1.051	1.97	False	False	Active	–	
530	IPI00395627	Q9HB71	CACYBP	Isoform 1 of Calcyclin-binding protein	26	1.839	0.935	1.97	False	False	Active	–	
92	IPI00304692	P38159	RBMX	Heterogeneous nuclear ribonucleoprotein G	42	4.988	2.520	1.98	False	False	Active	–	
262	IPI00297579	Q13185	CBX3	Chromobox protein homolog 3	21	5.103	2.580	1.98	False	False	Active	–	
496	IPI00854642	Q29RF7	PDS5A	Isoform 1 of Sister chromatid cohesion protein PDS5 homolog A	151	0.274	0.139	1.97	False	False	Active	–	
1443	IPI00742943	Q8NFH3	NUP43	Nucleoporin Nup43	42	0.182	0.092	1.98	False	False	Active	–	
37	IPI00420014	O75643	SNRNP200	Isoform 1 of U5 small nuclear ribonucleoprotein 200 kDa helicase	245	1.665	0.853	1.95	False	False	Active	–	
1877	IPI00032958	Q9NQW6	ANLN	Isoform 2 of Actin-binding protein anillin	120	0.018	0.009	2.00	False	False	Active	–	
2137	IPI00290652	Q96T23	RSF1	Isoform 1 of Remodeling and spacing factor 1	164	0.013	0.007	1.86	False	False	Active	–	
399	IPI00414127	P43487	RANBP1	Ran-specific GTPase-activating protein	23	2.771	1.429	1.94	False	False	Active	–	
554	IPI00025178	O75934	BCAS2	Pre-mRNA-splicing factor SPF27	26	1.255	0.648	1.94	False	False	Active	–	
233	IPI00410693	Q8NC51	SERBP1	Isoform 1 of Plasminogen activator inhibitor 1 RNA-binding protein	45	2.444	1.264	1.93	False	False	Active	–	
341	IPI00009104	Q9Y230	RUVBL2	RuvB-like 2	51	1.672	0.865	1.93	False	False	Active	–	
413	IPI00477971	Q12788	TBL3	Transducin beta-like protein 3	89	0.598	0.310	1.93	False	False	Active	–	
1276	IPI00011126	P62191	PSMC1	26S protease regulatory subunit 4	49	0.403	0.209	1.93	False	False	Active	–	
603	IPI00000001	O95793	STAU1	Isoform Long of Double-stranded RNA-binding protein Staufen homolog 1	63	0.647	0.339	1.91	False	False	Active	–	
694	IPI00005675	O15226	NKRF	NF-kappa-B-repressing factor	78	0.241	0.126	1.91	False	False	Active	–	
822	IPI00015911	P09622	DLD	Dihydrolipoyl dehydrogenase, mitochondrial	54	0.582	0.304	1.91	False	False	Active	–	
1660	IPI00026054	P49137	MAPKAPK2	Isoform 1 of MAP kinase-activated protein kinase 2	46	0.055	0.029	1.90	False	False	Active	–	
2061	IPI00017855	Q99798	ACO2	Aconitate hydratase, mitochondrial	85	0.030	0.015	2.00	False	False	Active	–	
268	IPI00152785	P23527	HIST1H2BO	Histone H2B type 1-O	14	74.446	36.617	2.03	False	False	Active	–	
438	IPI00026625	O75694	NUP155	Isoform 1 of Nuclear pore complex protein Nup155	155	0.352	0.173	2.03	False	False	Active	–	
70	IPI00000494	P46777	RPL5	60S ribosomal protein L5	34	7.459	3.691	2.02	False	False	Active	–	
190	IPI00182533	P46779	RPL28	60S ribosomal protein L28	16	6.329	3.132	2.02	False	False	Active	–	
354	IPI00219678	P05198	EIF2S1	Eukaryotic translation initiation factor 2 subunit 1	36	2.148	1.064	2.02	False	False	Active	–	
86	IPI00216587	P62241	RPS8	40S ribosomal protein S8	24	10.429	5.147	2.03	False	False	Active	–	
608	IPI00938339			hypothetical protein XP_002343863	15	1.152	0.569	2.02	False	False	Active	–	
537	IPI00220344	P83881	RPL36A	60S ribosomal protein L36a	12	2.214	1.094	2.02	False	False	Active	–	
1211	IPI00027705	P49643	PRIM2	Isoform 1 of DNA primase large subunit	59	0.171	0.084	2.04	False	False	Active	–	
95	IPI00010204	P84103	SFRS3	Splicing factor, arginine/serine-rich 3	19	10.299	5.136	2.01	False	False	Active	–	

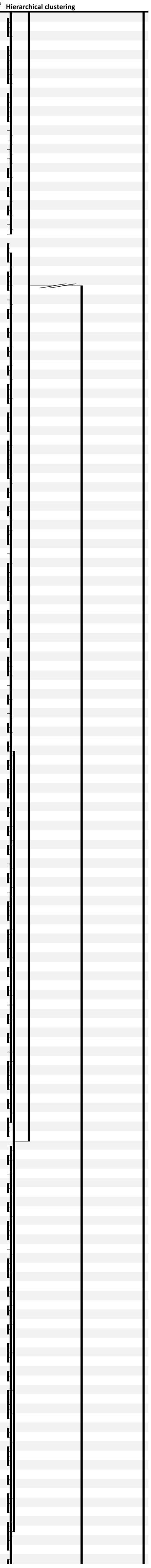

Supplementary Table 1 (cont.) | Hierarchical clustering analysis of proteins identified in active or inactive integrin adhesion complexes.

#	IPI Accession	UniProtKB Accession	Gene name	Protein name	Protein MW (kDa)	Relative abundance (mean normalised spectral count) ×10[3]: Active integrin	Inactive integrin	Fold enrichment (active/inactive integrin)[1]	Specific enrichment[2]: Active integrin	Inactive integrin	Cluster[3]	Adhesome[4]	Hierarchical clustering
371	IPI00009960	Q16891	IMMT	Isoform 1 of Mitochondrial inner membrane protein	84	0.774	0.386	2.01	False	False	Active	–	
55	IPI00217030	P62701	RPS4X	40S ribosomal protein S4, X isoform	30	9.643	4.785	2.02	False	False	Active	–	
9	IPI00303133	Q93079	HIST1H2BH	Histone H2B type 1-H	14	77.372	38.470	2.01	False	False	Active	–	
455	IPI00163187	Q16658	FSCN1	Fascin	55	0.914	0.454	2.01	False	False	Active	–	
1565	IPI00027704	P49642	PRIM1	DNA primase small subunit	50	0.079	0.039	2.03	False	False	Active	–	
94	IPI00017297	P43243	MATR3	Matrin-3	95	2.016	0.979	2.06	False	False	Active	–	
556	IPI00427330	Q9Y3A5	SBDS	Ribosome maturation protein SBDS	29	1.463	0.712	2.05	False	False	Active	–	
677	IPI00031522	P40939	HADHA	Trifunctional enzyme subunit alpha, mitochondrial	83	0.415	0.201	2.06	False	False	Active	–	
501	IPI00170692	Q9P0L0	VAPA	Isoform 1 of Vesicle-associated membrane protein-associated protein A	28	1.780	0.862	2.06	False	False	Active	–	
1430	IPI00003824	Q13356	PPIL2	Isoform 1 of Peptidyl-prolyl cis-trans isomerase-like 2	59	0.116	0.056	2.07	False	False	Active	–	
183	IPI00012772	P62917	RPL8	60S ribosomal protein L8	28	4.513	2.203	2.05	False	False	Active	–	
655	IPI00220528	P62306	SNRPF	Small nuclear ribonucleoprotein F	10	4.132	2.017	2.05	False	False	Active	–	
619	IPI00019912	P51659	HSD17B4	Peroxisomal multifunctional enzyme type 2	80	0.482	0.236	2.04	False	False	Active	–	
1171	IPI00029081	P49916	LIG3	Isoform Alpha of DNA ligase 3	113	0.084	0.041	2.05	False	False	Active	–	
303	IPI00021700	P12004	PCNA	Proliferating cell nuclear antigen	29	2.652	1.274	2.08	False	False	Active	–	
179	IPI00013917	P25398	RPS12	40S ribosomal protein S12	15	9.778	4.702	2.08	True	False	Active	–	
612	IPI00182289	P62273	RPS29	40S ribosomal protein S29	7	5.071	2.438	2.08	False	False	Active	–	
132	IPI00848161	Q13838	BAT1	Isoform 1 of Spliceosome RNA helicase BAT1	49	3.088	1.480	2.09	False	False	Active	–	
1009	IPI00006052	Q9UHV9	PFDN2	Prefoldin subunit 2	17	1.237	0.593	2.09	False	False	Active	–	
1046	IPI00024145	P45880	VDAC2	Isoform 2 of Voltage-dependent anion-selective channel protein 2	30	0.497	0.238	2.09	False	False	Active	–	
990	IPI00384624	O14660	GART	GARS-AIRS-GART (Fragment)	11	1.145	0.552	2.07	False	False	Active	–	
1134	IPI00163084	Q9HCS7	XAB2	Pre-mRNA-splicing factor SYF1	100	0.135	0.065	2.08	False	False	Active	–	
1094	IPI00007163	Q9UK45	LSM7	U6 snRNA-associated Sm-like protein LSm7	12	1.020	0.492	2.07	False	False	Active	–	
227	IPI00013808	O43707	ACTN4	Alpha-actinin-4	105	1.176	0.539	2.18	False	False	Active	YES	
213	IPI00012345	Q13247	SFRS6	Isoform SRP55-1 of Splicing factor, arginine/serine-rich 6	40	2.658	1.217	2.18	False	False	Active	–	
474	IPI00016613	P68400	CSNK2A1	CSNK2A1 protein	46	1.124	0.515	2.18	False	False	Active	–	
373	IPI00397904	Q8N1F7	NUP93	Nuclear pore complex protein Nup93	93	0.715	0.329	2.17	False	False	Active	–	
1192	IPI00032879	Q9Y3D8	AK6	Adenylate kinase isoenzyme 6	20	0.623	0.287	2.17	False	False	Active	–	
1073	IPI00012369	Q13257	MAD2L1	Mitotic spindle assembly checkpoint protein MAD2A	24	0.537	0.247	2.17	False	False	Active	–	
162	IPI00027270	P61254	RPL26	60S ribosomal protein L26	17	7.171	3.254	2.20	False	False	Active	–	
226	IPI00022774	P55072	VCP	Transitional endoplasmic reticulum ATPase	89	1.301	0.591	2.20	False	False	Active	–	
1260	IPI00413895	Q08379	GOLGA2	Golgi autoantigen, golgin subfamily a, 2	113	0.086	0.039	2.21	False	False	Active	–	
1496	IPI00031698	Q9BVC5	C2orf49	Ashwin	26	0.252	0.114	2.21	False	False	Active	–	
1747	IPI00219381	O43678	NDUFA2	NADH dehydrogenase [ubiquinone] 1 alpha subcomplex subunit 2	11	0.393	0.179	2.20	False	False	Active	–	
580	IPI00032827	Q9Y3B4	SF3B14	Pre-mRNA branch site protein p14	15	2.761	1.243	2.22	False	False	Active	–	
230	IPI00297779	P78371	CCT2	T-complex protein 1 subunit beta	57	2.099	0.948	2.21	False	False	Active	–	
1240	IPI00011528	Q05048	CSTF1	Cleavage stimulation factor subunit 1	48	0.182	0.082	2.22	False	False	Active	–	
495	IPI00654777	O00303	EIF3F	HCG1784554, isoform CRA_a	39	1.405	0.631	2.23	False	False	Active	–	
355	IPI00007765	P38646	HSPA9	Stress-70 protein, mitochondrial	74	1.157	0.519	2.23	False	False	Active	–	
1773	IPI00032872	Q9Y3D3	MRPS16	28S ribosomal protein S16, mitochondrial	15	0.293	0.131	2.24	False	False	Active	–	
1032	IPI00029728	P18074	ERCC2	TFIIH basal transcription factor complex helicase subunit	87	0.128	0.057	2.25	False	False	Active	–	
275	IPI00783781	Q92621	NUP205	Nuclear pore complex protein Nup205	228	0.399	0.188	2.12	False	False	Active	–	
886	IPI00337307	Q8IZ69	TRMT2A	Isoform 1 of tRNA (uracil-5-)-methyltransferase homolog A (Fragment)	69	0.280	0.132	2.12	False	False	Active	–	
797	IPI00073602	Q5RKV6	EXOSC6	Exosome complex exonuclease MTR3	28	1.016	0.479	2.12	False	False	Active	–	
938	IPI00328306	O75152	ZC3H11A	Zinc finger CCCH domain-containing protein 11A	89	0.105	0.050	2.10	False	False	Active	–	
976	IPI00010200	Q9H6S0	YTHDC2	Probable ATP-dependent RNA helicase YTHDC2	160	0.083	0.039	2.13	False	False	Active	–	
896	IPI00008922	Q01629	IFITM2	Interferon-induced transmembrane protein 2	15	1.646	0.783	2.10	False	False	Active	–	
522	IPI00642971	P29692	EEF1D	Isoform 2 of Elongation factor 1-delta	71	0.716	0.339	2.11	False	False	Active	–	
571	IPI00010105	P56537	EIF6	Eukaryotic translation initiation factor 6	27	1.667	0.789	2.11	False	False	Active	–	
1571	IPI00022334	P04181	OAT	Ornithine aminotransferase, mitochondrial	49	0.113	0.054	2.09	False	False	Active	–	
1254	IPI00003925	P11177	PDHB	Isoform 1 of Pyruvate dehydrogenase E1 component subunit beta, mitochondrial	39	0.321	0.152	2.11	False	False	Active	–	
549	IPI00010368	O00139	KIF2A	Isoform 1 of Kinesin-like protein KIF2A	78	0.742	0.348	2.13	False	False	Active	–	
334	IPI00419258	P09429	HMGB1	High mobility group protein B1	25	4.580	2.144	2.14	False	False	Active	–	
1922	IPI00292671	O95985	TOP3B	Isoform 1 of DNA topoisomerase 3-beta-1	97	0.029	0.014	2.07	False	False	Active	–	
739	IPI00033143	Q9UBQ5	EIF3K	Eukaryotic translation initiation factor 3 subunit K	25	1.135	0.526	2.16	False	False	Active	–	
1130	IPI00005179	O15160	POLR1C	Isoform 1 of DNA-directed RNA polymerases I and III subunit RPAC1	39	0.329	0.152	2.16	False	False	Active	–	
1474	IPI00024742	O14949	UQCRQ	Cytochrome b-c1 complex subunit 8	10	0.705	0.328	2.15	False	False	Active	–	
51	IPI00549248	P06748	NPM1	Isoform 1 of Nucleophosmin	33	9.010	2.875	3.13	False	False	Active	–	
103	IPI00783271	P42704	LRPPRC	Leucine-rich PPR motif-containing protein, mitochondrial	158	1.547	0.494	3.13	True	False	Active	–	
83	IPI00221088	P46781	RPS9	40S ribosomal protein S9	23	9.659	3.080	3.14	True	False	Active	–	
307	IPI00011569	Q13085	ACACA	Isoform 1 of Acetyl-CoA carboxylase 1	266	0.311	0.100	3.11	False	False	Active	–	
1341	IPI00009659	Q9NQG5	RPRD1B	Regulation of nuclear pre-mRNA domain-containing protein 1B	37	0.387	0.124	3.12	True	False	Active	–	
1534	IPI00017669	Q969G3	SMARCE1	Isoform 1 of SWI/SNF-related matrix-associated actin-dependent regulator of chromatin subfamily E member 1	47	0.175	0.056	3.13	False	False	Active	–	
184	IPI00456969	Q14204	DYNC1H1	Cytoplasmic dynein 1 heavy chain 1	532	0.284	0.092	3.09	False	False	Active	–	
786	IPI00384992	P12829	MYL4	Myosin light chain 4	22	1.329	0.429	3.10	True	False	Active	–	
977	IPI00022793	P55084	HADHB	Trifunctional enzyme subunit beta, mitochondrial	51	0.460	0.148	3.11	True	False	Active	–	
205	IPI00021266	P62750	RPL23A	60S ribosomal protein L23a	18	6.857	2.237	3.07	False	False	Active	–	
707	IPI00300078	Q15269	PWP2	Periodic tryptophan protein 2 homolog	102	0.294	0.096	3.06	False	False	Active	–	
403	IPI00006932	Q9Y383	LUC7L2	RNA-binding protein Luc7-like 2	54	1.068	0.347	3.08	True	False	Active	–	
1694	IPI00216085	P14854	COX6B1	Cytochrome c oxidase subunit 6B1	10	0.403	0.131	3.08	False	False	Active	–	
40	IPI00218753	P11388	TOP2A	Isoform 3 of DNA topoisomerase 2-alpha	179	2.188	0.688	3.18	False	False	Active	–	
102	IPI00019502	P35579	MYH9	Isoform 1 of Myosin-9	227	1.251	0.393	3.18	True	False	Active	YES	
24	IPI00604620	P19338	NCL	Nucleolin	77	7.219	2.275	3.17	False	False	Active	–	
662	IPI00157790	Q5VYK3	ECM29	KIAA0368 protein	224	0.137	0.043	3.19	False	False	Active	–	
724	IPI00293845	Q5UIP0	RIF1	Isoform 1 of Telomere-associated protein RIF1	274	0.093	0.029	3.21	False	False	Active	–	
309	IPI00395998	P62910	RPL32	60S ribosomal protein L32	16	5.496	1.712	3.21	True	False	Active	–	
392	IPI00022228	Q00341	HDLBP	Vigilin	141	0.476	0.148	3.22	True	False	Active	–	
836	IPI00024364	Q92973	TNPO1	Isoform 1 of Transportin-1	102	0.270	0.084	3.21	False	False	Active	–	
1372	IPI00397376	Q6PJT7	ZC3H14	Isoform 1 of Zinc finger CCCH domain-containing protein 14	83	0.076	0.024	3.17	False	False	Active	–	
600	IPI00031836	Q9Y295	DRG1	Developmentally-regulated GTP-binding protein 1	41	1.070	0.326	3.28	True	False	Active	–	
1263	IPI00797373	Q8NF50	DOCK8	Isoform 1 of Dedicator of cytokinesis protein 8	239	0.041	0.012	3.42	False	False	Active	–	
813	IPI00218319	P06753	TPM3	Isoform 2 of Tropomyosin alpha-3 chain	29	0.824	0.254	3.24	False	False	Active	–	
932	IPI00329633	P26639	TARS	Threonyl-tRNA synthetase, cytoplasmic	83	0.309	0.095	3.25	False	False	Active	–	
599	IPI00025418	Q02388	COL7A1	Isoform 1 of Collagen alpha-1(VII) chain	295	0.029	0.009	3.22	False	False	Active	–	
1598	IPI00334579	Q8N983	MRPL43	Mitochondrial ribosomal protein L43 (MRPL43), transcript variant 4	29	0.295	0.091	3.24	False	False	Active	–	
33	IPI00024067	Q00610	CLTC	Isoform 1 of Clathrin heavy chain 1	192	2.947	0.979	3.01	False	False	Active	–	
169	IPI00009342	P46940	IQGAP1	Ras GTPase-activating-like protein IQGAP1	189	0.800	0.265	3.02	False	False	Active	–	
362	IPI00411733	Q7L2E3	DHX30	Isoform 1 of Putative ATP-dependent RNA helicase DHX30	134	0.585	0.194	3.02	False	False	Active	–	
182	IPI00793696	P83731	RPL24	19 kDa protein	19	8.198	2.748	2.98	False	False	Active	–	
290	IPI00298961	O14980	XPO1	Exportin-1	123	0.766	0.255	3.00	False	False	Active	–	
349	IPI00013068	P60228	EIF3E	Eukaryotic translation initiation factor 3 subunit E	52	1.496	0.491	3.05	False	False	Active	–	
397	IPI00291783	Q8TEQ6	GEMIN5	Gem-associated protein 5	169	0.375	0.124	3.02	False	False	Active	–	
617	IPI00306518	Q9NVI1	FANCI	Isoform 3 of Fanconi anemia group I protein	149	0.225	0.074	3.04	False	False	Active	–	
1578	IPI00443474	Q6ZR21	FKBP5	51 kDa FK506-binding protein	51	0.117	0.039	3.00	False	False	Active	–	
642	IPI00760588	P55265	ADAR	Isoform 5 of Double-stranded RNA-specific adenosine deaminase	104	0.310	0.107	2.90	False	False	Active	–	
713	IPI00024524	Q9NRX1	PNO1	RNA-binding protein PNO1	28	0.882	0.302	2.92	False	False	Active	–	
1304	IPI00298547	Q99497	PARK7	Protein DJ-1	20	0.576	0.197	2.92	False	False	Active	–	
214	IPI00291755	Q8TEM1	NUP210	Isoform 1 of Nuclear pore membrane glycoprotein 210	205	0.611	0.206	2.97	False	False	Active	–	
513	IPI00414860	P61513	RPL37A	60S ribosomal protein L37a	10	5.033	1.706	2.95	False	False	Active	–	
820	IPI00005861	O43395	PRPF3	Isoform 1 of U4/U6 small nuclear ribonucleoprotein Prp3	78	0.340	0.115	2.96	True	False	Active	–	
487	IPI00009032	P05455	SSB	Lupus La protein	47	1.192	0.403	2.96	False	False	Active	–	
1691	IPI00456635	Q70J99	UNC13D	Isoform 3 of Protein unc-13 homolog D	129	0.038	0.013	2.92	False	False	Active	–	
1095	IPI00015972	P09669	COX6C	Cytochrome c oxidase subunit 6C	9	1.287	0.438	2.94	False	False	Active	–	
1808	IPI00154283	Q8TCG1	KIAA1524	Isoform 1 of Protein CIP2A	102	0.032	0.011	2.91	False	False	Active	–	
81	IPI00021405	P02545	LMNA	Isoform A of Lamin-A/C	74	3.445	1.214	2.84	False	False	Active	–	
819	IPI00028004	P49720	PSMB3	Proteasome subunit beta type-3	23	1.227	0.434	2.83	False	False	Active	–	
384	IPI00000015	Q08170	SFRS4	Splicing factor, arginine/serine-rich 4	57	0.499	0.174	2.87	False	False	Active	–	
386	IPI00020928	Q00059	TFAM	Transcription factor A, mitochondrial	29	2.404	0.842	2.86	False	False	Active	–	
1473	IPI00056494	Q969Q0	RPL36AL	60S ribosomal protein L36a-like	12	2.340	0.820	2.85	False	False	Active	–	
446	IPI00297477	P09661	SNRPA1	U2 small nuclear ribonucleoprotein A'	28	2.050	0.710	2.89	False	False	Active	–	
250	IPI00305282	Q92878	RAD50	Isoform 1 of DNA repair protein RAD50	154	0.593	0.206	2.88	False	False	Active	–	
674	IPI00029764	Q12874	SF3A3	Splicing factor 3A subunit 3	59	0.482	0.167	2.89	False	False	Active	–	
1163	IPI00386189	Q9BXJ9	NARG1	Isoform 1 of NMDA receptor-regulated protein 1	101	0.135	0.047	2.87	False	False	Active	–	
257	IPI00295992	Q9NVI7	ATAD3A	Isoform 2 of ATPase family AAA domain-containing protein 3A	66	1.543	0.533	2.89	False	False	Active	–	
760	IPI00022305	Q9Y6E2	BZW2	Basic leucine zipper and W2 domain-containing protein 2	48	0.633	0.219	2.89	True	False	Active	–	
865	IPI00103247	Q8WVV9	HNRPLL	Isoform 1 of Heterogeneous nuclear ribonucleoprotein L-like	60	0.329	0.113	2.91	False	False	Active	–	
1610	IPI00893485	Q8N201	INTS1	Putative uncharacterized protein INTS1	245	0.026	0.009	2.89	False	False	Active	–	
98	IPI00215719	Q07020	RPL18	60S ribosomal protein L18	22	10.011	3.000	3.34	False	False	Active	–	
138	IPI00221325	P49792	RANBP2	E3 SUMO-protein ligase RanBP2	358	0.488	0.146	3.34	True	False	Active	–	
229	IPI00220301	P30041	PRDX6	Peroxiredoxin-6	25	4.676	1.399	3.34	False	False	Active	–	
514	IPI00031554	Q9BQ39	DDX50	ATP-dependent RNA helicase DDX50	83	0.620	0.185	3.35	True	False	Active	–	
193	IPI00028275	Q14008	CKAP5	Isoform 1 of Cytoskeleton-associated protein 5	226	0.627	0.186	3.37	True	False	Active	–	
120	IPI00300371	Q15393	SF3B3	Isoform 1 of Splicing factor 3B subunit 3	136	1.415	0.420	3.37	True	False	Active	–	
730	IPI00218782	P47756	CAPZB	F-actin capping protein subunit beta	37	0.837	0.248	3.38	True	False	Active	–	
983	IPI00019996	Q9NWH9	SLTM	modulator of estrogen induced transcription isoform b	115	0.103	0.031	3.32	False	False	Active	–	
91	IPI00167941	Q9NU22	MDN1	Midasin	633	0.377	0.111	3.40	True	False	Active	–	
59	IPI00397526	P35580	MYH10	Isoform 1 of Myosin-10	229	1.375	0.406	3.39	True	False	Active	–	
1241	IPI00744598	Q9Y5S4	POLE	DNA polymerase	263	0.036	0.011	3.27	False	False	Active	–	
542	IPI00303207	P61221	ABCE1	ATP-binding cassette sub-family E member 1	67	0.669	0.196	3.41	True	False	Active	–	
855	IPI00020602	P19784	CSNK2A2	Casein kinase II subunit alpha'	41	0.764	0.224	3.41	True	False	Active	–	
421	IPI00300074	Q9NSD9	FARSB	Phenylalanyl-tRNA synthetase beta chain	66	0.954	0.277	3.44	True	False	Active	–	
1357	IPI00735181	Q9H3U1	UNC45A	Isoform 2 of Protein unc-45 homolog A	102	0.093	0.027	3.44	False	False	Active	–	
174	IPI00021840	P62753	RPS6	40S ribosomal protein S6	29	5.311	1.535	3.46	True	False	Active	–	
1367	IPI00033561	Q15287	RNPS1	Isoform 1 of RNA-binding protein with serine-rich domain 1	34	0.170	0.049	3.47	False	False	Active	–	
1685	IPI00239077	P49773	HINT1	Histidine triad nucleotide-binding protein 1	14	0.411	0.118	3.48	False	False	Active	–	
363	IPI00106491	Q9UKD2	MRTO4	mRNA turnover protein 4 homolog	28	2.768	0.778	3.56	True	False	Active	–	
416	IPI00396321	Q96AG4	LRRC59	Leucine-rich repeat-containing protein 59	35	1.879	0.527	3.57	True	False	Active	–	
473	IPI00915022	Q8NI27	THOC2	Isoform 1 of THO complex subunit 2	183	0.310	0.087	3.56	True	False	Active	–	
726	IPI00218918	P04083	ANXA1	Annexin A1	39	0.899	0.252	3.57	False	False	Active	–	
330	IPI00178352	Q14315	FLNC	Isoform 1 of Filamin-C	291	0.430	0.121	3.55	True	False	Active	YES*	
1416	IPI00171844	Q9BT78	COPS4	COP9 signalosome complex subunit 4	46	0.229	0.065	3.52	False	False	Active	–	
725	IPI00007334	Q9UKV3	ACIN1	Isoform 1 of Apoptotic chromatin condensation inducer in the nucleus	152	0.205	0.059	3.47	True	False	Active	–	
834	IPI00005969	P52907	CAPZA1	F-actin-capping protein subunit alpha-1	33	0.805	0.229	3.52	True	False	Active	–	
985	IPI00218775	Q13451	FKBP5	Peptidyl-prolyl cis-trans isomerase FKBP5	51	0.361	0.103	3.50	False	False	Active	–	
271	IPI00290204	P08621	SNRNP70	Isoform 1 of U1 small nuclear ribonucleoprotein 70 kDa	52	1.496	0.424	3.53	True	False	Active	–	
1629	IPI00018288	P19387	POLR2C	DNA-directed RNA polymerase II subunit RPB3	31	0.188	0.053	3.55	False	False	Active	–	
101	IPI00000846	Q14839	CHD4	Isoform 1 of Chromodomain-helicase-DNA-binding protein 4	218	0.943	0.264	3.57	True	False	Active	–	
187	IPI00402183	O60506	SYNCRIP	Isoform 3 of Heterogeneous nuclear ribonucleoprotein Q	63	2.444	0.684	3.57	True	False	Active	–	
1078	IPI00004902	P38117	ETFB	Isoform 1 of Electron transfer flavoprotein subunit beta	28	0.728	0.204	3.57	True	False	Active	–	
1261	IPI00004655	Q14331	FRG1	Protein FRG1	29	0.566	0.158	3.58	True	False	Active	–	
950	IPI00003420	Q15555	MAPRE2	Isoform 1 of Microtubule-associated protein RP/EB family member 2	37	0.564	0.158	3.57	True	False	Active	–	
777	IPI00183400	P48729	CSNK1A1	Isoform 1 of Casein kinase I isoform alpha	39	0.798	0.222	3.59	True	False	Active	–	
1205	IPI00014149	Q15006	TTC35	Tetratricopeptide repeat protein 35	35	0.403	0.113	3.57	False	False	Active	–	
276	IPI00059366	O75367	H2AFY	Isoform 3 of Core histone macro-H2A.1	39	2.613	0.725	3.60	True	False	Active	–	
1096	IPI00024620	Q9NPA8	ENY2	Enhancer of yellow 2 transcription factor homolog	12	1.182	0.328	3.60	False	False	Active	–	
1079	IPI00644482	Q969U7	PSMG2	Proteasome assembly chaperone 2	29	0.492	0.136	3.62	True	False	Active	–	
131	IPI00418313	Q12906	ILF3	interleukin enhancer binding factor 3 isoform d	96	1.898	0.507	3.74	True	False	Active	–	
164	IPI00555744	P50914	RPL14	Ribosomal protein L14 variant	24	6.413	1.715	3.74	True	False	Active	–	
465	IPI00647217	P42285	SKIV2L2	Superkiller viralicidic activity 2-like 2	118	0.442	0.120	3.68	True	False	Active	–	
424	IPI00642816	P49458	SRP9	Signal recognition particle 9 kDa protein	10	6.091	1.647	3.70	True	False	Active	–	
634	IPI00069084	Q9Y4A5	TRRAP	Isoform 1 of Transformation/transcription domain-associated protein	438	0.079	0.021	3.76	True	False	Active	–	
225	IPI00005198	Q12905	ILF2	Interleukin enhancer-binding factor 2	43	3.206	0.873	3.67	True	False	Active	–	
689	IPI00295851	P53618	COPB1	Coatomer subunit beta	107	0.285	0.077	3.70	True	False	Active	–	
58	IPI00302592	P21333	FLNA	Isoform 2 of Filamin-A	280	1.265	0.346	3.66	True	False	Active	YES	
581	IPI00217468	P16401	HIST1H1B	Histone H1.5	23	3.131	0.856	3.66	True	False	Active	–	

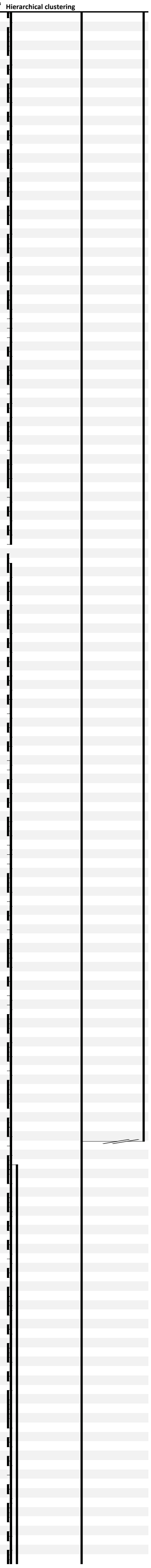

Supplementary Table 1 (cont.) | Hierarchical clustering analysis of proteins identified in active or inactive integrin adhesion complexes.

#	IPI Accession	UniProtKB Accession	Gene name	Protein name	Protein MW (kDa)	Relative abundance (mean normalised spectral count) ×10³: Active integrin	Relative abundance (mean normalised spectral count) ×10³: Inactive integrin	Fold enrichment (active/inactive integrin)[1]	Specific enrichment[2]: Active integrin	Specific enrichment[2]: Inactive integrin	Cluster[3]	Adhesome[4]	Hierarchical clustering
1136	IPI00013297	Q13442	PDAP1	28 kDa heat- and acid-stable phosphoprotein	21	0.686	0.188	3.65	False	False	Active	–	
621	IPI00025277	O75340	PDCD6	Programmed cell death protein 6	22	1.899	0.518	3.67	True	False	Active	–	
867	IPI00178431	P46063	RECQL	ATP-dependent DNA helicase Q1	73	0.248	0.068	3.65	False	False	Active	–	
173	IPI00300127	Q9H0A0	NAT10	N-acetyltransferase 10	116	1.321	0.362	3.65	False	False	Active	–	
1050	IPI00003964	Q93008	USP9X	ubiquitin specific protease 9, X-linked isoform 4	290	0.042	0.011	3.82	True	False	Active	–	
1103	IPI00031960	O95602	POLR1A	DNA-directed RNA polymerase I subunit RPA1	195	0.062	0.017	3.65	False	False	Active	–	
1189	IPI00306301	P08559	PDHA1	Mitochondrial PDHA1	48	0.399	0.109	3.66	False	False	Active	–	
1355	IPI00909083	Q96GF2	GSPT1	G1 to S phase transition 1 isoform 2	69	0.058	0.016	3.63	False	False	Active	–	
78	IPI00550021	P39023	RPL3	60S ribosomal protein L3	46	5.318	1.393	3.82	True	False	Active	–	
199	IPI00024279	Q9H583	HEATR1	HEAT repeat-containing protein 1	242	0.529	0.138	3.83	False	False	Active	–	
1175	IPI00219037	P16104	H2AFX	Histone H2A.x	15	19.260	5.031	3.83	False	False	Active	–	
1420	IPI00029133	P24539	ATP5F1	ATP synthase subunit b, mitochondrial	29	0.261	0.068	3.84	False	False	Active	–	
317	IPI00400922	Q14690	PDCD11	Protein RRP5 homolog	209	0.374	0.099	3.78	False	False	Active	–	
475	IPI00026215	P39748	FEN1	Flap endonuclease 1	43	1.273	0.336	3.79	True	False	Active	–	
577	IPI00009771	Q03252	LMNB2	Lamin-B2	70	0.860	0.227	3.79	True	False	Active	–	
720	IPI00032460	Q9Y333	LSM2	U6 snRNA-associated Sm-like protein LSm2	11	2.939	0.776	3.79	True	False	Active	–	
1537	IPI00003856	P36543	ATP6V1E1	V-type proton ATPase subunit E 1	26	0.285	0.076	3.75	False	False	Active	–	
1589	IPI00005904	Q9UHI6	DDX20	Probable ATP-dependent RNA helicase DDX20	92	0.081	0.021	3.86	False	False	Active	–	
93	IPI00742682	P12270	TPR	Nucleoprotein TPR	267	0.920	0.228	4.04	True	False	Active	–	
1045	IPI00018206	P00505	GOT2	Aspartate aminotransferase, mitochondrial	47	0.505	0.126	4.01	False	False	Active	–	
75	IPI00006196	Q14980	NUMA1	Isoform 2 of Nuclear mitotic apparatus protein 1	237	1.088	0.274	3.97	True	False	Active	–	
312	IPI00005978	Q01130	SFRS2	Splicing factor, arginine/serine-rich 2	25	2.915	0.735	3.97	True	False	Active	–	
935	IPI00939707	O75153	KIAA0664	Protein KIAA0664	147	0.154	0.039	3.95	False	False	Active	–	
685	IPI00009071	O75494	SFRS13A	Isoform 3 of Splicing factor, arginine/serine-rich 13A	22	1.303	0.328	3.97	True	False	Active	–	
1544	IPI00013895	P31949	S100A11	Protein S100-A11	12	0.648	0.164	3.95	False	False	Active	–	
686	IPI00024919	P30048	PRDX3	Thioredoxin-dependent peroxide reductase, mitochondrial	28	1.216	0.305	3.99	True	False	Active	–	
931	IPI00293276	P14174	MIF	Macrophage migration inhibitory factor	12	1.962	0.492	3.99	False	False	Active	–	
702	IPI00292000	Q8WWY3	PRPF31	Isoform 1 of U4/U6 small nuclear ribonucleoprotein Prp31	55	0.602	0.155	3.88	True	False	Active	–	
450	IPI00008982	P54886	ALDH18A1	Isoform Long of Delta-1-pyrroline-5-carboxylate synthetase	87	0.796	0.204	3.90	True	False	Active	–	
1595	IPI00619921	Q9BTC0	DIDO1	Isoform 4 of Death-inducer obliterator 1	244	0.018	0.005	3.60	False	False	Active	–	
1116	IPI00785096	Q7L1Q6	BZW1	Isoform 1 of Basic leucine zipper and W2 domain-containing protein 1	48	0.480	0.123	3.90	True	False	Active	–	
281	IPI00029750	P62847	RPS24	Isoform 1 of 40S ribosomal protein S24	15	7.308	1.669	4.38	True	False	Active	–	
158	IPI00026089	O75533	SF3B1	Splicing factor 3B subunit 1	146	1.187	0.273	4.35	True	False	Active	–	
439	IPI00006725	Q9BUQ8	DDX23	Probable ATP-dependent RNA helicase DDX23	96	0.636	0.146	4.36	True	False	Active	–	
123	IPI00029012	Q14152	EIF3A	Eukaryotic translation initiation factor 3 subunit A	167	1.181	0.277	4.26	True	False	Active	–	
497	IPI00007927	O95347	SMC2	Isoform 1 of Structural maintenance of chromosomes protein 2	136	0.390	0.091	4.29	True	False	Active	–	
97	IPI00871827	P18124	RPL7	Putative uncharacterized protein RPL7 (Fragment)	32	7.436	1.739	4.28	True	False	Active	–	
994	IPI00176642	Q96GD4	AURKB	Serine/threonine-protein kinase 12	39	0.458	0.107	4.28	False	False	Active	–	
336	IPI00411559	Q9NTJ3	SMC4	Isoform 1 of Structural maintenance of chromosomes protein 4	147	0.497	0.115	4.32	True	False	Active	–	
398	IPI00843975	P15311	EZR	Ezrin	69	0.946	0.219	4.32	True	False	Active	YES	
512	IPI00472160	Q92974	ARHGEF2	Rho/rac guanine nucleotide exchange factor 2	121	0.408	0.094	4.34	True	False	Active	YES	
519	IPI00027285	P14678	SNRPB	Isoform SM-B' of Small nuclear ribonucleoprotein-associated proteins B and B'	25	1.850	0.430	4.30	True	False	Active	–	
1148	IPI00217413	Q7Z478	DHX29	ATP-dependent RNA helicase DHX29	155	0.073	0.017	4.29	True	False	Active	–	
1025	IPI00301058	P50552	VASP	Vasodilator-stimulated phosphoprotein	40	0.495	0.115	4.30	True	False	Active	YES	
400	IPI00411937	O00567	NOP56	Nucleolar protein 56	66	1.016	0.242	4.20	False	False	Active	–	
748	IPI00216047	Q8TAQ2	SMARCC2	Isoform 1 of SWI/SNF complex subunit SMARCC2	133	0.331	0.079	4.19	True	False	Active	–	
764	IPI00300990	Q9Y3Y2	C1orf77	Isoform 1 of Uncharacterized protein C1orf77	26	0.983	0.232	4.24	True	False	Active	–	
454	IPI00220362	P61604	HSPE1	10 kDa heat shock protein, mitochondrial	11	6.303	1.523	4.14	True	False	Active	–	
534	IPI00021290	P53396	ACLY	ATP-citrate synthase	121	0.378	0.091	4.15	True	False	Active	–	
1440	IPI00477468	Q6PD62	CTR9	RNA polymerase-associated protein CTR9 homolog	134	0.034	0.008	4.25	False	False	Active	–	
1684	IPI00329719	O94832	MYO1D	Myosin-Id	116	0.047	0.011	4.27	False	False	Active	–	
1061	IPI00170935	Q8N1G4	LRRC47	Leucine-rich repeat-containing protein 47	63	0.280	0.068	4.12	True	False	Active	–	
1222	IPI00032831	O95721	SNAP29	Synaptosomal-associated protein 29	29	0.392	0.095	4.13	False	False	Active	–	
1173	IPI00303158	Q8NFW8	CMAS	Isoform 1 of N-acylneuraminate cytidylyltransferase	48	0.302	0.073	4.14	False	False	Active	–	
35	IPI00007928	Q6P2Q9	PRPF8	Pre-mRNA-processing-splicing factor 8	274	1.642	0.336	4.89	True	False	Active	–	
846	IPI00032849	Q9Y3C1	NOP16	Nucleolar protein 16	21	1.070	0.219	4.89	True	False	Active	–	
597	IPI00009922	Q9GZT3	SLIRP	SRA stem-loop-interacting RNA-binding protein, mitochondrial	12	3.486	0.711	4.90	True	False	Active	–	
436	IPI00163505	Q14498	RBM39	Isoform 1 of RNA-binding protein 39	59	1.122	0.228	4.92	True	False	Active	–	
1182	IPI00219673	Q9Y2Q3	GSTK1	Glutathione S-transferase kappa 1	25	0.645	0.131	4.92	True	False	Active	–	
741	IPI00019927	P51665	PSMD7	26S proteasome non-ATPase regulatory subunit 7	37	0.907	0.184	4.93	True	False	Active	–	
961	IPI00007208	Q9UJV9	DDX41	Probable ATP-dependent RNA helicase DDX41	70	0.234	0.047	4.98	False	False	Active	–	
769	IPI00554742	Q9BZZ5	API5	Isoform 2 of Apoptosis inhibitor 5	57	0.499	0.104	4.80	True	False	Active	–	
837	IPI00021728	P20042	EIF2S2	Eukaryotic translation initiation factor 2 subunit 2	38	0.669	0.138	4.85	True	False	Active	–	
596	IPI00456887	Q1KMD3	HNRNPUL2	Heterogeneous nuclear ribonucleoprotein U-like protein 2	85	0.402	0.085	4.73	True	False	Active	–	
804	IPI00304187	Q9NW13	RBM28	RNA-binding protein 28	86	0.260	0.055	4.73	False	False	Active	–	
708	IPI00419844	Q53GS9	USP39	U4/U6.U5 tri-snRNP-associated protein 2	65	0.517	0.110	4.70	True	False	Active	–	
1129	IPI00017256	Q15404	RSU1	Ras suppressor protein 1	32	0.360	0.076	4.74	True	False	Active	–	
348	IPI00412607	P42766	RPL35	60S ribosomal protein L35	15	5.266	1.177	4.47	True	False	Active	–	
502	IPI00011603	O43242	PSMD3	26S proteasome non-ATPase regulatory subunit 3	61	0.956	0.212	4.51	True	False	Active	–	
955	IPI00719622	P62857	RPS28	40S ribosomal protein S28	8	2.952	0.656	4.50	False	False	Active	–	
1210	IPI00009407	P61803	DAD1	Dolichyl-diphosphooligosaccharide--protein glycosyltransferase subunit DAD1	12	1.482	0.328	4.52	True	False	Active	–	
1307	IPI00187011	Q9UPT8	ZC3H4	Zinc finger CCCH domain-containing protein 4	140	0.089	0.020	4.45	True	False	Active	–	
960	IPI00021417	O43290	SART1	U4/U6.U5 tri-snRNP-associated protein 1	90	0.258	0.056	4.61	True	False	Active	–	
335	IPI00012074	O43390	HNRNPR	Isoform 1 of Heterogeneous nuclear ribonucleoprotein R	71	2.387	0.515	4.63	True	False	Active	–	
883	IPI00748303	Q96KR1	ZFR	Putative uncharacterized protein ZFR	115	0.203	0.044	4.61	True	False	Active	–	
1778	IPI00101987	Q9NWV8	MERIT40	Isoform 1 of BRCA1-A complex subunit MERIT40	37	0.138	0.030	4.60	False	False	Active	–	
876	IPI00070943	P42356	PI4KA	Isoform 1 of Phosphatidylinositol 4-kinase alpha	231	0.094	0.020	4.70	True	False	Active	–	
1335	IPI00012970	O00743	PPP6C	Isoform 1 of Serine/threonine-protein phosphatase 6 catalytic subunit	35	0.144	0.032	4.50	True	False	Active	–	
1439	IPI00643317	O15347	HMGB3	21 kDa protein	21	0.429	0.094	4.56	True	False	Active	–	
1262	IPI00169413	P82930	MRPS34	28S ribosomal protein S34, mitochondrial	26	0.573	0.126	4.55	True	False	Active	–	
423	IPI00339379	Q92888	ARHGEF1	Isoform 2 of Rho guanine nucleotide exchange factor 1	99	0.105	0.020	5.25	False	False	Active	–	
544	IPI00465294	Q99459	CDC5L	Cell division cycle 5-like protein	92	0.528	0.099	5.33	True	False	Active	–	
235	IPI00256684	O95782	AP2A1	Isoform B of AP-2 complex subunit alpha-1	105	1.369	0.258	5.31	True	False	Active	–	
924	IPI00013830	Q13573	SNW1	SNW domain-containing protein 1	61	0.286	0.054	5.30	True	False	Active	–	
1638	IPI00396279	Q7Z460	CLASP1	Isoform 1 of CLIP-associating protein 1	169	0.041	0.008	5.13	False	False	Active	–	
569	IPI00418169	P07355	ANXA2	Isoform 2 of Annexin A2	40	1.231	0.230	5.35	True	False	Active	–	
1373	IPI00005160	O15143	ARPC1B	Actin-related protein 2/3 complex subunit 1B	41	0.256	0.048	5.33	False	False	Active	YES*	
302	IPI00413611	P11387	TOP1	DNA topoisomerase 1	91	0.920	0.175	5.26	True	False	Active	–	
1281	IPI00791573	Q9Y2Z0	SUGT1	Isoform 2 of Suppressor of G2 allele of SKP1 homolog	38	0.273	0.052	5.25	False	False	Active	–	
831	IPI00744711	Q8TCS8	PNPT1	Polyribonucleotide nucleotidyltransferase 1, mitochondrial	86	0.239	0.046	5.20	True	False	Active	–	
988	IPI00514053	P48444	ARCN1	Coatomer subunit delta	57	0.361	0.069	5.23	False	False	Active	–	
1106	IPI00883647	Q13123	IK	RED protein	66	0.208	0.040	5.20	False	False	Active	–	
1174	IPI00025086	P20674	COX5A	Cytochrome c oxidase subunit 5A, mitochondrial	17	1.419	0.270	5.26	False	False	Active	–	
29	IPI00298994	Q9Y490	TLN1	Talin-1	270	2.487	0.487	5.11	True	False	Active	YES	
565	IPI00293464	Q16531	DDB1	DNA damage-binding protein 1	127	0.331	0.064	5.17	True	False	Active	–	
829	IPI00291200	Q8WUM0	NUP133	Nuclear pore complex protein Nup133	129	0.206	0.040	5.15	True	False	Active	–	
1340	IPI00440689	Q9BY77	POLDIP3	Isoform 2 of Polymerase delta-interacting protein 3	43	0.313	0.061	5.13	False	False	Active	–	
140	IPI00014898	Q15149	PLEC1	Isoform 1 of Plectin-1	532	0.338	0.065	5.20	True	False	Active	YES	
778	IPI00002624	O43660	PLRG1	Isoform 1 of Pleiotropic regulator 1	57	0.536	0.104	5.15	True	False	Active	–	
821	IPI00001091	Q9Y4W6	AFG3L2	AFG3-like protein 2	89	0.294	0.057	5.16	True	False	Active	–	
1320	IPI00855998	P49454	CENPF	Centromere protein F	368	0.028	0.005	5.60	False	False	Active	–	
653	IPI00465132	O14579	COPE	Coatomer subunit epsilon	34	0.949	0.188	5.05	True	False	Active	–	
1280	IPI00790937	Q96D46	NMD3	60S ribosomal export protein NMD3	58	0.143	0.029	4.93	True	False	Active	–	
1551	IPI00291419	Q9BWD1	ACAT2	Acetyl-CoA acetyltransferase, cytosolic	45	0.146	0.029	5.03	False	False	Active	–	
1609	IPI00295066	Q96GC5	MRPL48	39S ribosomal protein L48, mitochondrial	24	0.273	0.055	4.96	False	False	Active	–	
1644	IPI00032416	Q9Y219	JAG2	Isoform Long of Protein jagged-2	133	0.049	0.010	4.90	False	False	Active	–	
664	IPI00030243	P61289	PSME3	Isoform 1 of Proteasome activator complex subunit 3	30	1.284	0.227	5.66	True	False	Active	–	
701	IPI00031556	P26368	U2AF2	Isoform 1 of Splicing factor U2AF 65 kDa subunit	54	0.619	0.109	5.68	True	False	Active	–	
947	IPI00220833	P17480	UBTF	Isoform UBF2 of Nucleolar transcription factor 1	85	0.131	0.023	5.70	True	False	Active	–	
1377	IPI00011631	O43264	ZW10	Centromere/kinetochore protein zw10 homolog	89	0.105	0.019	5.53	True	False	Active	–	
838	IPI00009464	Q01780	EXOSC10	Isoform 1 of Exosome component 10	101	0.222	0.038	5.84	True	False	Active	–	
1002	IPI00292499	Q0VDF9	HSPA14	Heat shock 70 kDa protein 14	55	0.403	0.070	5.76	True	False	Active	–	
1334	IPI00334159	P61758	VBP1	Von Hippel-Lindau binding protein 1	27	0.235	0.041	5.73	False	False	Active	–	
1376	IPI00003438	O75937	DNAJC8	DnaJ homolog subfamily C member 8	30	0.377	0.066	5.71	False	False	Active	–	
351	IPI00025329	P84098	RPL19	60S ribosomal protein L19	23	3.063	0.563	5.44	True	False	Active	–	
1324	IPI00001655	Q9Y6A4	C16orf80	UPF0468 protein C16orf80	23	0.470	0.086	5.47	True	False	Active	–	
1405	IPI00010427	Q9BV81	TMEM93	Transmembrane protein 93	12	1.200	0.219	5.48	False	False	Active	–	
488	IPI00104050	Q9Y2W1	THRAP3	Thyroid hormone receptor-associated protein 3	109	0.367	0.066	5.56	False	False	Active	–	
875	IPI00783982	Q9Y678	COPG	Coatomer subunit gamma	98	0.275	0.049	5.61	True	False	Active	–	
1209	IPI00018931	Q96QK1	VPS35	Vacuolar protein sorting-associated protein 35	92	0.167	0.030	5.57	False	False	Active	–	
1323	IPI00185533	Q96EE3	SEH1L	Isoform A of Nucleoporin SEH1	40	0.275	0.049	5.61	True	False	Active	–	
1455	IPI00017763	Q99733	NAP1L4	Putative uncharacterized protein NAP1L4	44	0.281	0.050	5.62	True	False	Active	–	
1338	IPI00019450	P10155	TROVE2	Isoform Long of 60 kDa SS-A/Ro ribonucleoprotein	61	0.201	0.036	5.58	False	False	Active	–	
1454	IPI00953593	P55196	MLLT4	Putative uncharacterized protein MLLT4	198	0.046	0.008	5.75	True	False	Active	–	
383	IPI00021812	Q09666	AHNAK	Neuroblast differentiation-associated protein AHNAK	629	0.120	0.007	17.14	True	False	Active	–	
789	IPI00867509	Q9ULV4	CORO1C	Coronin-1C_i3 protein	59	0.574	0.033	17.39	True	False	Active	YES*	
464	IPI00301503	P62995	TRA2B	Isoform 1 of Transformer-2 protein homolog beta	34	1.530	0.090	17.00	True	False	Active	–	
828	IPI00305833	Q2TAY7	SMU1	WD40 repeat-containing protein SMU1	58	0.325	0.019	17.11	True	False	Active	–	
668	IPI00640981	Q5T4S7	UBR4	Isoform 4 of E3 ubiquitin-protein ligase UBR4	574	0.064	0.003	21.33	True	False	Active	–	
891	IPI00333010	Q8IWX8	CHERP	Calcium homeostasis endoplasmic reticulum protein	104	0.193	0.011	17.55	True	False	Active	–	
962	IPI00550069	P13489	RNH1	Ribonuclease inhibitor	50	0.587	0.033	17.79	True	False	Active	–	
360	IPI00299254	O60841	EIF5B	Eukaryotic translation initiation factor 5B	139	0.545	0.036	15.14	True	False	Active	–	
448	IPI00900293	O75369	FLNB	filamin B isoform 1	282	0.326	0.022	14.82	True	False	Active	YES*	
839	IPI00021924	Q92522	H1FX	Histone H1x	22	1.316	0.089	14.79	True	False	Active	–	
1014	IPI00477686	P13984	GTF2F2	General transcription factor IIF subunit 2	28	0.692	0.047	14.72	True	False	Active	–	
518	IPI00022143	Q9BSJ8	ESYT1	Isoform 1 of Extended synaptotagmin-1	123	0.518	0.032	16.19	True	False	Active	–	
1121	IPI00410330	Q86YP4	GATAD2A	Isoform 1 of Transcriptional repressor p66-alpha	68	0.314	0.019	16.53	True	False	Active	–	
462	IPI00005154	Q08945	SSRP1	FACT complex subunit SSRP1	81	0.664	0.041	16.20	True	False	Active	–	
927	IPI00106567	Q9C0J8	WDR33	WD repeat-containing protein 33	146	0.148	0.009	16.44	True	False	Active	–	
860	IPI00154975	Q8WXX5	DNAJC9	DnaJ homolog subfamily C member 9	30	0.905	0.055	16.45	True	False	Active	–	
1035	IPI00215743	Q9P2E9	RRBP1	Isoform 3 of Ribosome-binding protein 1	152	0.119	0.007	17.00	True	False	Active	–	
1142	IPI00334291	P36776	LONP1	Lon protease homolog	103	0.210	0.013	16.15	True	False	Active	–	
972	IPI00019488	Q9NV31	IMP3	U3 small nucleolar ribonucleoprotein protein IMP3	22	0.937	0.060	15.62	True	False	Active	–	
1065	IPI00165393	Q9BTT0	ANP32E	Acidic leucine-rich nuclear phosphoprotein 32 family member E	31	0.676	0.042	16.10	True	False	Active	–	
150	IPI00003918	P36578	RPL4	60S ribosomal protein L4	48	4.192	0.306	13.70	True	False	Active	–	
656	IPI00006181	O15371	EIF3D	Eukaryotic translation initiation factor 3 subunit D	64	0.660	0.048	13.75	True	False	Active	–	
453	IPI00008708	O76021	RSL1D1	Ribosomal L1 domain-containing protein 1	55	1.223	0.088	13.90	True	False	Active	–	
127	IPI00329389	Q02878	RPL6	60S ribosomal protein L6	33	5.688	0.435	13.08	True	False	Active	–	
919	IPI00010471	P13796	LCP1	Plastin-2	70	0.368	0.028	13.14	True	False	Active	–	
765	IPI00185374	O00232	PSMD12	26S proteasome non-ATPase regulatory subunit 12	53	0.550	0.042	13.10	True	False	Active	–	
951	IPI00015905	Q13868	EXOSC2	Exosome complex exonuclease RRP4	33	0.786	0.060	13.10	True	False	Active	–	
853	IPI00925702	B7Z2Z1	SAFB	Scaffold attachment factor B	91	0.566	0.042	13.48	True	False	Active	–	
995	IPI00300341	Q15369	TCEB1	Transcription elongation factor B polypeptide 1	12	1.230	0.092	13.37	True	False	Active	–	
910	IPI00929427	O14497	ARID1A	Isoform 3 of AT-rich interactive domain-containing protein 1A	206	0.085	0.006	14.17	True	False	Active	–	
964	IPI00297261	P18031	PTPN1	Tyrosine-protein phosphatase non-receptor type 1	50	0.439	0.033	13.30	True	False	Active	YES	
909	IPI00026466	Q6KC79	NIPBL	Isoform 2 of Nipped-B-like protein	304	0.094	0.007	13.43	True	False	Active	–	
922	IPI00033494	O14950	MYL12B	Myosin regulatory light chain 12B	20	1.268	0.098	12.94	True	False	Active	–	
870	IPI00020194	Q92804	TAF15	Isoform Short of TATA-binding protein-associated factor 2N	62	0.377	0.032	11.78	True	False	Active	–	
805	IPI00298058	O00267	SUPT5H	Isoform 1 of Transcription elongation factor SPT5	121	0.195	0.016	12.19	True	False	Active	–	
1107	IPI00021338	P10515	DLAT	Dihydrolipoyllysine-residue acetyltransferase component of pyruvate dehydrogenase complex, mitochondrial	69	0.230	0.019	12.11	True	False	Active	–	
806	IPI00072377	Q01105	SET	Isoform 1 of Protein SET	33	0.727	0.060	12.12	True	False	Active	–	
1184	IPI00218495	P29372	MPG	Isoform 2 of DNA-3-methyladenine glycosylase	32	0.500	0.041	12.20	True	False	Active	–	
1077	IPI00021129	O00203	AP3B1	Isoform 1 of AP-3 complex subunit beta-1	121	0.114	0.009	12.67	True	False	Active	–	
460	IPI00465233	Q9Y262	EIF3L	Eukaryotic translation initiation factor 3, subunit E interacting protein	71	1.013	0.090	11.26	True	False	Active	–	
968	IPI00178150	O95239	KIF4A	Isoform 1 of Chromosome-associated kinesin KIF4A	140	0.129	0.012	10.75	True	False	Active	–	

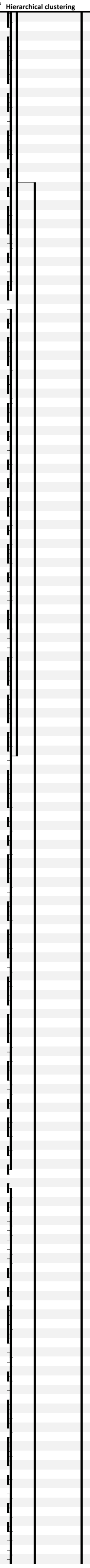

Supplementary Table 1 (cont.) | Hierarchical clustering analysis of proteins identified in active or inactive integrin adhesion complexes.

#	IPI Accession	UniProtKB Accession	Gene name	Protein name	Protein MW (kDa)	Relative abundance (mean normalised spectral count) ×10[3] Active integrin	Relative abundance (mean normalised spectral count) ×10[3] Inactive integrin	Fold enrichment (active/inactive integrin)[1]	Specific enrichment[2] Active integrin	Specific enrichment[2] Inactive integrin	Cluster[3]	Adhesome[4]	Hierarchical clustering
1223	IPI00102425	Q96P16	RPRD1A	Isoform 1 of Regulation of nuclear pre-mRNA domain-containing protein 1A	36	0.340	0.031	10.97	True	False	Active	–	
1295	IPI00005979	Q9UI10	EIF2B4	Isoform 1 of Translation initiation factor eIF-2B subunit delta	58	0.261	0.023	11.35	True	False	Active	–	
1297	IPI00006298	Q13427	PPIG	Isoform 1 of Peptidyl-prolyl cis-trans isomerase G	89	0.172	0.015	11.47	True	False	Active	–	
509	IPI00221394	O60832	DKC1	H/ACA ribonucleoprotein complex subunit 4	58	0.923	0.096	9.61	True	False	Active	–	
595	IPI00152890	Q9H6R4	NOL6	Isoform 1 of Nucleolar protein 6	128	0.314	0.033	9.52	True	False	Active	–	
978	IPI00302281	Q9NY93	DDX56	Probable ATP-dependent RNA helicase DDX56	62	0.307	0.032	9.59	True	False	Active	–	
986	IPI00240812	Q9NTI5	PDS5B	165 kDa protein	165	0.078	0.008	9.75	True	False	Active	–	
247	IPI00026970	Q9Y5B9	SUPT16H	FACT complex subunit SPT16	120	0.831	0.081	10.26	True	False	Active	–	
573	IPI00069817	Q9UIG0	BAZ1B	Isoform 1 of Tyrosine-protein kinase BAZ1B	171	0.248	0.024	10.33	True	False	Active	–	
1135	IPI00012079	P23588	EIF4B	Eukaryotic translation initiation factor 4B	69	0.193	0.019	10.16	True	False	Active	–	
856	IPI00396329	Q9H7B2	BXDC1	Ribosome production factor 2 homolog	36	0.556	0.055	10.11	True	False	Active	–	
1036	IPI00219229	P62310	LSM3	U6 snRNA-associated Sm-like protein LSm3	12	1.662	0.164	10.13	True	False	Active	–	
525	IPI00385042	Q9BZE4	GTPBP4	Nucleolar GTP-binding protein 1	74	0.711	0.071	10.01	True	False	Active	–	
1015	IPI00021435	P35998	PSMC2	26S protease regulatory subunit 7	49	0.453	0.045	10.07	True	False	Active	–	
1336	IPI00182180	Q8N6M0	OTUD6B	OTU domain containing 6B	37	0.300	0.030	10.00	True	False	Active	–	
1378	IPI00012837	P33176	KIF5B	Kinesin-1 heavy chain	110	0.120	0.012	10.00	True	False	Active	–	
688	IPI00024662	P45973	CBX5	Chromobox protein homolog 5	22	1.748	0.165	10.59	True	False	Active	–	
788	IPI00216697	P16157	ANK1	Isoform Er1 of Ankyrin-1	206	0.143	0.013	11.00	True	False	Active	–	
996	IPI00006252	Q12904	AIMP1	Aminoacyl tRNA synthetase complex-interacting multifunctional protein 1	34	0.623	0.058	10.74	True	False	Active	–	
601	IPI00745955	Q99848	EBNA1BP2	Probable rRNA-processing protein EBP2	35	1.171	0.113	10.36	True	False	Active	–	
1052	IPI00003627	O96019	ACTL6A	Isoform 1 of Actin-like protein 6A	47	0.437	0.042	10.40	True	False	Active	–	
1066	IPI00000733	Q9Y5J1	UTP18	U3 small nucleolar RNA-associated protein 18 homolog	64	0.267	0.026	10.27	True	False	Active	–	
1258	IPI00644570			18 kDa protein	18	0.964	0.092	10.48	True	False	Active	–	
1329	IPI00013122	Q16543	CDC37	Hsp90 co-chaperone Cdc37	44	0.314	0.030	10.47	True	False	Active	–	
329	IPI00016801	P00367	GLUD1	Glutamate dehydrogenase 1, mitochondrial	61	1.675	0.181	9.25	True	False	Active	–	
1185	IPI00158020	Q8IX01	SFRS14	Isoform 1 of Putative splicing factor, arginine/serine-rich 14	120	0.101	0.011	9.18	True	False	Active	–	
96	IPI00004233	P46013	MKI67	Isoform Long of Antigen KI-67	359	0.625	0.068	9.19	True	False	Active	–	
695	IPI00328753	Q86UP2	KTN1	Isoform 1 of Kinectin	156	0.214	0.023	9.30	True	False	Active	YES	
944	IPI00030131	P42167	TMPO	Isoform Beta of Lamina-associated polypeptide 2, isoforms beta/gamma	51	1.545	0.167	9.25	True	False	Active	–	
1401	IPI00030702	P50213	IDH3A	Isoform 1 of Isocitrate dehydrogenase [NAD] subunit alpha, mitochondrial	40	0.302	0.033	9.15	True	False	Active	–	
1005	IPI00797126	Q13765	NACA	nascent polypeptide-associated complex alpha subunit isoform a	95	0.258	0.028	9.21	True	False	Active	–	
553	IPI00005648	Q14151	SAFB2	Scaffold attachment factor B2	107	0.434	0.046	9.43	True	False	Active	–	
1012	IPI00064328	O14744	PRMT5	protein arginine methyltransferase 5 isoform b	71	0.261	0.028	9.32	True	False	Active	–	
1013	IPI00064328	O14744	PRMT5	protein arginine methyltransferase 5 isoform b	71	0.261	0.028	9.32	True	False	Active	–	
1337	IPI00218993	Q92598	HSPH1	Isoform Beta of Heat shock protein 105 kDa	92	0.113	0.012	9.42	False	False	Active	–	
1519	IPI00018120	P51398	DAP3	28S ribosomal protein S29, mitochondrial	46	0.268	0.029	9.24	True	False	Active	–	
679	IPI00003768	O00541	PES1	Isoform 1 of Pescadillo homolog	68	0.525	0.058	9.05	True	False	Active	–	
946	IPI00032533	Q9BV38	WDR18	WD repeat-containing protein 18	47	0.507	0.056	9.05	True	False	Active	–	
1063	IPI00334190	Q9UJZ1	STOML2	Stomatin-like protein 2	39	0.609	0.067	9.09	True	False	Active	–	
984	IPI00102752	Q96T37	RBM15	Isoform 1 of Putative RNA-binding protein 15	107	0.168	0.018	9.33	True	False	Active	–	
868	IPI00550766	Q96J73	RRP1	Ribosomal RNA processing protein 1 homolog A	53	0.504	0.056	9.00	True	False	Active	–	
1082	IPI00376481	Q7Z7K6	CENPV	Isoform 3 of Centromere protein V	30	0.590	0.066	8.94	True	False	Active	–	
758	IPI00000643	O95816	BAG2	BAG family molecular chaperone regulator 2	24	1.269	0.151	8.40	True	False	Active	–	
1001	IPI00430472	Q8N3C0	ASCC3	Activating signal cointegrator 1 complex subunit 3	251	0.066	0.008	8.25	True	False	Active	–	
124	IPI00027280	Q02880	TOP2B	Isoform Beta-2 of DNA topoisomerase 2-beta	183	0.966	0.116	8.33	True	False	Active	–	
587	IPI00216699	Q86UX7	FERMT3	Isoform 2 of Fermitin family homolog 3	75	0.730	0.088	8.30	True	False	Active	YES	
1344	IPI00030911	Q9BV40	VAMP8	Vesicle-associated membrane protein 8	11	1.492	0.179	8.34	True	False	Active	–	
1051	IPI00106495	Q9BPX3	NCAPG	Condensin complex subunit 3	114	0.161	0.019	8.47	True	False	Active	–	
1402	IPI00022276	Q9Y2Q9	MRPS28	28S ribosomal protein S28, mitochondrial	21	0.518	0.063	8.22	True	False	Active	–	
858	IPI00007175	Q9Y221	NIP7	Isoform 1 of 60S ribosome subunit biogenesis protein NIP7 homolog	20	1.124	0.131	8.58	True	False	Active	–	
897	IPI00456940	Q6DKI1	RPL7L1	60S ribosomal protein L7-like 1	29	0.777	0.091	8.54	True	False	Active	–	
566	IPI00168388	Q9UHB9	SRP68	Isoform 1 of Signal recognition particle 68 kDa protein	71	0.684	0.077	8.88	True	False	Active	–	
993	IPI00019282	P78395	PRAME	Melanoma antigen preferentially expressed in tumors	58	0.294	0.034	8.65	True	False	Active	–	
1183	IPI00005658	P11441	UBL4A	Ubiquitin-like protein 4A	18	0.800	0.092	8.70	True	False	Active	–	
1037	IPI00332499	P49321	NASP	Isoform 3 of Nuclear autoantigenic sperm protein	86	0.200	0.023	8.70	True	False	Active	–	
1265	IPI00008477	Q9ULW0	TPX2	Targeting protein for Xklp2	86	0.133	0.015	8.87	True	False	Active	–	
457	IPI00376317	Q6P2E9	EDC4	Isoform 1 of Enhancer of mRNA-decapping protein 4	152	0.366	0.052	7.04	True	False	Active	–	
560	IPI00217223	P22234	PAICS	Multifunctional protein ADE2	50	0.976	0.138	7.07	True	False	Active	–	
527	IPI00016910	Q99613	EIF3C	Eukaryotic translation initiation factor 3 subunit C	105	0.555	0.081	6.85	True	False	Active	–	
393	IPI00008529	P05387	RPLP2	60S acidic ribosomal protein P2	12	6.710	0.996	6.74	True	False	Active	–	
1067	IPI00029079	P49915	GMPS	GMP synthase [glutamine-hydrolyzing]	77	0.230	0.034	6.76	True	False	Active	–	
224	IPI00304612	P40429	RPL13A	60S ribosomal protein L13a	24	4.072	0.553	7.36	True	False	Active	–	
574	IPI00291175	P18206	VCL	Isoform 1 of Vinculin	117	0.456	0.062	7.35	True	False	Active	YES	
916	IPI00179700	P17096	HMGA1	Isoform HMG-I of High mobility group protein HMG-I/HMG-Y	12	1.698	0.230	7.38	True	False	Active	–	
1214	IPI00006865	O75396	SEC22B	Vesicle-trafficking protein SEC22b	25	0.657	0.088	7.47	True	False	Active	–	
1286	IPI00023078	Q5T750	C1orf68	Skin-specific protein 32	26	0.562	0.076	7.39	False	False	Active	–	
321	IPI00784156	P63010	AP2B1	Isoform 1 of AP-2 complex subunit beta	105	0.955	0.132	7.23	True	False	Active	–	
456	IPI00301323	Q9NVP1	DDX18	ATP-dependent RNA helicase DDX18	75	0.782	0.108	7.24	True	False	Active	–	
632	IPI00294891	P46087	NOP2	Putative uncharacterized protein NOP2	94	0.473	0.065	7.28	True	False	Active	–	
736	IPI00328715	Q86UE4	MTDH	Protein LYRIC	64	0.584	0.081	7.21	True	False	Active	–	
1140	IPI00789041	Q9H307	PNN	Isoform 1 of Pinin	82	0.146	0.020	7.30	True	False	Active	–	
1441	IPI00412771	Q9Y5K6	CD2AP	CD2-associated protein	71	0.134	0.018	7.44	True	False	Active	–	
1118	IPI00170594	Q8WYP5	AHCTF1	transcription factor ELYS	253	0.031	0.004	7.75	True	False	Active	–	
1069	IPI00018402	Q15813	TBCE	Tubulin-specific chaperone E	59	0.323	0.044	7.34	True	False	Active	–	
1238	IPI00295889	P09132	SRP19	Signal recognition particle 19 kDa protein	16	0.900	0.123	7.32	True	False	Active	–	
298	IPI00256861	Q9UPN3	MACF1	Isoform 2 of Microtubule-actin cross-linking factor 1, isoforms 1/2/3/5	620	0.159	0.020	7.95	True	False	Active	YES	
508	IPI00007423	Q92688	ANP32B	Isoform 1 of Acidic leucine-rich nuclear phosphoprotein 32 family member B	29	1.485	0.186	7.98	True	False	Active	–	
794	IPI00219913	P54578	USP14	Ubiquitin carboxyl-terminal hydrolase 14	56	0.562	0.070	8.03	True	False	Active	–	
857	IPI00006213	Q15154	PCM1	Isoform 1 of Pericentriolar material 1 protein	228	0.131	0.016	8.19	True	False	Active	–	
1312	IPI00793443	O00410	IPO5	Isoform 1 of Importin-5	124	0.125	0.016	7.81	True	False	Active	–	
477	IPI00006379	Q9Y2X3	NOP58	Nucleolar protein 58	60	1.109	0.138	8.04	True	False	Active	–	
503	IPI00328987	Q13895	BYSL	Bystin	50	1.224	0.151	8.11	True	False	Active	–	
2260	IPI00219299	Q9Y4G6	TLN2	Talin-2	272	0.334	0.041	8.15	True	False	Active	YES*	
1000	IPI00221300	Q14232	EIF2B1	Translation initiation factor eIF-2B subunit alpha	34	0.591	0.077	7.68	True	False	Active	–	
1403	IPI00013256	P33240	CSTF2	Isoform 1 of Cleavage stimulation factor subunit 2	61	0.163	0.022	7.41	True	False	Active	–	
749	IPI00004459	Q9UNQ2	DIMT1L	Probable dimethyladenosine transferase	35	1.010	0.131	7.71	True	False	Active	–	
1278	IPI00021248	P53350	PLK1	Serine/threonine-protein kinase PLK1	68	0.149	0.019	7.84	True	False	Active	–	
1364	IPI00411614	O75717	WDHD1	WD repeat and HMG-box DNA-binding protein 1	126	0.069	0.009	7.67	True	False	Active	–	
1485	IPI00027717	P57678	GEMIN4	Component of gems 4	120	0.179	0.023	7.78	True	False	Active	–	
529	IPI00105598	O00231	PSMD11	Proteasome 26S non-ATPase subunit 11 variant (Fragment)	48	1.199	0.188	6.38	True	False	Active	–	
808	IPI00477040	Q5SRE5	NUP188	Isoform 1 of Nucleoporin NUP188 homolog	196	0.126	0.020	6.30	True	False	Active	–	
535	IPI00953342	P18583	SON	Putative uncharacterized protein SON	248	0.172	0.028	6.14	True	False	Active	–	
1155	IPI00174442	Q8NCA5	FAM98A	Protein FAM98A	55	0.298	0.048	6.21	True	False	Active	–	
767	IPI00302176	Q9NY12	GAR1	Isoform 1 of H/ACA ribonucleoprotein complex subunit 1	22	1.362	0.209	6.52	True	False	Active	–	
768	IPI00302176	Q9NY12	GAR1	Isoform 1 of H/ACA ribonucleoprotein complex subunit 1	22	1.362	0.209	6.52	True	False	Active	–	
810	IPI00006079	Q9NYF8	BCLAF1	Isoform 1 of Bcl-2-associated transcription factor 1	106	0.204	0.031	6.58	False	False	Active	–	
532	IPI00006025	Q15020	SART3	Isoform 1 of Squamous cell carcinoma antigen recognized by T-cells 3	110	0.433	0.066	6.56	True	False	Active	–	
928	IPI00017373	P35244	RPA3	Replication protein A 14 kDa subunit	14	1.564	0.237	6.60	True	False	Active	–	
781	IPI00004839	P46109	CRKL	Crk-like protein	34	0.894	0.135	6.62	True	False	Active	YES	
1042	IPI00830108	Q99543	DNAJC2	Isoform 1 of DnaJ homolog subfamily C member 2	72	0.203	0.031	6.55	False	False	Active	–	
1308	IPI00006440	Q9Y2R9	MRPS7	28S ribosomal protein S7, mitochondrial	28	0.465	0.070	6.64	False	False	Active	–	
1310	IPI00453476			29 kDa protein	29	0.449	0.068	6.60	False	False	Active	–	
304	IPI00005614	Q01082	SPTBN1	Isoform Long of Spectrin beta chain, brain 1	275	0.393	0.067	5.87	True	False	Active	–	
203	IPI00643152	P34931	HSPA1L	Heat shock 70 kDa protein 1L	78	1.384	0.234	5.91	True	False	Active	–	
894	IPI00103525	Q8WXF1	PSPC1	Isoform 1 of Paraspeckle component 1	59	0.334	0.056	5.96	True	False	Active	–	
1419	IPI00760846	Q92614	MYO18A	Isoform 1 of Myosin-XVIIIa	233	0.042	0.007	6.00	False	False	Active	–	
404	IPI00655650	P62854	RPS26	40S ribosomal protein S26	13	5.855	0.959	6.11	True	False	Active	–	
1483	IPI00009335	Q9BTY7	BRP16	Brain protein 16	42	0.240	0.039	6.15	False	False	Active	–	
787	IPI00215888	O76094	SRP72	Signal recognition particle 72 kDa protein	75	0.406	0.066	6.15	True	False	Active	–	
1591	IPI00410666	Q14160	SCRIB	Isoform 3 of Protein scribble homolog	178	0.038	0.006	6.33	False	False	Active	–	
1314	IPI00296053	P07954	FH	Isoform Mitochondrial of Fumarate hydratase, mitochondrial	55	0.293	0.048	6.10	True	False	Active	–	
1711	IPI00013847	P31930	UQCRC1	Cytochrome b-c1 complex subunit 1, mitochondrial	53	0.152	0.025	6.08	True	False	Active	–	
538	IPI00143753	O15042	SR140	Isoform 1 of U2-associated protein SR140	118	0.380	0.009	42.22	True	False	Active	–	
607	IPI00329791	Q7L014	DDX46	Probable ATP-dependent RNA helicase DDX46	117	0.342	0.009	38.00	True	False	Active	–	
636	IPI00181728	Q8TDN6	BXDC2	Ribosome biogenesis protein BRX1 homolog	41	1.043	0.032	32.59	True	False	Active	–	
639	IPI00844215	Q13813	SPTAN1	Isoform 1 of Spectrin alpha chain, brain	285	0.155	0.006	25.83	True	False	Active	–	
378	IPI00005024	Q9BQG0	MYBBP1A	Isoform 1 of Myb-binding protein 1A	149	0.500	0.022	22.73	True	False	Active	–	
678	IPI00178440	P24534	EEF1B2	Elongation factor 1-beta	25	1.386	0.066	21.00	True	False	Active	–	
945	IPI00031661	Q9BVI4	NOC4L	Nucleolar complex protein 4 homolog	58	0.392	0.019	20.63	True	False	Active	–	
827	IPI00409671	Q86XP3	DDX42	Isoform 1 of ATP-dependent RNA helicase DDX42	103	0.230	0.011	20.91	True	False	Active	–	
1463	IPI00028955	Q14137	BOP1	Ribosome biogenesis protein BOP1	84	0.090	0	ND (active)	True	False	Active	–	
1465	IPI00442821	Q6ZP32	PPP2R2A	Serine/threonine protein phosphatase 2A, 55 kDa regulatory subunit B, alpha isoform	53	0.038	0	ND (active)	False	False	Active	–	
1957	IPI00103554	Q8WXI9	GATAD2B	Transcriptional repressor p66-beta	65	0.033	0	ND (active)	False	False	Active	–	
1380	IPI00169430	Q96SI9	STRBP	Isoform 1 of Spermatid perinuclear RNA-binding protein	74	0.407	0	ND (active)	True	False	Active	–	
2018	IPI00011102	P48730	CSNK1D	Isoform 1 of Casein kinase I isoform delta	47	0.031	0	ND (active)	False	False	Active	–	
1576	IPI00015973	O43491	EPB41L2	Band 4.1-like protein 2	113	0.038	0	ND (active)	False	False	Active	–	
1898	IPI00163230	Q7L5N1	COPS6	COP9 signalosome complex subunit 6	36	0.072	0	ND (active)	False	False	Active	–	
1558	IPI00006103	O95400	CD2BP2	CD2 antigen cytoplasmic tail-binding protein 2	38	0.279	0	ND (active)	True	False	Active	–	
1406	IPI00026445	Q9H9Y6	POLR1B	Isoform 2 of DNA-directed RNA polymerase I subunit RPA2	122	0.100	0	ND (active)	True	False	Active	–	
1472	IPI00019385	P51571	SSR4	Translocon-associated protein subunit delta precursor	20	0.565	0	ND (active)	True	False	Active	–	
1748	IPI00478230	Q15291	RBBP5	Retinoblastoma-binding protein 5	63	0.090	0	ND (active)	True	False	Active	–	
1572	IPI00414482	Q12789	GTF3C1	Isoform 1 of General transcription factor 3C polypeptide 1	241	0.019	0	ND (active)	True	False	Active	–	
1330	IPI00179589	P58546	MTPN	14 kDa protein	14	0.890	0	ND (active)	True	False	Active	–	
2109	IPI00018195	P27361	MAPK3	Mitogen-activated protein kinase 3	43	0.067	0	ND (active)	False	False	Active	–	
1346	IPI00031650	Q9BRJ7	NUDT16L1	Isoform 1 of Protein syndesmos	23	0.313	0	ND (active)	True	False	Active	YES	
2081	IPI00028881	O95167	NDUFA3	NADH dehydrogenase [ubiquinone] 1 alpha subcomplex subunit 3	9	0.280	0	ND (active)	False	False	Active	–	
1486	IPI00301346	Q9NW08	POLR3B	DNA-directed RNA polymerase III subunit RPC2	128	0.046	0	ND (active)	True	False	Active	–	
1735	IPI00375462	Q8WXA9	SFRS12	Isoform 2 of Splicing factor, arginine/serine-rich 12	72	0.080	0	ND (active)	True	False	Active	–	
1476	IPI00784614	Q9UHD8	SEPT9	septin 9 isoform a	65	0.178	0	ND (active)	True	False	Active	–	
1683	IPI00464952	Q05519	SFRS11	Splicing factor, arginine/serine-rich 11	54	0.080	0	ND (active)	False	False	Active	–	
1461	IPI00743813	Q8IZT6	ASPM	Isoform 1 of Abnormal spindle-like microcephaly-associated protein	410	0.015	0	ND (active)	True	False	Active	–	
1966	IPI00410284	Q8N567	ZCCHC9	Zinc finger CCHC domain-containing protein 9	30	0.048	0	ND (active)	False	False	Active	–	
1464	IPI00006987	Q9GZR7	DDX24	ATP-dependent RNA helicase DDX24	96	0.098	0	ND (active)	True	False	Active	–	
1524	IPI00385464	Q8N9Z2	FLJ36031	Isoform 1 of Uncharacterized protein FLJ36031	26	0.327	0	ND (active)	True	False	Active	–	
1167	IPI00477118	Q6NUQ4	TMEM214	Transmembrane protein 214	77	0.273	0	ND (active)	True	False	Active	–	
1264	IPI00297084	P39656	DDOST	Dolichyl-diphosphooligosaccharide--protein glycosyltransferase 48 kDa subunit	51	0.277	0	ND (active)	True	False	Active	–	
1501	IPI00549516	Q96GM8	TOE1	Target of EGR1 protein 1	57	0.138	0	ND (active)	True	False	Active	–	
1216	IPI00220766	Q04760	GLO1	Lactoylglutathione lyase	21	0.487	0	ND (active)	True	False	Active	–	
1356	IPI00024642	Q96A33	CCDC47	Isoform 1 of Coiled-coil domain-containing protein 47	56	0.188	0	ND (active)	True	False	Active	–	
2035	IPI00006187	O15379	HDAC3	Isoform 1 of Histone deacetylase 3	49	0.059	0	ND (active)	False	False	Active	–	
1969	IPI00015077	P41567	EIF1	Eukaryotic translation initiation factor 1	13	0.210	0	ND (active)	False	False	Active	–	
2150	IPI00790064	Q9Y6M9	NDUFB9	NADH dehydrogenase (ubiquinone) 1 beta subcomplex subunit 9	27	0.187	0	ND (active)	True	False	Active	–	
1244	IPI00007694	Q9Y570	PPME1	Isoform 1 of Protein phosphatase methylesterase 1	42	0.151	0	ND (active)	True	False	Active	–	
1296	IPI00000279	Q8WU90	ZC3H15	Zinc finger CCCH domain-containing protein 15	49	0.245	0	ND (active)	True	False	Active	–	
1187	IPI00019848	P51610	HCFC1	Isoform 1 of Host cell factor 1	209	0.050	0	ND (active)	True	False	Active	–	
1246	IPI00001589	Q9Y5L4	TIMM13	Mitochondrial import inner membrane translocase subunit Tim13	11	1.728	0	ND (active)	True	False	Active	–	
1755	IPI00419263	O75521	PECI	peroxisomal D3,D2-enoyl-CoA isomerase isoform 1	40	0.113	0	ND (active)	True	False	Active	–	
1563	IPI00022431	P02765	AHSG	Alpha-2-HS-glycoprotein	47	0.322	0	ND (active)	True	False	Active	–	
1145	IPI00296388	Q9UIF9	BAZ2A	Isoform 2 of Bromodomain adjacent to zinc finger domain protein 2A	211	0.036	0	ND (active)	True	False	Active	–	
2171	IPI00000398	Q9BXS6	NUSAP1	Isoform 1 of Nucleolar and spindle-associated protein 1	49	0.051	0	ND (active)	True	False	Active	–	
1706	IPI00012199	Q9H6F5	CCDC86	Coiled-coil domain-containing protein 86	40	0.175	0	ND (active)	True	False	Active	–	
1635	IPI00032903	Q9Y3E5	PTRH2	Peptidyl-tRNA hydrolase 2, mitochondrial	19	0.114	0	ND (active)	False	False	Active	–	
1991	IPI00179298	Q7Z6Z7	HUWE1	482 kDa protein	482	0.011	0	ND (active)	True	False	Active	–	
2135	IPI00329591	Q9NWS8	RMND1	Isoform 1 of Required for meiotic nuclear division protein 1 homolog	52	0.035	0	ND (active)	False	False	Active	–	
2147	IPI00157734	O60645	EXOC3	Isoform 1 of Exocyst complex component 3	87	0.025	0	ND (active)	False	False	Active	–	

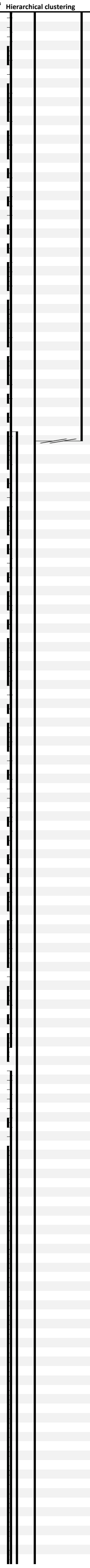

Supplementary Table 1 (cont.) | Hierarchical clustering analysis of proteins identified in active or inactive integrin adhesion complexes.

#	IPI Accession	UniProtKB Accession	Gene name	Protein name	Protein MW (kDa)	Relative abundance (mean normalised spectral count) ×10³: Active integrin	Relative abundance: Inactive integrin	Fold enrichment (active/inactive integrin)[1]	Specific enrichment[2]: Active integrin	Specific enrichment: Inactive integrin	Cluster[3]	Adhesome[4]	Hierarchical clustering
1617	IPI00000685	P50613	CDK7	Cell division protein kinase 7	39	0.129	0	ND (active)	True	False	Active	–	
2103	IPI00165477	Q9H9T3	ELP3	Isoform 1 of Elongator complex protein 3	62	0.029	0	ND (active)	False	False	Active	–	
1490	IPI00032881	Q9Y3D9	MRPS23	28S ribosomal protein S23, mitochondrial	22	0.570	0	ND (active)	True	False	Active	–	
1231	IPI00002134	Q16401	PSMD5	26S proteasome non-ATPase regulatory subunit 5	56	0.234	0	ND (active)	True	False	Active	–	
1643	IPI00639870	Q8N7B7	CT45A4	Cancer/testis antigen family 45 member A4/A6	21	0.364	0	ND (active)	True	False	Active	–	
1157	IPI00028980	Q14146	URB2	Unhealthy ribosome biogenesis protein 2 homolog	171	0.095	0	ND (active)	True	False	Active	–	
1256	IPI00062336	Q96P16	RPRD1A	Isoform 2 of Regulation of nuclear pre-mRNA domain-containing protein 1A	33	0.371	0	ND (active)	True	False	Active	–	
1579	IPI00019962	Q96EY4	C4orf43	UPF0534 protein C4orf43	24	0.345	0	ND (active)	True	False	Active	–	
1315	IPI00007004	Q9Y3B9	RRP15	RRP15-like protein	31	0.397	0	ND (active)	True	False	Active	–	
1123	IPI00470883	Q6MZP3	STAG2	stromal antigen 2 isoform a	146	0.111	0	ND (active)	True	False	Active	–	
2151	IPI00295400	P23381	WARS	Isoform 1 of Tryptophanyl-tRNA synthetase, cytoplasmic	53	0.057	0	ND (active)	False	False	Active	–	
1665	IPI00029264	P08574	CYC1	Cytochrome c1, heme protein, mitochondrial	35	0.144	0	ND (active)	True	False	Active	–	
2188	IPI00657943	Q9BSV6	TSEN34	tRNA splicing endonuclease 34 homolog	27	0.080	0	ND (active)	False	False	Active	–	
2194	IPI00794240	Q9NYK5	MRPL39	Putative uncharacterized protein MRPL39	34	0.074	0	ND (active)	False	False	Active	–	
1581	IPI00012149	O00566	MPHOSPH10	U3 small nucleolar ribonucleoprotein protein MPP10	79	0.096	0	ND (active)	True	False	Active	–	
1925	IPI00299977	Q9NRX4	PHPT1	14 kDa phosphohistidine phosphatase	14	0.334	0	ND (active)	True	False	Active	–	
1612	IPI00062037	Q96FJ2	DYNLL2	Dynein light chain 2, cytoplasmic	10	3.593	0	ND (active)	True	False	Active	–	
2041	IPI00103087	Q8WXD5	GEMIN6	Gem-associated protein 6	19	0.114	0	ND (active)	False	False	Active	–	
1860	IPI00006442	P38432	COIL	Coilin	63	0.114	0	ND (active)	True	False	Active	–	
1494	IPI00107745	O95232	LUC7L3	Isoform 1 of Luc7-like protein 3	51	0.198	0	ND (active)	True	False	Active	–	
1555	IPI00002564	P18887	XRCC1	DNA repair protein XRCC1	70	0.072	0	ND (active)	True	False	Active	–	
1996	IPI00550986	Q9NVM9	C12orf11	Cell cycle regulator Mat89Bb homolog	80	0.037	0	ND (active)	False	False	Active	–	
1247	IPI00465128	P46379	BAT3	Isoform 1 of Large proline-rich protein BAT3	119	0.114	0	ND (active)	True	False	Active	–	
1386	IPI00549540	Q9NWT1	PAK1IP1	p21-activated protein kinase-interacting protein 1	44	0.172	0	ND (active)	True	False	Active	–	
956	IPI00290460	O75821	EIF3G	Eukaryotic translation initiation factor 3 subunit G	36	0.574	0	ND (active)	True	False	Active	–	
1178	IPI00239815	Q969X6	CIRH1A	Isoform 1 of Cirhin	77	0.145	0	ND (active)	True	False	Active	–	
1285	IPI00395568	Q8IWS0	PHF6	Isoform 1 of PHD finger protein 6	41	0.214	0	ND (active)	True	False	Active	–	
1681	IPI00164949	Q8IXH7	TH1L	Isoform NELF-C of Negative elongation factor C/D	66	0.087	0	ND (active)	True	False	Active	–	
905	IPI00937477	Q15061	WDR43	WD repeat-containing protein 43	75	0.283	0	ND (active)	True	False	Active	–	
1218	IPI00714673	Q9MZ32	FN1	Fibronectin 1 isoform 4 preproprotein isoform 10	253	0.072	0	ND (active)	True	False	Active	–	
2148	IPI00478834	Q9NWB6	ARGLU1	Isoform 1 of Arginine and glutamate-rich protein 1	33	0.087	0	ND (active)	True	False	Active	–	
1787	IPI00305383	P22695	UQCRC2	Cytochrome b-c1 complex subunit 2, mitochondrial	48	0.136	0	ND (active)	True	False	Active	–	
1862	IPI00219682	P27105	STOM	Erythrocyte band 7 integral membrane protein	32	0.189	0	ND (active)	True	False	Active	–	
1876	IPI00293867	P30046	DDT	D-dopachrome decarboxylase	13	0.244	0	ND (active)	True	False	Active	–	
1781	IPI00006211	O95292	VAPB	Isoform 1 of Vesicle-associated membrane protein-associated protein B/C	27	0.429	0	ND (active)	True	False	Active	–	
1642	IPI00431697	Q6NSH3	CT45A5	Cancer/testis antigen family 45 member A5	21	0.364	0	ND (active)	True	False	Active	–	
2169	IPI00218895	P62487	POLR2G	DNA-directed RNA polymerase II subunit RPB7	19	0.076	0	ND (active)	False	False	Active	–	
2196	IPI00106928	Q96AU5	PTPN2	protein tyrosine phosphatase, non-receptor type 2	41	0.053	0	ND (active)	False	False	Active	YES	
1640	IPI00747764	O60684	KPNA6	Importin alpha-7 subunit	61	0.097	0	ND (active)	True	False	Active	–	
2000	IPI00098902	Q02218	OGDH	2-oxoglutarate dehydrogenase, mitochondrial	116	0.030	0	ND (active)	False	False	Active	–	
1245	IPI00004795	P54105	CLNS1A	Methylosome subunit pICln	26	0.612	0	ND (active)	True	False	Active	–	
1248	IPI00006702	Q8IZL8	PELP1	Proline-, glutamic acid-, leucine-rich protein 1 (PELP1)	125	0.123	0	ND (active)	True	False	Active	–	
1666	IPI00023649	A3KN83	SBNO1	Isoform 1 of Protein strawberry notch homolog 1	154	0.016	0	ND (active)	False	False	Active	–	
963	IPI00719680	O14617	AP3D1	Isoform 5 of AP-3 complex subunit delta-1	137	0.149	0	ND (active)	True	False	Active	–	
1266	IPI00024157	Q00688	FKBP3	Peptidyl-prolyl cis-trans isomerase FKBP3	25	0.507	0	ND (active)	True	False	Active	–	
2078	IPI00470537	Q68CP9	ARID2	Isoform 1 of AT-rich interactive domain-containing protein 2	197	0.013	0	ND (active)	True	False	Active	–	
1633	IPI00873809	Q9Y5X1	SNX9	Sorting nexin-9	46	0.146	0	ND (active)	True	False	Active	–	
830	IPI00102069	Q7L2H7	EIF3M	Eukaryotic translation initiation factor 3 subunit M	43	0.738	0	ND (active)	True	False	Active	–	
1099	IPI00549664	Q9NXF1	TEX10	Testis-expressed sequence 10 protein	106	0.156	0	ND (active)	True	False	Active	–	
887	IPI00645369	Q5T0V7	DST	dystonin isoform 2	650	0.004	0	ND (active)	False	False	Active	–	
2227	IPI00001364	Q9Y5B6	GCFC1	Isoform A of GC-rich sequence DNA-binding factor homolog	105	0.021	0	ND (active)	False	False	Active	–	
2028	IPI00299526	Q15022	SUZ12	Polycomb protein SUZ12	83	0.043	0	ND (active)	True	False	Active	–	
1820	IPI00008247	Q9UJX4	ANAPC5	Isoform 1 of Anaphase-promoting complex subunit 5	85	0.064	0	ND (active)	True	False	Active	–	
1840	IPI00658109	P12532	CKMT1A	Isoform 1 of Creatine kinase U-type, mitochondrial	47	0.204	0	ND (active)	True	False	Active	–	
1740	IPI00176706	Q96I25	RBM17	Splicing factor 45	45	0.136	0	ND (active)	True	False	Active	–	
1545	IPI00004353	P52657	GTF2A2	Transcription initiation factor IIA subunit 2	12	0.540	0	ND (active)	True	False	Active	–	
1299	IPI00790299	Q9NWH9	SLTM	68 kDa protein	68	0.117	0	ND (active)	True	False	Active	–	
2209	IPI00060107	Q96BP2	CHCHD1	Coiled-coil-helix-coiled-coil-helix domain-containing protein 1	13	0.194	0	ND (active)	False	False	Active	–	
2222	IPI00063903	Q96IX5	USMG5	Up-regulated during skeletal muscle growth protein 5	6	0.336	0	ND (active)	False	False	Active	–	
1836	IPI00216412	Q8IYT3	C6orf97	Isoform 2 of Coiled-coil domain-containing protein C6orf97	83	0.022	0	ND (active)	False	False	Active	–	
1790	IPI00022215	Q9H2P0	ADNP	Activity-dependent neuroprotector homeobox protein	124	0.053	0	ND (active)	True	False	Active	–	
1475	IPI00011857	Q13112	CHAF1B	Chromatin assembly factor 1 subunit B	61	0.169	0	ND (active)	True	False	Active	–	
2122	IPI00020984	P27824	CANX	Calnexin	72	0.056	0	ND (active)	True	False	Active	–	
1523	IPI00025796	O75489	NDUFS3	NADH dehydrogenase [ubiquinone] iron-sulfur protein 3, mitochondrial	30	0.346	0	ND (active)	True	False	Active	–	
2040	IPI00643554	O43665	RGS10	Isoform 3 of Regulator of G-protein signaling 10	21	0.069	0	ND (active)	False	False	Active	–	
1091	IPI00003886	Q9BVP2	GNL3	Isoform 2 of Guanine nucleotide-binding protein-like 3	61	0.324	0	ND (active)	True	False	Active	–	
1195	IPI00221114	Q9UPN9	TRIM33	Isoform Beta of E3 ubiquitin-protein ligase TRIM33	121	0.110	0	ND (active)	True	False	Active	–	
1422	IPI00018500	Q99856	ARID3A	AT-rich interactive domain-containing protein 3A	63	0.198	0	ND (active)	True	False	Active	–	
1033	IPI00026320	O95071	UBR5	E3 ubiquitin-protein ligase UBR5	309	0.042	0	ND (active)	True	False	Active	–	
1132	IPI00154590	Q9BYG3	MKI67IP	MKI67 FHA domain-interacting nucleolar phosphoprotein	34	0.610	0	ND (active)	True	False	Active	–	
1234	IPI00419473	P20290	BTF3	Isoform 2 of Transcription factor BTF3	18	0.736	0	ND (active)	True	False	Active	–	
1363	IPI00006970	Q9Y399	MRPS2	Mitochondrial 28S ribosomal protein S2	36	0.320	0	ND (active)	True	False	Active	–	
936	IPI00019640	Q99986	VRK1	Serine/threonine-protein kinase VRK1	45	0.475	0	ND (active)	True	False	Active	–	
991	IPI00300659	Q6P1J9	CDC73	Parafibromin	61	0.387	0	ND (active)	True	False	Active	–	
1885	IPI00177437	Q9BZJ0	CRNKL1	Isoform 1 of Crooked neck-like protein 1	100	0.022	0	ND (active)	False	False	Active	–	
1825	IPI00032791	O95707	POP4	Ribonuclease P protein subunit p29	25	0.245	0	ND (active)	True	False	Active	–	
815	IPI00784161	Q7KZ85	SUPT6H	Isoform 1 of Transcription elongation factor SPT6	199	0.113	0	ND (active)	True	False	Active	–	
872	IPI00156374	Q8TEX9	IPO4	Isoform 1 of Importin-4	119	0.238	0	ND (active)	True	False	Active	–	
1068	IPI00028005	P57740	NUP107	Nuclear pore complex protein Nup107	106	0.187	0	ND (active)	True	False	Active	–	
1166	IPI00332428	Q9BXY0	MAK16	Protein MAK16 homolog	35	0.482	0	ND (active)	True	False	Active	–	
1857	IPI00434390	Q8N9N2	ASCC1	Activating signal cointegrator 1 complex subunit 1 (ASCC1)	44	0.139	0	ND (active)	True	False	Active	–	
1878	IPI00218493	P00492	HPRT1	Hypoxanthine-guanine phosphoribosyltransferase	25	0.173	0	ND (active)	True	False	Active	–	
1271	IPI00429191	P62495	ETF1	Eukaryotic peptide chain release factor subunit 1	49	0.325	0	ND (active)	True	False	Active	–	
2239	IPI00300060	Q9NW82	WDR70	WD repeat-containing protein 70	73	0.020	0	ND (active)	False	False	Active	–	
2247	IPI00003310	P52435	POLR2J	RPB11a protein	14	0.103	0	ND (active)	False	False	Active	–	
1987	IPI00006900	Q9NQZ2	UTP3	Something about silencing protein 10	55	0.065	0	ND (active)	True	False	Active	–	
1488	IPI00306708	Q96KB5	PBK	T-lymphokine-activated killer cell-originated protein kinase	37	0.212	0	ND (active)	True	False	Active	–	
2197	IPI00419626	Q7Z7F7	MRPL55	Isoform 2 of 39S ribosomal protein L55, mitochondrial	19	0.163	0	ND (active)	True	False	Active	–	
2238	IPI00017184	Q9H4M9	EHD1	EH domain-containing protein 1	61	0.033	0	ND (active)	False	False	Active	–	
1903	IPI00009958	Q92905	COPS5	COP9 signalosome complex subunit 5	38	0.186	0	ND (active)	True	False	Active	–	
1233	IPI00242273	Q92925	SMARCD2	SWI/SNF-related matrix-associated actin-dependent regulator of chromatin d2 isoform 2	57	0.241	0	ND (active)	True	False	Active	–	
1165	IPI00301434	Q9H3K6	BOLA2	BolA-like protein 2	17	0.796	0	ND (active)	True	False	Active	–	
2210	IPI00217143	Q8IW48	SDHA	SDHA protein	57	0.053	0	ND (active)	False	False	Active	–	
1639	IPI00027448	O75964	ATP5L	ATP synthase subunit g, mitochondrial	11	0.890	0	ND (active)	True	False	Active	–	
1606	IPI00002938	Q9ULW3	ABT1	Activator of basal transcription 1	31	0.232	0	ND (active)	True	False	Active	–	
1580	IPI00156032	Q13601	KRR1	KRR1 small subunit processome component homolog	44	0.139	0	ND (active)	True	False	Active	–	
1858	IPI00015351	Q9GZP4	C1orf128	Isoform 1 of UPF0424 protein C1orf128	24	0.225	0	ND (active)	True	False	Active	–	
1389	IPI00032423	Q9Y2R4	DDX52	Probable ATP-dependent RNA helicase DDX52	67	0.151	0	ND (active)	True	False	Active	–	
1431	IPI00439194	O95983	MBD3	Isoform 1 of Methyl-CpG-binding domain protein 3	33	0.284	0	ND (active)	True	False	Active	–	
1383	IPI00303105	P63165	SUMO1	Small ubiquitin-related modifier 1	12	0.510	0	ND (active)	True	False	Active	–	
469	IPI00299147	P55854	SUMO3	Small ubiquitin-related modifier 3	12	1.021	0	ND (active)	False	False	Active	–	
563	IPI00014151	Q15008	PSMD6	26S proteasome non-ATPase regulatory subunit 6	46	1.038	0	ND (active)	True	False	Active	–	
1213	IPI00299417	Q13769	THOC5	THO complex subunit 5 homolog	79	0.170	0	ND (active)	True	False	Active	–	
1456	IPI00014624	Q92572	AP3S1	AP-3 complex subunit sigma-1	32	0.290	0	ND (active)	True	False	Active	–	
1782	IPI00176903	Q6NZI2	PTRF	Isoform 1 of Polymerase I and transcript release factor	43	0.132	0	ND (active)	False	False	Active	–	
1822	IPI00031410	P42345	FRAP1	Serine/threonine-protein kinase mTOR	289	0.016	0	ND (active)	True	False	Active	–	
1815	IPI00219525	P52209	PGD	6-phosphogluconate dehydrogenase, decarboxylating	53	0.095	0	ND (active)	True	False	Active	–	
1797	IPI00006091	P11532	DMD	Isoform 4 of Dystrophin	427	0.007	0	ND (active)	True	False	Active	–	
1783	IPI00003739	Q9Y6V7	DDX49	Probable ATP-dependent RNA helicase DDX49	54	0.067	0	ND (active)	True	False	Active	–	
1744	IPI00647188	Q4G0P3	HYDIN	Isoform 1 of Hydrocephalus-inducing protein homolog	576	0.004	0	ND (active)	False	False	Active	–	
1746	IPI00794880	Q9P2D1	CHD7	Isoform 1 of Chromodomain-helicase-DNA-binding protein 7	336	0.005	0	ND (active)	False	False	Active	–	
1871	IPI00005087	Q9NYL9	TMOD3	Tropomodulin-3	40	0.169	0	ND (active)	False	False	Active	–	
1730	IPI00743121	Q9NXE4	SMPD4	sphingomyelin phosphodiesterase 4 isoform 1	95	0.057	0	ND (active)	False	False	Active	–	
1731	IPI00743121	Q9NXE4	SMPD4	sphingomyelin phosphodiesterase 4 isoform 1	95	0.057	0	ND (active)	False	False	Active	–	
1283	IPI00409635	A0FGR8	ESYT2	Isoform 2 of Extended synaptotagmin-2	99	0.143	0	ND (active)	True	False	Active	–	
2064	IPI00305887	Q9BZD4	NUF2	Kinetochore protein Nuf2	54	0.040	0	ND (active)	False	False	Active	–	
1560	IPI00217541	Q8N8A6	DDX51	ATP-dependent RNA helicase DDX51	72	0.110	0	ND (active)	True	False	Active	–	
1268	IPI00294229	Q9Y2P8	RCL1	RNA 3'-terminal phosphate cyclase-like protein	41	0.371	0	ND (active)	True	False	Active	–	
1381	IPI00333215	P23193	TCEA1	Isoform 1 of Transcription elongation factor A protein 1	34	0.381	0	ND (active)	True	False	Active	–	
646	IPI00010320	P83916	CBX1	Chromobox protein homolog 1	21	1.732	0	ND (active)	True	False	Active	–	
651	IPI00185919	Q6PKG0	LARP1	Isoform 1 of La-related protein 1	124	0.250	0	ND (active)	True	False	Active	–	
1016	IPI00032523	Q9BQY4	RHOXF2	Rhox homeobox family member 2	32	0.079	0	ND (active)	False	False	Active	–	
2114	IPI00395865	Q16576	RBBP7	Histone-binding protein RBBP7	48	0.188	0	ND (active)	False	False	Active	–	
1856	IPI00017704	Q14019	COTL1	Coactosin-like protein	16	0.248	0	ND (active)	True	False	Active	–	
431	IPI00004970	O75691	UTP20	Small subunit processome component 20 homolog	318	0.175	0	ND (active)	True	False	Active	–	
613	IPI00022256	Q96CW1	AP2M1	Isoform 1 of AP-2 complex subunit mu	50	0.986	0	ND (active)	True	False	Active	–	
703	IPI00642238	Q5SSJ5	HP1BP3	Isoform 1 of Heterochromatin protein 1-binding protein 3	61	0.638	0	ND (active)	True	False	Active	–	
1417	IPI00329536	Q15075	EEA1	Early endosome antigen 1	162	0.009	0	ND (active)	False	False	Active	–	
405	IPI00101186	Q5JTH9	RRP12	Isoform 1 of RRP12-like protein	144	0.434	0	ND (active)	True	False	Active	–	
879	IPI00385267	P08240	SRPR	Signal recognition particle receptor subunit alpha	70	0.424	0	ND (active)	True	False	Active	–	
2155	IPI00018040	Q15528	MED22	Isoform Surf5B of Mediator of RNA polymerase II transcription subunit 22	22	0.229	0	ND (active)	True	False	Active	–	
28	IPI00217465	P16403	HIST1H1C	Histone H1.2	21	23.811	0	ND (active)	True	False	Active	–	
582	IPI00100716	Q9UHX1	PUF60	Isoform 2 of Poly(U)-binding-splicing factor PUF60	58	0.834	0	ND (active)	True	False	Active	–	
2241	IPI00017672	P00491	NP	PURINE NUCLEOSIDE PHOSPHORYLASE	33	0.065	0	ND (active)	False	False	Active	–	
1039	IPI00916569	Q96G21	IMP4	27 kDa protein	27	0.707	0	ND (active)	True	False	Active	–	
2219	IPI00028520	P49821	NDUFV1	Isoform 1 of NADH dehydrogenase [ubiquinone] flavoprotein 1, mitochondrial	51	0.049	0	ND (active)	False	False	Active	–	
557	IPI00020021	P35659	DEK	Protein DEK	43	1.122	0	ND (active)	True	False	Active	–	
809	IPI00003965	Q93009	USP7	Ubiquitin carboxyl-terminal hydrolase 7	128	0.225	0	ND (active)	True	False	Active	–	
2246	IPI00103521	Q8WXE9	STON2	Isoform 1 of Stonin-2	101	0.020	0	ND (active)	False	False	Active	–	
2218	IPI00014367	Q9NXX6	NSMCE4A	Isoform 1 of Non-structural maintenance of chromosomes element 4 homolog A	44	0.041	0	ND (active)	False	False	Active	–	
1830	IPI00165092	Q9Y2Z4	YARS2	Tyrosyl-tRNA synthetase, mitochondrial	53	0.116	0	ND (active)	True	False	Active	–	
1827	IPI00020472	Q9P0I2	TMEM111	Isoform 1 of Transmembrane protein 111	30	0.180	0	ND (active)	True	False	Active	–	
1826	IPI00005161	O15144	ARPC2	Actin-related protein 2/3 complex subunit 2	34	0.207	0	ND (active)	True	False	Active	YES	
1854	IPI00220014	Q13907	IDI1	Isoform 2 of Isopentenyl-diphosphate Delta-isomerase 1	32	0.101	0	ND (active)	True	False	Active	–	
1861	IPI00101645	Q96HN2	AHCYL2	Putative adenosylhomocysteinase 3	67	0.022	0	ND (active)	False	False	Active	–	
1101	IPI00006099	Q14692	BMS1	Ribosome biogenesis protein BMS1 homolog	146	0.094	0	ND (active)	True	False	Active	–	
889	IPI00292753	Q14C86	GAPVD1	Isoform 6 of GTPase-activating protein and VPS9 domain-containing protein 1	166	0.164	0	ND (active)	True	False	Active	–	
2253	IPI00019983	Q07817	BCL2L1	Isoform Bcl-X(L) of Bcl-2-like protein 1	26	0.083	0	ND (active)	False	False	Active	–	
1521	IPI00012202	Q9BQA1	WDR77	Methylosome protein 50	37	0.282	0	ND (active)	True	False	Active	–	
1791	IPI00646689	Q9BRA2	TXNDC17	Thioredoxin domain-containing protein 17	14	0.360	0	ND (active)	True	False	Active	–	
1883	IPI00165506	Q9Y2S7	POLDIP2	Polymerase delta-interacting protein 2	42	0.065	0	ND (active)	False	False	Active	–	
1762	IPI00015105	P33552	CKS2	Cyclin-dependent kinases regulatory subunit 2	10	0.180	0	ND (active)	False	False	Active	–	
971	IPI00027192	Q02809	PLOD1	Procollagen-lysine, 2-oxoglutarate 5-dioxygenase 1	88	0.173	0	ND (active)	True	False	Active	–	
2230	IPI00007311	O15525	MAFG	Transcription factor MafG	18	0.140	0	ND (active)	True	False	Active	–	
2170	IPI00031563	Q9BW61	DDA1	DET1- and DDB1-associated protein 1	12	0.180	0	ND (active)	False	False	Active	–	
1938	IPI00009444	Q9P0M9	MRPL27	39S ribosomal protein L27, mitochondrial	16	0.409	0	ND (active)	True	False	Active	–	
1592	IPI00642584	Q8N766	KIAA0090	Isoform 1 of Uncharacterized protein KIAA0090	112	0.099	0	ND (active)	True	False	Active	–	
1108	IPI00036742	Q69YN4	KIAA1429	Isoform 1 of Protein virilizer homolog	202	0.065	0	ND (active)	True	False	Active	–	
1100	IPI00014253	Q15050	RRS1	Ribosome biogenesis regulatory protein homolog	41	0.297	0	ND (active)	True	False	Active	–	
2229	IPI00103467	P30837	ALDH1B1	Aldehyde dehydrogenase X, mitochondrial	57	0.035	0	ND (active)	False	False	Active	–	
2121	IPI00030098	Q14320	FAM50A	Protein FAM50A	40	0.126	0	ND (active)	True	False	Active	–	
1765	IPI00220503	Q13561	DCTN2	dynactin 2	45	0.162	0	ND (active)	True	False	Active	–	
1916	IPI00022790	P55081	MFAP1	Microfibrillar-associated protein 1	52	0.090	0	ND (active)	True	False	Active	–	
1508	IPI00152510	Q8TDD1	DDX54	DEAD (Asp-Glu-Ala-Asp) box polypeptide 54 isoform 1	99	0.100	0	ND (active)	True	False	Active	–	
2089	IPI00166873	Q8N5L8	C9orf23	Alba-like protein C9orf23	18	0.140	0	ND (active)	True	False	Active	–	

Supplementary Table 1 (cont.) | Hierarchical clustering analysis of proteins identified in active or inactive integrin adhesion complexes.

#	IPI Accession	UniProtKB Accession	Gene name	Protein name	Protein MW (kDa)	Relative abundance (mean normalised spectral count) ×10^3		Fold enrichment (active/inactive integrin)[1]	Specific enrichment[2]		Cluster[3]	Adhesome[4]	Hierarchical clustering
						Active integrin	Inactive integrin		Active integrin	Inactive integrin			
2203	IPI00002203	Q9P287	BCCIP	Isoform 1 of BRCA2 and CDKN1A-interacting protein	36	0.080	0	ND (active)	True	False	Active	–	
2213	IPI00333699	Q9UHR5	SAP30BP	Isoform 2 of SAP30-binding protein	32	0.101	0	ND (active)	True	False	Active	–	
1668	IPI00025333	Q6FI81	CIAPIN1	Isoform 3 of Anamorsin	32	0.200	0	ND (active)	True	False	Active	–	
1776	IPI00005826	O95714	HERC2	Probable E3 ubiquitin-protein ligase HERC2	527	0.007	0	ND (active)	False	False	Active	–	
2126	IPI00306207	Q6NXR4	C8orf41	Uncharacterized protein C8orf41	57	0.025	0	ND (active)	False	False	Active	–	
2157	IPI00154645	Q8TC07	TBC1D15	TBC1 domain family, member 15 isoform 1	82	0.040	0	ND (active)	False	False	Active	–	
1520	IPI00217236	O75347	TBCA	Tubulin-specific chaperone A	13	0.903	0	ND (active)	True	False	Active	–	
1618	IPI00012773	Q13330	MTA1	Isoform Long of Metastasis-associated protein MTA1	81	0.084	0	ND (active)	False	False	Active	–	
1387	IPI00384909	Q9ULH0	KIDINS220	Isoform 2 of Kinase D-interacting substrate of 220 kDa	186	0.076	0	ND (active)	True	False	Active	–	
1433	IPI00952909	Q9NVA2	SEPT11	Isoform 1 of Septin-11	49	0.259	0	ND (active)	True	False	Active	–	
426	IPI00216654	Q14978	NOLC1	Isoform Beta of Nucleolar and coiled-body phosphoprotein 1	75	0.913	0	ND (active)	True	False	Active	–	
934	IPI00010404	Q9BWJ5	SF3B5	Splicing factor 3B subunit 5	10	2.685	0	ND (active)	True	False	Active	–	
1098	IPI00292894	Q2NL82	TSR1	Pre-rRNA-processing protein TSR1 homolog	92	0.221	0	ND (active)	True	False	Active	–	
1832	IPI00009070	Q9Y450	HBS1L	Isoform 1 of HBS1-like protein	75	0.054	0	ND (active)	True	False	Active	–	
627	IPI00019770	P35544	FAU	ubiquitin-like protein fubi and ribosomal protein S30 precursor	14	2.201	0	ND (active)	True	False	Active	–	
861	IPI00023972	Q9H0S4	DDX47	Probable ATP-dependent RNA helicase DDX47	51	0.626	0	ND (active)	True	False	Active	–	
2235	IPI00299413	Q06546	GABPA	GA-binding protein alpha chain	51	0.035	0	ND (active)	False	False	Active	–	
1478	IPI00217240	Q8IWA0	WDR75	WD repeat-containing protein 75	95	0.080	0	ND (active)	True	False	Active	–	
1270	IPI00007089	O95478	NSA2	Ribosome biogenesis protein NSA2 homolog	30	0.439	0	ND (active)	True	False	Active	–	
902	IPI00304232	Q9GZL7	WDR12	Ribosome biogenesis protein WDR12	48	0.524	0	ND (active)	True	False	Active	–	
2263	IPI00910208	Q05519	SRSF11	Splicing factor arginine/serine-rich 11	13	0.111	0	ND (active)	False	False	Active	–	
1937	IPI00023086	Q9P015	MRPL15	39S ribosomal protein L15, mitochondrial	33	0.153	0	ND (active)	True	False	Active	–	
1816	IPI00414410	Q9NWU5	MRPL22	Isoform 1 of 39S ribosomal protein L22, mitochondrial	24	0.237	0	ND (active)	False	False	Active	–	
1796	IPI00010951	P58107	EPPK1	epiplakin 1	556	0.003	0	ND (active)	False	False	Active	–	
1792	IPI00794194	P55039	DRG2	Putative uncharacterized protein DRG2	38	0.066	0	ND (active)	False	False	Active	–	
1328	IPI00646000	A6XGL0	YJEFN3	Isoform 1 of YjeF N-terminal domain-containing protein 3	33	0.076	0	ND (active)	False	False	Active	–	
918	IPI00168262	Q8NBJ5	GLT25D1	Procollagen galactosyltransferase 1	72	0.346	0	ND (active)	True	False	Active	–	
2248	IPI00028109	Q9C005	DPY30	Protein dpy-30 homolog	11	0.164	0	ND (active)	False	False	Active	–	
2011	IPI00018196	Q9NVX2	NLE1	Notchless protein homolog 1	53	0.068	0	ND (active)	True	False	Active	–	
1809	IPI00299554	Q15058	KIF14	Kinesin-like protein KIF14	186	0.019	0	ND (active)	True	False	Active	–	
1896	IPI00848328			Delta(3,5)-Delta(2,4)-dienoyl-CoA isomerase, mitochondrial precursor	36	1.260	0	ND (active)	True	False	Active	–	
1941	IPI00016839	Q92759	GTF2H4	General transcription factor IIH subunit 4	52	0.090	0	ND (active)	True	False	Active	–	
1732	IPI00163496	Q13136	PPFIA1	Isoform 1 of Liprin-alpha-1	136	0.049	0	ND (active)	True	False	Active	YES	
2189	IPI00014230	Q07021	C1QBP	Complement component 1 Q subcomponent-binding protein, mitochondrial	31	0.065	0	ND (active)	False	False	Active	–	
2207	IPI00394679	Q9H3P2	WHSC2	Wolf-Hirschhorn syndrome candidate 2 protein	59	0.043	0	ND (active)	True	False	Active	–	
1725	IPI00293921	Q92547	TOPBP1	DNA topoisomerase 2-binding protein 1	171	0.029	0	ND (active)	True	False	Active	–	
1983	IPI00789008	Q14254	FLOT2	Flotillin-2	47	0.086	0	ND (active)	True	False	Active	–	
1567	IPI00064931	Q96L91	EP400	Isoform 2 of E1A-binding protein p400	340	0.017	0	ND (active)	True	False	Active	–	
1613	IPI00000769	Q14807	KIF22	Kinesin-like protein KIF22	73	0.092	0	ND (active)	True	False	Active	–	
1561	IPI00217686	Q8IY81	FTSJ3	Putative rRNA methyltransferase 3	97	0.071	0	ND (active)	True	False	Active	–	
1891	IPI00097492	Q96ME7	ZNF512	Zinc finger protein 512	65	0.039	0	ND (active)	True	False	Active	–	
1044	IPI00479946	Q3ZCU9	STIP1	STIP1 protein	68	0.286	0	ND (active)	True	False	Active	–	
904	IPI00303813	Q9H8H0	NOL11	Nucleolar protein 11	81	0.275	0	ND (active)	True	False	Active	–	
2262	IPI00644171			Putative uncharacterized protein RPL17P9	21	1.272	0	ND (active)	False	False	Active	–	
1824	IPI00029107	Q14191	WRN	Werner syndrome ATP-dependent helicase	163	0.033	0	ND (active)	True	False	Active	–	
1839	IPI00027415	Q9H2U1	DHX36	Isoform 1 of Probable ATP-dependent RNA helicase DHX36	115	0.034	0	ND (active)	True	False	Active	–	
1794	IPI00061531	Q96EL3	MRPL53	39S ribosomal protein L53, mitochondrial	12	0.462	0	ND (active)	True	False	Active	–	
937	IPI00020128	Q9NYH9	UTP6	U3 small nucleolar RNA-associated protein 6 homolog	70	0.258	0	ND (active)	True	False	Active	–	
2237	IPI00335541	Q9UNS1	TIMELESS	Isoform 1 of Protein timeless homolog	139	0.010	0	ND (active)	False	False	Active	–	
2143	IPI00020530	Q9NPJ3	ACOT13	Acyl-coenzyme A thioesterase 13	15	0.202	0	ND (active)	False	False	Active	–	
2075	IPI00383680	Q5JYR6	RPN2	ribophorin II isoform 2 precursor	68	0.062	0	ND (active)	False	False	Active	–	
1882	IPI00940614	Q53EL6	PDCD4	50 kDa protein	50	0.109	0	ND (active)	True	False	Active	–	
1766	IPI00328293	Q8IYB3	SRRM1	Serine/arginine repetitive matrix protein 1	104	0.058	0	ND (active)	True	False	Active	–	
1649	IPI00181617	P42696	RBM34	Isoform 1 of RNA-binding protein 34	49	0.146	0	ND (active)	True	False	Active	–	
1267	IPI00003921	P11171	EPB41	Isoform 1 of Protein 4.1	97	0.171	0	ND (active)	True	False	Active	–	
2208	IPI00019906	P35613	BSG	Isoform 2 of Basigin	29	0.122	0	ND (active)	False	False	Active	–	
1954	IPI00844000	O94874	KIAA0776	Isoform 1 of E3 UFM1-protein ligase 1	90	0.047	0	ND (active)	True	False	Active	–	
1559	IPI00004290	Q68CQ4	DEF	Digestive organ expansion factor homolog	87	0.099	0	ND (active)	True	False	Active	–	
1365	IPI00021828	P04080	CSTB	Cystatin-B	11	1.237	0	ND (active)	True	False	Active	–	
1481	IPI00644674	Q9Y5Y2	NUBP2	Cytosolic Fe-S cluster assembly factor NUBP2	29	0.112	0	ND (active)	True	False	Active	–	
1723	IPI00735681	Q86Y91	KIF18B	kinesin family member 18B	93	0.027	0	ND (active)	False	False	Active	–	
1604	IPI00013146	P82650	MRPS22	28S ribosomal protein S22, mitochondrial	41	0.221	0	ND (active)	True	False	Active	–	
1641	IPI00015104	P61024	CKS1B	Cyclin-dependent kinases regulatory subunit 1	10	0.576	0	ND (active)	True	False	Active	–	
1679	IPI00216719	Q8IUD2	ERC1	Isoform 1 of ELKS/RAB6-interacting/CAST family member 1	128	0.051	0	ND (active)	True	False	Active	–	
1243	IPI00022042	Q8NBF6	AVL9	Isoform 2 of Late secretory pathway protein AVL9 homolog	65	0.031	0	ND (active)	False	False	Active	–	
742	IPI00009471	Q9UNX4	WDR3	WD repeat-containing protein 3	106	0.231	0	ND (active)	True	False	Active	–	
2251	IPI00061178	Q96E39	RBMXL1	RNA binding motif protein, X-linked-like 1	42	0.918	0	ND (active)	False	False	Active	–	
696	IPI00411886	Q9Y3T9	NOC2L	Nucleolar complex protein 2 homolog	85	0.337	0	ND (active)	True	False	Active	–	
2243	IPI00029193	Q04756	HGFAC	Hepatocyte growth factor activator	71	0.036	0	ND (active)	True	False	Active	–	
2097	IPI00296099	P07996	THBS1	Thrombospondin-1	129	0.017	0	ND (active)	False	False	Active	–	
1846	IPI00000690	O95831	AIFM1	Isoform 1 of Apoptosis-inducing factor 1, mitochondrial	67	0.060	0	ND (active)	True	False	Active	–	
1855	IPI00299033	O00505	KPNA3	Importin subunit alpha-3	58	0.099	0	ND (active)	True	False	Active	–	
969	IPI00060627	Q96CT7	CCDC124	Coiled-coil domain-containing protein 124	26	0.778	0	ND (active)	True	False	Active	–	
2240	IPI00015773	Q9UBI1	COMMD3	COMM domain-containing protein 3	22	0.115	0	ND (active)	True	False	Active	–	
2124	IPI00748700	Q9BRX5	GINS3	Isoform 1 of DNA replication complex GINS protein PSF3	25	0.115	0	ND (active)	True	False	Active	–	
1759	IPI00006552	Q9UIV1	CNOT7	CCR4-NOT transcription complex subunit 7	33	0.199	0	ND (active)	True	False	Active	–	
1848	IPI00026546	P68402	PAFAH1B2	Platelet-activating factor acetylhydrolase IB subunit beta	26	0.233	0	ND (active)	True	False	Active	–	
1887	IPI00385321	Q9ULU4	ZMYND8	Isoform 2 of Protein kinase C-binding protein 1	67	0.070	0	ND (active)	True	False	Active	–	
1910	IPI00100748	Q9NZL4	HSPBP1	Hsp70-binding protein 1	44	0.116	0	ND (active)	True	False	Active	–	
1291	IPI00030770	O95456	PSMG1	Isoform 1 of Proteasome assembly chaperone 1	33	0.377	0	ND (active)	True	False	Active	–	
2202	IPI00181359	Q99590	SFRS2IP	Isoform 2 of SFRS2-interacting protein	129	0.011	0	ND (active)	False	False	Active	–	
1775	IPI00298731	Q96QC0	PPP1R10	Serine/threonine-protein phosphatase 1 regulatory subunit 10	99	0.033	0	ND (active)	True	False	Active	–	
1350	IPI00644231	Q7L576	CYFIP1	Isoform 1 of Cytoplasmic FMR1-interacting protein 1	145	0.089	0	ND (active)	True	False	Active	–	
2086	IPI00301489	P06132	UROD	Uroporphyrinogen decarboxylase	41	0.111	0	ND (active)	True	False	Active	–	
1788	IPI00747787	P42695	NCAPD3	Condensin-2 complex subunit D3	169	0.021	0	ND (active)	True	False	Active	–	
1190	IPI00152708	Q8TED0	UTP15	U3 small nucleolar RNA-associated protein 15 homolog	58	0.205	0	ND (active)	True	False	Active	–	
911	IPI00297241	O60287	URB1	Nucleolar pre-ribosomal-associated protein 1	254	0.087	0	ND (active)	True	False	Active	–	
2242	IPI00002646	Q9UBK9	UXT	Protein UXT	18	0.140	0	ND (active)	True	False	Active	–	
771	IPI00552073	O60231	DHX16	Putative pre-mRNA-splicing factor ATP-dependent RNA helicase DHX16	119	0.205	0	ND (active)	True	False	Active	–	
840	IPI00013891	Q13595	TRA2A	Isoform Long of Transformer-2 protein homolog alpha	33	0.958	0	ND (active)	True	False	Active	–	
2141	IPI00797749	Q12846	STX4	Syntaxin 4A (Placental), isoform CRA_c	34	0.133	0	ND (active)	True	False	Active	–	
1462	IPI00014177	Q15019	SEPT2	Isoform 1 of Septin-2	41	0.260	0	ND (active)	True	False	Active	–	
1867	IPI00003394	Q16637	SMN1	Isoform SMN of Survival motor neuron protein	32	0.079	0	ND (active)	True	False	Active	–	
1818	IPI00029629	Q14258	TRIM25	E3 ubiquitin/ISG15 ligase TRIM25	71	0.086	0	ND (active)	True	False	Active	–	
1853	IPI00019901	P35611	ADD1	Isoform 1 of Alpha-adducin	81	0.081	0	ND (active)	True	False	Active	–	
1859	IPI00008274	Q01518	CAP1	Isoform 1 of Adenylyl cyclase-associated protein 1	52	0.155	0	ND (active)	True	False	Active	–	
1449	IPI00456429	P62987	UBA52	ubiquitin and ribosomal protein L40 precursor	15	18.649	0	ND (active)	True	False	Active	–	
844	IPI00295098	Q9Y5M8	SRPRB	Signal recognition particle receptor subunit beta	30	0.972	0	ND (active)	True	False	Active	–	
2254	IPI00162330	Q9BZE1	MRPL37	39S ribosomal protein L37, mitochondrial	48	0.042	0	ND (active)	False	False	Active	–	
1979	IPI00034159	P61421	ATP6V0D1	V-type proton ATPase subunit d 1	40	0.189	0	ND (active)	True	False	Active	–	
1784	IPI00254408	Q12830	BPTF	Isoform 2 of Nucleosome-remodeling factor subunit BPTF	325	0.008	0	ND (active)	False	False	Active	–	
1793	IPI00293331	Q99575	POP1	Ribonucleases P/MRP protein subunit POP1	115	0.047	0	ND (active)	True	False	Active	–	
1764	IPI00339384	Q8TC12	RDH11	Isoform 1 of Retinol dehydrogenase 11	35	0.173	0	ND (active)	True	False	Active	–	
1895	IPI00031461	P50395	GDI2	Rab GDP dissociation inhibitor beta	51	0.138	0	ND (active)	True	False	Active	–	
1713	IPI00025366	O75390	CS	Citrate synthase, mitochondrial	52	0.145	0	ND (active)	True	False	Active	–	
1671	IPI00006979	Q9HD34	LYRM4	LYR motif-containing protein 4	11	0.838	0	ND (active)	True	False	Active	–	
1908	IPI00177381	Q9HCG8	CWC22	Pre-mRNA-splicing factor CWC22 homolog	106	0.027	0	ND (active)	False	False	Active	–	
1705	IPI00032851	P61923	COPZ1	Coatomer subunit zeta-1	20	0.108	0	ND (active)	False	False	Active	–	
1968	IPI00746360	Q9NPQ8	RIC8A	Isoform 1 of Synembryn-A	60	0.204	0	ND (active)	True	False	Active	–	
859	IPI00032139	P50453	SERPINB9	Serpin B9	42	0.759	0	ND (active)	True	False	Active	–	
979	IPI00297851	O14646	CHD1	Isoform 1 of Chromodomain-helicase-DNA-binding protein 1	197	0.093	0	ND (active)	True	False	Active	–	
1919	IPI00028493	P49815	TSC2	Isoform 1 of Tuberin	201	0.010	0	ND (active)	False	False	Active	–	
1813	IPI00554521	P02794	FTH1	Ferritin heavy chain	21	0.322	0	ND (active)	True	False	Active	–	
1817	IPI00032439	Q9Y2S0	POLR1D	Isoform 1 of DNA-directed RNA polymerases I and III subunit RPAC2	15	0.096	0	ND (active)	False	False	Active	–	
1342	IPI00879210	Q9Y5Q8	GTF3C5	Isoform 3 of General transcription factor 3C polypeptide 5	60	0.229	0	ND (active)	True	False	Active	–	
1027	IPI00033153	Q9UBU9	NXF1	Nuclear RNA export factor 1	70	0.287	0	ND (active)	True	False	Active	–	
2201	IPI00159899	Q9P2R3	ANKFY1	Ankyrin repeat and FYVE domain-containing protein 1	133	0.016	0	ND (active)	False	False	Active	–	
1622	IPI00383163	Q9P016	THYN1	Isoform 1 of Thymocyte nuclear protein 1	26	0.166	0	ND (active)	True	False	Active	–	
1124	IPI00879950			15 kDa protein	15	0.168	0	ND (active)	False	False	Active	–	
2224	IPI00642318	Q8TCF1	ZFAND1	Isoform 2 of AN1-type zinc finger protein 1	18	0.080	0	ND (active)	False	False	Active	–	
2198	IPI00220710	Q9Y305	ACOT9	Isoform 1 of Acyl-coenzyme A thioesterase 9, mitochondrial	50	0.050	0	ND (active)	True	False	Active	–	
1886	IPI00026940	Q9UKX7	NUP50	Nuclear pore complex protein Nup50	50	0.065	0	ND (active)	True	False	Active	–	
1111	IPI00016621	O94973	AP2A2	Isoform 2 of AP-2 complex subunit alpha-2	104	0.572	0	ND (active)	True	False	Active	–	
2178	IPI00019599	Q13404	UBE2V1	Isoform 1 of Ubiquitin-conjugating enzyme E2 variant 1	26	0.155	0	ND (active)	True	False	Active	–	
1688	IPI00010414	O00151	PDLIM1	PDZ and LIM domain protein 1	36	0.154	0	ND (active)	True	False	Active	–	
1712	IPI00171412	Q8NBJ7	SUMF2	sulfatase modifying factor 2 isoform b precursor	36	0.240	0	ND (active)	True	False	Active	–	
1888	IPI00005055	Q9H5V9	CXorf56	UPF0428 protein CXorf56	26	0.097	0	ND (active)	False	False	Active	–	
1575	IPI00056499	Q969S3	ZNF622	Zinc finger protein 622	54	0.080	0	ND (active)	True	False	Active	–	
2104	IPI00873810	P62942	FKBP1A	Peptidyl-prolyl cis-trans isomerase FKBP1A	12	0.168	0	ND (active)	False	False	Active	–	
2161	IPI00009301	Q9H9Q2	COPS7B	Isoform 1 of COP9 signalosome complex subunit 7b	30	0.084	0	ND (active)	False	False	Active	–	
2010	IPI00034069	Q9H5Q4	TFB2M	Dimethyladenosine transferase 2, mitochondrial	45	0.080	0	ND (active)	True	False	Active	–	
2026	IPI00300567	P42126	DCI	Isoform 1 of 3,2-trans-enoyl-CoA isomerase, mitochondrial	33	0.168	0	ND (active)	True	False	Active	–	
2118	IPI00795627	P09001	MRPL3	Mitochondrial 39S ribosomal protein L3	42	0.084	0	ND (active)	True	False	Active	–	
2063	IPI00015956	Q9NQT5	EXOSC3	Exosome complex exonuclease RRP40	30	0.096	0	ND (active)	True	False	Active	–	
1489	IPI00024163	O14802	POLR3A	DNA-directed RNA polymerase III subunit RPC1	156	0.035	0	ND (active)	True	False	Active	–	
1992	IPI00297635	Q53FZ2	ACSM3	Isoform 1 of Acyl-coenzyme A synthetase ACSM3, mitochondrial	66	0.039	0	ND (active)	False	False	Active	–	
1413	IPI00029513	Q9BSC4	NOL10	Isoform 1 of Nucleolar protein 10	80	0.090	0	ND (active)	True	False	Active	–	
1450	IPI00219800	O95819	MAP4K4	Isoform 3 of Mitogen-activated protein kinase kinase kinase kinase 4	151	0.019	0	ND (active)	True	False	Active	–	
2053	IPI00329625	Q969Z0	TBRG4	Transforming growth factor beta regulator 4 (TBRG4), transcript variant 1	72	0.040	0	ND (active)	True	False	Active	–	
1625	IPI00479143	O95613	PCNT	Pericentrin	378	0.009	0	ND (active)	True	False	Active	–	
1144	IPI00306642	Q9NV06	DCAF13	WD repeats and SOF1 domain containing	68	0.217	0	ND (active)	True	False	Active	–	
816	IPI00306723	Q03701	CEBPZ	CCAAT/enhancer-binding protein zeta	121	0.208	0	ND (active)	True	False	Active	–	
2259	IPI00552191	Q5HYL3	PSMD13	Putative uncharacterized protein DKFZp686H16220	8	0.180	0	ND (active)	False	False	Active	–	
1804	IPI00183002	O14974	PPP1R12A	Isoform 1 of Protein phosphatase 1 regulatory subunit 12A	115	0.044	0	ND (active)	True	False	Active	–	
1677	IPI00748490	Q9BTE6	AARSD1	alanyl-tRNA synthetase domain containing 1 isoform 1	66	0.114	0	ND (active)	True	False	Active	–	
1197	IPI00025874	P04843	RPN1	Dolichyl-diphosphooligosaccharide--protein glycosyltransferase subunit 1 precursor	73	0.218	0	ND (active)	True	False	Active	–	
2252	IPI00328285	Q9Y2K6	USP20	Ubiquitin carboxyl-terminal hydrolase 20	102	0.030	0	ND (active)	False	False	Active	–	
2164	IPI00006378	Q9Y2S6	CCDC72	Coiled-coil domain-containing protein 72	7	0.298	0	ND (active)	False	False	Active	–	
1884	IPI00925578	Q0VDD8	DNAH14	Isoform 4 of Dynein heavy chain 14, axonemal	519	0.003	0	ND (active)	False	False	Active	–	
1426	IPI00015808	Q13823	GNL2	Nucleolar GTP-binding protein 2	84	0.108	0	ND (active)	True	False	Active	–	
2186	IPI00014513	P25490	YY1	Transcriptional repressor protein YY1	45	0.032	0	ND (active)	False	False	Active	–	
2234	IPI00031508	Q14562	DHX8	ATP-dependent RNA helicase DHX8	139	0.080	0	ND (active)	True	False	Active	–	
2191	IPI00166749	Q10713	PMPCA	Mitochondrial-processing peptidase subunit alpha	58	0.043	0	ND (active)	False	False	Active	–	
2220	IPI00216999	Q86U38	C14orf21	Pumilio domain-containing protein C14orf21	69	0.046	0	ND (active)	False	False	Active	–	
1978	IPI00007058	Q9BR76	CORO1B	Coronin-1B	54	0.075	0	ND (active)	True	False	Active	YES	
2048	IPI00024282	Q92930	RAB8B	Ras-related protein Rab-8B	24	0.270	0	ND (active)	False	False	Active	–	
2101	IPI00796856	Q99836	MYD88	Myeloid differentiation primary response protein MyD88	34	0.059	0	ND (active)	False	False	Active	–	
1502	IPI00027378	Q04323	UBXN1	Isoform 1 of UBX domain-containing protein 1	33	0.351	0	ND (active)	True	False	Active	–	
1869	IPI00026111	Q9UM00	TMCO1	Isoform 1 of Transmembrane and coiled-coil domain-containing protein 1	21	0.123	0	ND (active)	False	False	Active	–	
1428	IPI00604664	P28331	NDUFS1	NADH-ubiquinone oxidoreductase 75 kDa subunit	81	0.131	0	ND (active)	True	False	Active	–	
2129	IPI00216969	P00519	ABL1	Isoform IA of Tyrosine-protein kinase ABL1	123	0.025	0	ND (active)	False	False	Active	YES	
1339	IPI00554811	P59998	ARPC4	Actin-related protein 2/3 complex subunit 4	20	0.648	0	ND (active)	True	False	Active	YES*	
2193	IPI00549381	Q9BYD6	MRPL1	39S ribosomal protein L1, mitochondrial	37	0.082	0	ND (active)	False	False	Active	–	
1700	IPI00291467	P12236	SLC25A6	ADP/ATP translocase 3	33	1.375	0	ND (active)	True	False	Active	–	
1702	IPI00026942	O94905	ERLIN2	Isoform 1 of Erlin-2	38	0.186	0	ND (active)	True	False	Active	–	

Supplementary Table 1 (cont.) | Hierarchical clustering analysis of proteins identified in active or inactive integrin adhesion complexes.

#	IPI Accession	UniProtKB Accession	Gene name	Protein name	Protein MW (kDa)	Relative abundance (mean normalised spectral count) ×10^{3}		Fold enrichment (active/inactive integrin)[1]	Specific enrichment[2]		Cluster[3]	Adhesome[4]	Hierarchical clustering
						Active integrin	Inactive integrin		Active integrin	Inactive integrin			
1162	IPI00032957	P63279	UBE2I	SUMO-conjugating enzyme UBC9	18	0.632	0	ND (active)	True	False	Active	–	
1517	IPI00305692	O43396	TXNL1	Thioredoxin-like protein 1	32	0.212	0	ND (active)	True	False	Active	–	
1935	IPI00854619	Q9NSI8	SAMSN1	Putative uncharacterized protein SAMSN1	50	0.108	0	ND (active)	False	False	Active	–	
1767	IPI00844014	Q5T280	C9orf114	Uncharacterized protein C9orf114	42	0.137	0	ND (active)	True	False	Active	–	
1779	IPI00514832	Q9BRZ2	TRIM56	Isoform 1 of Tripartite motif-containing protein 56	81	0.018	0	ND (active)	False	False	Active	–	
1829	IPI00000155	Q49A26	GLYR1	Isoform 1 of Putative oxidoreductase GLYR1	61	0.083	0	ND (active)	True	False	Active	–	
2066	IPI00101659	Q96P11	NSUN5	Isoform 2 of Putative methyltransferase NSUN5	50	0.058	0	ND (active)	False	False	Active	–	
2105	IPI00021016	P43897	TSFM	Isoform 1 of Elongation factor Ts, mitochondrial	35	0.093	0	ND (active)	True	False	Active	–	
2215	IPI00384122	Q86TW7	DLST	Dihydrolipoyllysine-residue succinyltransferase component of 2- oxoglutarate dehydrogenase complex, mitochondrial	40	0.063	0	ND (active)	False	False	Active	–	
2244	IPI00031106	Q9BT73	PSMG3	Proteasome assembly chaperone 3	13	0.194	0	ND (active)	True	False	Active	–	
1959	IPI00419266	P56556	NDUFA6	NADH dehydrogenase (ubiquinone) 1 alpha subcomplex, 6	18	0.316	0	ND (active)	False	False	Active	–	
1984	IPI00220317	P09884	POLA1	DNA polymerase alpha catalytic subunit	166	0.015	0	ND (active)	True	False	Active	–	
1298	IPI00027898	Q9NSI2	C21orf70	Isoform A of Uncharacterized protein C21orf70	25	0.432	0	ND (active)	True	False	Active	–	
997	IPI00015838	Q9NX58	LYAR	Cell growth-regulating nucleolar protein	44	0.326	0	ND (active)	True	False	Active	–	
1004	IPI00023860	P55209	NAP1L1	Nucleosome assembly protein 1-like 1	45	0.360	0	ND (active)	True	False	Active	–	
2175	IPI00007812	P21281	ATP6V1B2	V-type proton ATPase subunit B, brain isoform	57	0.044	0	ND (active)	False	False	Active	–	
965	IPI00099996	Q7L0Y3	RG9MTD1	Mitochondrial ribonuclease P protein 1	47	0.423	0	ND (active)	True	False	Active	–	
1217	IPI00185146	Q96P70	IPO9	Importin-9	116	0.099	0	ND (active)	True	False	Active	–	
1761	IPI00006606	Q9BYN8	MRPS26	28S ribosomal protein S26, mitochondrial	24	0.282	0	ND (active)	True	False	Active	–	
1880	IPI00011118	P31350	RRM2	ribonucleotide reductase M2 polypeptide isoform 1	51	0.057	0	ND (active)	True	False	Active	–	
970	IPI00293078	Q96GQ7	DDX27	Probable ATP-dependent RNA helicase DDX27	90	0.233	0	ND (active)	True	False	Active	–	
2212	IPI00431082	Q96CS2	HAUS1	Isoform 1 of HAUS augmin-like complex subunit 1	32	0.085	0	ND (active)	False	False	Active	–	
1971	IPI00217104	Q96MG7	NDNL2	Melanoma-associated antigen G1	34	0.085	0	ND (active)	True	False	Active	–	
1615	IPI00009726	Q9HAN9	NMNAT1	Nicotinamide mononucleotide adenylyltransferase 1	32	0.257	0	ND (active)	True	False	Active	–	
2180	IPI00219538	P06746	POLB	DNA polymerase beta	38	0.076	0	ND (active)	True	False	Active	–	
2221	IPI00024567	Q9NZM5	GLTSCR2	Glioma tumor suppressor candidate region gene 2 protein	54	0.027	0	ND (active)	False	False	Active	–	
1933	IPI00160421	Q9H0U6	MRPL18	39S ribosomal protein L18, mitochondrial	21	0.264	0	ND (active)	True	False	Active	–	
1423	IPI00005705	P36873	PPP1CC	Isoform Gamma-1 of Serine/threonine-protein phosphatase PP1-gamma catalytic subunit	37	0.458	0	ND (active)	False	False	Active	–	
1414	IPI00413100	Q86UU1	PHLDB1	Isoform 1 of Pleckstrin homology-like domain family B member 1	151	0.097	0	ND (active)	True	False	Active	–	
2200	IPI00401962	Q6PK04	CCDC137	Coiled-coil domain-containing protein 137	33	0.044	0	ND (active)	False	False	Active	–	
1656	IPI00005740	O43402	COX4NB	Neighbor of COX4	24	0.234	0	ND (active)	True	False	Active	–	
1669	IPI00306400	Q9BW19	KIFC1	Kinesin-like protein KIFC1	74	0.073	0	ND (active)	True	False	Active	–	
1946	IPI00642862	Q8WUA2	PPIL4	Peptidyl-prolyl cis-trans isomerase-like 4	57	0.076	0	ND (active)	True	False	Active	–	
1972	IPI00329594	Q9P031	CCDC59	Thyroid transcription factor 1-associated protein 26	29	0.112	0	ND (active)	True	False	Active	–	
1879	IPI00329692	P30419	NMT1	Isoform Long of Glycylpeptide N-tetradecanoyltransferase 1	57	0.102	0	ND (active)	True	False	Active	–	
1990	IPI00304803	P36959	GMPR	GMP reductase 1	37	0.054	0	ND (active)	False	False	Active	–	
2043	IPI00945795	P43307	SSR1	Putative uncharacterized protein SSR1	30	0.185	0	ND (active)	False	False	Active	–	
1122	IPI00219871	O95777	LSM8	U6 snRNA-associated Sm-like protein LSm8	10	1.447	0	ND (active)	True	False	Active	–	
1701	IPI00008437	Q9UHA3	RSL24D1	Probable ribosome biogenesis protein RLP24	20	0.220	0	ND (active)	True	False	Active	–	
1029	IPI00019196	P78346	RPP30	Ribonuclease P protein subunit p30	29	0.522	0	ND (active)	True	False	Active	–	
1249	IPI00303099	Q53HL2	CDCA8	Borealin	31	0.555	0	ND (active)	True	False	Active	–	
1945	IPI00166422	Q6NZY4	ZCCHC8	Isoform 2 of Zinc finger CCHC domain-containing protein 8	51	0.071	0	ND (active)	True	False	Active	–	
973	IPI00791325	Q15397	KIAA0020	Pumilio domain-containing protein KIAA0020	77	0.215	0	ND (active)	True	False	Active	–	
2190	IPI00872759	O14686	MLL2	564 kDa protein	564	0.003	0	ND (active)	False	False	Active	–	
1909	IPI00008728	O76031	CLPX	ATP-dependent Clp protease ATP-binding subunit clpX-like, mitochondrial	69	0.059	0	ND (active)	True	False	Active	–	
1616	IPI00294575	P30260	CDC27	Cell division cycle protein 27 homolog	92	0.043	0	ND (active)	True	False	Active	–	
2206	IPI00290514	Q9NV88	INTS9	Integrator complex subunit 9	74	0.019	0	ND (active)	False	False	Active	–	
2214	IPI00328257	Q10567	AP1B1	Isoform A of AP-1 complex subunit beta-1	105	0.456	0	ND (active)	True	False	Active	–	
1708	IPI00642549	Q5W0S5	RAD23B	UV excision repair protein RAD23 homolog B	42	0.111	0	ND (active)	True	False	Active	–	
1786	IPI00028091	P61158	ACTR3	Actin-related protein 3	47	0.139	0	ND (active)	True	False	Active	–	
1526	IPI00023126	O15213	WDR46	WD repeat-containing protein 46	68	0.053	0	ND (active)	True	False	Active	–	
1349	IPI00012462	Q9BY44	EIF2A	Eukaryotic translation initiation factor 2A	68	0.123	0	ND (active)	True	False	Active	–	
2177	IPI00010653	Q9GZR2	REXO4	Isoform 1 of RNA exonuclease 4	47	0.054	0	ND (active)	True	False	Active	–	
1737	IPI00000296	Q8WUW1	C3orf10	Isoform 1 of Probable protein BRICK1	9	0.392	0	ND (active)	True	False	Active	–	
1845	IPI00008248	Q9UJX3	ANAPC7	anaphase-promoting complex subunit 7 isoform a	67	0.104	0	ND (active)	True	False	Active	–	
1944	IPI00106502	Q14145	KEAP1	Kelch-like ECH-associated protein 1	70	0.072	0	ND (active)	True	False	Active	YES	
1994	IPI00219005	Q02790	FKBP4	Peptidyl-prolyl cis-trans isomerase FKBP4	52	0.039	0	ND (active)	False	False	Active	–	
1527	IPI00027438	O75955	FLOT1	Flotillin-1	47	0.268	0	ND (active)	True	False	Active	–	
1976	IPI00010845	O00217	NDUFS8	NADH dehydrogenase [ubiquinone] iron-sulfur protein 8, mitochondrial	24	0.168	0	ND (active)	True	False	Active	–	
1471	IPI00553153	Q9UII2	ATPIF1	Putative uncharacterized protein DKFZp564G0422	12	0.756	0	ND (active)	True	False	Active	–	
1741	IPI00002255	P50851	LRBA	Isoform 1 of Lipopolysaccharide-responsive and beige-like anchor protein	319	0.021	0	ND (active)	True	False	Active	–	
1446	IPI00023591	Q00577	PURA	Transcriptional activator protein Pur-alpha	35	0.253	0	ND (active)	True	False	Active	–	
1738	IPI00022403	Q9BYD1	MRPL13	39S ribosomal protein L13, mitochondrial	21	0.312	0	ND (active)	True	False	Active	–	
1573	IPI00030255	O60568	PLOD3	Procollagen-lysine,2-oxoglutarate 5-dioxygenase 3	85	0.073	0	ND (active)	True	False	Active	–	
1652	IPI00032799			37 kDa protein	37	0.185	0	ND (active)	True	False	Active	–	
1736	IPI00328170	Q13724	MOGS	Mannosyl-oligosaccharide glucosidase	92	0.035	0	ND (active)	True	False	Active	–	
1282	IPI00166865	Q8N5K1	CISD2	CDGSH iron sulfur domain-containing protein 2	15	0.778	0	ND (active)	True	False	Active	–	
989	IPI00029159	P49959	MRE11A	DOUBLE-STRAND BREAK REPAIR PROTEIN MRE11A	81	0.286	0	ND (active)	True	False	Active	–	
2231	IPI00010244	P82912	MRPS11	Isoform 1 of 28S ribosomal protein S11, mitochondrial	21	0.168	0	ND (active)	False	False	Active	–	
2167	IPI00022479	Q15751	HERC1	Putative uncharacterized protein HERC1	532	0.005	0	ND (active)	False	False	Active	–	
1900	IPI00168165	O75122	CLASP2	CLASP2 protein	166	0.038	0	ND (active)	True	False	Active	–	
1902	IPI00005657	O15212	PFDN6	Prefoldin subunit 6	15	0.120	0	ND (active)	False	False	Active	–	
1347	IPI00023640	O14737	PDCD5	Programmed cell death protein 5	14	1.162	0	ND (active)	True	False	Active	–	
2183	IPI00063827	Q96IU4	ABHD14B	Isoform 1 of Abhydrolase domain-containing protein 14B	22	0.098	0	ND (active)	False	False	Active	–	
2228	IPI00032911	P62380	TBPL1	TATA box-binding protein-like protein 1	21	0.120	0	ND (active)	True	False	Active	–	
1851	IPI00306719	Q9BWS9	CHID1	cDNA FLJ55809	51	0.064	0	ND (active)	True	False	Active	–	
1870	IPI00301364	P63208	SKP1	Isoform 1 of S-phase kinase-associated protein 1	19	0.209	0	ND (active)	True	False	Active	–	
2057	IPI00411816	Q5T653	MRPL2	39S ribosomal protein L2, mitochondrial	33	0.107	0	ND (active)	True	False	Active	–	
2100	IPI00291328	P19404	NDUFV2	NADH dehydrogenase [ubiquinone] flavoprotein 2, mitochondrial	27	0.080	0	ND (active)	False	False	Active	–	
2181	IPI00306446	P17028	ZNF24	Zinc finger protein 24	42	0.034	0	ND (active)	False	False	Active	–	
1199	IPI00294211	Q92620	DHX38	Pre-mRNA-splicing factor ATP-dependent RNA helicase PRP16	141	0.061	0	ND (active)	True	False	Active	–	
1141	IPI00087498	Q56NI9	ESCO2	N-acetyltransferase ESCO2	68	0.042	0	ND (active)	False	False	Active	–	
1601	IPI00413451	P35237	SERPINB6	Serpin B6	46	0.192	0	ND (active)	True	False	Active	–	
1733	IPI00783302	Q96EY7	PTCD3	Isoform 1 of Pentatricopeptide repeat-containing protein 3, mitochondrial	79	0.071	0	ND (active)	True	False	Active	–	
1907	IPI00795263	Q96DA6	DNAJC19	Putative uncharacterized protein DNAJC19	10	0.216	0	ND (active)	False	False	Active	–	
1918	IPI00012578	O00629	KPNA4	Importin subunit alpha-4	58	0.056	0	ND (active)	False	False	Active	–	
1947	IPI00218463	Q9Y3D7	TIMM16	Mitochondrial import inner membrane translocase subunit Tim16	14	0.360	0	ND (active)	True	False	Active	–	
1952	IPI00410717	Q7Z3K3	POGZ	Isoform 1 of Pogo transposable element with ZNF domain	155	0.033	0	ND (active)	True	False	Active	–	
1655	IPI00816474	A6ZKI3	FAM127A	Protein FAM127A	13	0.255	0	ND (active)	True	False	Active	–	
1890	IPI00298828	P02749	APOH	Beta-2-glycoprotein 1	38	0.093	0	ND (active)	False	False	Active	–	
2009	IPI00436705	Q4VBZ9	MORC3	MORC family CW-type zinc finger 3	107	0.020	0	ND (active)	False	False	Active	–	
2127	IPI00219291	P56134	ATP5J2	Putative uncharacterized protein ATP5J2	11	0.131	0	ND (active)	False	False	Active	–	
1177	IPI00007729	Q9UMY1	NOL7	Isoform 1 of Nucleolar protein 7	29	0.472	0	ND (active)	True	False	Active	–	
2165	IPI00550995	Q9NWS0	PIH1D1	PIH1 domain-containing protein 1	32	0.063	0	ND (active)	False	False	Active	–	
1143	IPI00000948	Q9Y4P3	TBL2	Transducin beta-like protein 2	50	0.386	0	ND (active)	True	False	Active	–	
2162	IPI00026182	P47755	CAPZA2	F-actin-capping protein subunit alpha-2	33	0.229	0	ND (active)	True	False	Active	–	
1110	IPI00470596	Q14966	ZNF638	Isoform 1 of Zinc finger protein 638	221	0.053	0	ND (active)	True	False	Active	–	
1196	IPI00217626	Q8IXT5	RBM12B	Putative uncharacterized protein RBM12B	103	0.103	0	ND (active)	True	False	Active	–	
2163	IPI00878649			113 kDa protein	113	0.013	0	ND (active)	False	False	Active	–	
1892	IPI00018240	Q9NVU7	SDAD1	Isoform 1 of Protein SDA1 homolog	80	0.068	0	ND (active)	True	False	Active	–	
1137	IPI00895860	Q14676	MDC1	Isoform 3 of Mediator of DNA damage checkpoint protein 1	196	0.046	0	ND (active)	True	False	Active	–	
2168	IPI00216219	Q07157	TJP1	Isoform Long of Tight junction protein ZO-1	195	0.009	0	ND (active)	False	False	Active	–	
2090	IPI00062599	Q96GM5	SMARCD1	71 kDa protein	71	0.061	0	ND (active)	True	False	Active	–	
2031	IPI00294744	O95453	PARN	Poly(A)-specific ribonuclease PARN	73	0.054	0	ND (active)	True	False	Active	–	
2176	IPI00024971	P22059	OSBP	Isoform 1 of Oxysterol-binding protein 1	89	0.040	0	ND (active)	True	False	Active	–	
1985	IPI00024255	Q92917	GPKOW	G patch domain and KOW motifs-containing protein	52	0.076	0	ND (active)	True	False	Active	–	
1699	IPI00552920	Q96B26	EXOSC8	Exosome complex exonuclease RRP43	30	0.144	0	ND (active)	True	False	Active	–	
1998	IPI00037599	Q12800	TFCP2	Isoform 1 of Alpha-globin transcription factor CP2	57	0.037	0	ND (active)	False	False	Active	–	
1973	IPI00019195	P78345	RPP38	Ribonuclease P protein subunit p38	32	0.113	0	ND (active)	True	False	Active	–	
2099	IPI00171252	Q15910	EZH2	enhancer of zeste 2 isoform b	81	0.022	0	ND (active)	False	False	Active	–	
2074	IPI00030247	O60563	CCNT1	Cyclin-T1	81	0.053	0	ND (active)	False	False	Active	–	
1577	IPI00186194	Q5SW79	CEP170	Isoform 1 of Centrosomal protein of 170 kDa	175	0.049	0	ND (active)	True	False	Active	–	
1722	IPI00183938	Q6P3X3	TTC27	Tetratricopeptide repeat protein 27	97	0.061	0	ND (active)	False	False	Active	–	
1259	IPI00329038	Q96SN8	CDK5RAP2	Isoform 1 of CDK5 regulatory subunit-associated protein 2	215	0.014	0	ND (active)	False	False	Active	–	
1707	IPI00006164	Q9H0C8	ILKAP	Integrin-linked kinase-associated serine/threonine phosphatase 2C	43	0.161	0	ND (active)	True	False	Active	YES	
1912	IPI00103483	Q8WX92	COBRA1	Negative elongation factor B	66	0.060	0	ND (active)	True	False	Active	–	
1156	IPI00305668	P82932	MRPS6	28S ribosomal protein S6, mitochondrial	14	0.869	0	ND (active)	True	False	Active	–	
1332	IPI00217630	Q8IY37	DHX37	Probable ATP-dependent RNA helicase DHX37	130	0.083	0	ND (active)	True	False	Active	–	
2087	IPI00025849	P39687	ANP32A	Acidic leucine-rich nuclear phosphoprotein 32 family member A	29	0.224	0	ND (active)	True	False	Active	–	
2024	IPI00009316	Q9UNP9	PPIE	Isoform A of Peptidyl-prolyl cis-trans isomerase E	33	0.164	0	ND (active)	True	False	Active	–	
1695	IPI00166555	Q5T8P6	RBM26	114 kDa protein	114	0.028	0	ND (active)	False	False	Active	–	
1714	IPI00297322	Q8N6H7	ARFGAP2	ADP-ribosylation factor GTPase-activating protein 2	57	0.076	0	ND (active)	True	False	Active	–	
1188	IPI00180454	Q9Y3A2	UTP11L	Probable U3 small nucleolar RNA-associated protein 11	30	0.456	0	ND (active)	True	False	Active	–	
2187	IPI00299085	O60732	MAGEC1	Melanoma-associated antigen C1	124	0.014	0	ND (active)	False	False	Active	–	
2006	IPI00002408	Q9H6T3	RPAP3	Isoform 1 of RNA polymerase II-associated protein 3	76	0.028	0	ND (active)	False	False	Active	–	
1645	IPI00427242	Q9Y324	FCF1	rRNA-processing protein FCF1 homolog	23	0.313	0	ND (active)	True	False	Active	–	
1795	IPI00015143	O43913	ORC5L	Origin recognition complex subunit 5	50	0.137	0	ND (active)	True	False	Active	–	
1600	IPI00014812	P82921	MRPS21	28S ribosomal protein S21, mitochondrial	11	0.779	0	ND (active)	True	False	Active	–	
2153	IPI00018671	P51452	DUSP3	Dual specificity protein phosphatase 3	20	0.180	0	ND (active)	True	False	Active	–	
2156	IPI00028387	Q96HY6	DDRGK1	Isoform 1 of DDRGK domain-containing protein 1	36	0.078	0	ND (active)	False	False	Active	–	
2003	IPI00015806	Q9Y5Q9	GTF3C3	Isoform 1 of General transcription factor 3C polypeptide 3	101	0.038	0	ND (active)	False	False	Active	–	
2091	IPI00025174	Q6UX04	CWC27	Isoform 1 of Peptidyl-prolyl cis-trans isomerase SDCCAG10	54	0.033	0	ND (active)	False	False	Active	–	
2134	IPI00005745	O15269	SPTLC1	Serine palmitoyltransferase 1	53	0.048	0	ND (active)	False	False	Active	YES	
1388	IPI00795394	Q9NYC9	DNAH9	Isoform 2 of Dynein heavy chain 9, axonemal	503	0.004	0	ND (active)	False	False	Active	–	
1915	IPI00021320	Q7L2J0	MEPCE	7SK snRNA methylphosphate capping enzyme	74	0.024	0	ND (active)	False	False	Active	–	
1596	IPI00844214	Q8WYA6	CTNNBL1	Isoform 1 of Beta-catenin-like protein 1	65	0.095	0	ND (active)	True	False	Active	–	
1672	IPI00216192	Q9H078	CLPB	Isoform 2 of Caseinolytic peptidase B protein homolog	75	0.062	0	ND (active)	True	False	Active	–	
1487	IPI00828098	Q9HCF4	KIAA1618	ring finger protein 213 isoform 1	596	0.011	0	ND (active)	True	False	Active	–	
1186	IPI00217862	O43818	RRP9	U3 small nucleolar RNA-interacting protein 2	52	0.296	0	ND (active)	True	False	Active	–	
2130	IPI00306048	Q5T9A4	ATAD3B	Isoform 1 of ATPase family AAA domain-containing protein 3B	73	0.296	0	ND (active)	False	False	Active	–	
1823	IPI00413265	Q8IY18	SMC5	Structural maintenance of chromosomes protein 5	129	0.034	0	ND (active)	True	False	Active	–	
1960	IPI00028412	O60232	SSSCA1	Sjoegren syndrome/scleroderma autoantigen 1	21	0.096	0	ND (active)	False	False	Active	–	
2144	IPI00418262	P09972	ALDOC	Fructose-bisphosphate aldolase	48	0.105	0	ND (active)	False	False	Active	–	
2185	IPI00032387	Q14691	GINS1	DNA replication complex GINS protein PSF1	23	0.110	0	ND (active)	True	False	Active	–	
1232	IPI00290461	O75822	EIF3J	Eukaryotic translation initiation factor 3 subunit J	29	0.358	0	ND (active)	True	False	Active	–	
1742	IPI00012341	Q13243	SFRS5	Isoform SRP40-1 of Splicing factor, arginine/serine-rich 5	31	1.011	0	ND (active)	True	False	Active	–	
1219	IPI00297900	Q13206	DDX10	Probable ATP-dependent RNA helicase DDX10	101	0.136	0	ND (active)	True	False	Active	–	
2120	IPI00303292	P52294	KPNA1	Importin subunit alpha-1	60	0.036	0	ND (active)	False	False	Active	–	
2054	IPI00022002	Q92552	MRPS27	Mitochondrial 28S ribosomal protein S27	49	0.041	0	ND (active)	False	False	Active	–	
1675	IPI00375731	P98175	RBM10	Putative uncharacterized protein DKFZp686E2459	110	0.046	0	ND (active)	True	False	Active	–	
1964	IPI00792072	Q59GE1	SEPT5-	H5 variant	44	0.105	0	ND (active)	True	False	Active	–	
1650	IPI00031615	Q9BRT6	LLPH	Protein LLP homolog	15	0.360	0	ND (active)	True	False	Active	–	
1943	IPI00019463	P19525	EIF2AK2	Interferon-induced, double-stranded RNA-activated protein kinase	62	0.076	0	ND (active)	True	False	Active	–	
2017	IPI00216338	P83876	TXNL4A	Thioredoxin-like protein 4A	17	0.191	0	ND (active)	True	False	Active	–	
1460	IPI00219841	P18858	LIG1	DNA ligase 1	102	0.047	0	ND (active)	True	False	Active	–	
2125	IPI00329598	Q8NBQ5	HSD17B11	Estradiol 17-beta-dehydrogenase 11	33	0.120	0	ND (active)	True	False	Active	–	
1631	IPI00023097	Q86U86	PBRM1	Isoform 5 of Protein polybromo-1	182	0.027	0	ND (active)	True	False	Active	–	
1272	IPI00641990	Q9Y4W2	LAS1L	Isoform 1 of Protein LAS1 homolog	83	0.126	0	ND (active)	True	False	Active	–	
1727	IPI00300504	Q9HAU5	UPF2	Isoform 1 of Regulator of nonsense transcripts 2	148	0.046	0	ND (active)	True	False	Active	–	
1704	IPI00294742	Q4G0J3	LARP7	La-related protein 7	68	0.100	0	ND (active)	True	False	Active	–	
1965	IPI00219287	P10155	TROVE2	Isoform Short of 60 kDa SS-A/Ro ribonucleoprotein	23	0.172	0	ND (active)	False	False	Active	–	
2123	IPI00291131	Q6I9Y2	THOC7	THO complex subunit 7 homolog	24	0.135	0	ND (active)	True	False	Active	–	
1623	IPI00011250	P15374	UCHL3	Ubiquitin carboxyl-terminal hydrolase isozyme L3	26	0.305	0	ND (active)	True	False	Active	–	
1410	IPI00000051	O60925	PFDN1	Prefoldin subunit 1	14	0.669	0	ND (active)	True	False	Active	–	
2068	IPI00020495	P82909	MRPS36	28S ribosomal protein S36, mitochondrial	11	0.504	0	ND (active)	True	False	Active	–	

Supplementary Table 1 (cont.) | Hierarchical clustering analysis of proteins identified in active or inactive integrin adhesion complexes.

#	IPI Accession	UniProtKB Accession	Gene name	Protein name	Protein MW (kDa)	Relative abundance (mean normalised spectral count) ×10[3]: Active integrin	Relative abundance (mean normalised spectral count) ×10[3]: Inactive integrin	Fold enrichment (active/inactive integrin)[1]	Specific enrichment[2]: Active integrin	Specific enrichment[2]: Inactive integrin	Cluster[3]	Adhesome[4]	Hierarchical clustering
1646	IPI00292135	Q14739	LBR	Lamin-B receptor	71	0.057	0	ND (active)	True	False	Active	–	
2021	IPI00004488	Q16864	ATP6V1F	V-type proton ATPase subunit F	13	0.139	0	ND (active)	False	False	Active	–	
1597	IPI00026513	P49247	RPIA	Ribose-5-phosphate isomerase	33	0.282	0	ND (active)	True	False	Active	–	
1311	IPI00220059	O95168	NDUFB4	NADH dehydrogenase [ubiquinone] 1 beta subcomplex subunit 4	15	0.413	0	ND (active)	True	False	Active	–	
2034	IPI00018924	Q9NP66	HMG20A	Isoform 1 of High mobility group protein 20A	40	0.052	0	ND (active)	False	False	Active	–	
2030	IPI00006167	O15355	PPM1G	Protein phosphatase 1G	59	0.073	0	ND (active)	True	False	Active	–	
1648	IPI00006715	O60216	RAD21	Double-strand-break repair protein rad21 homolog	72	0.100	0	ND (active)	True	False	Active	–	
1995	IPI00031023	Q13045	FLII	Protein flightless-1 homolog	145	0.029	0	ND (active)	True	False	Active	–	
1958	IPI00171248	O75530	EED	Isoform 3 of Polycomb protein EED	46	0.063	0	ND (active)	True	False	Active	–	
1988	IPI00220361	P05937	CALB1	Calbindin	30	0.180	0	ND (active)	True	False	Active	–	
2082	IPI00452731	O95182	NDUFA7	NADH dehydrogenase [ubiquinone] 1 alpha subcomplex subunit 7	13	0.310	0	ND (active)	True	False	Active	–	
2174	IPI00003406	Q16643	DBN1	Isoform 1 of Drebrin	71	0.028	0	ND (active)	False	False	Active	–	
2055	IPI00456048	Q86X83	COMMD2	COMM domain-containing protein 2	23	0.125	0	ND (active)	True	False	Active	–	
2098	IPI00410657	O43148	RNMT	Isoform 2 of mRNA cap guanine-N7 methyltransferase	58	0.050	0	ND (active)	False	False	Active	–	
2116	IPI00446176	Q6ZVE6	PMF1	HCG1999356, isoform CRA_c	20	0.090	0	ND (active)	False	False	Active	–	
1620	IPI00759472	Q9NQS7	INCENP	Isoform 2 of Inner centromere protein	105	0.051	0	ND (active)	True	False	Active	–	
2112	IPI00021742	Q9UP22	PDC	phosducin isoform b	22	0.160	0	ND (active)	True	False	Active	–	
1594	IPI00004859	P54132	BLM	Bloom syndrome protein	159	0.029	0	ND (active)	True	False	Active	–	
1939	IPI00028122	O75475	PSIP1	Isoform 1 of PC4 and SFRS1-interacting protein	60	0.042	0	ND (active)	False	False	Active	–	
1986	IPI00016634	Q9NWU2	C20orf11	Protein C20orf11	27	0.187	0	ND (active)	True	False	Active	–	
2036	IPI00100791	Q13889	GTF2H3	General transcription factor IIH subunit 3	34	0.051	0	ND (active)	False	False	Active	–	
2067	IPI00305442	Q9BSL1	UBAC1	Ubiquitin-associated domain-containing protein 1	45	0.080	0	ND (active)	True	False	Active	–	
2173	IPI00169400	P82675	MRPS5	Isoform 1 of 28S ribosomal protein S5, mitochondrial	48	0.073	0	ND (active)	False	False	Active	–	
2154	IPI00396058	Q86XI2	NCAPG2	Isoform 2 of Condensin-2 complex subunit G2	132	0.022	0	ND (active)	True	False	Active	–	
1881	IPI00154528	Q96SB8	SMC6	Isoform 1 of Structural maintenance of chromosomes protein 6	126	0.026	0	ND (active)	True	False	Active	–	
2050	IPI00026519	P30405	PPIF	Peptidyl-prolyl cis-trans isomerase F, mitochondrial	22	0.160	0	ND (active)	False	False	Active	–	
1550	IPI00003168	O60256	PRPSAP2	Phosphoribosyl pyrophosphate synthase-associated protein 2	41	0.209	0	ND (active)	True	False	Active	–	
1366	IPI00305289	P52732	KIF11	Kinesin-like protein KIF11	119	0.087	0	ND (active)	True	False	Active	–	
1529	IPI00026606	O75683	SURF6	Surfeit locus protein 6	41	0.167	0	ND (active)	True	False	Active	–	
1602	IPI00747053	P19447	ERCC3	TFIIH basal transcription factor complex helicase XPB subunit	89	0.061	0	ND (active)	True	False	Active	–	
1273	IPI00218466	P61619	SEC61A1	Protein transport protein Sec61 subunit alpha isoform 1	53	0.156	0	ND (active)	True	False	Active	–	
1409	IPI00414836	Q92882	OSTF1	Osteoclast-stimulating factor 1	24	0.360	0	ND (active)	True	False	Active	YES	
2012	IPI00328390	Q86SE9	PCGF5	Isoform 1 of Polycomb group RING finger protein 5	30	0.108	0	ND (active)	True	False	Active	–	
1863	IPI00030383	Q12972	PPP1R8	Isoform Alpha of Nuclear inhibitor of protein phosphatase 1	38	0.106	0	ND (active)	True	False	Active	–	
2085	IPI00302238	Q9NY61	AATF	Protein AATF	63	0.051	0	ND (active)	True	False	Active	–	
2142	IPI00018452	Q99829	CPNE1	Copine-1	59	0.037	0	ND (active)	False	False	Active	–	
1407	IPI00005822	Q9UJX2	CDC23	Isoform 1 of Cell division cycle protein 23 homolog	69	0.142	0	ND (active)	True	False	Active	–	
2106	IPI00025285	O75348	ATP6V1G1	V-type proton ATPase subunit G 1	14	0.314	0	ND (active)	True	False	Active	–	
1442	IPI00022694	P55036	PSMD4	Isoform Rpn10A of 26S proteasome non-ATPase regulatory subunit 4	41	0.253	0	ND (active)	True	False	Active	–	
2139	IPI00183603	Q9NRP0	OSTC	Oligosaccharyltransferase complex subunit OSTC	17	0.119	0	ND (active)	False	False	Active	–	
1849	IPI00874020	Q9H0N5	PCBD2	Pterin-4-alpha-carbinolamine dehydratase 2	14	0.453	0	ND (active)	True	False	Active	–	
1619	IPI00024403	O75131	CPNE3	Copine-3	60	0.121	0	ND (active)	True	False	Active	–	
1932	IPI00016932	O15357	INPPL1	Isoform 1 of Phosphatidylinositol-3,4,5-trisphosphate 5-phosphatase 2	139	0.022	0	ND (active)	False	False	Active	YES	
2146	IPI00329528	Q9Y4B6	VPRBP	Isoform 1 of Protein VPRBP	169	0.017	0	ND (active)	True	False	Active	–	
2015	IPI00010438	O00161	SNAP23	Isoform SNAP-23a of Synaptosomal-associated protein 23	23	0.141	0	ND (active)	True	False	Active	–	
1970	IPI00027142	O75817	POP7	Ribonuclease P protein subunit p20	16	0.203	0	ND (active)	True	False	Active	–	
2094	IPI00220302	Q15007	WTAP	Isoform 1 of Pre-mRNA-splicing regulator WTAP	44	0.074	0	ND (active)	True	False	Active	–	
1504	IPI00026496	O75607	NPM3	Nucleoplasmin-3	19	0.133	0	ND (active)	False	False	Active	–	
1847	IPI00172591	Q9NRX2	MRPL17	39S ribosomal protein L17, mitochondrial	20	0.227	0	ND (active)	True	False	Active	–	
1447	IPI00550638	Q9GZS1	POLR1E	Isoform 2 of DNA-directed RNA polymerase I subunit RPA49	47	0.138	0	ND (active)	True	False	Active	–	
1424	IPI00216393	P09496	CLTA	Isoform Non-brain of Clathrin light chain A	24	0.534	0	ND (active)	True	False	Active	–	
1331	IPI00033025	Q16181	SEPT7	51 kDa protein	51	0.279	0	ND (active)	True	False	Active	–	
1479	IPI00292168	Q9Y294	ASF1A	Histone chaperone ASF1A	23	0.094	0	ND (active)	False	False	Active	–	
1949	IPI00293396	O43747	AP1G1	adaptor-related protein complex 1, gamma 1 subunit isoform a	92	0.060	0	ND (active)	True	False	Active	–	
1313	IPI00170596	Q96ST3	SIN3A	Paired amphipathic helix protein Sin3a	145	0.081	0	ND (active)	True	False	Active	–	
1670	IPI00375358	P35251	RFC1	Isoform 1 of Replication factor C subunit 1	128	0.034	0	ND (active)	True	False	Active	–	
1516	IPI00299635	Q9NR09	BIRC6	baculoviral IAP repeat-containing 6	530	0.010	0	ND (active)	True	False	Active	–	
2145	IPI00293126	Q99426	TBCB	Tubulin-folding cofactor B	27	0.053	0	ND (active)	False	False	Active	–	
2052	IPI00304409	Q9Y2V2	CARHSP1	Calcium-regulated heat stable protein 1	16	0.113	0	ND (active)	False	False	Active	–	
1812	IPI00004497	P11274	BCR	Isoform 1 of Breakpoint cluster region protein	143	0.022	0	ND (active)	True	False	Active	–	
1621	IPI00426640	Q13888	GTF2H2	General transcription factor IIH subunit 2	44	0.180	0	ND (active)	True	False	Active	–	
2007	IPI00217557	Q8IXM6	NRM	Isoform 1 of Nurim	29	0.147	0	ND (active)	True	False	Active	–	
2065	IPI00019495	P63172	DYNLT1	Dynein light chain Tctex-type 1	12	0.210	0	ND (active)	False	False	Active	–	
2119	IPI00025721	Q9UNS2	COPS3	COP9 signalosome complex subunit 3	48	0.030	0	ND (active)	False	False	Active	–	
1505	IPI00000162	Q8NEJ9	NGDN	Isoform 1 of Neuroguidin	36	0.282	0	ND (active)	True	False	Active	–	
1535	IPI00032206	P50479	PDLIM4	Isoform 1 of PDZ and LIM domain protein 4	35	0.177	0	ND (active)	True	False	Active	–	
1703	IPI00178861	Q9NXH9	TRMT1	Isoform 2 of N(2),N(2)-dimethylguanosine tRNA methyltransferase	69	0.107	0	ND (active)	True	False	Active	–	
1412	IPI00440122	Q6LAI4	MSH6	Sperm protein	37	0.080	0	ND (active)	True	False	Active	–	
2023	IPI00022613	P78316	NOP14	Isoform 1 of Nucleolar protein 14	98	0.026	0	ND (active)	False	False	Active	–	
1434	IPI00107113	Q9BVJ6	UTP14A	Isoform 1 of U3 small nucleolar RNA-associated protein 14 homolog A	88	0.098	0	ND (active)	True	False	Active	–	
2128	IPI00024551	Q9NPA0	C15orf24	UPF0480 protein C15orf24	26	0.055	0	ND (active)	False	False	Active	–	
1807	IPI00297593	Q70CQ2	USP34	Isoform 1 of Ubiquitin carboxyl-terminal hydrolase 34	404	0.009	0	ND (active)	False	False	Active	–	
1904	IPI00027193	Q9NZA1	CLIC5	Isoform 2 of Chloride intracellular channel protein 5	47	0.098	0	ND (active)	True	False	Active	–	
2108	IPI00001636	Q9UBB4	ATXN10	Ataxin-10	53	0.038	0	ND (active)	False	False	Active	–	
2133	IPI00099433	Q8WYB5	MYST4	Isoform 1 of Histone acetyltransferase MYST4	231	0.008	0	ND (active)	False	False	Active	–	
2013	IPI00020364	P78560	CRADD	Death domain-containing protein CRADD	23	0.094	0	ND (active)	False	False	Active	–	
2059	IPI00930346	Q9UBU8	MORF4L1	Isoform 2 of Mortality factor 4-like protein 1	37	0.095	0	ND (active)	True	False	Active	–	
1628	IPI00910781	P06744	GPI	Glucose-6-phosphate isomerase	64	0.106	0	ND (active)	True	False	Active	–	
2058	IPI00946908	Q14738	PPP2R5D	Serine/threonine-protein phosphatase 2A 56 kDa regulatory subunit delta isoform	69	0.037	0	ND (active)	True	False	Active	–	
1509	IPI00102815	Q8WTT2	NOC3L	Nucleolar complex protein 3 homolog	93	0.077	0	ND (active)	True	False	Active	–	
1477	IPI00032459	Q9Y2T2	AP3M1	AP-3 complex subunit mu-1	47	0.166	0	ND (active)	True	False	Active	–	
2092	IPI00031479	Q14554	PDIA5	Protein disulfide-isomerase A5	60	0.042	0	ND (active)	True	False	Active	–	
1690	IPI00011898	Q13144	EIF2B5	Translation initiation factor eIF-2B subunit epsilon	80	0.052	0	ND (active)	True	False	Active	–	
2093	IPI00102997	Q96S55	WRNIP1	Isoform 2 of ATPase WRNIP1	69	0.052	0	ND (active)	True	False	Active	–	
2025	IPI00946154	P09038	FGF2	Isoform 1 of Heparin-binding growth factor 2	31	0.046	0	ND (active)	False	False	Active	–	
2060	IPI00018813	P61201	COPS2	Isoform 2 of COP9 signalosome complex subunit 2	52	0.028	0	ND (active)	False	False	Active	–	
2132	IPI00185859	Q9BRJ2	MRPL45	39S ribosomal protein L45, mitochondrial	35	0.132	0	ND (active)	True	False	Active	–	
198	IPI00911039	P08107	HSPA1A	Heat shock 70 kDa protein 1	64	1.716	0	ND (active)	True	False	Active	–	
2077	IPI00418290	Q6P1L8	MRPL14	39S ribosomal protein L14, mitochondrial	16	0.261	0	ND (active)	True	False	Active	–	
1850	IPI00007024	Q9Y3D0	FAM96B	Protein FAM96B	18	0.160	0	ND (active)	False	False	Active	–	
2070	IPI00304885	Q03188	CENPC1	Centromere protein C 1	107	0.024	0	ND (active)	True	False	Active	–	
1484	IPI00059762	Q5VWZ2	LYPLAL1	Isoform 1 of Lysophospholipase-like protein 1	26	0.224	0	ND (active)	True	False	Active	–	
1844	IPI00412404	Q8IYB8	SUPV3L1	ATP-dependent RNA helicase SUPV3L1, mitochondrial	88	0.070	0	ND (active)	True	False	Active	–	
1522	IPI00100160	Q86VP6	CAND1	Isoform 1 of Cullin-associated NEDD8-dissociated protein 1	136	0.067	0	ND (active)	True	False	Active	–	
1400	IPI00016572	P62308	SNRPG	Small nuclear ribonucleoprotein G	8	0.441	0	ND (active)	False	False	Active	–	
1950	IPI00292221	Q9H9Y2	RPF1	Ribosome production factor 1	40	0.108	0	ND (active)	True	False	Active	–	
2033	IPI00023409	Q9BZI7	UPF3B	Isoform 1 of Regulator of nonsense transcripts 3B	58	0.075	0	ND (active)	True	False	Active	–	
2005	IPI00015924	Q9UBB9	TFIP11	Isoform 1 of Tuftelin-interacting protein 11	97	0.015	0	ND (active)	False	False	Active	–	
2039	IPI00293476	Q16540	MRPL23	39S ribosomal protein L23, mitochondrial	18	0.100	0	ND (active)	False	False	Active	–	
1525	IPI00171390	Q8NAV1	PRPF38A	Isoform 1 of Pre-mRNA-splicing factor 38A	37	0.195	0	ND (active)	True	False	Active	–	
1889	IPI00184884	Q8WV22	NSMCE1	Non-structural maintenance of chromosomes element 1 homolog	31	0.163	0	ND (active)	True	False	Active	–	
1459	IPI00301618	O60524	SDCCAG1	Isoform 1 of Serologically defined colon cancer antigen 1	123	0.084	0	ND (active)	True	False	Active	–	
2014	IPI00009329	P46939	UTRN	Utrophin	394	0.005	0	ND (active)	False	False	Active	–	
1408	IPI00641364	Q96G91	P2RY11	PPAN-P2RY11 protein	88	0.106	0	ND (active)	True	False	Active	–	
1548	IPI00334914	Q7Z2T5	C1orf25	Isoform 1 of TRM1-like protein	82	0.120	0	ND (active)	True	False	Active	–	
2008	IPI00641330	Q06587	RING1	Isoform 1 of E3 ubiquitin-protein ligase RING1	42	0.086	0	ND (active)	True	False	Active	–	
2051	IPI00014877	Q9NXS2	QPCTL	Isoform 2 of Glutaminyl-peptide cyclotransferase-like protein	43	0.067	0	ND (active)	True	False	Active	–	
2115	IPI00021305	P51946	CCNH	Cyclin-H	38	0.047	0	ND (active)	False	False	Active	–	
1491	IPI00014198	Q15024	EXOSC7	Exosome complex exonuclease RRP42	32	0.277	0	ND (active)	True	False	Active	–	
1852	IPI00383565	Q9NRL2	BAZ1A	Isoform 2 of Bromodomain adjacent to zinc finger domain protein 1A	175	0.021	0	ND (active)	True	False	Active	–	
1717	IPI00024143	Q9NRG9	AAAS	Aladin	60	0.048	0	ND (active)	True	False	Active	–	
1552	IPI00646762	Q9UKK9	NUDT5	Putative uncharacterized protein NUDT5	26	0.222	0	ND (active)	True	False	Active	–	
1934	IPI00045914	Q96T58	SPEN	Msx2-interacting protein	402	0.004	0	ND (active)	False	False	Active	–	
2102	IPI00219084	Q04206	RELA	Isoform 2 of Transcription factor p65	59	0.024	0	ND (active)	False	False	Active	–	
2069	IPI00290410	Q5QJE6	DNTTIP2	Deoxynucleotidyltransferase terminal-interacting protein 2	91	0.044	0	ND (active)	True	False	Active	–	
1506	IPI00015955	Q9NQT4	EXOSC5	Exosome complex exonuclease RRP46	25	0.259	0	ND (active)	True	False	Active	–	
2073	IPI00218803	P23142	FBLN1	Isoform B of Fibulin-1	77	0.061	0	ND (active)	True	False	Active	–	
1843	IPI00301202	Q9H0U3	MAGT1	magnesium transporter 1	42	0.077	0	ND (active)	True	False	Active	–	
1553	IPI00029697	Q06265	EXOSC9	Isoform 2 of Exosome complex exonuclease RRP45	51	0.237	0	ND (active)	True	False	Active	–	
2113	IPI00164481	Q9NUL3	STAU2	Isoform 2 of Double-stranded RNA-binding protein Staufen homolog 2	59	0.092	0	ND (active)	True	False	Active	–	

[1]Fold enrichments for proteins not detected in one data set could not be determined (ND).
[2]Specific enrichment determined by statistical analysis using Qspec based on Bayes factor >10 and absolute natural-logarithm-transformed fold change >1.5, resulting in estimated FDR <5%.
[3]Clusters selected using a Pearson correlation coefficient threshold of 0.80.
[4]Proteins present in the literature-curated database of adhesion-related components (the "adhesome") are indicated by YES. Additional subunits or paralogues of adhesome components are indicated by YES*.

Supplementary Table 2 | Functional enrichment analysis of proteins differentially enriched in integrin adhesion complexes.

Cluster[1]	Term[2,3]	Proteins[4]	Count (proteins per term)	Fold enrichment	Corrected *P* value
Active	Acetylation	SYNCRIP, RPLP2, SART3, OGDH, WTAP, DHX38, MAK16, PGAM5, DHX36, PDHA1, STAG2, KIF5B, PCBD2, PCID2, LIG3, MYH9, UNC13D, AQR, PTRF, RCC2, ASCC3, MAPK3, NLE1, EIF2AK2, MYBBP1A, RAD23B, PABPC4, AHCTF1, XAB2, PFN1, NAT10, PABPC1, FH, DHX8, SSSCA1, ZC3H14, DHX9, ACACA, NXF1, UBE2L3, VDAC2, SEPT11, PRPSAP2, GART, NOLC1, POP1, PARP1, EIF2A, USP15, USP14, HIST1H1E, HIST1H1C, ANP32B, HIST1H1B, AIFM1, ADNP, EPB41L2, EIF4A3, TAF15, TXN, EIF4A1, THOC7, THOC4, NUP107, EEF1D, SRP9, THOC1, SF3B5, SF3B3, SF3B2, SF3B1, USP39, DHX15, EXOC3, LYPLAL1, SNAP23, ETFB, FEN1, SSRP1, AIMP1, CS, TRIM28, LMNA, KIAA1967, TRIM25, LRRC47, ETF1, SF3A1, SF3A3, SDHA, HNRNPUL2, POLDIP3, SFPQ, RPL36A, ILKAP, SNRPD2, SRP19, CTNNBL1, CMBL, CSNK2A2, CSNK2A1, SND1, LRRC59, DNAJC8, API5, RPS27A, RPL35A, CMAS, RELA, TOPBP1, TIMELESS, VAMP8, SMARCA5, STMN1, SMARCA4, HDLBP, GLUD1, PPIL2, LRBA, UBE2V1, ARPC4, STIP1, RNF160, ARPC2, RPL10A, RAB8B, RBM12B, FSCN1, MYO1D, MPHOSPH10, USMG5, HDAC2, SMARCC1, SMARCC2, PTGES3, HINT3, RSF1, HP1BP3, HINT1, EDC4, ACOT9, PLRG1, DKC1, PRMT5, AHNAK, ZCCHC8, PPP2R1A, IK, KIF11, DDB1, CYCS, TBCE, PRPF4, PRPF6, TBCB, RIF1, TBCA, SNRNP200, MAPRE2, MAPRE1, PPP2R2A, FKBP5, FKBP4, FKBP3, SR140, TIMM13, IARS, PRPF8, CEBPZ, GTF3C5, NSUN5, RPL13A, CCT2, AURKB, CCT3, DDX18, RPP30, GTPBP4, SF3B14, BYSL, UBR4, DCTN1, DCTN2, BAZ1B, ABT1, DLD, ATP5C1, DBN1, MDH2, LMNB1, LMNB2, ALDOC, ACAT2, SUPV3L1, WDHD1, TKT, AFG3L2, PPA1, CCT7, NOP14, CCT5, CCT4, PTCD3, NOP16, CCT8, SUPT16H, IDI1, CHAF1B, ATP5B, VBP1, CANX, HSPH1, AP2B1, ATP5L, CAP1, HIST3H2BB, RBBP5, RBBP4, MBD3, RBBP7, RAD50, FARSB, RAB5A, UROD, PYCRL, YWHAZ, FKBP1A, MIF, TOE1, MDC1, BAZ2A, CSNK1A1, GDI2, PDS5B, PDS5A, IMMT, RRP15, YWHAB, YWHAE, YWHAG, AP2A2, YWHAH, UBTF, ATP5A1, TEX10, SEPT2, CLPB, EIF5B, EIF5A, TOP1, RAD21, MYST4, GNL3, NUP133, CLPX, COX6C, SLTM, PYCR1, PYCR2, TRIM33, CSTB, ANKFY1, SEPT7, SEPT9, SNX9, EEF1B2, BLM, GRB2, DIAPH1, COPS7B, SNX3, ZMYND8, EIF3C, EIF3A, EIF3B, PELP1, EIF3E, MTPN, NPM1, EIF3F, EIF3K, EIF3L, NPM3, GLO1, EIF3J, EIF3M, MRPS28, MRPS23, MRPS22, SNW1, TSN, PPIF, EIF4B, CORO1C, CYFIP1, RRS1, DNMT1, SPTBN1, TMPO, PDCD5, GLYR1, PUF60, DUT, UQCRC2, OSBP, UQCRC1, NUP188, CLTC, LARP1, RANBP1, MSN, RANBP2, TPR, MDN1, ZW10, UBE2I, MRPS7, TARS, BPTF, LARP7, ARCN1, CFL1, COMMD3, MATR3, PRKDC, HCFC1, RPA1, SAFB, SFRS2IP, GEMIN4, GEMIN5, DLST, TCP1, NUP153, TUBGCP2, PARK7, THRAP3, PSPC1, NGDN, ZC3H11A, RPL18, RPL19, RPL14, RPL13, LSM6, RPL15, XRCC6, PGD, STOML2, INTS1, RANGAP1, H1FX, HIST1H2BO, WDR75, LSM4, RPL10, LUC7L2, LSM3, LUC7L3, KRR1, YARS, EMG1, EIF2S3, PCM1, EIF2S1, RPS15, EIF2S2, UBL4A, RPS12, FLII, RPS13, AHCY, NHP2L1, HSPA1A, DIDO1, HADHA, HADHB, C3ORF10, RPS25, RPS26, RPS28, RPS29, CACYBP, RPS21, MEPCE, RPS23, NUDC, RPS24, UNC45A, MKI67, RPS9, WDR70, DLAT, RPS6, FXR1, RPS7, PCNA, MAP4, CRNKL1, NAP1L1, QARS, NAP1L4, HIST2H2AB, MTHFD2, DYNLL1, NUBP2, DYNLL2, WDR33, SYMPK, ARHGEF2, EXOSC9, ACO2, TTC35, EXOSC7, EXOSC4, EXOSC2, TPX2, EXOSC3, FLNB, SRPK1, FLNA, SMN1, PFDN1, PFDN6, NUP205, RUVBL2, CAND1, RUVBL1, KIAA1429, TUFM, NACA, HIST1H2AG, WRNIP1, RPL35, RPL36, RPL38, KIAA0776, RPL30, NUMA1, EZR, RPL31, RPL34, CSDE1, WDR12, RQCD1, TCEA1, HSD17B4, NMD3, SHMT2, HIST1H2BH, RPL26, RPL27, PPP1R10, RPL24, ACLY, RPL28, RPL29, TCEB2, PHF5A, TCEB1, PES1, XPO1, CHERP, XPO5, AP1G1, CAPZA2, CAPZA1, IQGAP1, VCL, DPY30, DDX24, RPN1, EIF1, DDX21, SNRPA1, ACTN4, MTA2, NUDT5, EEF2, ARID1A, HNRNPR, HNRNPU, DDX39, ARPC1B, PA2G4, MFAP1, YARS2, BAT3, LRPPRC, EP400, UBA52, BAT1, HMGB1, HMGB2, HMGB3, OTUD6B, ANLN, C14ORF166, HNRNPL, DDX47, TROVE2, HNRNPK, DDX46, RPL7, RPL6, RPL9, RPL8, RPL3, RPL5, HSPE1, HNRNPC, RPL7A, RPL4, DDX42, XPOT, ATP5J2, SMCHD1, MYL12B, RPL23A, WHSC2, RBMX, IDH3A, SAFB2, DDX56, VCP, NDUFV1, GTF2F2, DDX54, DDX52, MYH10, SKIV2L2, DEK, PRDX3, RPS2, PRDX1, DCAF13, RPS3A, LBR, NDUFS1, SARS, NUSAP1, NUP85, IPO9, PBK, CDC5L, RPS4X, NCL, HMGA1, ZFR, VASP, CDK2, PURA, COBRA1, PPM1G, RECQL, IPO7, PRDX6, IPO4, DDT, IPO5, LCP1, BCLAF1, NUP93, RPS15A, CDC73, GCN1L1, ESYT1, DCI, DAD1, SEC22B, HSPA5, HSPA8, CHD4, HSPA9, HBD, PDCD11, BOLA2, PNO1, ATP5F1, SMC5, ANXA1, COTL1, SMC2, SMC3, FBL, PWP2, SMC4, ANXA2, RDH11, SMPD4, VAPA, VAPB, U2AF2, SRP68, PDLIM1, SFRS6, PRIM1, GOT2, SFRS7, SFRS5, SIN3A, H2AFV, MRPL37, H2AFX, MRPL39, SUPT5H, EIF2B5, MRPL53, CLNS1A, OLA1, SFRS1, SFRS3, SFRS2, RFC3, RFC4, GAPVD1, MAD2L1, NOP2, RFC1, HUWE1, SERBP1, RARS, SRP72, ADD1, SUPT6H, TALDO1, BOP1, CAPZB, CDC37, PIN1, TPI1, MOV10, MACF1, NUP50, COX6B1, EMD, SUB1, S100A11, CDC23, PMF1, ABCF2, COPS5, AP1B1, COPS3, COPS4, CCNT1, UTP15, ACTR3, PARN, ATAD3A, ATAD3B, BAG2, SLC25A1, DYNC1H1, TOP2B, TOP2A, DNAJC19, AP3B1, ABCE1, POGZ, POLR1D, SLC25A6, POLR1C, GMPS, TRAP1, GTF2I, RBM39, PMPCA, PRPF38A, USP7, POLR2E, ECH1, POLR2J, RPL27A, ATP6V1G1, HPRT1, PDAP1, SUMO3, PRPF19, SET, FASN, ACTL6A, AATF, SNRNP70, RBM28, RBM26, BCAS2, NASP, ILF3, POLR3A, EIF2B1, PSMD13, PSMD12, PSMD11, ATP6V1E1, PGK1, CIT, RBM15, PAICS, RBM17, MORF4L1, CBX3, CALB1, PNN, TBC1D15, CISD2, SRRM1, ACIN1, RBM10, MLL2, EFTUD2, PPP1CC, PECI, RSL1D1, PSMA6, PSMA5, PSMA3, SNRPA, SNRPF, MYL6, PPME1, PNPT1, COPZ1, TRRAP, PSMA7, GCLM, ZFAND1, CSE1L, PSMB3, GSTK1, MAFG, TBL3, TBL2, CSTF2, CKAP5, PTPN2, RNPS1, SSB, ABHD14B, SUGT1, PSMC5, TXNDC17, PSMC4, PSMC3, PSMC2, PSMC1, CPNE1, CPNE3, PTPN1, WDR1, SMC1A, DAP3, HIST4H4, TLN1, TLN2, MKI67IP, BZW2, SBDS, PSMD1, PSMD2, PSMD5, PSMD7, KPNB1, FTSJ3, DYNLT1, MCM2, SMU1, C1QBP, KPNA6, PSME3, KPNA4, KPNA3, KPNA2, GLTSCR2, GLRX3, NDUFB4, SBNO1, NDUFB9, TPM3, THYN1, TNPO1, APEX1, ENO1, TNPO3, MSH6, NDUFA5, NDUFA2, ERH, NDUFA6, NDUFA7, CENPF, PSMG3, DIS3, GPI, SON, PSMG1, CKS2, SPTAN1	798	4.23	0
Active	Phosphoprotein	PPP2R5D, RPLP2, SYNCRIP, VPRBP, SART3, WTAP, SART1, C1ORF77, DHX38, MAK16, INCENP, PDHA1, DHX30, STAG2, BSG, KIF5B, NDNL2, PCID2, LIG1, LIG3, MYH9, NCAPD3, NCAPD2, UNC13D, PTRF, DHX29, RCC2, ASCC3, KIAA1524, MAPK3, NLE1, EIF2AK2, MYBBP1A, RAD23B, SDAD1, KIF4A, OSTF1, PABPC4, AHCTF1, XAB2, SCRIB, ZNF512, PFN1, NCAPG2, NAT10, PABPC1, SFRS14, SFRS11, FH, SFRS12, ZC3H14, DHX8, SSSCA1, DHX9, ZC3H15, KIAA0664, ACACA, VDAC2, PRPSAP2, GART, NOLC1, POP1, POP4, PARP1, AP2S1, EZH2, EIF2A, AASDHPPT, SRPR, ANP32A, USP15, USP14, HIST1H1E, HIST1H1C, ANP32B, AIFM1, HIST1H1B, ANP32E, ADNP, PI4KA, EPB41L2, EIF4A3, TAF15, TXN, EIF4A1, USP20, THOC4, THOC5, NUP107, EEF1D, THOC2, TMCO1, THOC1, SNAP29, PHLDB1, SF3B5, SF3B2, SF3B1, USP39, DHX15, DHX16, SNAP23, USP34, SSRP1, UPF2, EPPK1, AIMP2, CWC27, TRIM28, LMNA, KIAA1967, TRIM25, LRRC47, SF3A1, SF3A3, ACSM3, SDHA, HNRNPUL2, CSNK1D, POLDIP3, SFPQ, POLDIP2, CWC22, TXNL4A, ILKAP, RPL36A, HBS1L, CTNNBL1, CSNK2A2, CSNK2A1, DNAJC9, SND1, LRRC59, DNAJC8, EED, DNAJC2, API5, RPS27A, RELA, TOPBP1, MRTO4, TIMELESS, VAMP8, SMARCA5, DNTTIP2, STMN1, SMARCA4, ARFGAP2, HDLBP, GLUD1, LRBA, UBE2V1, STIP1, MAPKAPK2, PTRH2, RNF160, SMARCB1, PPIL4, RPL10A, ASF1B, ASF1A, AP2M1, RAB8B, RBM12B, FSCN1, MYO1D, MPHOSPH10, EPS15, NMT1, HDAC3, HDAC2, SMARCC1, SMARCC2, ABL1, PTGES3, KIF22, RSF1, HP1BP3, LYAR, EDC4, WARS, KIF2C, DKC1, DDA1, AHNAK, KIF2A, ZCCHC8, KIF14, IK, KIF11, DDB1, TBCE, PRPF3, PRPF6, TBCB, RIF1, SNRNP200, MAPRE2, MAPRE1, OAT, FKBP5, FKBP4, SR140, FKBP3, ARID2, IARS, TFAM, PRPF8, CEBPZ, VPS35, EHD1, GTF3C1, NSUN5, GTF3C3, SFRS13A, SAMSN1, RPP38, RAB3GAP2, TRMT2A, UTRN, AURKB, CCT3, RPP30, DDX10, ANAPC1, TXNL1, GTPBP4, BCR, ANAPC5, SF3B14, BYSL, UBR4, DCTN1, DCTN2, BAZ1A, BAZ1B, UBR5, ATP5C1, DBN1, MDH2, LMNB1, LMNB2, ALDOC, COIL, AHSG, TK1, VRK1, C1ORF128, DMD, SUPV3L1, WDHD1, TKT, AARSD1, DDX6, NOP14, NSMCE4A, NOP16, CCT8, SUPT16H, CHAF1B, ATP5B, FERMT3, CANX, HSPH1, AP2B1, SEH1L, FANCI, CAP1, ATP5I, ATP6V0D1, HIST3H2BB, GOLGA2, RBBP5, RBBP4, CCNH, MBD3, RBBP7, RAD50, FARSB, RAB5A, UROD, CARHSP1, YWHAZ, MTDH, PPFIA1, FKBP1A, MIF, TOE1, MDC1, NCAPG, BAZ2A, MLLT4, UBXN1, CSNK1A1, GDI2, PDS5B, RRP12, PDS5A, RRP15, YWHAB, BIRC6, YWHAE, YWHAG, AP2A2, YWHAH, UBTF, AP2A1, NOP58, ATP5A1, NOP56, TEX10, MRPS36, SEPT2, EIF5B, EIF5A, COX4NB, PDHB, TOP1, RAD21, GNL2, MYST4, GNL3, NUP133, RRP1, RRP9, CLPX, SLTM, PYCR2, SEC61B, TRIM33, CSTB, ERC1, SEPT7, DST, SEPT9, SNX9, MRPS16, ZC3H4, EEF1B2, BLM, DIAPH1, GRB2, TH1L, SNX3, ZMYND8, SPC24, EIF3C, EIF3D, EIF3A, EIF3B, EIF3G, EIF3H, PELP1, EIF3E, CENPC1, EIF3F, NPM1, EIF3K, NPM3, EIF3J, EIF3M, FN1, TSR1, SNW1, EIF4B, CORO1C, CORO1B, TRIM56, PPIG, SPTBN2, C2ORF49, CYFIP1, RRS1, DNMT1, SPTBN1, TMPO, PDCD5, GLYR1, PHPT1, PUF60, PHF6, DUT, UQCRC2, COPA, OSBP, CLTA, UQCRC1, NUP188, CLTC, PDCD4, LARP1, RANBP1, MSN, RANBP2, TPR, MDN1, ZW10, G3BP2, MRPS5, LAS1L, TARS, BPTF, LARP7, CFL1, MATR3, COPE, UTP3, NUP160, TRA2B, TRA2A, HCFC1, PRKDC, RPA3, RPA1, SAFB, SFRS2IP, GEMIN6, ERCC3, GEMIN4, GEMIN5, TCP1, NUP153, SAP30BP, NUP155, TUBGCP2, PARK7, RPAP3, MPG, THRAP3, PSPC1, NGDN, ZC3H11A, RPL18, KIFC1, RPL17, RPL19, RPL14, RPL13, SURF6, RPL15, XRCC6, INTS1, STOML2, RANGAP1, H1FX, ZNF638, INTS3, XRCC1, CIAPIN1, SSR1, HIST1H2BO, WDR75, CDCA8, WDR77, LUC7L2, LSM2, LUC7L3, KRR1, EMG1, HMG20A, EIF2S3, CCDC137, PCM1, AAAS, EIF2S1, EIF2S2, UBL4A, RPS13, FLII, MYO18A, HSPA1A, DIDO1, HADHA, C3ORF10, RPS26, RPS28, CACYBP, NUDC, MEPCE, RPS24, UNC45A, ZNF622, CCDC86, MKI67, PCNT, NUF2, RPS9, WDR70, RPS6, RPS8, GCFC1, FXR1, UPF3B, MAP4, RG9MTD1, NAP1L1, QARS, NAP1L4, HIST2H2AB, DYNLL1, WDR33, SYMPK, SRPK2, STX4, ARHGEF2, ARHGEF1, EXOSC9, C21ORF70, EXOSC5, EXOSC2, TPX2, FLNC, FLNB, FLNA, SRPK1, SMN1, RRM2, NUP205, CAND1, RUVBL2, WDR43, WDR46, KIAA1429, NACA, HIST1H2AG, WRNIP1, POLA1, BCCIP, KIAA0776, NUMA1, RPL30, EZR, WDR18, REXO4, RPL31, RPL34, NUP210, CSDE1, TCEA1, HSD17B4, NMD3, NSA2, SHMT2, HIST1H2BH, PAK1IP1, ZNF24, RPL26, PPP1R10, RPL24, ACLY, RPL28, RPL29, RPL21, PES1, EIF6, XPO1, CHERP, RNMT, XPO5, CAPZA2, EIF5, CNOT1, IQGAP1, VCL, DDX27, EBNA1BP2, ANK1, SMARCD2, DDX23, DDX24, PSIP1, EIF1, DDX21, DDX20, RPN2, RPRD1B, ASPM, SNRPA1, ACTN4, MTA2, NUDT5, MTA1, ARID1A, EEF2, HNRNPU, DDX39, ARPC1B, PA2G4, SMARCE1, MFAP1, YARS2, BAT3, EP400, UBA52, NMNAT1, BAT1, HMGB1, HMGB2, OTUD6B, SNRPB2, ANLN, HNRNPL, DDX47, TROVE2, DDX46, NIPBL, HNRNPK, RPL7, RPL6, RPL8, RPL3, RPL5, HSPE1, HNRNPC, RPL4, DDX41, DDX42, SMCHD1, NIN, EPB41, ARID3A, MYL12B, RPL23A, DOCK8, WHSC2, RBMX, SAFB2, HSPBP1, DDX56, VCP, PLK1, TSC2, GTF2F2, DDX50, DDX54, DDX52, MYH10, DDX51, DEK, RPS2, PRDX1, GPKOW, RPS3A, CDK5RAP2, LBR, SARS, CDK9, NUSAP1, KTN1, NUP85, CDK7, PBK, CDC5L, KIDINS220, RPS4X, NCL, ZFR, HMGA1, VASP, CDK2, PURA, COBRA1, PPM1G, FAM120A, IPO7, PRDX6, IPO4, IPO5, UCHL3, CPSF3, CPSF1, LCP1, STON2, BCLAF1, RNH1, NUP93, CDC73, ESYT2, GCN1L1, ESYT1, CHD7, CCDC124, SEC22B, CHD1, PAFAH1B2, HSPA5, HSPA8, CHD4, TSEN34, HBD, HSPA9, PDCD11, PNO1, ANXA1, SMC5, SMC6, DRG2, COTL1, PWP2, FBL, SMC3, SMC4, ANXA2, RDH11, ORC3L, TSFM, PDC, SMPD4, VAPB, U2AF2, PDLIM4, SRP68, PDLIM1, CD2AP, GOT2, SFRS6, SFRS7, SFRS4, SIN3A, SFRS5, PRIM2, H2AFY, H2AFX, SUPT5H, EIF2B4, EIF2B5, CLNS1A, YY1, POLE, TBRG4, SFRS1, ESCO2, SFRS3, RIC8A, SFRS2, RFC3, GAPVD1, MAD2L1, NOP2, RFC1, HUWE1, SERBP1, RARS, SRP72, NUP43, ADD1, SUPT6H, TALDO1, MRE11A, NOC3L, BOP1, IGF2BP3, CAPZB, CDC37, PIN1, MOV10, TPI1, MACF1, CEP170, NUP50, PBRM1, EMD, SUB1, S100A11, CDC23, YTHDC2, PMF1, CDC27, CKMT1A, TMOD3, UTP14A, UTP20, ADAR, ABCF1, COPS2, ABCF2, AP1B1, UTP18, COPS3, CCNT1, TRMT1, ACTR3, PARN, ATAD3A, BAG2, DYNC1H1, TOP2B, TOP2A, AP3B1, TFIP11, PRPF31, POLR1E, POGZ, INPPL1, FLOT2, SLC25A6, FLOT1, POLR1A, POLR1C, HERC2, SPEN, HERC1, GMPS, NOC2L, TRAP1, GTF2I, NOL10, RBM39, PRPF38A, USP7, RBM34, SHROOM3, ECH1, RPL27A, POLR2C, BMS1, STAU2, PDAP1, STAU1, PRPF19, SUMO1, SET, CD2BP2, GATAD2A, FASN, GATAD2B, AP3D1, AATF, ACTL6A, SNRNP70, RBM27, RBM28, RBM26, C14ORF21, RRBP1, NASP, ILF3, POLR3B, PSMD14, TJP1, HYDIN, PSMD13, ILF2, PPP1R8, PSMD11, ATP6V1E1, PGK1, CIT, RBM15, PAICS, RBM17, CBX3, CBX1, PNN, CBX5, TBC1D15, SRRM1, ACIN1, RBM10, MLL2, ARGLU1, EFTUD2, PPP1CC, RSL1D1, SUZ12, MAP4K4, PSMA6, PSMA5, PSMA3, SNRPA, MYL6, PPME1, NAPG, STRAP, COPZ1, PNPT1, KEAP1, ATP6V1B2, TRRAP, UBAC1, PSMA7, FTH1, C12ORF11, BTF3, CLASP1, CLASP2, STRBP, CSTF2, TBL2, CKAP5, PTPN2, RNPS1, SSB, HEATR1, WRN, SUGT1, CTR9, PSMC4, PSMC3, PSMC1, CPNE1, WDR3, CPNE3, AHCYL2, PTPN1, WDR1, SMC1A, CSTF1, DAP3, HIST4H4, TLN1, TLN2, MKI67IP, BZW2, BZW1, SBDS, URB1, PSMD1, PSMD2, PSMD4, PSMD7, KPNB1, FTSJ3, NOL6, ELP3, MPP1, NOL7, RING1, MCM2, STOM, CRKL, C1QBP, KPNA6, PSME3, KPNA4, KPNA3, KPNA2, KPNA1, SBNO1, USP9X, NDUFB9, TPM3, RGS10, UTP11L, PPP1R12A, THYN1, APEX1, TNPO1, TNPO3, ENO1, MSH6, NDUFA7, CENPF, KIF18B, GPI, PSMG1, SON, CENPV, SPTAN1	959	1.84	5.40E-134
Active	Nucleus	KIFC1, SURF6, PPP2R5D, LSM6, XRCC6, LSM7, INTS1, SYNCRIP, H1FX, RANGAP1, ZNF638, MED22, INTS3, SART3, WTAP, XRCC1, SART1, HIST1H2BO, INTS9, WDR75, CDCA8, DHX38, MAK16, INCENP, WDR77, LSM4, DHX36, LSM3, LSM2, STAG2, LUC7L3, TBPL1, KRR1, NDNL2, EMG1, LIG1, LIG3, HMG20A, NCAPD3, NCAPD2, UXT, AQR, PTRF, RCC2, NLE1, FLII, MYBBP1A, RAD23B, KIF4A, SDAD1, NHP2L1, CHCHD1, AHCTF1, BCL2L1, DIDO1, XAB2, ZNF512, NCAPG2, CACYBP, NAT10, PABPC1, SFRS14, NUDC, SFRS11, SFRS12, UNC45A, DHX8, ZC3H14, ZNF622, DHX9, CCDC86, ZC3H15, MKI67, NUF2, TFCP2, NXF1, GCFC1, UPF3B, NOLC1, POP1, PCNA, POP4, PARP1, POP7, CRNKL1, GAR1, EZH2, NAP1L1, NAP1L4, HIST2H2AB, NUBP2, ANP32A, WDR33, SRPK2, SYMPK, HIST1H1E, EXOSC8, EXOSC9, ANP32B, EXOSC6, HIST1H1C, EXOSC7, AIFM1, HIST1H1B, TTC35, EXOSC4, EXOSC5, ANP32E, EXOSC2, GTF2H4, ADNP, TPX2, EXOSC3, GTF2H3, SRPK1, GTF2H2, SMN1, EIF4A3, TAF15, NUP205, THOC7, CAND1, RUVBL2, THOC4, RUVBL1, THOC5, NUP107, THOC2, WDR43, KIAA1429, THOC1, NACA, HIST1H2AG, WRNIP1, POLA1, BCCIP, SF3B5, FCF1, SF3B3, SF3B2, SF3B1, NUMA1, REXO4, NUP210, USP39, DHX15, DHX16, RQCD1, WDR12, TCEA1, FEN1, NMD3, NSA2, SSRP1, AIMP1, AIMP2, HIST1H2BH, PAK1IP1, TRIM28, ZNF24, LMNA, PPP1R10, SF3A1, SF3A3, HNRNPUL2, SFPQ, POLDIP3, POLDIP2, TCEB2, PHF5A, TCEB1, CWC22, PES1, TXNL4A, EIF6, XPO1, RPL36A, RNMT, XPO5, SNRPD3, SNRPD1, SNRPD2, CNOT7, CTNNBL1, DDX27, DPY30, EBNA1BP2, IMP3, DDX23, SMARCD2, SND1, SMARCD1, PSIP1, EED, DDX21, DDX20, DNAJC2, IMP4, API5, ASPM, RPS27A, SNRPA1, ACTN4, CMAS, MTA2, RELA, GABPA, MTA1, ARID1A, TOPBP1, HNRNPR, HNRNPU, MRTO4, RCL1, DDX39, PA2G4, TIMELESS, SMARCE1, SMARCA5, DNTTIP2, BAT3, LRPPRC, EP400, UBA52, SMARCA4, NMNAT1, BAT1, HMGB1, HDLBP, HMGB2, HMGB3, PPIL2, SNRPB2, UBE2V1, C14ORF166, STIP1, ANLN, HNRNPL, DDX47, DDX46, NIPBL, HNRNPK, SMARCB1, PPIL4, RPL5, HNRNPC, ASF1B, DDX41, RHOXF2, ASF1A, DDX42, GINS1, XPOT, EPB41, GINS3, ARID3A, MPHOSPH10, WHSC2, RBMX, SAFB2, HDAC3, DDX56, HDAC2, VCP, PLK1, SMARCC1, SMARCC2, GTF2F2, DDX50, ABL1, DDX54, DDX52, DDX51, HINT3, KIF22, RSF1, HP1BP3, HINT1, LYAR, EDC4, DEK, SKIV2L2, KIF2C, DCAF13, PLRG1, GPKOW, DKC1, RPS3A, PRMT5, GTF2A2, LBR, AHNAK, KIF14, IK, DDB1, NUSAP1, CDK9, PRPF3, NUP85, IPO9, CDC5L, CDK7, NCL, ZFR, HMGA1, PRPF4, PURA, PRPF6, COBRA1, RECQL, RIF1, IPO7, IPO4, SNRNP200, IPO5, ORC5L, CPSF3, CPSF1, BCLAF1, FKBP5, FKBP4, FKBP3, NUP93, CDC73, ARID2, HIC2, CHD7, PRPF8, CEBPZ, GTF3C5, CHD1, MYCBP, GTF3C1, CHD4, TSEN34, GTF3C3, PDCD11, PNO1, ANXA1, SFRS13A, SMC5, SMC6, SMC2, SMC3, FBL, PWP2, SMC4, ORC3L, RPP38, U2AF2, SRP68, AURKB, SFRS6, SFRS7, SFRS4, SFRS5, SIN3A, H2AFV, RPP30, H2AFY, H2AFX, SUPT5H, CLNS1A, GTPBP4, PRAME, SF3B14, YY1, BYSL, POLE, UBR4, POLB, SFRS1, ESCO2, SFRS3, SFRS2, RFC3, RFC4, BAZ1A, MAD2L1, NOP2, BAZ1B, RFC1, HUWE1, ABT1, SERBP1, UBR5, NUP43, SUPT6H, LMNB1, LMNB2, MRE11A, NOC3L, BOP1, IGF2BP3, COIL, PIN1, VRK1, FRG1, NUP50, PBRM1, SUPV3L1, WDHD1, EMD, NKRF, NOC4L, SUB1, S100A11, PMF1, CDC27, NOP10, RPF1, NOP14, NOP16, SUPT16H, UTP14A, UTP20, CHAF1B, ADAR, ENY2, COPS2, COPS5, UTP18, COPS6, COPS3, COPS4, CCNT1, VBP1, UTP15, PARN, SEH1L, FANCI, NSMCE1, HIST3H2BB, TOP2B, TOP2A, TFIP11, PRPF31, RBBP5, RBBP4, POLR1E, POGZ, CCNH, POLR1D, NIP7, POLR1A, POLR1C, MBD3, POLR1B, SPEN, RBBP7, RAD50, NOC2L, GTF2I, NOL10, NOL11, SNRNP40, RBM39, NHP2, PRPF38A, USP7, POLR2G, RBM34, MTDH, POLR2E, POLR2J, DIMT1L, POLR2C, BMS1, STAU2, POLR2B, RPP14, PRPF19, SUMO3, SUMO1, SET, TOE1, NCAPG, MDC1, GATAD2A, GATAD2B, AATF, ACTL6A, SNRNP70, RBM27, RBM28, BAZ2A, BCAS2, PDS5B, RRP12, PDS5A, NASP, ILF3, POLR3A, POLR3B, ILF2, UBTF, PPP1R8, NOP58, NOP56, RBM15, TEX10, RBM17, MORF4L1, EIF5A, CBX3, CBX1, PNN, CBX5, TOP1, RAD21, CLP1, SRRM1, ACIN1, GNL2, RBM10, MYST4, GNL3, MLL2, NUP133, RRP1, EFTUD2, RRP9, PPP1CC, SUZ12, SLTM, RSL1D1, TRIM33, PSMA6, PSMA5, PSMA3, CSTB, SNRPB, SNRPA, SNRPF, SNRPG, BLM, STRAP, TH1L, COPS7B, KEAP1, TRRAP, PSMA7, SPC24, CSE1L, PELP1, PSMB3, EIF3E, CENPC1, NPM1, EIF3K, BTF3, NPM3, MAFG, TBL3, CSTF2, TSR1, RNPS1, SSB, SNW1, ABHD14B, HEATR1, WRN, TSN, CTR9, PPIE, PSMC5, PPIG, PSMC4, PSMC3, PSMC2, PSMC1, CPNE1, WDR3, DNMT1, RRS1, TMPO, SMC1A, CSTF1, PDCD6, GLYR1, PUF60, PHF6, DUT, HIST4H4, NUP188, MKI67IP, PDCD4, C20ORF11, URB2, URB1, CIRH1A, RANBP2, TPR, MDN1, KPNB1, FTSJ3, NOL6, ELP3, NOL7, RING1, LAS1L, UBE2I, MCM2, SMU1, BPTF, NRM, LARP7, CFL1, PSME3, KPNA4, KPNA3, KPNA2, KPNA1, MATR3, UTP3, GLTSCR2, NUP160, TRA2B, UTP6, TRA2A, HCFC1, PRKDC, CCDC59, RPA3, RPA1, EXOSC10, KIAA0020, UTP11L, SAFB, THYN1, RSL24D1, SFRS2IP, GEMIN6, ERCC3, TNPO1, APEX1, GEMIN4, TNPO3, ERCC2, ENO1, GEMIN5, FAM50A, MSH6, NUP153, CENPF, SAP30BP, NUP155, PARK7, DIS3, MPG, SON, PSMG2, THRAP3, CENPV, PSPC1, NGDN	656	2.14	5.45E-101
Active	Ribonucleoprotein	MRPS36, RPL18, RPL17, SRP14, RPL36A, MRPS34, RPL19, RPL14, RPL13, SNRPD3, LSM6, RPL15, SRP68, LSM7, SNRPD1, RPLP2, SNRPD2, SYNCRIP, SRP19, IMP3, RPL26L1, LSM4, FAU, RPL10, MRPL37, LSM3, LSM2, MRPL39, IMP4, RPS27A, RPL36AL, MRPL53, SNRPA1, KRR1, RPL35A, EFTUD2, RRP9, HNRNPR, HNRNPU, PA2G4, RPS15, RPS12, SNRPB, SNRPA, RPS13, SRP72, MRPL48, MRPL45, SNRPF, UBA52, MRPL43, SNRPG, MRPS16, NHP2L1, MRPS11, SNRPB2, RPS25, HNRNPL, RPS26, TROVE2, RPS28, HNRNPK, RPS29, RPL7, RPL6, RPL9, RPL8, RPL3, MRPL55, RPL5, HNRNPC, RPL4, RPL10A, RPL7A, RPS21, RPS23, RPS24, MRPS27, MRPS26, MRPS28, MRPS23, MRPS22, MPHOSPH10, RPS9, SSB, MRPS21, HEATR1, RPL23A, RPS6, NOP10, RBMX, RPS8, RPS7, RPL37A, PUF60, DAP3, GAR1, RPS2, HNRPLL, DCAF13, DKC1, RPS3A, MRPL2, PRPF31, MRPL1, MRPL3, MRPS5, MRPS6, MRPS7, SRPRB, RPS4X, MRPS2, CPSF3, NHP2, SRP9, RPL27A, RPL35, RPL36, RPS15A, RPL38, MRPL11, RPL30, MRPL13, RPL32, MRPL15, RPL31, MRPL14, PRPF8, RPL34, MRPL17, MRPL18, SNRNP70, NSA2, RPL26, RPL27, RPL24, RPL28, FBL, RPL29, MRPL23, MRPL22, MRPL27, RPL13A, RPL21, RPL7L1	145	7.26	2.08E-87
Active	**RNA-binding**	XPO1, SRP14, RPL36A, RNMT, SURF6, XPO5, SNRPD3, LSM6, TRMT2A, U2AF2, SRP68, LSM7, EIF5A, SYNCRIP, ZNF638, SRP19, SART3, SFRS6, SFRS7, IMP3, SFRS4, SFRS5, DDX18, EIF1AX, DDX24, LSM4, SRRM1, LSM3, DDX21, DDX10, LSM2, RBM10, DHX30, KRR1, RPL35A, SNRPA1, YARS, SF3B14, RRP9, SFRS1, HNRNPR, HNRNPU, SFRS3, SFRS2, SLTM, PA2G4, AQR, PTRF, ABT1, EIF2S1, SERBP1, SNRPB, SNRPA, SNRPF, EIF2AK2, LRPPRC, BAT1, SNRPG, HDLBP, NHP2L1, PABPC4, PNPT1, SNRPB2, IGF2BP3, HNRNPL, MOV10, DDX47, TROVE2, EIF3B, HNRNPK, DDX46, DDX49, RPL7, EIF3G, RPL8, NPM1, PPIL4, RPL5, HNRNPC, STRBP, PABPC1, DDX41, SFRS11, DDX42, XPOT, ZC3H14, DHX9, CSTF2, RBM12B, RPS9, SSB, RPL23A, RNPS1, NXF1, RBMX, RPF1, FXR1, DDX6, SAFB2, EIF4B, PPIE, DDX56, UPF3B, DDX50, CPNE1, DDX54, DDX52, PUF60, ADAR, DDX51, GAR1, MKI67IP, PDCD4, HNRPLL, LARP1, PARN, DKC1, NOL6, PRPF31, EXOSC8, EXOSC9, EXOSC6, EXOSC7, NIP7, EXOSC4, EXOSC5, EXOSC2, G3BP2, EXOSC3, CDC5L, SPEN, RPS4X, NCL, ZFR, SMN1, EIF4A3, TAF15, FAM120A, LARP7, EIF4A1, THOC7, THOC4, RBM39, THOC5, THOC2, CPSF3, NHP2, MATR3, CPSF1, SRP9, THOC1, POLR2G, RBM34, TRA2B, TRA2A, SR140, DIMT1L, STAU2, STAU1, EXOSC10, KIAA0020, SAFB, PRPF8, CSDE1, SNRNP70, RBM27, BAZ2A, RBM28, GEMIN5, RBM26, C14ORF21, UPF2, AIMP1, PNO1, SFRS13A, PPP1R10, ILF3, FBL, DIS3, SON, PPP1R8, POLDIP3, SFPQ, PSPC1, RBM15, RBM17	186	4.81	6.48E-76
Active	**mRNA splicing**	U2AF2, LSM6, SNRPD3, LSM7, SNRPD1, SNRPD2, SYNCRIP, WTAP, SART1, PNN, SFRS6, SFRS7, SFRS4, SFRS5, DHX38, DDX23, SRRM1, LSM4, LSM3, LSM2, DDX20, RBM10, LUC7L3, SNRPA1, SF3B14, EFTUD2, SFRS1, HNRNPR, SFRS3, HNRNPU, SFRS2, DDX39, AQR, SNRPB, SNRPA, SNRPF, SNRPG, BAT1, NHP2L1, STRAP, SNRPB2, XAB2, DDX46, HNRNPK, FRG1, HNRNPC, PABPC1, DDX41, SFRS14, SFRS11, SFRS12, DHX8, SNW1, RNPS1, RBMX, PPIE, PUF60, CRNKL1, SKIV2L2, PLRG1, ZCCHC8, TFIP11, SRPK2, PRPF31, PRPF3, CDC5L, PRPF4, SRPK1, SMN1, PRPF6, EIF4A3, SNRNP200, THOC7, THOC4, SNRNP40, THOC5, RBM39, THOC2, THOC1, KIAA1429, PRPF38A, TRA2B, TRA2A, SF3B5, SF3B3, SF3B2, PRPF19, SF3B1, PRPF8, USP39, DHX15, DHX16, SFRS2IP, GEMIN6, RBM28, GEMIN4, GEMIN5, BCAS2, SFRS13A, SF3A1, SF3A3, PPP1R8, SFPQ, PHF5A, CWC22, TXNL4A, RBM17	107	7.15	1.56E-62
Active	**mRNA processing**	RNMT, U2AF2, LSM6, SNRPD3, LSM7, SNRPD1, SNRPD2, SYNCRIP, WTAP, SART1, PNN, SFRS6, SFRS7, SFRS4, SFRS5, DHX38, DDX23, CLP1, SRRM1, LSM4, LSM3, DDX20, LSM2, RBM10, LUC7L3, SNRPA1, SF3B14, EFTUD2, SFRS1, HNRNPR, SFRS3, HNRNPU, SFRS2, DDX39, AQR, SNRPB, SNRPA, SNRPF, SNRPG, BAT1, NHP2L1, STRAP, SNRPB2, XAB2, DDX46, HNRNPK, FRG1, HNRNPC, PABPC1, DDX41, SFRS14, SFRS11, SFRS12, DHX8, CSTF2, SNW1, RNPS1, RBMX, PPIE, CSTF1, PUF60, ADAR, CRNKL1, SKIV2L2, PLRG1, ZCCHC8, TFIP11, SRPK2, SYMPK, PRPF31, PRPF3, CDC5L, PRPF4, SRPK1, SMN1, PRPF6, EIF4A3, SNRNP200, THOC7, THOC4, SNRNP40, THOC5, RBM39, CPSF3, THOC2, CPSF1, PRPF38A, THOC1, KIAA1429, TRA2B, TRA2A, SF3B5, SF3B3, SF3B2, PRPF19, SF3B1, PRPF8, USP39, DHX15, DHX16, SFRS2IP, SNRNP70, GEMIN6, RBM28, GEMIN4, TSEN34, GEMIN5, BCAS2, SFRS13A, SF3A1, SF3A3, PPP1R8, SFPQ, PHF5A, CWC22, TXNL4A, RBM17	117	6.29	7.29E-61
Active	Protein biosynthesis	EIF6, RPL18, RPL17, RPL19, RPL13, EIF5, HBS1L, EIF5B, EIF5A, RPLP2, QARS, EIF2A, RPS2, WARS, RPS3A, EIF1AX, RPL10, FAU, EIF1, RPS27A, EIF2B4, EIF2B5, RPL36AL, RPL35A, YARS, MRPL3, SARS, EIF2S3, EEF2, RPS4X, EIF4A3, TARS, DHX29, EIF2S1, RARS, EIF2S2, RPS15, EIF4A1, FARSB, RPS12, RPS13, YARS2, EEF1D, UBA52, TUFM, EEF1B2, RPS15A, RPL36, RPS25, IARS, EIF3C, RPS26, EIF3D, EIF3A, RPL30, RPS28, EIF3B, EIF3G, RPL7, RPL32, RPS29, RPL31, EIF3H, RPL6, EIF3E, RPL9, EIF3F, RPL8, EIF3K, RPL3, EIF3L, RPL5, RPL7A, EIF3J, RPS21, RPS23, EIF3M, RPS24, AIMP1, AIMP2, RPL26, RPL27, RPS9, RPL24, ETF1, RPS6, EIF2B1, RPS8, RPL29, RPS7, EIF4B, GSPT1, RPL13A, TSFM, RPL21	95	7.06	1.81E-54
Active	Ribosomal protein	RPL18, MRPS36, RPL17, RPL36A, MRPS34, RPL19, RPL14, RPL13, RPL15, RPLP2, RPS2, IMP3, RPS3A, RPL26L1, RPL10, FAU, MRPL37, MRPL39, RPS27A, RPL36AL, MRPL53, MRPL2, RPL35A, MRPL1, MRPL3, MRPS5, MRPS6, MRPS7, RPS4X, MRPS2, RPS15, RPS12, RPS13, MRPL48, MRPL45, MRPL43, UBA52, MRPS16, MRPS11, RPL27A, RPL35, RPS15A, RPL36, RPL38, RPS25, MRPL11, RPS26, MRPL13, RPL30, RPS28, RPL7, MRPL15, RPS29, RPL32, RPL31, RPL6, MRPL14, RPL34, MRPL17, RPL9, RPL8, RPL3, MRPL55, MRPL18, RPL5, RPL4, RPL10A, RPL7A, RPS21, RPS23, RPS24, MRPS27, MRPS26, MRPS28, MRPS23, MRPS22, RPL26, RPL27, RPS9, MRPS21, RPL24, RPL23A, RPS6, RPL28, RPS8, RPL29, RPS7, MRPL23, MRPL22, RPL13A, MRPL27, RPL21, RPL7L1, RPL37A, DAP3	95	7.06	1.81E-54
Active	**Spliceosome**	CRNKL1, SNRPD3, U2AF2, SNRPD1, SYNCRIP, SNRPD2, SKIV2L2, SART1, PNN, DHX38, PLRG1, DDX23, SRRM1, LSM3, DDX20, LSM2, ZCCHC8, TFIP11, PRPF31, SNRPA1, EFTUD2, PRPF3, CDC5L, SFRS1, PRPF4, HNRNPR, HNRNPU, SMN1, PRPF6, EIF4A3, AQR, SNRNP200, SNRPB, SNRPA, THOC4, SNRNP40, SNRPF, BAT1, PRPF38A, SNRPG, NHP2L1, STRAP, SNRPB2, SF3B5, XAB2, SF3B3, SF3B2, PRPF19, SF3B1, HNRNPK, FRG1, PRPF8, HNRNPC, PABPC1, GEMIN6, DDX41, RBM28, GEMIN4, GEMIN5, SFRS12, DHX8, BCAS2, SNW1, RBMX, SF3A1, SF3A3, PPIE, PPP1R8, PHF5A, CWC22, RBM17	71	8.26	3.55E-46
Active	Cytoplasm	RPL18, KIFC1, LSM6, PPP2R5D, PGD, ASCC1, STOML2, SYNCRIP, VPRBP, RANGAP1, SART3, CIAPIN1, CDCA8, INCENP, WDR77, DHX36, DHX30, TBPL1, YARS, NDNL2, KIF5B, PCM1, NCAPD2, UXT, UNC13D, PTRF, DHX29, RCC2, ASCC3, KIAA1524, RPS12, FLII, MYBBP1A, MYO18A, RAD23B, OSTF1, KIF4A, AHCY, DNAH14, PABPC4, AHCTF1, HSPA1A, DIDO1, SCRIB, C3ORF10, PFN1, CACYBP, PABPC1, NUDC, UNC45A, FH, ZNF622, DHX9, ZC3H15, PCNT, ACACA, RPS9, NXF1, SEPT11, RPS8, FXR1, UPF3B, NOLC1, CRNKL1, QARS, AASDHPPT, DYNLL1, NUBP2, DYNLL2, COPB1, ANP32A, SYMPK, SRPK2, EXOSC8, ARHGEF2, ARHGEF1, EXOSC9, EXOSC6, EXOSC4, ANP32E, TPX2, EXOSC2, EXOSC3, FLNC, FLNB, FLNA, SMN1, SRPK1, EPB41L2, SERPINB9, EIF4A3, SERPINB6, RRM2, CLIC5, TXN, THOC7, USP20, RUVBL2, THOC4, THOC5, RUVBL1, SRP9, THOC1, SNAP29, NACA, EEA1, EZR, CSDE1, LYPLAL1, NMD3, UPF2, AIMP1, EPPK1, AIMP2, ACLY, TRIM25, ETF1, CSNK1D, EIF6, XPO1, SRP14, RPL36A, ILKAP, CHERP, XPO5, CAPZA1, SRP19, VCL, ANK1, SND1, DDX20, API5, ASPM, RPS27A, ACTN4, RELA, MTA1, TOPBP1, EEF2, HNRNPR, HNRNPU, ARPC1B, PA2G4, COPG2, STMN1, UBA52, ARFGAP2, HDLBP, C14ORF166, ARPC4, ANLN, STIP1, HNRNPL, TROVE2, HNRNPK, ARPC2, RPL8, RPL3, RPL5, XPOT, EPB41, NIN, FSCN1, ARID3A, SAFB2, EPS15, NMT1, VCP, TSC2, SPTA1, ABL1, PTGES3, HINT3, HINT1, EDC4, PRDX1, KIF2C, WARS, RPS3A, PRMT5, CDK5RAP2, KIF2A, KIF14, DDB1, SARS, TBCE, NUSAP1, IPO9, NUP85, CDC5L, RPS4X, NCL, PMM2, ZFR, VASP, PPM1G, FAM120A, RIF1, TBCB, TBCA, PRDX6, IPO7, IPO4, IPO5, DDT, UCHL3, MAPRE2, MAPRE1, LCP1, STON2, BCLAF1, FKBP5, FKBP4, HAUS1, RNH1, IARS, CHD1, MYCBP, VPS35, PAFAH1B2, HSPA8, SFRS13A, ANXA1, DRG1, DRG2, COTL1, SMC2, SMC4, PDC, RAB3GAP2, UTRN, SRP68, CCT2, PDLIM1, AURKB, CCT3, CD2AP, MYD88, ATXN10, CLNS1A, TXNL1, BYSL, UBR4, SFRS1, DCTN1, RIC8A, DCTN2, MAD2L1, HUWE1, RARS, SERBP1, SRP72, DBN1, ADD1, TALDO1, IGF2BP3, ACAT2, CAPZB, CDC37, TK1, MOV10, MACF1, CEP170, DMD, S100A11, PPA1, DDX6, CCT7, CCT5, CCT4, CCT8, TMOD3, CHAF1B, ADAR, COPS2, COPS5, COPS6, COPS3, COPS4, VBP1, ACTR3, HSPH1, PARN, TOP2B, DYNC1H1, TOP2A, TFIP11, ABCE1, INPPL1, HERC1, GMPS, GTF2I, FARSB, UROD, PPP6C, CARHSP1, YWHAZ, SHROOM3, MTDH, PPFIA1, FKBP1A, HPRT1, STAU2, MIF, STAU1, SUMO3, SUMO1, SET, CD2BP2, NCAPG, FASN, AP3D1, RBM27, CSNK1A1, UBXN1, GDI2, NASP, YWHAB, ILF3, YWHAE, YWHAG, ILF2, PPP1R8, CIT, PGK1, SEPT2, EIF5B, EIF5A, RPL36AL, PPP1CC, PSMA6, PSMA5, PSMA3, CSTB, ERC1, SEPT7, DST, ANKFY1, SEPT9, STRAP, DIAPH1, COPZ1, COPS7B, KEAP1, UBAC1, PSMA7, EIF3C, EIF3D, EIF3A, EIF3B, CSE1L, EIF3G, EIF3H, PELP1, PSMB3, MTPN, EIF3E, EIF3F, EIF3K, EIF3L, CLASP1, EIF3J, STRBP, CLASP2, EIF3M, PTPN2, ABHD14B, RNPS1, TSN, CORO1B, PSMC5, TXNDC17, PSMC4, PSMC3, PSMC2, PSMC1, SPTBN2, SPTBN1, CYFIP1, WDR1, PHPT1, PDCD6, COPA, TLN1, OSBP, TLN2, PDCD4, SBDS, NUDT16L1, ACOT13, MSN, KPNB1, ZW10, ELP3, G3BP2, TARS, SMU1, BPTF, ARCN1, CFL1, PSME3, KPNA4, KPNA3, COPG, KPNA2, KPNA1, COPE, GLRX3, USP9X, HCFC1, TPM3, EXOSC10, PPP1R12A, GEMIN6, TNPO1, GEMIN4, GEMIN5, ENO1, TNPO3, TCP1, CENPF, TUBGCP2, PARK7, DIS3, GPI, PSMG1, PSPC1, CENPV, NGDN, SPTAN1	430	1.80	8.36E-37
Active	Ribosome	RPL18, RPL17, RPL19, RPL13, RPLP2, RPS2, RPS3A, FAU, RPL10, RPS27A, RPL36AL, RPL35A, MRPL3, RPS4X, RPS15, RPS12, RPS13, UBA52, RPL36, RPS15A, RPS25, RPS26, RPL30, RPS28, RPS29, RPL32, RPL7, RPL6, RPL31, RPL9, RPL8, RPL3, RPL5, RPL7A, RPL4, RPS21, RPS23, RPS24, RPL26, RPS9, RPL27, RPL24, RPS6, RPS8, RPS7, RPL29, RPL13A, RPL21, RPL37A	49	9.38	9.87E-35
Active	rRNA processing	GAR1, UTP18, LSM6, UTP6, UTP15, BOP1, FCF1, DIMT1L, EXOSC10, DCAF13, IMP3, UTP11L, DKC1, FRG1, TFB2M, WDR12, IMP4, FTSJ3, NSA2, KRR1, PDCD11, EXOSC8, EXOSC9, RRP1, EXOSC6, EXOSC7, EMG1, EXOSC4, EXOSC5, MPHOSPH10, EXOSC2, EXOSC3, HEATR1, RRP9, NOP10, RPF1, FBL, DIS3, NOP14, EIF4A3, DDX56, PA2G4, PES1, NHP2, DDX51	45	10.30	1.75E-34
Active	Ribosome biogenesis	SDAD1, GAR1, BOP1, FCF1, BMS1, DCAF13, EBNA1BP2, IMP3, SBDS, DKC1, FRG1, WDR12, RSL24D1, GNL2, IMP4, NSA2, KRR1, GTPBP4, TSR1, EMG1, NIP7, BYSL, MPHOSPH10, HEATR1, NOP10, RPF1, MRTO4, NOP14, RCL1, DDX56, RRS1, NOP58, NOP56, PES1, UTP14A, NHP2, DDX51	37	10.77	5.86E-29
Active	Helicase	XRCC6, SKIV2L2, DDX27, DDX18, DDX23, DHX38, DHX37, DDX24, DHX36, DDX21, DDX10, DDX20, DHX30, DDX39, EIF4A3, RECQL, DHX29, ASCC3, SNRNP200, EIF4A1, SMARCA5, RUVBL2, RUVBL1, EP400, SMARCA4, BAT1, BLM, MOV10, DDX47, DDX46, CHD7, DDX49, DHX15, DHX16, CHD1, SUPV3L1, DDX41, ERCC3, CHD4, DDX42, ERCC2, DHX8, DHX9, YTHDC2, WRN, DDX6, DDX56, GTF2F2, DDX50, DDX54, DDX52, DDX51	52	5.42	7.15E-22

Supplementary Table 2 (cont.) | Functional enrichment analysis of proteins differentially enriched in integrin adhesion complexes.

Cluster[1]	Term[2,3]	Proteins[4]	Count (proteins per term)	Fold enrichment	Corrected *P* value
Active	**Mitosis**	KIFC1, SEPT2, AURKB, CD2AP, KIF2C, CDCA8, RAD21, SEH1L, INCENP, DYNC1H1, STAG2, ASPM, ZW10, ANAPC1, ARHGEF2, KIF11, ANAPC5, NUSAP1, UBE2I, CDK2, NCAPD3, NCAPD2, MAD2L1, RCC2, TIMELESS, MAPRE2, ANAPC7, RUVBL1, MAPRE1, NUP43, SEPT7, USP9X, HAUS1, ANLN, SPC24, NUMA1, C12ORF11, NCAPG, NCAPG2, CLASP1, CLASP2, NUDC, CSNK1A1, SSSCA1, PDS5B, PDS5A, CKAP5, NUF2, CENPF, CDC23, PMF1, SMC2, SMC3, SMC4, PLK1, CENPV, SMC1A, CIT, TXNL4A	**59**	**4.50**	**2.26E-20**
Active	**Cell division**	KIFC1, SEPT2, CCNT1, AURKB, CD2AP, KIF2C, CDCA8, RAD21, ATAD3B, SEH1L, INCENP, STAG2, ASPM, ZW10, ANAPC1, ARHGEF2, KIF11, ANAPC5, LIG1, LIG3, NUSAP1, UBE2I, CDK7, PPP1CC, NCAPD3, CDK2, NCAPD2, MAD2L1, TIMELESS, RCC2, MAPRE2, MAPRE1, ANAPC7, RUVBL1, NUP43, SEPT7, SEPT9, CKS1B, USP9X, HAUS1, AHCTF1, ANLN, SPC24, C12ORF11, NCAPG, NCAPG2, CLASP1, CLASP2, NUDC, CSNK1A1, SSSCA1, PDS5B, PDS5A, CKAP5, NUF2, CDC23, CENPF, PMF1, SMC2, SMC3, SEPT11, SMC4, PLK1, CENPV, CKS2, SMC1A, CIT, TXNL4A	**68**	**3.60**	**8.56E-18**
Active	Cell cycle	KIFC1, SEPT2, CCNT1, AURKB, WTAP, CD2AP, KIF2C, CDCA8, RAD21, SEH1L, FANCI, INCENP, H2AFX, STAG2, ASPM, ZW10, ANAPC1, ARHGEF2, RBBP4, KIF11, ANAPC5, CCNH, LIG1, LIG3, NUSAP1, UBE2I, CDK7, CDC5L, MCM2, PPP1CC, RAD50, NCAPD3, CDK2, ESCO2, NCAPD2, MAD2L1, TIMELESS, RCC2, RIF1, MAPK3, MAPRE2, PSME3, MAPRE1, RUVBL1, ANAPC7, SEPT7, NUP43, SEPT9, PPP6C, CKS1B, USP9X, HAUS1, HCFC1, AHCTF1, BCCIP, ANLN, CDC73, PIN1, SPC24, NIPBL, C12ORF11, SMARCB1, MDC1, NCAPG, NCAPG2, CLASP1, CLASP2, NUDC, CSNK1A1, SSSCA1, ERH, PDS5B, MKI67, PDS5A, CKAP5, NASP, NUF2, CDC23, CENPF, PMF1, SMC2, SMC3, SEPT11, SMC4, PLK1, CKS2, CENPV, SMC1A, CIT, CHAF1B, TXNL4A	91	2.76	4.27E-16
Active	Mitochondrion	MRPS36, MRPS34, OGDH, COX5A, PDHB, GOT2, PGAM5, RPL10, MRPL37, TFB2M, MRPL39, PDHA1, DHX30, MRPL53, LYRM4, PECI, CLPX, COX6C, PYCR1, PTRF, DLD, ATP5C1, ATPIF1, YARS2, MRPL48, MRPL45, LRPPRC, MDH2, MRPL43, MRPS16, GLUD1, MRPS11, PNPT1, BCL2L1, PTRH2, HADHA, HADHB, COX6B1, MRPL55, HSPE1, SUPV3L1, FH, MRPS27, MRPS26, ATP5J2, MRPS28, MRPS23, MRPS22, MRPS21, DLAT, AFG3L2, VDAC2, IDH3A, PPIF, CKMT1A, USMG5, PTCD3, NDUFV1, NDUFV2, DAP3, DUT, UQCRC2, UQCRC1, ATP5B, CYC1, RG9MTD1, PRDX3, UQCRQ, ACOT9, MTHFD2, LONP1, NDUFS8, ACOT13, ATP5L, SLC25A1, ATP5I, NDUFS3, NDUFS1, DNAJC19, MRPL2, MRPL1, ABCE1, MRPL3, ACO2, AIFM1, SLC25A6, CYCS, MRPS5, MRPS6, MRPS7, MRPS2, TRAP1, C1QBP, ALDH1B1, PMPCA, OAT, TUFM, NDUFB4, ALDH18A1, ECH1, NDUFB9, TIMM16, TIMM13, DCI, MRPL11, TFAM, MRPL13, MRPL15, MRPL14, MRPL17, MRPL18, MYCBP, ETFB, HSPA9, DLST, NDUFA5, NDUFA2, SHMT2, NDUFA3, IMMT, NDUFA6, NDUFA7, CS, ATP5F1, SDHA, ACSM3, MRPL23, MRPL22, MRPL27, TSFM, ATP5A1	131	2.20	3.09E-15
Active	ATP-binding	KIFC1, CLPB, XRCC6, CCT2, AURKB, CCT3, CSNK2A2, DDX27, TOP1, CSNK2A1, DDX18, DDX23, DHX38, DHX37, CLP1, DDX24, DHX36, DDX21, DDX20, DDX10, DHX30, YARS, BCR, KIF5B, LIG1, LIG3, OLA1, MYH9, HNRNPU, CLPX, MAP4K4, DDX39, RFC3, RFC4, RFC1, DHX29, BAZ1B, ASCC3, RARS, MAPK3, SMARCA5, YARS2, EIF2AK2, MYO18A, EP400, BAT1, SMARCA4, NMNAT1, KIF4A, BLM, DNAH14, GLUD1, HSPA1A, MAPKAPK2, TK1, HSPA1L, VRK1, MOV10, DDX47, DDX46, DDX49, SUPV3L1, NAT10, DDX41, DDX42, DHX8, DHX9, MKI67, NIN, MYO1D, ACACA, YTHDC2, WRN, AFG3L2, UBE2L3, DDX6, GART, CCT7, CKMT1A, DDX56, CCT5, CCT4, PSMC5, PSMC4, NOLC1, VCP, PLK1, PSMC3, PSMC2, CCT8, PSMC1, GTF2F2, DDX50, HSPA14, ABL1, DDX54, SMC1A, DDX52, MYH10, DDX51, ABCF1, KIF22, ABCF2, ATP5B, SKIV2L2, QARS, ACTR3, KIF2C, WARS, HSPH1, ATAD3A, LONP1, ATAD3B, NUBP2, TOP2B, DYNC1H1, MDN1, TOP2A, KIF2A, KIF14, SRPK2, ABCE1, KIF11, SARS, PI4KA, CDK9, UBE2I, MCM2, CDK7, PBK, GMPS, RAD50, CDK2, SRPK1, TRAP1, TARS, EIF4A3, RECQL, EIF4A1, SNRNP200, FARSB, ORC5L, RUVBL2, RUVBL1, TOP3B, DNAH9, ALDH18A1, WRNIP1, PRKDC, BMS1, IARS, CHD7, DHX15, DHX16, CHD1, HSPA5, ERCC3, EHD1, CHD4, HSPA8, ERCC2, HSPA9, CSNK1A1, MSH6, TCP1, SMC5, KIF18B, SMC6, ACLY, SMC2, SMC3, SMC4, ACSM3, CSNK1D, THRAP3, ATP5A1, PGK1, CIT, PAICS	179	1.89	1.50E-14
Active	Initiation factor	EIF6, EIF5, EIF5B, EIF2A, EIF3C, EIF3D, EIF3A, EIF3B, EIF3G, EIF3H, EIF1AX, EIF3E, EIF3F, EIF3K, EIF3L, EIF1, EIF3J, EIF2B4, EIF3M, EIF2B5, EIF2S3, EIF2B1, EIF4B, DHX29, EIF2S1, EIF2S2, EIF4A1	27	7.25	5.90E-14
Active	**mRNA transport**	ENY2, XPO1, NUP160, EIF5A, AHCTF1, NUP93, NUP188, SEH1L, NUP210, NUP50, RANBP2, TPR, NUP133, NUP153, G3BP2, NUP85, NXF1, NUP155, EIF4A3, UPF3B, NUP205, THOC7, THOC4, THOC5, NUP107, THOC2, NUP43, LRPPRC, THOC1, BAT1	30	5.66	3.24E-12
Active	**Chaperone**	PTGES3, FKBP5, VBP1, CCT2, HSPA1A, TIMM13, CCT3, CANX, CDC37, DNAJC9, BAG2, DNAJC8, NPM1, NPM3, HSPE1, ASF1B, DNAJC2, ASF1A, MDN1, DNAJC19, HSPA8, HSPA9, UNC45A, ABCE1, TCP1, TBCE, RBBP7, PARK7, CLPX, PSMG3, CCT7, TRAP1, PFDN2, PSMG1, CCT5, PFDN1, CCT4, PSMG2, TBCB, TBCA, PFDN6, CCT8, THOC4	43	3.80	2.55E-11
Active	Transit peptide	UQCRC2, UQCRC1, ATP5B, CYC1, RG9MTD1, PRDX3, COX5A, OGDH, PDHB, ACOT9, GOT2, MTHFD2, LONP1, NDUFS8, MRPL37, TFB2M, SLC25A1, PDHA1, NDUFS3, NDUFS1, MRPL53, MRPL2, MRPL1, ACO2, AIFM1, MRPS7, PECI, CLPX, TRAP1, C1QBP, ALDH1B1, DLD, ATP5C1, ATPIF1, YARS2, MRPL48, MRPL45, OAT, PMPCA, LRPPRC, MDH2, MRPL43, TUFM, MRPS16, ECH1, GLUD1, MRPS11, PNPT1, PTRH2, DCI, HADHA, HADHB, MRPL11, TFAM, MRPL15, MRPL14, MRPL17, MRPL55, MRPL18, SUPV3L1, FH, HSPA9, DLST, MRPS26, SHMT2, MRPS28, CS, ATP5F1, DLAT, IDH3A, SDHA, PPIF, ACSM3, CKMT1A, MRPL22, PTCD3, TSFM, NDUFV1, NDUFV2, ATP5A1, DAP3, DUT	82	2.41	1.02E-10
Active	Nucleotide-binding	KIFC1, XRCC6, CCT2, CCT3, AURKB, DDX18, DHX38, DHX37, DHX36, DDX10, DHX30, GTPBP4, YARS, BCR, KIF5B, LIG1, LIG3, EIF2S3, OLA1, MYH9, RFC3, RFC4, BAZ1B, DHX29, RFC1, ASCC3, RARS, MAPK3, EIF2AK2, MYO18A, KIF4A, DNAH14, HSPA1A, TK1, HSPA1L, MOV10, VRK1, NAT10, SUPV3L1, DHX8, DHX9, MKI67, ACACA, YTHDC2, AFG3L2, UBE2L3, VDAC2, SEPT11, DDX6, GART, CCT7, CCT5, CKMT1A, CCT4, NOLC1, CCT8, HSPA14, ABCF1, ABCF2, ATP5B, QARS, ACTR3, HSPH1, ATAD3A, LONP1, ATAD3B, NUBP2, SRPR, TOP2B, DYNC1H1, TOP2A, ABCE1, SRPK2, PI4KA, GMPS, RAD50, SRPK1, TRAP1, EIF4A3, EIF4A1, RAB5A, FARSB, RUVBL2, RUVBL1, TOP3B, TUFM, ALDH18A1, WRNIP1, BMS1, DHX15, DHX16, CSNK1A1, ACLY, ACSM3, CSNK1D, ATP5A1, CIT, PGK1, PAICS, SEPT2, CLPB, EIF5, HBS1L, EIF5B, CSNK2A2, DDX27, TOP1, CSNK2A1, DDX23, CLP1, DDX24, DDX21, DDX20, GNL2, GNL3, EFTUD2, EEF2, HNRNPU, CLPX, MAP4K4, DDX39, SMARCA5, YARS2, SEPT7, EP400, SEPT9, BAT1, NMNAT1, SMARCA4, BLM, GLUD1, MAPKAPK2, DDX47, DDX46, DDX49, DDX41, DDX42, RAB8B, NIN, MYO1D, WRN, DDX56, PSMC5, PSMC4, VCP, PLK1, PSMC3, PSMC2, GTF2F2, PSMC1, DDX50, DDX54, SMC1A, ABL1, DDX52, DDX51, MYH10, KIF22, SKIV2L2, WARS, KIF2C, MDN1, KIF2A, KIF14, KIF11, SARS, CDK9, UBE2I, SRPRB, CDK7, MCM2, PBK, CDK2, RECQL, TARS, SNRNP200, ORC5L, DNAH9, PRKDC, IARS, CHD7, CHD1, HSPA5, ERCC3, EHD1, CHD4, HSPA8, ERCC2, HSPA9, MSH6, TCP1, SMC5, KIF18B, SMC6, DRG1, DRG2, SMC2, SMC3, SMC4, GSPT1, THRAP3	201	1.67	1.06E-10
Active	Isopeptide bond	ABCF1, HIST4H4, SYNCRIP, VPRBP, RANGAP1, OGDH, UQCRQ, HIST1H2BO, HIST2H2AB, TOP1, H2AFV, FANCI, RPL10, H2AFY, H2AFX, RPN2, RANBP2, ACIN1, HIST3H2BB, PSMD7, KPNB1, RPS27A, CUL1, KIF14, RBBP4, ACO2, ACTN4, HIST1H1C, LIG1, KTN1, UBE2I, RBBP7, TARS, PSMA6, SNRNP200, CPSF3, DST, UBA52, HMGB1, HIST1H2AG, DIAPH1, WRNIP1, SUMO3, PFN1, SUMO1, SET, HNRNPK, NPM1, HNRNPC, RBM27, FN1, MAFG, HIST1H2BH, ANXA1, TRIM25, PARK7, RPL29, HDAC3, CCT8, PSMC1, PCNA, SSR4, ADAR	63	2.76	1.99E-10
Active	**Cytoskeleton**	KIFC1, SEPT2, CAPZA1, UTRN, STOML2, PDLIM1, RANGAP1, AURKB, CD2AP, VCL, CDCA8, ANK1, INCENP, CLNS1A, KIF5B, UBR4, TOPBP1, PCM1, DCTN1, DCTN2, ARPC1B, UXT, RCC2, FLII, SEPT7, DST, MYO18A, ADD1, SEPT9, KIF4A, DIAPH1, DNAH14, ANLN, ARPC4, C14ORF166, CAPZB, C3ORF10, PFN1, MACF1, ARPC2, CEP170, DMD, CLASP1, CLASP2, NUDC, NIN, EPB41, PCNT, SEPT11, CORO1B, SPTBN2, TMOD3, SPTBN1, SPTA1, WDR1, ABL1, TLN1, TLN2, ACTR3, KIF2C, NUBP2, CDK5RAP2, MSN, KIF2A, ZW10, KIF14, SYMPK, ARHGEF2, INPPL1, TPX2, TBCE, NUSAP1, NUP85, FLNC, FLNB, VASP, FLNA, EPB41L2, RIF1, TBCB, TBCA, CLIC5, CFL1, MAPRE1, RUVBL1, LCP1, SHROOM3, HAUS1, TPM3, EZR, CSNK1A1, EPPK1, CENPF, COTL1, TUBGCP2, CENPV, SPTAN1	**97**	**2.13**	**9.77E-10**
Active	Host-virus interaction	XPO1, COPS6, VAPB, CCNT1, SYNCRIP, VPRBP, APOBEC3C, TOP1, SND1, COPB1, GTF2A2, PSIP1, KPNB1, ABCE1, RELA, DDB1, SLC25A6, UBR4, UBE2I, SPEN, HMGA1, SRPK1, C1QBP, IPO7, MAPK3, IPO5, PSMA3, THOC4, EIF2AK2, KPNA2, KPNA1, USP7, POLR2E, NACA, GRB2, POLA1, HCFC1, PSMA7, SF3B2, HNRNPK, SMARCB1, PSMB3, NPM1, ERCC3, TNPO1, HSPA8, ERCC2, CCDC86, TRIM25, NXF1, YWHAE, FBLN1, PSMC3, PSMC2, TCEB1, RBM15	56	2.74	5.72E-09
Active	Proteasome	RAD23B, PSMA7, PSMD14, PSMD13, PSMC5, PSMA6, PSMD12, PSMC4, PSMA5, PSMC3, PSMD11, PSMB3, PSMC2, PSMD1, PSMC1, PSMA3, PSMD2, PSMD3, PSMD4, PSME3, PSMD5, PSMD6, PSMD7	23	5.74	5.74E-09
Active	DNA replication	BLM, WRNIP1, POLA1, RPA3, RPA1, PRIM1, TOP1, PRIM2, GINS1, SSRP1, RBBP4, NASP, LIG1, GINS3, POLE, LIG3, POLB, MCM2, RBBP7, RFC3, ORC3L, RFC4, RFC1, RRM2, SUPT16H, PCNA, ORC5L, CHAF1B	28	4.44	2.32E-08
Active	DNA damage	MORF4L1, RAD23B, MRE11A, WRNIP1, XRCC6, PRKDC, BCCIP, INTS3, XRCC1, XAB2, PRPF19, RAD21, MDC1, FANCI, NSMCE1, H2AFX, ERCC3, APEX1, ERCC2, MSH6, SSRP1, DDB1, LIG1, POLE, LIG3, SMC5, GTF2H4, GTF2H3, SMC6, POLB, TOPBP1, CDK7, RAD50, SMC3, GTF2H2, MPG, RIF1, BAZ1B, TIMELESS, SFPQ, SUPT16H, SMC1A, PARP1, CHAF1B	44	3.00	6.58E-08
Active	**Kinetochore**	CSNK1A1, NUP133, NUF2, CENPF, AHCTF1, NUP85, RANGAP1, PMF1, SPC24, KIF2C, MAD2L1, SEH1L, PLK1, CENPC1, INCENP, CENPV, CLASP1, CLASP2, NUP107, SMC1A, NUP43, SEPT7, ZW10	**23**	**5.10**	**8.68E-08**
Active	Ubl conjugation	ABCF1, KIF22, HIST4H4, SYNCRIP, VPRBP, RANGAP1, XRCC1, OGDH, UQCRQ, SART1, HIST1H2BO, TOP1, HIST2H2AB, CDCA8, H2AFV, FANCI, RPL10, H2AFY, H2AFX, RPN2, ACIN1, RANBP2, HIST3H2BB, PSMD7, KPNB1, CUL1, RPS27A, KIF14, RBBP4, ACO2, ACTN4, HIST1H1C, LIG1, DDB1, RELA, NUSAP1, KTN1, TOPBP1, UBE2I, RBBP7, DCTN1, TARS, PSMA6, TBCB, GTF2I, SNRNP200, USP20, CPSF3, DST, UBA52, USP7, HMGB1, HIST1H2AG, DIAPH1, WRNIP1, ANLN, KEAP1, SCRIB, CDC37, SUMO3, PFN1, SET, HNRNPK, NPM1, HNRNPC, RBM27, MAFG, SSRP1, AIMP2, HIST1H2BH, ZNF24, SMC6, TRIM25, DRG1, DRG2, PARK7, RPL29, HDAC3, PSMC3, CCT8, PSMC1, PCNA, DNMT1, SSR4, PES1, ADAR	86	2.04	1.76E-07
Active	Protein transport	COPA, XPO1, AP1B1, XPO5, AP1G1, AP2S1, EIF5A, AP3S1, NUP188, AP2B1, SEH1L, COPB1, RANBP2, TPR, KPNB1, DNAJC19, ZW10, AP3B1, NUP133, ACTN4, NUP85, IPO9, COPG2, SEC61B, IPO7, IPO4, NUP205, IPO5, ARCN1, RAB5A, KPNA6, KPNA4, NUP107, KPNA3, COPG, ERC1, KPNA2, NUP43, COPE, KPNA1, SNAP29, SNX9, ARFGAP2, SDAD1, NACA, NUP160, NAPG, TIMM16, COPZ1, NUP93, AHCTF1, TIMM13, SNX3, CSE1L, AP3M1, NUP210, NUP50, AP3D1, SEC22B, EXOC3, VPS35, SNAP23, TNPO1, SEC61A1, TNPO3, NMD3, AP2M1, NUP153, RAB8B, RRBP1, NASP, NUP155, EPS15, AP2A2, AP2A1	75	2.16	2.24E-07
Active	Chromatin regulator	UTP3, ENY2, MORF4L1, RSF1, EZH2, CBX3, TRRAP, ARID2, DPY30, CHD7, SMARCD2, SMARCB1, PRMT5, SMARCD1, EED, H2AFY, CHD1, PBRM1, ACTL6A, ASF1B, BAZ2A, ASF1A, MYST4, CHD4, MLL2, RBBP5, RBBP4, RING1, HMG20A, ARID1A, RBBP7, SUZ12, HDAC3, HDAC2, BPTF, SMARCE1, SMARCC1, SMARCC2, SMARCA5, DNMT1, RUVBL2, RUVBL1, EP400, SMARCA4	44	2.89	2.36E-07
Active	DNA repair	MORF4L1, RAD23B, MRE11A, XRCC6, PRKDC, BCCIP, INTS3, XRCC1, XAB2, PRPF19, RAD21, MDC1, FANCI, NSMCE1, H2AFX, ERCC3, APEX1, ERCC2, MSH6, SSRP1, DDB1, LIG1, POLE, LIG3, GTF2H4, SMC5, GTF2H3, SMC6, POLB, TOPBP1, CDK7, RAD50, SMC3, GTF2H2, MPG, RECQL, SFPQ, SUPT16H, SMC1A, PARP1, CHAF1B	41	3.00	3.05E-07
Active	WD repeat	COPA, UTP18, UTP15, EDC4, EIF2A, WDR75, DCAF13, WDR36, PLRG1, SEH1L, WDR77, EED, CIRH1A, WDR33, RBBP5, RBBP4, RRP9, HERC2, RBBP7, HERC1, PRPF4, ARPC1B, SMU1, AAAS, NLE1, NOL10, SNRNP40, NUP43, WDR43, WDR46, PPP2R2A, STRAP, LRBA, BOP1, PRPF19, WDR18, EIF3B, WDR12, WDHD1, GEMIN5, TBL3, TBL2, PAK1IP1, WDR70, PWP2, CORO1C, CORO1B, WDR3, WDR1, CHAF1B, CSTF1	51	2.58	4.97E-07
Active	**RNA binding**	SRP14, CSTF2, SNRPB2, RNPS1, SSB, NCL, FXR1, EIF4B, EIF4A3, HNRNPK, TAF15, EIF4A1, SNRPA, RPS27A, UBA52	15	7.48	7.08E-07
Active	Viral nucleoprotein	SNRPA1, EFTUD2, SNRPB2, MPHOSPH10, SNRPD1, SYNCRIP, SSB, RBMX, HNRNPR, HNRNPU, HNRNPL, HNRNPK, SNRPB, IMP4, AHNAK	15	7.23	1.28E-06
Active	Isomerase	PTGES3, ECH1, FKBP5, FKBP4, FKBP3, PPIL2, PDIA5, FKBP1A, DCI, MIF, PIN1, TOP1, TPI1, DKC1, PPIL4, HSD17B4, RPIA, RANBP2, TOP2B, TOP2A, CWC27, TOPBP1, PMM2, PECI, PPIF, GPI, PPIE, PPIG, IDI1, TOP3B	30	3.49	2.32E-06
Active	Nuclear pore complex	NUP133, NUP153, NUP160, AHCTF1, NUP93, EIF5A, NUP188, NUP85, NUP155, SEH1L, NUP205, NUP210, NUP50, RANBP2, NUP107, TPR, NUP43	17	5.94	2.78E-06
Active	Translocation	NUP133, NUP153, NUP160, RRBP1, TIMM16, AHCTF1, NUP93, EIF5A, NUP188, NUP85, TIMM13, NUP155, SEC61B, SEH1L, NUP205, NUP210, NUP50, NUP107, RANBP2, TPR, NUP43, SEC61A1, DNAJC19	23	4.34	2.80E-06
Active	**Actin-binding**	SHROOM3, DIAPH1, CAPZA2, UTRN, CAPZA1, ANLN, ARPC4, CAPZB, VCL, TPM3, ACTR3, PFN1, MACF1, ARPC2, DMD, CAP1, EMD, EPB41, ACTN4, INPPL1, MYO1D, FSCN1, MYH9, COTL1, FLNC, FLNB, VASP, FLNA, CORO1C, EPB41L2, ARPC1B, CORO1B, CFL1, SPTBN2, FLII, SPTBN1, TMOD3, CYFIP1, WDR1, SPTA1, DBN1, DST, LCP1, MYH10, SPTAN1, ADD1	**46**	**2.60**	**2.85E-06**
Active	Exosome	DIS3, EXOSC10, EXOSC8, EXOSC9, EXOSC6, EXOSC7, EXOSC4, EXOSC5, EXOSC2, EXOSC3	10	11.64	4.59E-06
Active	Chromosomal protein	HMGB1, HMGB2, HIST4H4, HMGB3, HIST1H2AG, HP1BP3, XRCC6, H1FX, AURKB, HIST1H2BO, HIST2H2AB, KIF2C, CDCA8, H2AFV, FANCI, CENPC1, H2AFY, H2AFX, HIST3H2BB, SSRP1, HIST1H1E, HIST1H1C, HIST1H1B, HIST1H2BH, SMC5, CENPF, SMC6, HMGA1, RAD50, RIF1, SUPT16H, TMPO	32	3.08	1.56E-05
Active	Respiratory chain	UQCRC2, NDUFB4, NDUFA5, NDUFA2, NDUFA3, UQCRC1, NDUFB9, NDUFA6, CYC1, CYCS, NDUFA7, COX5A, UQCRQ, COX6C, NDUFV1, NDUFS8, NDUFV2, COX6B1, NDUFS3, NDUFS1	20	3.93	1.93E-04
Active	P-loop	TUFM, KIFC1, ABCF2, ATP5B, TK1, LONP1, DDX18, SRPR, DDX21, DYNC1H1, DDX42, DHX9, ABCE1, KIF11, KIF5B, EIF2S3, EEF2, HNRNPU, DDX6, RECQL, EIF4A3, RFC1, EIF4A1, RAB5A, ATP5A1, SEPT7	26	3.13	2.83E-04
Active	DNA-directed RNA polymerase	POLR2G, POLR2E, POLR1E, POLR1D, POLR2J, POLR1A, POLR3A, POLR1C, POLR1B, POLR2C, POLR3B, POLR2B, PRIM1, PRIM2	14	5.29	4.52E-04
Active	Nucleotide binding	TUFM, KIFC1, ABCE1, KIF11, KIF5B, ATP5B, EIF2S3, EEF2, HNRNPU, TK1, DDX6, EIF4A3, RECQL, LONP1, DDX18, RFC1, SRPR, EIF4A1, RAB5A, DDX21, ATP5A1, DYNC1H1, SEPT7, DDX42	24	3.13	8.35E-04
Active	**Actin binding**	UTRN, FLNC, FLNA, ANXA2, TPM3, PFN1, EZR, DMD, CFL1, SPTBN1, SPTA1, MSN, LCP1, SPTAN1	**14**	**4.89**	**1.25E-03**
Active	**Microtubule**	KIFC1, KIF22, DNAH9, KIF4A, SHROOM3, DNAH14, HAUS1, ARPC4, STAU2, KIF2C, DYNLL1, DYNLL2, INCENP, CEP170, CLASP1, CLASP2, DYNC1H1, EMD, NUDC, KIF2A, KIF14, ARHGEF2, KIF11, KIF5B, NIN, NUSAP1, KIF18B, DYNLT1, TUBGCP2, DCTN1, DCTN2, TBCB, RCC2, TBCA, MAP4, MAPRE2, STMN1, MAPRE1	**38**	**2.30**	**1.66E-03**
Active	Exonuclease	EXOSC8, EXOSC9, EXOSC6, EXOSC7, MRE11A, EXOSC4, EXOSC5, EXOSC2, EXOSC3, DIS3, EXOSC10, PARN, REXO4, FEN1	14	4.55	3.10E-03
Active	Mitochondrion inner membrane	UQCRC2, NDUFB4, ALDH18A1, UQCRC1, ATP5B, TIMM16, NDUFB9, CYC1, TIMM13, COX5A, UQCRQ, ATP5L, SLC25A1, NDUFS3, ATP5I, NDUFS1, DNAJC19, NDUFA5, NDUFA2, ATP5J2, NDUFA3, IMMT, SLC25A6, NDUFA6, NDUFA7, ATP5F1, COX6C, SDHA, CKMT1A, NDUFV1, NDUFV2, ATP5C1, ATP5A1	33	2.39	3.62E-03
Active	Methylation	RPL36A, HIST4H4, HIST1H2AG, TRA2B, SNRPD3, PABPC4, SNRPD1, HIST1H2BO, HIST2H2AB, HNRNPK, H2AFY, PABPC1, HIST3H2BB, SUPT5H, RPL36AL, DHX9, HIST1H1E, RAB8B, HIST1H1C, HIST1H2BH, G3BP2, ILF3, POLB, SFRS1, HMGA1, RBMX, NCL, FBL, HNRNPU, RPL29, FXR1, FAM120A, TAF15, SFPQ, THRAP3, SNRPB, THOC4, UBA52	38	2.19	4.97E-03
Active	**Molecular chaperone**	LONP1, TCP1, CCT8, HSPE1, HSPA1A, CCT3, CANX, HSPA8	8	8.60	5.84E-03
Active	Electron transport	UQCRC2, TXNL1, NDUFB4, NDUFA5, NDUFA2, NDUFA3, UQCRC1, NDUFA6, NDUFB9, NDUFA7, CYCS, CYC1, UQCRQ, SDHA, NDUFV1, TXN, NDUFS8, NDUFV2, NDUFS3, ETFB, NDUFS1	21	2.93	1.21E-02
Active	**Pre-mRNA splicing**	SFRS4, SFPQ, SNRPB2, SNRPA, SFRS2IP, SF3A1	6	11.97	1.82E-02
Active	Rotamase	PPIF, PPIE, PPIG, FKBP5, FKBP4, CWC27, FKBP3, PPIL2, PPIL4, FKBP1A, RANBP2, PIN1	12	4.53	1.91E-02
Active	Chromosome partition	KIF2C, PPP2R1A, RAD21, SEH1L, USP9X, INCENP, UBE2I, PMF1, NUP43, SRPK1, STAG2	11	4.96	1.97E-02
Active	Systemic lupus erythematosus	TROVE2, SNRPD3, SNRPB2, XRCC6, SNRPD1, SNRPB, PCNA, SNRPD2, SSB, PSME3	10	5.17	3.58E-02
Inactive	Acetylation	CTPS, DTYMK, TUBB2C, UBQLN4, SAE1, UBQLN2, ACSS2, RNF214, UBQLN1, RAB1A, ACTG1, CUL2, NANS, CISD1, RAVER2, SRRM2, RAVER1, DNAJC7, VPS4A, OGT, PUM2, NQO1, RBM12, TOR1AIP2, CAPNS1, AARS, YTHDF2, DCTN3, ALDH16A1, STK4, JUP, UHRF1, EP300, TIAL1, ZNHIT2, RAB14, RNF138, TUBA4A, MAPK8, AKAP8, ARL8B, RAB10, BAT2, CRTC2, ADSS, FN3K, HMGCS1, UBA6, ITGB1, CMPK1, PXN, DAZAP1, HNRNPM, DOCK2, SFRS15, TUBB, RAC2, METTL1, UBAP2L, RUNX1, DVL2, EEF1A1, LCMT1, NPLOC4, C6ORF130, AKAP8L, CSRP1, LARP4B, HNRNPH3, HNRNPH2, EIF4E, HEATR2, UBA1, UBA2, POLD2, ASNA1, XPO7, NCOR2, DCXR, TSG101, PRKAG1, ESD, FUBP1, MTHFD1, CDC42, FUBP3, MCM7, SLC25A3, RANBP1, TUBA1C, DCAF16, TUBB3, TBL1XR1, PFKL, ACTA1, ACTA2, PFKP, SF1, IRF2BP2, IRF2BP1, METTL2B, CDK5, PRKCB, UBE2N, LAP3, PANK4, UMPS, EIF4G3, G6PD, LARP4, NCOA3, GNB1, DEF6, PKM2, UBE2M, RRM1, KHSRP, CARM1, METTL11A, DCD, NUP98, KRT6B, USP5, ADH5, UGDH, ASNS, NSUN6, CBR1, ATXN2L, ACSL1, ATIC, SEC16A, RAB11A, MSI2, BDH2, BCL9L, TSTA3, KDM3B, ACSL4, BUB3, TRIP13, EHD4, DUS3L, RBM22, EIF4ENIF1, SAMHD1, ITPR2, CAPN1, APRT, CCNB1, ITPA, RNF114, HNRNPUL1, RAB35, DPM1, TUBAL3, CUL4B	157	2.66	2.31E-30
Inactive	Phosphoprotein	PDLIM7, TUBB2A, TUBB2C, PTPN23, RNF214, RAB1A, ACTG1, CUL2, FAM125A, APOE, RAVER2, RAVER1, ILK, OGT, PITX1, RAB27A, SYK, CIZ1, AARS, MED12, SKP2, KRT10, JUP, PNKP, UHRF1, SCYL2, RAB14, ZNHIT2, TUBA4A, MAPK8, RAB13, PIAS1, SIP1, GIGYF2, CRTC2, COASY, GNAI3, PFKFB4, MOB2, HMGCS1, IGF2BP2, MST4, PXN, SFRS15, ARIH2, TCF20, GORASP2, POTEF, TCF3, SEC23IP, DVL2, DVL3, IKZF1, OXSR1, CSRP1, LARP4B, METTL13, PKP1, ARF1, UBA1, PKP3, UBA2, RAD54L2, LGTN, TCF12, NCOR2, HTATIP2, TSG101, CPEB4, HELZ, COPS8, FAM195A, CKB, RNGTT, MTHFD1, RBM4B, SMAP2, AES, SLC25A3, TUBB6, QKI, TUBB3, TUBB4, TOMM34, FUNDC2, ZCCHC11, ACTA1, ZC3H7A, ACTA2, SF1, METTL2B, G6PD, TOMM20, USP5, UGDH, STAT6, CBR1, ACSL1, ATXN2L, KTI12, C7ORF27, SQSTM1, FAT1, PCSK9, BCL9L, C19ORF61, RBM25, BUB3, TES, TRIP13, EIF4ENIF1, UPF1, DLGAP5, TRIM24, C7ORF11, STAT3, APRT, ITPR2, CCNB1, TP53RK, RPS6KA3, PHF19, RPS6KA1, HNRNPUL1, MAPK14, TUBAL3, DPM1, RBM14, AACS, GOLGB1, PLXNA1, ATP6AP2, CTPS, RBM4, UBQLN4, SAE1, ACSS2, UBQLN1, TTN, MKRN2, CNOT4, GATA1, DAB2, TIA1, TARDBP, SRRM2, AKR7A2, PUM1, DNAJC7, MLST8, PUM2, SAR1A, RBM12, TOR1AIP2, SATB2, DTL, YTHDF2, RRP8, RNASEH2A, STK4, EP300, PRCC, TIAL1, BMP2K, RNF138, DSP, AKAP8, XRN1, BAT2, GNE, FN3K, ITGB1, RABGGTB, DAZAP1, HNRNPM, TUBB, DOCK2, TCERG1, ALB, METTL1, ORC6L, ZNF598, UBAP2L, CCDC21, RUNX1, EWSR1, EEF1A1, RAB8A, SMG7, AKAP8L, CASC3, HNRNPH3, HNRNPH2, EIF4E, DSG1, RAP1B, AHCYL1, DNAJB4, XPO7, TRIP6, DCXR, DNAJB6, NAMPT, PTGES2, CXORF38, KIAA0913, YLPM1, PPIP5K2, PPIP5K1, TTLL12, MEN1, FUBP1, CASP6, FUBP3, FAM83A, MCM7, CTU1, GRWD1, EDC3, PRMT6, RHOA, RHOC, YRDC, RANBP1, CSK, TUBA1B, TUBA1C, KDM5C, NSF, KIAA0907, ARL2, TBL1XR1, IRAK1, SSBP3, PFKL, LDB1, PFKP, IRF2BP2, IRF2BP1, PFKM, IPO8, CDK5, GAK, PRKCB, EIF4G3, SASS6, UMPS, LARP4, NCOA3, DEF6, PKM2, UBE2M, CPSF7, KHSRP, UBAP2, BTAF1, NUP98, KRT6B, DCK, ASNS, NSUN6, ALDH1A2, ATIC, SEC16A, POU2F1, KRT2, MSI2, ZNF609, KRT3, BDH2, KRT4, KDM3B, TNRC6B, EHD2, EHD4, DUS3L, KAT2A, DGCR6L, ARMC10, SAMHD1, PPP1R13L, MEMO1, RPRD2, CUL4B	283	1.74	8.77E-30
Inactive	Nucleotide-binding	CTPS, TUBB2A, TUBB2C, DTYMK, ACSS2, TTN, RAB1A, ACTG1, ILK, RALB, RAB23, VPS4A, SAR1B, SAR1A, SYK, RAB27A, AARS, STK4, PNKP, BMP2K, RAB14, TUBA4A, MAPK8, ARL8B, RAB13, RAB10, SRXN1, PRPS2, ADSS, COASY, GNAI3, PFKFB4, GNE, UBA6, ARF6, ARF5, MST4, CMPK1, TUBB, RAC2, GMPPA, EEF1A1, RAB8A, RTCD1, OXSR1, ARF1, UBA1, UBA2, ARF4, RAP1A, RHEB, RAD54L2, RAP1B, ASNA1, PPIP5K2, PPIP5K1, HELZ, PMVK, RNGTT, CKB, MTHFD1, CDC42, MCM7, ORC4L, RHOA, TUBB6, RHOC, CSK, TUBA1B, TUBA1C, TUBB3, NSF, RHOG, TUBB4, RAP2B, ARL2, NLRP7, IRAK1, RABL3, ACTA1, PFKL, ACTA2, PFKP, PFKM, IRGC, NLRP2, CDK5, GAK, PRKCB, UBE2N, PANK4, PKM2, UBE2M, RRM1, BTAF1, DCK, ASNS, DNAH6, ACSL1, KTI12, TOR1B, RAB11A, ACSL4, PAPSS1, SPATA5, EHD2, TRIP13, EHD4, UPF1, TP53RK, RPS6KA3, RPS6KA1, MAPK14, RAB35, TUBAL3, AACS	116	3.07	4.54E-26
Inactive	**GTP-binding**	ADSS, GNAI3, TUBB2A, TUBB2C, ARF6, ARF5, RAB1A, RNGTT, CDC42, TUBB, RAC2, GMPPA, RALB, RAB23, RHOA, TUBB6, RAB11A, RHOC, SAR1B, SAR1A, TUBA1B, TUBB3, RHOG, TUBA1C, TUBB4, RAB27A, ARL2, RAP2B, EEF1A1, RAB8A, RABL3, IRGC, ARF1, RAB35, ARF4, RAB14, TUBAL3, TUBA4A, RAP1A, RHEB, RAP1B, RAB13, ARL8B, RAB10	**44**	**5.97**	**2.14E-18**
Inactive	Cytoplasm	S100A8, PDLIM7, RBM4, UBQLN4, UBQLN2, ACSS2, TTN, UBQLN1, CNOT4, ACTG1, GSTM2, FAM125A, RAVER2, RAVER1, SULT1A1, SULT1A3, AKR7A2, PUM1, OGT, PUM2, MLST8, NQO1, CAPNS1, DTL, AARS, LYPLA2, DCTN5, DCTN3, STK4, JUP, SCYL2, KRT14, DSP, ARL8B, SIP1, XRN1, SRXN1, BAT2, SRI, COASY, CRTC2, ADSS, MOB2, GNE, HMGCS1, IGF2BP2, ARF5, MST4, CMPK1, PXN, DAZAP1, DOCK2, RAC2, NMRAL1, SNF8, POTEF, EWSR1, DVL2, EEF1A1, DVL3, NPLOC4, IKZF1, SMG7, AKAP8L, CASC3, HNRNPH2, ARF1, RAP1B, ASNA1, TRIP6, XPO7, DNAJB4, ZNF385A, DNAJB6, NAMPT, HTATIP2, PTGES2, TSG101, ESD, PPIP5K2, PPIP5K1, COPS8, CKB, MTHFD1, CASP6, SMAP2, CTU1, PRMT7, EDC3, RHOA, QKI, CSK, NSF, TOMM34, ARL2, ELP2, ZCCHC11, AIFM2, ACTA1, ACTA2, IPO8, CDK5, NLRP2, GAK, PRKCB, LAP3, PANK4, SASS6, NCOA3, DEF6, PKM2, RRM1, KHSRP, RBM38, CARM1, SSU72, ADH5, STAT6, ALDH1A2, CBR1, SH3GLB2, SQSTM1, SH3GLB1, SPRR2A, PAFAH1B3, MSI2, BDH2, TNRC6B, SPATA5, RBM25, TES, RBM22, EIF4ENIF1, UPF1, DLGAP5, CAPN2, PPP1R13L, C7ORF11, STAT3, CAPN1, APRT, CCNB1, ITPA, MAPK14, PTP4A2, AACS, RBM14, VPS25	147	1.97	6.30E-15
Inactive	Methylation	ACTG1, CDC42, TUBB, ATXN2L, RAC2, GORASP2, PRMT6, RALB, RAB23, RHOA, QKI, RAB11A, RHOC, RUNX1, TUBA1B, EWSR1, RHOG, RAB27A, RAP2B, EEF1A1, RAB8A, ACTA1, ACTA2, HNRNPH3, HNRNPH2, EP300, NCOA3, HNRNPUL1, RAB14, RAP1A, RHEB, RAP1B, RAB13	33	6.09	2.05E-13
Inactive	ATP-binding	CTPS, DTYMK, PPIP5K2, PPIP5K1, HELZ, ACSS2, PMVK, TTN, CKB, MTHFD1, ACTG1, MCM7, ORC4L, ILK, VPS4A, CSK, NSF, SYK, NLRP7, IRAK1, ACTA1, PFKL, ACTA2, AARS, PFKP, PFKM, STK4, NLRP2, CDK5, GAK, PRKCB, UBE2N, PANK4, PNKP, PKM2, UBE2M, RRM1, BMP2K, MAPK8, SRXN1, PRPS2, BTAF1, COASY, PFKFB4, GNE, DCK, UBA6, ASNS, MST4, CMPK1, DNAH6, ACSL1, KTI12, TOR1B, ACSL4, PAPSS1, SPATA5, EHD2, EHD4, TRIP13, UPF1, RTCD1, OXSR1, TP53RK, RPS6KA3, RPS6KA1, UBA1, MAPK14, UBA2, RAD54L2, ASNA1, AACS	72	2.42	1.74E-09
Inactive	Transferase	NAMPT, DPH5, DTYMK, PPIP5K2, PPIP5K1, PMVK, TTN, CKB, RNGTT, FDFT1, N6AMT1, GSTM2, NANS, CTU1, PRMT7, SULT1A1, ILK, PRMT6, SULT1A3, OGT, PCYT2, CSK, SYK, IRAK1, ZCCHC11, PFKL, PFKP, RRP8, PFKM, METTL2B, STK4, CDK5, GAK, PRKCB, PANK4, PNKP, UMPS, EP300, NCOA3, PKM2, BMP2K, MAPK8, CARM1, METTL11A, PRPS2, COASY, PFKFB4, GNE, FN3K, HMGCS1, DCK, MST4, RABGGTB, CMPK1, NSUN6, ATIC, METTL1, GMPPA, PAPSS1, KAT2A, LCMT1, OXSR1, APRT, TP53RK, METTL13, RPS6KA3, RPS6KA1, MAPK14, POLD2, DPM1, QPRT	71	2.27	4.54E-08
Inactive	**RNA-binding**	CPEB4, RBM4, IGF2BP2, CNOT4, DAZAP1, HNRNPM, RBM4B, SFRS15, CTU1, RAVER2, TIA1, TARDBP, METTL1, SRRM2, RAVER1, PUM1, QKI, MSI2, RBM47, PUM2, TNRC6B, EWSR1, RBM12, RBM22, AARS, SF1, MBNL1, CASC3, LARP4B, EIF4G3, HNRNPH3, LARP4, EIF4E, HNRNPH2, HNRNPUL1, TIAL1, KHSRP, CPSF7, RBM38, RBM14, XRN1	40	3.31	4.76E-08
Inactive	**Prenylation**	RAP2B, RAB8A, RAB1A, CDC42, RAC2, RAB35, PTP4A2, RHOA, RAB23, RALB, RAB14, RHEB, RAB11A, RAP1A, RAP1B, RHOC, RAB13, RAB10, RHOG, RAB27A	20	5.72	7.99E-07
Inactive	Nucleotide binding	ARL2, RAB8A, GNAI3, DTYMK, ARF6, ARF5, RAB1A, CDC42, ARF1, RAC2, RAB35, ARF4, RHEB, RAB13, NSF, RAB27A	16	6.67	5.83E-06

Supplementary Table 2 (cont.) | Functional enrichment analysis of proteins differentially enriched in integrin adhesion complexes.

Cluster[1]	Term[2,3]	Proteins[4]	Count (proteins per term)	Fold enrichment	Corrected *P* value
Inactive	**GTP binding**	EEF1A1, RAB8A, GNAI3, TUBB2A, RAB1A, CDC42, RAC2, GNB1, RAB35, ARF4, RHEB, RAB13, RAB27A	**13**	**8.66**	**9.76E-06**
Inactive	P-loop	ARL2, RAB8A, GNAI3, DTYMK, ARF6, ARF5, RAB1A, CDC42, ARF1, RAC2, RAB35, ARF4, RHEB, RAB13, NSF, RAB27A	16	6.16	1.77E-05
Inactive	**Kinase**	COASY, PFKFB4, GNE, PRKAG1, FN3K, DTYMK, DCK, PPIP5K2, PPIP5K1, TTN, PMVK, MST4, CMPK1, CKB, ILK, PAPSS1, CSK, SYK, DVL3, IRAK1, PFKL, PFKP, PFKM, OXSR1, STK4, CDK5, GAK, PRKCB, TP53RK, PANK4, PNKP, RPS6KA3, RPS6KA1, MAPK14, PKM2, BMP2K, MAPK8, PRPS2	**38**	**2.46**	**3.10E-04**
Inactive	Multifunctional enzyme	MTHFD1, PNKP, COASY, UMPS, PFKFB4, ATIC, GNE, TSTA3, PAPSS1, RNGTT, FDFT1	11	7.01	1.24E-03
Inactive	Protein transport	NUP98, TSG101, VPS37B, ARF6, ARF5, RAB1A, FAM125A, SEC16A, SNF8, RAB23, VPS4A, RAB11A, SAR1B, SAR1A, NSF, EIF4ENIF1, RAB8A, IPO8, C18ORF55, NUPL1, ARF1, RAB35, ARF4, TOMM20, RAB14, RAB13, RAB10, XPO7, VPS25	29	2.67	1.87E-03
Inactive	**Prenylated cysteine**	CDC42, RAB8A, RAC2, RAB35, RAB13, RAB1A, RAB27A	7	13.58	3.25E-03
Inactive	Ligase	ADSS, CTPS, RTCD1, AARS, UBA6, SAE1, ASNS, ACSS2, RNF214, CNOT4, MKRN2, UBE2N, MTHFD1, UHRF1, ACSL1, UBA1, UBA2, UBE2M, RNF138, ACSL4, AACS	21	3.07	7.05E-03
Inactive	**Phosphotransferase**	PFKFB4, PFKL, DTYMK, PFKP, DCK, PFKM, CDK5, PRKCB, RPS6KA3, RPS6KA1, MAPK14, PKM2, ILK, MAPK8, CSK, SYK	16	3.55	1.79E-02
Inactive	Palmoplantar keratoderma	KRT9, JUP, KRT6B, DSG1, KRT14, DSP	6	14.09	1.80E-02
Inactive	Monomer	RPS6KA3, CBR1, RPS6KA1, PKM2, ARF4, FDXR, RHEB	7	10.08	2.02E-02
Inactive	Magnesium	ADSS, TTN, MST4, ITGAM, FDFT1, ACSL1, RHOA, SAR1B, ACSL4, NSF, IRAK1, PFKL, PFKP, PDXP, PFKM, OXSR1, STK4, LAP3, RPS6KA3, ITPA, RPS6KA1, PKM2, RHEB, PRPS2, SRXN1	25	2.52	2.31E-02
Inactive	**mRNA processing**	RBM22, RBM4, SF1, PRPF39, CASC3, RNGTT, HNRNPM, RBM4B, HNRNPH3, SRRM2, TARDBP, KHSRP, CPSF7, QKI, RBM38, SIP1, RBM14, RBM25, SSU72	18	3.09	3.02E-02
Inactive	Heterodimer	CAPNS1, S100A8, TUBB2A, ITGA5, ITGAV, TUBA4A, CAPN2, ITGB1, TUBA1B, ITGAM, CAPN1	11	4.77	3.77E-02
Unenriched	Acetylation	XRCC5, LDHB, LDHA, PDLIM5, WDR82, BTK, NONO, RBM8A, RPLP0, U2AF1, RPL11, RPL12, EIF2B3, RAN, PTBP1, JUP, MAPK1, SPAG9, NME2, KRT19, RPS18, PPP1CA, RPS19, KRT18, RPS16, RPS17, RFC2, RPS11, MCTS1, NEK7, ALDOA, HSD17B10, FIP1L1, CHCHD3, DUSP12, TAGLN2, ACP1, ACAT1, HNRNPA3, C1ORF57, IDH2, RPS20, PRPF40A, HNRNPAB, RPSA, SRP54, LGALS1, PHB, GARS, DDX1, EPRS, HBA1, DDX5, RPS5, HNRNPA1, HNRNPA0, VDAC1, WDR61, LASP1, NIPSNAP1, NUTF2, GNB2L1, RAB5C, TSNAX, TCOF1, C22ORF28, DGCR14, YBX1, LSM14A, ACTR2, COPB2, RRP1B, GBAS, ATP5O, RBM42, SLC25A5, UFD1L, PIK3C2A, CCT6A, DECR1, CLIC1, HNRPDL, GNB2, ROD1, CLIC4, EEF1G, FARSA, THOC3, POLR2H, LIMS1, NOB1, LANCL1, NUFIP2, POLR2A, NUP214, DDX19A, PCMT1, MARS, MAT2A, CSNK2B, MACROD1, SGTA, EEF1E1, PSMD10, PHGDH, YWHAQ, HSPA4L, USP48, DNM2, MMS19, RALY, NCBP1, ZC3HAV1, IQGAP2, MTHFD1L, CBFB, AIP, PPP4C, DARS, HNRNPA2B1, CORO7, PPP1CB, PSMA2, ADRM1, PSMA1, PSMA4, HSPB1, EDF1, PDCD6IP, RNF20, XRN2, PRPS1, CHMP2A, CNBP, SRM, KARS, TTC4, SPC25, PSMB6, PSMB1, ARPC3, DDX3X, ZNF326, HNRNPF, PSMB2, DNAJA1, HNRNPD, EIF3I, HIST2H3A, WDR5, SAP18, SMG1, AK2, C8ORF33, PSMC6, PPIH, HDAC1, PPIA, POLD1, EIF4H, SEC13, HSPD1, DNAJB1, HNRNPH1, EIF4E2, HSP90AB1, METAP1, PRDX5, PRDX2, CAD, BUD31, RPS3, ACTR1A, MAGOHB, IMPDH2, KHDRBS1, HSP90AA1, ACADM, SSBP1, G3BP1, NDUFC2, CDK6, MCM3, CDK4, MCM4, MCM5, MCM6, SARNP, EIF4G1, EIF4G2, UCHL5, HBG1, HBG2, UBE2S, GSTP1, SEC23B, KRT6C, VPS29, KRT6A, VIM, HAT1, VARS, ANXA7, RPA2, PSMF1, KRT8, NUDT21, HSPA4, CNN2, UCK2, GSTO1, GAPDH, GTF3C4, NDUFA4, PDCD10, MSH2, PHB2, ANXA11, HBZ, LSM12	220	4.19	1.41E-88
Unenriched	Phosphoprotein	XRCC5, LDHB, LDHA, HRNR, SEC31A, PDLIM5, STAT5A, KPRP, WDR82, BTK, NONO, WDR74, DDX17, RBM8A, RPLP0, SFRS9, U2AF1, PQBP1, RPL11, RPL12, NUP35, CDCA5, CCAR1, RAN, RCOR1, PTBP1, JUP, MAPK1, SPAG9, KRT19, NME2, PPP1CA, KRT18, KRT17, RPS17, RPS14, CMIP, RPS10, PELO, RPS11, MCTS1, NEK7, FUS, GLG1, ALDOA, FIP1L1, CHCHD3, FLG2, IGF2BP1, RCC1, TAGLN2, ACP1, HNRNPA3, RAC1, RPS20, PRPF40A, HNRNPAB, RPSA, PHB, LGALS1, DDX1, GARS, EPRS, NDC80, HBA1, DDX5, HNRNPA1, RPS5, HNRNPA0, VDAC1, SYNE1, RPL18A, NUP62, LASP1, GNB2L1, TCOF1, TSNAX, PDIA6, C22ORF28, DGCR14, YBX1, LSM14A, ANKRD17, COPB2, RRP1B, USP10, CTBP1, NDUFB10, UFD1L, SLC25A5, PIK3C2A, CCT6A, EXOSC1, CLIC1, GTF2B, HNRPDL, GNB2, FANCD2, CDK11A, EEF1G, FARSA, SRCAP, RBM33, NOB1, NUFIP2, MLF2, POLR2A, SRRT, NUP214, PPP2CB, TRIP11, ACSL3, ETFA, MARS, MAT2A, CSNK2B, ELAVL1, STAT1, SGTA, TRIM21, CAPRIN1, NRAS, CCNB2, TFRC, RPL23, PHGDH, YWHAQ, HSPA4L, USP48, TRMT112, DNM2, MMS19, RALY, NCBP1, ZC3HAV1, RBM3, IQGAP2, RBM7, RPS27L, CBFB, AIP, NUDCD2, CGGBP1, DARS, BAIAP2, HNRNPA2B1, CORO7, PPP1CB, PSMA2, ADRM1, PSMA1, PSMA4, TELO2, EDF1, HSPB1, PDCD6IP, RNF20, DCUN1D5, XRN2, CHMP2A, AGFG1, LIN28B, KARS, TTC4, PSMB4, PSMB7, DDX3X, ARPC3, PSMB1, ZNF326, HNRNPF, NUMB, PRKRA, HNRNPD, DNAJA1, EIF3I, DNAJA2, HIST2H3A, CSTF3, WDR5, SMG1, QRICH1, PSMC6, NOSIP, HDAC1, PPIA, POLD1, PPID, EIF4H, SEC13, HSPD1, HNRNPH1, HSP90AB1, METAP1, CAD, RPS3, PRMT1, PCBP1, PCBP2, MAGOHB, CAT, TUBA1A, CLINT1, IMPDH2, PSMD9, KHDRBS1, HSP90AA1, SSBP1, NUP88, HMBS, G3BP1, CDK6, MCM3, CDK4, MCM4, MCM6, SARNP, EIF4G1, ATP6V1A, EIF4G2, CPSF6, CPSF4, HBG2, CPSF2, GSTP1, KRT6C, KRT6A, VIM, HAT1, CTNND1, COMT, VARS, STUB1, RNF126, TYMS, RPA2, PSMF1, KRT5, KRT8, NUDT21, KRT1, LARS, HSPA4, CNN2, UCK2, YES1, GAPDH, NSUN2, GTF3C4, GIGYF1, PSTPIP2, MSH2, ATXN2, HSP90B1, KRT77, PHB2, ANXA11, LSM12, HBE1, RNF40	260	1.80	8.50E-31
Unenriched	**RNA-binding**	RALY, EIF2C2, ZC3HAV1, RBM3, TIMM50, RBM7, YBX1, RPS3, NONO, ANKRD17, DDX17, RBM8A, PCBP1, PCBP2, SFRS9, U2AF1, MAGOHB, RPL11, RPL12, KHDRBS1, RBM42, PTBP1, G3BP1, HNRNPA2B1, EXOSC1, SARNP, HNRPDL, EIF4G1, RPS18, ROD1, THOC6, CPSF6, RPS11, CPSF4, THOC3, CPSF2, SNRPE, FUS, FIP1L1, RBM33, IGF2BP1, LIN28B, NUFIP2, HNRNPA3, DDX3X, DDX19A, HNRNPF, NUDT21, PRKRA, HNRNPD, NSUN2, HNRNPAB, MARS, SRP54, DDX1, EPRS, ELAVL1, DDX5, HNRNPA1, TRIM21, HNRNPA0, RPL22, EIF4H, CIRBP, HNRNPH1, EIF4E2	66	6.14	8.54E-30
Unenriched	Cytoplasm	NCBP1, LDHB, EIF2C2, LDHA, SEC31A, ZC3HAV1, RBM3, STAT5A, KPRP, BTK, AIP, RAE1, RBM8A, RPLP0, TUBG1, PPP4C, CCAR1, NUDT4, DARS, RAN, BAIAP2, HNRNPA2B1, CORO7, LYPLA1, PPP1CB, PSMA2, JUP, SPAG9, ADRM1, PSMA1, NME2, PPP1CA, RPS18, KRT18, DCAF7, KRT17, PSMA4, TELO2, EDF1, HSPB1, CMIP, RPS10, MVK, PELO, PDCD6IP, MCTS1, NEK7, ACTBL2, CNBP, MAGEA1, IGF2BP1, RCC1, ACP1, LIN28B, KARS, PSMB5, PEF1, PSMB4, PSMB7, PSMB6, DDX3X, ARPC3, PSMB1, PSMB2, PRKRA, HNRNPD, EIF3I, HNRNPAB, RPSA, SRP54, SAP18, SMG1, HNRNPA1, NOSIP, PSMC6, PPIH, SYNE1, NUP62, PPIA, LASP1, EIF4H, PPID, CIRBP, NUTF2, DNAJB1, HSPD1, HSP90AB1, TSNAX, PRDX5, PRDX2, CAD, YBX1, RPS3, LSM14A, ACTR2, COPB2, ANKRD17, ACTR1A, PCBP1, PCBP2, CLINT1, AKR1C1, CTBP1, RBM42, HSP90AA1, PIK3C2A, UFD1L, HMBS, G3BP1, CLIC2, CCT6A, CLIC1, HNRPDL, GNB2, CDK11A, FARSA, VPS29, NXT1, LANCL1, CTNND1, HAT1, COMT, STUB1, NUFIP2, MLF2, SRRT, DDX19A, PPP2CB, KRT8, LARS, PCMT1, HSPA4, GSTO1, YES1, GAPDH, NSUN2, TRIP11, MARS, PSTPIP2, ELAVL1, STAT1, CAPRIN1, TRIM21, UBL5, ATXN2, PHB2, EEF1E1, ANXA11, YWHAQ, HSPA4L, USP48, DUSP9, DNM2	153	2.31	2.37E-23
Unenriched	Protein biosynthesis	VARS, KARS, RPS3, RPLP0, LARS, RPL11, EIF3I, RPL12, RPS20, EIF2B3, MARS, RPSA, DARS, GARS, EPRS, RPS5, EIF4G1, EIF4G2, RPS18, RPS19, RPL23, RPS16, RPL18A, RPL22, RPS17, RPS14, EIF4H, EEF1E1, EEF1G, RPS10, RPS11, FARSA, EIF4E2	33	8.82	2.61E-18
Unenriched	Nucleus	MMS19, RALY, XRCC5, NCBP1, EIF2C2, ZC3HAV1, RBM3, STAT5A, WDR82, MED20, CBFB, BTK, WDR74, NONO, DDX17, RAE1, CGGBP1, RBM8A, SFRS9, U2AF1, PQBP1, NUP37, NUP35, PPP4C, CDCA5, RAN, RCOR1, HNRNPA2B1, PTBP1, PPP1CB, RFC5, PSMA2, ADRM1, PSMA1, NME2, PPP1CA, RPS19, DCAF7, RFC2, PSMA4, TELO2, EDF1, HSPB1, CMIP, PELO, SNRPE, RNF20, XRN2, FUS, FIP1L1, AGFG1, IGF2BP1, DUSP12, RCC1, LIN28B, KARS, PSMB5, HNRNPA3, SPC25, PSMB4, PSMB7, PSMB6, DDX3X, PSMB1, ZNF326, HNRNPF, PSMB2, HNRNPD, NUP54, HNRNPAB, PRPF40A, HIST2H3A, RPSA, CSTF3, SRP54, WDR5, SAP18, SMG1, NDC80, DDX5, HNRNPA1, HNRNPA0, NOSIP, PSMC6, PPIH, SYNE1, NUP62, HDAC1, POLD1, CIRBP, DNAJB1, HNRNPH1, TSNAX, TCOF1, TIMM50, BUD31, DGCR14, RPS19BP1, YBX1, ANKRD17, PRMT1, ASH2L, RRP1B, PCBP1, PCBP2, MAGOHB, KHDRBS1, CTBP1, RBM42, PIK3C2A, NUP88, UFD1L, G3BP1, CLIC1, EXOSC1, MCM3, MCM4, GTF2B, MCM5, MCM6, SARNP, HNRPDL, FANCD2, CLIC4, CDK11A, CPSF6, CPSF4, THOC3, SRCAP, CPSF2, POLR2H, NXT1, NOB1, LANCL1, WBSCR22, CTNND1, HAT1, NUFIP2, MLF2, POLR2A, RPA2, SRRT, NUP214, DDX19A, NUDT21, PPP2CB, SUPT4H1, GAPDH, NSUN2, GTF3C4, MSH2, STAT1, TRIM21, PHB2, EEF1E1, ANXA11, HSPA4L, USP48, RNF40	159	1.86	5.81E-15
Unenriched	Ribonucleoprotein	RALY, EIF2C2, RPS27L, RPS3, LSM14A, HNRNPA3, MRPL12, HNRNPF, PCBP1, RPLP0, PCBP2, HNRNPD, RPL11, RPL12, RPS20, HNRNPAB, RPSA, SRP54, HNRNPA2B1, HNRNPA1, RPS5, TRIM21, HNRNPA0, RPS18, RPS19, RPL23, RPL18A, RPS16, RPL22, RPS17, RPS14, RPS10, RPS11, HNRNPH1, SNRPE	35	6.30	7.28E-15
Unenriched	Host-virus interaction	VIM, KARS, PSMB5, PSMB4, ANKRD17, PSMB7, PSMB6, DDX3X, PSMB1, RPLP0, KRT8, PSMB2, CTBP1, SLC25A5, RAN, RCOR1, STAT1, GTF2B, HNRNPA1, SGTA, VDAC1, EIF4G1, MAPK1, KRT19, KRT18, HDAC1, TFRC, PPIA, PSMA4, EIF4H, CPSF4, HSPD1, PDCD6IP, SRCAP	34	5.99	8.15E-14
Unenriched	**mRNA processing**	RALY, NCBP1, FIP1L1, PPIL1, DGCR14, YBX1, NONO, HNRNPA3, RBM8A, HNRNPF, SFRS9, NUDT21, U2AF1, MAGOHB, PPIL3, PRPF40A, CSTF3, PTBP1, HNRNPA2B1, DDX5, HNRNPA1, PPIH, THOC6, CPSF6, CPSF4, HNRNPH1, CPSF2, SNRPE, THOC3, XRN2	30	5.79	2.09E-11
Unenriched	Proteasome	PSMB5, PSMA2, PSMB4, PSMA1, ADRM1, PSMF1, PSMC6, PSMB7, PSMB6, PSMB1, PSMA4, PSMD10, PSMB2, UCHL5, PSMD8, PSMD9	16	14.35	6.38E-11
Unenriched	Ribosome	RPSA, RPS5, RPS3, RPS18, RPS19, RPL18A, RPL23, RPS16, RPS17, RPL22, RPS14, RPLP0, RPS10, RPL11, RPS11, RPS20, RPL12	17	11.70	2.96E-10
Unenriched	**mRNA splicing**	RALY, NCBP1, PPIL1, PTBP1, HNRNPA2B1, DDX5, DGCR14, HNRNPA1, YBX1, NONO, HNRNPA3, PPIH, RBM8A, HNRNPF, SFRS9, THOC6, U2AF1, MAGOHB, PPIL3, HNRNPH1, THOC3, SNRPE, PRPF40A	23	5.53	7.62E-08
Unenriched	**mRNA transport**	NCBP1, AGFG1, NUP88, HNRNPA1, NUP214, NUP62, RBM8A, DDX19A, THOC6, MAGOHB, NUP37, NUP54, NUP35, THOC3	14	9.50	7.84E-07
Unenriched	Threonine protease	PSMA2, PSMB5, PSMB4, PSMA1, PSMB7, PSMB6, PSMB1, PSMA4, PSMB2	9	22.60	8.44E-07
Unenriched	Methylation	KHDRBS1, FUS, HIST2H3A, SLC25A5, RBM3, HNRNPA2B1, G3BP1, ELAVL1, DDX5, HNRNPA1, HNRNPA0, HNRNPA3, HNRPDL, NRAS, PPP2CB, RAC1, HNRNPD, KRT1, HNRNPH1, PPP4C, GAPDH, HNRNPAB	22	4.57	6.45E-06
Unenriched	Ribosomal protein	RPSA, RPS27L, RPS5, RPS3, RPS18, RPS19, MRPL12, RPS16, RPL18A, RPL23, RPS17, RPL22, RPS14, RPLP0, RPS10, RPL11, RPS11, RPS20, RPL12	19	5.08	1.52E-05
Unenriched	ATP-binding	XRCC5, HSP90AB1, CAD, MTHFD1L, BTK, ACTR2, DDX17, ACTR1A, HSP90AA1, DARS, PIK3C2A, G3BP1, CDK6, CCT6A, MCM3, CDK4, MCM4, MCM5, MCM6, RFC5, MAPK1, ATP6V1A, NME2, RFC2, CDK11A, MVK, FARSA, UBE2S, SRCAP, NEK7, PRPS1, ACTBL2, VARS, KARS, GALK1, UBE2D3, C1ORF57, DDX3X, DDX19A, LARS, HSPA4, UCK2, YES1, ACSL3, MARS, MAT2A, MSH2, DDX1, GARS, SMG1, AK2, EPRS, DDX5, HSP90B1, PSMC6, HSPA4L, HSPD1	57	2.16	2.43E-05
Unenriched	Nucleotide-binding	XRCC5, HSP90AB1, RAB5C, CAD, MTHFD1L, BTK, ACTR2, DDX17, ACTR1A, TUBG1, TUBA1A, ARL1, HSP90AA1, DARS, PIK3C2A, RAN, G3BP1, CDK6, CCT6A, MCM3, CDK4, MCM4, MCM5, MCM6, RFC5, MAPK1, ATP6V1A, NME2, RFC2, CDK11A, MVK, FARSA, UBE2S, SRCAP, NEK7, PRPS1, ACTBL2, VARS, KARS, GALK1, UBE2D3, C1ORF57, DDX3X, DDX19A, RAC1, LARS, HSPA4, UCK2, YES1, ACSL3, MARS, SRP54, MAT2A, MSH2, DDX1, GARS, EPRS, SMG1, AK2, DDX5, NRAS, HSP90B1, PSMC6, HSPA4L, HSPD1, DNM2	66	1.97	5.52E-05
Unenriched	Oxygen carrier	HBZ, HBQ1, HBG1, HBG2, HBA1, HBE1	6	37.67	5.89E-05
Unenriched	Proteinase	PSMA2, PSMB5, PSMB4, PSMB6, PSMB1, PSMA4, PSMB2	7	20.68	2.23E-04
Unenriched	**Spliceosome**	RALY, PPIL1, HNRNPA2B1, DDX5, HNRNPA1, DGCR14, HNRNPA3, PPIH, RBM8A, HNRNPF, U2AF1, PPIL3, HNRNPH1, SNRPE	14	5.86	2.88E-04
Unenriched	Blood	HBZ, HBQ1, HBG1, HBG2, HBA1, HBE1	6	27.39	4.62E-04
Unenriched	Oxygen transport	HBZ, HBQ1, HBG1, HBG2, HBA1, HBE1	6	25.11	7.80E-04
Unenriched	Intermediate filament	KRT6C, KRT6A, KRT19, KRT18, KRT77, KRT17, KRT5, KRT16, KRT8, VIM, KRT1	11	7.08	1.18E-03
Unenriched	Stress response	HSP90AB1, HSP90AA1, RBM3, HSPA4L, HSPB1, CIRBP, HSPA4, DNAJB1, HSPD1, SERPINH1	10	7.61	2.19E-03
Unenriched	Aminoacyl-tRNA synthetase	DARS, LARS, GARS, EPRS, FARSA, VARS, KARS, MARS	8	10.30	3.76E-03
Unenriched	Erythrocyte	HBZ, HBQ1, HBG1, HBG2, HBA1, HBE1	6	15.07	1.33E-02
Unenriched	Metalloprotein	PPP1CA, MAT2A, HBZ, PPP2CB, HBQ1, HBG1, HBG2, HBA1, CAT, HBE1, PPP4C, PPP1CB	12	4.86	1.36E-02
Unenriched	WD repeat	SEC31A, GNB1L, WDR5, CORO7, WDR82, WDR92, WDR74, COPB2, DCAF7, GNB2, WDR61, RAE1, THOC6, SEC13, EIF3I, NUP37, GNB2L1, THOC3	18	3.28	1.47E-02
Unenriched	Translation regulation	LSM14A, SARNP, EIF4G1, NCBP1, EIF4G2, EIF2C2, RBM8A, IGF2BP1, EIF4E2	9	6.75	1.92E-02
Unenriched	Cell cycle	KHDRBS1, RAN, CDK6, NDC80, CDK4, MCM3, RCC1, PPP1CB, MCM6, MAPK1, SPC25, PPP1CA, CCNB2, KRT18, FANCD2, CDK11A, ANXA11, PELO, NUP37, PDCD6IP, CDCA5, UBE2S, MCTS1, CCAR1	24	2.61	1.95E-02
Unenriched	Heterotetramer	KRT5, HBZ, KRT1, CSNK2B, HBQ1, HBG1, HBG2, HBA1, HBE1	9	6.55	2.37E-02
Unenriched	**Cell division**	RAN, CDK6, NDC80, RCC1, CDK4, PPP1CB, MCM5, SPC25, PPP1CA, CCNB2, CDK11A, ANXA11, PELO, NUP37, PDCD6IP, CDCA5, UBE2S	**17**	**3.23**	**2.97E-02**
Unenriched	Nuclear pore complex	NUP214, NUP62, NUP88, DDX19A, NUP37, NUP54, NUP35	7	8.79	4.59E-02

[1]Proteins identified by MS and assigned to active, inactive or unenriched clusters (Supplementary Table 1) were subjected to analysis of enrichment of SwissProt Protein Information Resource keywords.
[2]Terms with fold enrichment ≥1.5, Bonferroni-corrected *P* value <0.05, EASE score (modified Fisher's exact probability) <0.05 and at least two proteins per term were considered significantly overrepresented.
[3]Terms highlighted in the text are indicated in bold font. Data associated with terms significantly overrepresented in all three clusters are indicated in grey font.
[4]Proteins are indicated by gene name for clarity.

Supplementary Table 3 | Effect of reduced expression of selected +TIPs and other proteins on cortical microtubule targeting.

Gene name[1]	Protein name[1]	Alias(es)	Expression knockdown (%)[2]			Effect on MT localisation to cell periphery[3]
			HFF	K562	U2OS	
ARHGEF2	Rho/rac guanine nucleotide exchange factor 2	GEF-H1	0	70	0	–
CKAP5	Cytoskeleton-associated protein 5	Ch-TOG, XMAP215	70	75	n.d.	–*
MACF1	Microtubule-actin cross-linking factor 1	ABP620, ACF7	80	0	n.d.	–
MAPRE1	Microtubule-associated protein RP/EB family member 1	EB1	90	n.d.	n.d.	–
CDK9	Cyclin-dependent kinase 9	CDC2L4, TAK	40	n.d.	n.d.	–
FLNA	Filamin-A	ABP-280	0	n.d.	90	–
GNB2L1	Guanine nucleotide-binding protein subunit beta-2-like 1	HLC-7, RACK1	50	n.d.	50	–*
GRB2	Growth factor receptor-bound protein 2	ASH	70	n.d.	n.d.	–
LGALS1	Galectin-1	Galaptin, HLBP14	90	n.d.	n.d.	–
MPG	DNA-3-methyladenine glycosylase	ADPG	85	n.d.	n.d.	–
PLEC	Plectin	HD1	80	n.d.	n.d.	+
SNRPB	Small nuclear ribonucleoprotein-associated proteins B and B'	Sm-B/B'	70	n.d.	n.d.	–*
EZR	Ezrin	Cytovillin, VIL2	85	n.d.	n.d.	–
ILK	Integrin-linked protein kinase	–	n.d.	n.d.	90	++
IQGAP1	Ras GTPase-activating-like protein IQGAP1	p195	40	n.d.	90	–
PHLDB2	Pleckstrin homology-like domain family B member 2	LL5β	0	n.d.	70	–
RCC2	Regulator of chromosome condensation 2	TD-60	90	n.d.	90	+

[1]Quantitative MS data for all proteins identified in the active integrin data set were mapped onto a merged, multi-source interactome database to generate an active integrin interactome (see methods for details). From the identified proteins reported to interact directly with or indirectly within three interactions of β1 integrin (the three-hop interaction network), proximity subnetworks seeded with β1 integrin or +TIPs were generated *in silico*. Proteins situated at the intersections of β1 integrin and +TIPs one- or two-hop subnetworks were identified as candidates that may functionally link active integrin and +TIPs. Expression of selected +TIPs (red bar), candidate connecting proteins from interaction network analysis (blue bar) and other potential connecting or active integrin–enriched proteins (green bar) was depleted by siRNA in a range of cell types. Knockdown of ILK expression has previously been shown to affect microtubule localisation at the cell periphery[5].

[2]Experiments were performed in HFF, K562 and U2OS cells. 0, expression knockdown not effective; n.d., not done.

[3]Forty-eight to seventy-two hours after siRNA transfection, cells were spread on FN for 1 h, fixed and stained for α-tubulin to assess the effect of expression knockdown on microtubule targeting to the cell cortex. ++, substantial effect (microtubules did not reach the cell periphery in most cells); +, slight effect (microtubules did not reach the cell periphery in some cells); −, no effect (microtubules did reach the cell periphery); *, expression knockdown produced off-target effects (such as reduced cell spreading or cell viability).

Supplementary Table 4 | Statistics source data.

(**a**) Statistics source data for Figure 4b.

	MT density[1,2]		
Cell repl.	FN	Stim.	Inhib.
1	2.89 (9)	5.13 (15)	1.00 (10)
2	5.00 (11)	6.36 (11)	0.42 (12)
3	2.67 (6)	7.71 (14)	0.40 (15)
4	4.67 (3)	6.79 (19)	0.38 (29)
5	3.63 (8)	5.85 (13)	1.00 (24)
6	2.62 (13)	8.36 (11)	0.69 (29)
7	2.43 (21)	5.40 (15)	0.55 (44)
8	2.85 (13)	6.00 (9)	0.30 (30)
9	3.44 (18)	6.88 (8)	–
10	–	8.56 (16)	–
Mean	3.35	6.70	0.59
s.d.	0.93	1.19	0.28
n	9	10	8
P (NGD)[3,4]	0.36	0.60	0.45

[1]Number of MTs per 5 × 2-µm region at cell periphery
[2]Number of regions quantified per cell indicated in parentheses
[3]*P* value (H_1, NGD, non-Gaussian distribution)
[4]D'Agostino & Pearson omnibus normality test, α = 0.05

	Comparison		
Statistic[5]	FN vs stim.	Stim. vs inhib.	FN vs inhib.
Mean difference	−3.35	6.11	2.76
95% CI of difference	−4.40 to −2.30	5.03 to 7.20	1.65 to 3.88
Corrected *P* value	< 0.0001	< 0.0001	< 0.0001

[5]One-way analysis of variance, Tukey's multiple comparisons test, α = 0.05

(**b**) Statistics source data for Figure 4c.

	MT density[1,2], before NOC			MT density[1,2], after washout		
Cell repl.	FN	Stim.	Inhib.	FN	Stim.	Inhib.
1	3.00 (36)	3.05 (20)	0.58 (19)	2.48 (31)	2.62 (21)	0.10 (20)
2	3.50 (32)	2.41 (27)	0.43 (23)	2.53 (36)	1.60 (20)	0.00 (20)
3	3.47 (34)	3.47 (19)	0.61 (23)	3.16 (32)	2.52 (31)	0.09 (34)
4	–	–	0.59 (34)	–	–	0.00 (21)
Mean	3.32	2.98	0.55	2.72	2.25	0.05
s.d.	0.28	0.54	0.07	0.38	0.56	0.05
n	3	3	4	3	3	4
P (NGD)[3,4,5]	ND	ND	ND	ND	ND	ND

[1]Number of MTs per 5 × 2-µm region at cell periphery
[2]Number of regions quantified per cell indicated in parentheses
[3]*P* value (H_1, NGD, non-Gaussian distribution)
[4]D'Agostino & Pearson omnibus normality test, α = 0.05
[5]ND, not determined (too few values to compute normality)

	Comparison, before NOC			Comparison, after washout			Compar., FN	Compar., stim.	Compar., inhib.
Statistic[6]	FN vs stim.	Stim. vs inhib.	FN vs inhib.	FN vs stim.	Stim. vs inhib.	FN vs inhib.	Before vs after	Before vs after	Before vs after
Mean difference	0.35	2.42	2.77	0.48	2.20	2.68	0.60	0.73	0.51
95% CI of difference	−0.58 to 1.27	1.56 to 3.29	1.91 to 3.64	−0.45 to 1.40	1.33 to 3.06	1.81 to 3.54	−0.32 to 1.53	−0.19 to 1.66	−0.30 to 1.31
Corrected *P* value	0.8200	< 0.0001	< 0.0001	0.5600	< 0.0001	< 0.0001	0.3300	0.1600	0.3500

[6]Two-way analysis of variance, Tukey's multiple comparisons test, α = 0.05

(**c**) Statistics source data for Figure 4d.

	MT density[1,2], DMSO		MT density[1,2], cytochalasin	
Cell repl.	Stim.	Inhib.	Stim.	Inhib.
1	3.72 (18)	0.08 (38)	3.43 (21)	0.76 (25)
2	3.82 (17)	0.03 (31)	4.77 (30)	1.63 (24)
3	4.39 (18)	0.42 (43)	4.64 (25)	2.15 (27)
4	3.18 (17)	0.28 (32)	4.15 (26)	1.84 (25)
5	2.95 (21)	0.36 (39)	3.94 (16)	1.82 (17)
6	–	–	–	1.63 (24)
7	–	–	–	1.61 (23)
Mean	3.61	0.23	4.19	1.63
s.d.	0.57	0.17	0.54	0.43
n	5	5	5	7
P (NGD)[3,4,5]	ND	ND	ND	ND

[1]Number of MTs per 5 × 2-µm region at cell periphery
[2]Number of regions quantified per cell indicated in parentheses
[3]*P* value (H_1, NGD, non-Gaussian distribution)
[4]D'Agostino & Pearson omnibus normality test, α = 0.05
[5]ND, not determined (too few values to compute normality)

	Compar., DMSO	Compar., cyto.	Compar., stim.	Compar., inhib.
Statistic[6]	Stim. vs inhib.	Stim. vs inhib.	DMSO vs cyto.	DMSO vs cyto.
Mean difference	3.38	2.55	−0.57	−1.40
95% CI of difference	2.57 to 4.19	1.80 to 3.30	−1.38 to 0.24	−2.15 to −0.65
Corrected *P* value	< 0.0001	< 0.0001	0.2246	0.0003

[6]Two-way analysis of variance, Tukey's multiple comparisons test, α = 0.05

Supplementary Table 4 (cont.) | Statistics source data.

(**d**) Statistics source data for Figure 5a.

	MT density[1]		
**Patch**	**FN**	**Stim.**	**Inhib.**
1	32.66	166.66	23.89
2	7.23	318.47	15.82
3	147.01	237.14	23.30
4	22.43	137.33	86.57
5	27.56	79.88	77.48
6	5.05	30.53	21.73
7	7.79	36.40	36.92
8	69.07	26.34	70.63
9	5.35	28.51	19.49
10	13.28	21.63	28.95
11	324.87	43.72	17.19
12	95.64	30.33	16.37
13	331.86	156.48	33.45
14	235.51	193.51	24.20
15	50.68	27.75	36.84
16	84.05	27.43	56.43
17	92.80	120.40	11.78
18	77.19	228.66	11.40
19	10.35	91.00	20.83
20	92.58	19.29	37.34
21	418.90	46.37	57.24
22	608.84	46.37	44.03
23	248.34	42.42	46.73
24	475.57	112.11	23.03
25	325.48	53.06	32.70
26	31.77	453.29	89.83
27	73.18	62.08	78.72
28	626.04	170.86	53.77
29	42.21	311.45	79.12
30	189.94	186.80	39.89
31	180.98	187.55	37.34
32	249.38	91.16	57.24
33	234.12	114.34	44.03
34	369.53	201.33	46.73
35	248.48	43.51	23.03
36	307.22	107.45	32.70
37	124.81	83.84	89.83
38	193.56	135.02	78.72
39	56.61	227.37	53.77
40	232.97	93.96	79.12
41	202.79	80.96	39.89
42	107.07	233.50	21.48
43	26.22	48.90	17.71
44	217.85	64.64	18.13
45	104.96	388.06	18.07
46	188.83	68.32	28.46
47	36.57	95.15	18.11
48	294.35	288.45	16.06
49	52.79	45.23	23.87
50	8.04	123.81	40.47
51	42.48	223.57	46.90
52	50.67	84.88	71.68
53	310.96	149.25	122.04
54	287.07	117.84	67.70
55	319.83	369.57	21.79
56	22.07	102.20	45.98
57	171.15	107.95	11.43
58	92.55	129.33	22.82
59	102.58	145.51	10.47
60	20.35	66.14	56.35
61	34.70	28.09	109.12
62	76.86	174.38	51.50
63	19.91	33.41	271.78
64	454.45	127.50	37.38
65	33.74	258.43	12.87
66	8.62	31.14	116.95
67	200.65	218.82	33.45
68	20.09	73.61	7.91
69	66.40	128.65	72.48
70	948.28	146.60	19.30
71	5.31	20.28	78.78
72	356.60	121.65	21.01
73	667.07	105.61	36.43
74	92.29	61.74	17.42
75	206.55	69.90	82.59
76	90.54	212.28	36.87
77	158.27	200.05	9.69
78	22.57	37.79	8.93
79	344.39	77.86	10.14
80	31.62	718.35	10.02
81	196.43	132.53	54.88
82	12.87	90.12	24.33
83	182.36	261.42	6.81
84	109.94	93.81	33.40
85	101.76	88.64	20.83
86	32.79	28.23	8.15
87	81.47	83.62	11.48
88	28.41	60.02	10.62
89	60.23	104.70	22.88
90	150.57	222.95	23.78
91	64.10	208.08	57.63
92	161.77	161.19	14.59
93	342.23	80.51	34.93
94	85.40	82.73	18.90
95	10.16	196.73	20.24
96	–	64.15	15.28
97	–	56.49	25.62
98	–	348.51	–
99	–	272.87	–
100	–	223.66	–
101	–	24.10	–
102	–	53.42	–
103	–	178.54	–
104	–	32.12	–
105	–	118.69	–
106	–	82.27	–
107	–	96.28	–
108	–	45.88	–
109	–	40.39	–
110	–	182.66	–
111	–	41.75	–
112	–	196.50	–
113	–	81.41	–
114	–	38.13	–
115	–	109.67	–
116	–	76.84	–
117	–	96.75	–
118	–	36.08	–
119	–	80.36	–
120	–	93.40	–
121	–	44.11	–
122	–	37.06	–
123	–	51.94	–
124	–	47.17	–
125	–	57.51	–
Mean	158.84	123.00	40.48
s.d.	168.48	102.25	35.50
n	95	125	97
P (NGD)[2,3]	< 0.0001	< 0.0001	< 0.0001

[1]Tubulin fluorescence intensity per patch at cell periphery

[2]*P* value (H_1, NGD, non-Gaussian distribution)

[3]D'Agostino & Pearson omnibus normality test, α = 0.05

	Comparison		
**Statistic[4]**	**FN vs stim.**	**Stim. vs inhib.**	**FN vs inhib.**
Mean rank difference	−7.02	93.30	100.30
Corrected *P* value	> 0.9999	< 0.0001	< 0.0001

[4]Kruskal–Wallis test, Dunn's multiple comparisons test, α = 0.05

(**e**) Statistics source data for Figure 5c.

	MT density[1,2]			
**Cell repl.**	**FN**	**Stim.**	**Inhib.**	**Inhib. + stim.[3]**
1	3.58 (40)	2.31 (35)	0.11 (36)	2.64 (56)
2	3.50 (36)	3.64 (14)	0.32 (34)	2.03 (33)
3	4.61 (31)	2.88 (40)	0.38 (32)	3.64 (47)
4	4.00 (55)	2.00 (24)	0.22 (32)	2.36 (45)
5	5.56 (18)	2.90 (21)	0.56 (36)	1.92 (39)
6	5.06 (17)	3.03 (33)	0.85 (33)	2.95 (56)
7	3.04 (23)	2.63 (19)	0.10 (39)	3.29 (35)
8	5.63 (8)	2.31 (48)	1.08 (36)	3.00 (33)
9	4.08 (13)	4.79 (33)	0.24 (34)	2.13 (45)
10	–	2.96 (26)	0.39 (31)	4.00 (37)
11	–	1.88 (25)	0.35 (34)	–
12	–	3.75 (60)	–	–
Mean	4.34	2.92	0.42	2.80
s.d.	0.93	0.82	0.30	0.70
n	9	12	11	10
P (NGD)[4,5]	0.54	0.18	0.09	0.66

[1]Number of MTs per 5 × 2-μm region at cell periphery

[2]Number of regions quantified per cell indicated in parentheses

[3]Inhibitory:stimulatory, 1000:1

[4]*P* value (H_1, NGD, non-Gaussian distribution)

[5]D'Agostino & Pearson omnibus normality test, α = 0.05

	Comparison
**Statistic[6]**	**Inhib. vs inhib. + stim.[3]**
Mean difference	2.38 ± 0.24
95% CI of difference	1.85 to 2.90
Corrected *P* value	< 0.0001

[6]Two-tailed unpaired *t* test, Welch's correction

Supplementary References

1. Winograd-Katz, S. E., Fässler, R., Geiger, B. & Legate, K. R. The integrin adhesome: from genes and proteins to human disease. *Nat. Rev. Mol. Cell Biol.* **15,** 273–288 (2014).
2. Zaidel-Bar, R., Itzkovitz, S., Ma'ayan, A., Iyengar, R. & Geiger, B. Functional atlas of the integrin adhesome. *Nat. Cell Biol.* **9,** 858–867 (2007).
3. Ye, F., Lagarrigue, F. & Ginsberg, M. H. Talin and the modular nature of the integrin adhesome. *Cell* **156,** 1340–1340.e1 (2013).
4. Applegate, K. T. *et al.* plusTipTracker: Quantitative image analysis software for the measurement of microtubule dynamics. *J. Struct. Biol.* **176,** 168–184 (2011).
5. Wickström, S. A. *et al.* Integrin-linked kinase controls microtubule dynamics required for plasma membrane targeting of caveolae. *Dev. Cell* **19,** 574–588 (2010).
